# A comprehensive analysis of the kinetics of infection of lytic bacteriophages specific to the ESKAPE and critical pathogens

**DOI:** 10.1007/s11274-025-04762-4

**Published:** 2026-02-28

**Authors:** Olaf Bajrak, Martyna Cieślik, Andrzej Górski, Ewa Jończyk-Matysiak

**Affiliations:** 1 Bacteriophage Laboratory, Hirszfeld Institute of Immunology and Experimental Therapy, Polish Academy of Sciences (HIIET PAS), Wrocław, Poland; 2 Phage Therapy Unit, Hirszfeld Institute of Immunology and Experimental Therapy, Polish Academy of Sciences (HIIET PAS), Wrocław, Poland; 3 Department of Clinical Immunology, Medical University of Warsaw, Warsaw, Poland

**Keywords:** ESKAPE group, Lytic bacteriophage, Cycle parameters

## Abstract

Bacteriophages are ubiquitous particles that are able to infect bacterial and archaeal cells and may serve as an antimicrobial agent. Herein, a scoping review of strictly lytic bacteriophages infecting bacteria belonging to the ESKAPE group and critical pathogens is proposed in order to summarize information about their biological characteristics in vitro. The data collected herein will be useful for the rapid in vitro characterization of newly isolated phages, facilitating their likely effective use in phage therapy and allowing them to be compared with previously described phages of the same specificity. Data were obtained from original, peer-reviewed, full-length scientific articles published between 1998 and 2025, available in the PubMed database, focusing on the analysis of phage infection kinetics (adsorption rate and parameters derived from one-step growth experiments). However, additional features, including morphology of their plaques (like the presence or absence of a ‘halo’ effect), type of morphology of phage virions or lytic spectrum have also been included, in order to relate acquired data regarding to phage replication cycle kinetics to the mentioned phage characteristics.

**Supplementary Information:**

The online version contains supplementary material available at 10.1007/s11274-025-04762-4.

## Introduction

In February 2017, the World Health Organization (WHO) presented a list of the most dangerous multidrug resistant (MDR) pathogens, with particular focus on those emerging in healthcare facilities worldwide, which was updated in 2024 (WHO Bacterial Priority Pathogens List 2024). MDR bacteria are estimated to be responsible for even 10 million deaths worldwide annually by 2050 (Mączyńska et al. 2023). The emergence of such pathogens is associated with the excessive use of antibiotics in the medical and food industries, as well as with stagnation in the antibiotic development process (Sommer et al. 2017). The WHO report identifies bacteria classified as “critical” pathogens, currently regarded as some of the most significant global health threats (De Oliveira et al. 2020). Many species from this group also possess the ability to form biofilm, which substantially reduces treatment effectiveness (Paganelli et al. 2013; Yang et al. 2019a, b, c; Wang et al. 2020a, b; Thi et al. 2020; Misra et al. 2022; Bowden et al. 2023; Grygiel et al. 2024; Cieślik et al. 2025b). According to estimates MDR pathogens infect over 2 million people annually in the United States, causing approximately 29,000 deaths and generating healthcare costs of around $5 billion (Centers for Disease Control And Prevention 2019). In Europe, these pathogens are estimated to cause nearly 1 million infections and 33,000 deaths each year (Cassini et al. 2019). The continuing spread of bacteria resistant to multiple antibiotic classes, driven by the overuse of these chemotherapeutics, underscores the urgent need for novel therapeutic strategies to address this growing public health challenge.

## ESKAPE group of pathogens

Bacterial species classified within the ESKAPE (*Enterococcus faecium*, *Staphylococcus aureus*, *Klebsiella pneumoniae*, *Acinetobacter baumannii*, *Pseudomonas aeruginosa*, and *Enterobacter* spp.) group were selected due to their specific mechanisms that allow them to 'escape' the action of antibiotics (Tarasenko et al. 2025). Each of them is capable of developing advanced MDR mechanisms (Cieślik et al. 2022; Cirit et al. 2019; De Oliveira et al. 2020; de Souza et al. 2023; Li et al. 2025a, b, c, d, e, f, g; Lv et al. 2020; Mlynarczyk-Bonikowska et al. 2022; Nam et al. 2013; Pang et al. 2019; Park and Park 2021; Rahimzadeh Torabi et al. 2021; Sabet et al. 2020; Shridhar et al. 2022).

*E. faecium* – a Gram-positive bacterium, frequently resides in the mammalian intestinal tract (Wei et al. 2024). It is a common nosocomial pathogen responsible for septicemia, endocarditis, urinary tract infections (UTIs), wound infections, and others (Shridhar et al. 2022; Rehman et al. 2018), and is mainly associated with resistance to vancomycin, one of the most important drugs used to treat infections caused by Gram-positive bacteria (Lee et al. 2019a, b).

*S. aureus* – a Gram-positive, facultatively anaerobic bacterium, which is one of the leading causes of healthcare-associated infections (HAIs) (Alonso et al. 2022). It expresses a diverse group of virulence factors that facilitate host colonization, mediate tissue damage, and enable evasion of the host immune response (Bien et al. 2011), causing scalded skin syndrome, superficial skin, and soft tissue infections, osteomyelitis, pneumonia, meningitis, endocarditis, bacteremia, and sepsis. This bacterium is associated with advanced mechanisms of methicillin resistance, specifically methicillin-resistant *Staphylococcus aureus* (MRSA) (McGuinness et al. 2017; Hamwi and Salem-Sokhn 2025).

Pneumonia, liver abscesses, bacteremia, and UTI are infections frequently caused by an opportunistic Gram-negative bacterium – *K. pneumoniae* (Wang et al. 2020a, b). It primarily affects individuals with impaired immune systems (Tan et al. 2019), contributing to high mortality rates, reaching up to 50% of infected people (Russo et al. 2023).

*A. baumannii* is also a Gram-negative, aerobic bacterium that currently constitutes a significant threat, especially to immunocompromised patients (Bagińska et al. 2021; Ibrahim et al. 2021). Moreover, the most recent WHO Bacterial Priority Pathogens List designates carbapenem-resistant *Acinetobacter baumannii* (CRAB) as one of the most critical threats to human health among Gram-negative bacteria (WHO Bacterial Priority Pathogens List 2024).

Another Gram-negative bacterium belonging to ESKAPE group is *P. aeruginosa*, a pathogen with one of the highest levels of genome plasticity among already dscribed bacteria, which allows it to evade antibiotic therapy (Gellatly and Hancock 2013; Das et al. 2020). This microbe may be a cause of a highly lethal ventilator-associated pneumonia (VAP), chronic lung infections associated with cystic fibrosis or non-cystic fibrosis bronchiectasis (De Oliveira et al. 2020; Maurice et al. 2018).

In the past three decades, bacteria belonging to the *Enterobacter* spp. (mainly *E. cloacae*, *E. hormaechei*, as well as *E. aerogenes* – which has been reclassified as *Klebsiella aerogenes*) have emerged as notable challenges in neonatal wards and intensive care units (ICUs) patients, particularly requiring mechanical ventilation (De Oliveira et al. 2020). Infections caused by *E. cloacae* include bacteremia, osteomyelitis, septic arthritis, soft tissue and intra-abdominal infections, respiratory infections, and UTIs (Davin-Regli and Pagès 2015).

Despite not being included in the ESKAPE group, *E. coli* is one of the pathogens that WHO has classified as “critical” in its antimicrobial resistance report (Fuga et al. 2022). This bacterium is highly genetically plastic and capable of colonizing many tissues, and it can also cause various infections such as diarrhea, bloodstream infections, and UTIs (Xu et al. 2025).

## Bacteriophages

Bacteriophages are prokaryotic viruses that specifically infect their bacterial hosts (Olszak et al. 2017; Hetta et al. 2023). Their specificity to bacterial strains (within the same host species) initially led to their consideration as potential antimicrobial agents, particularly as antibiotic resistance continues to rise within microbial communities (Jończyk-Matysiak et al. 2019). Bacteriophages exhibit two most prevalent infection cycles: lytic and lysogenic. Although, there are known temperate phages (phages undergoing lysogenic cycle), which can exhibit lytic activity against ESKAPE pathogens via cycle switch (James et al. 2015; Das et al. 2020; Zhang et al. 2022a, b, c), these phages are not recommended in terms of phage therapy (Popescu et al. 2021) because of their ability to integrate their genetic material into the bacterial genome. Temperate phages can acquire bacterial genes (encoding toxins or determining resistance mechanisms) and facilitate their spread within the bacterial population via horizontal gene transfer (HGT). In contrast, lytic bacteriophages are considered one of the potential tools against MDR bacteria (Górski et al. 2018; Cieślik et al. 2021). The phage lytic cycle consists of four main stages: (i) adsorption of viral receptor binding proteins (RBPs) to receptors expressed on bacterial cell surface; (ii) injection of a viral genetic material to the host cell; (iii) replication of phage genome, gene expression, and assembly of complete virions; and (iv) lysis of the host cell mediated by phage-encoded lytic enzymes (Olszak et al. 2017). The number of progeny virions released from one infected bacterial cell is termed the burst size and is usually expressed in plaque forming unit per cell (PFU/cell).

Successful treatments of infections caused by ESKAPE pathogens using phage therapy have been reported (Letkiewicz et al. 2009; Cano et al. 2021; Oyejobi et al. 2022; Teney et al. 2024; Van Nieuwenhuyse et al. 2024); however, there are still challenges in evaluating the capability of newly isolated bacteriophages in vitro. Comprehensive biological characterization of bacteriophages is essential in the phage preparation process (Jończyk-Matysiak et al. 2019). Phages exhibiting a high adsorption rate, a large burst size, and a short latent period are generally favored for therapeutic purposes. In addition, broad-host-range phages – capable of infecting multiple strains – are advantageous because they reduce the likelihood of phage-resistant mutant emergence, since resistance mechanisms (such as receptor modification, CRISPR-Cas activity, or restriction-modification systems) are often strain-specific. A phage that can infect multiple bacterial strains or species maintains therapeutic efficacy even if a subpopulation develops resistance, thereby limiting the chances of treatment failure (Egido et al. 2022). Moreover, phages may encode lytic enzymes, such as depolymerases (revealed by a ‘halo’ effect around the lysis area), which degrade bacterial capsules and extracellular layers consisting of polysaccharides, which are important components of biofilm structure (Egido et al. 2022; Grygiel et al. 2024). Thus, phages encoding depolymerases in their genomes may also be advantageous in phage therapy, as they can accelerate treatment and increase the likelihood of a positive outcome. Hence, in this scoping review, available data about phages were summarized to make it possible to estimate the parameters of the phage infection cycle of a newly isolated bacteriophage. Although this work focuses primarily on analyzing phage biological parameters associated with infection kinetics, the significance of features such as host range and ‘halo’ presence of phages are also mentioned. Therefore, it will be possible to determine whether a newly isolated bacteriophage exhibits strong or weak lytic activity. A better understanding of the kinetic properties of these bacteriophages provides not only a comparative framework for assessing phage fitness but also establishes reference values that may serve as benchmarks for future studies. Such data are essential for the rational design of therapeutic phage cocktails, enabling the selection of strains with favorable replication kinetics and supporting the prioritization of candidates for clinical application. Furthermore, the generated dataset enables cross-species comparisons within the ESKAPE group, providing insights into why phage therapy may exhibit variable efficacy depending on the pathogen. Importantly, these kinetic parameters constitute the foundation for mathematical modeling of phage-bacteria dynamics in vivo and set biological limits that can guide evolutionary approaches or genetic engineering strategies aimed at improving phage performance. Altogether, this work provides the first consolidated reference for phage growth kinetics regarding ESKAPE pathogens, bridging fundamental biology with translational applications in the fight against antimicrobial resistance.

## Materials and methods

A literature search was conducted in December 2025 in the PubMed database (articles published from 1998 to 2025) using the following keywords: “Enterococcus phage”, “Staphylococcus phage”, “Klebsiella phage”, “Acinetobacter phage”, “Pseudomonas phage”, “Enterobacter phage”, “E. coli phage” (corresponding respectively to different ESKAPE pathogens). For articles about phages infecting *Staphylococcus*; *Klebsiella*; *Acinetobacter*; *Pseudomonas* and *E. coli* – only works containing data about phages infecting specifically *S. aureus*; *K. pneumoniae*; *A. baumannii*; *P. aeruginosa* and *E. coli* were taken under consideration. In terms of articles about phages infecting *Enterococcus* spp. and *Enterobacter* spp., it was not species-segregated to collect more information (in order to get results more statistically significant). Only peer-reviewed full-length articles containing data regarding the time needed for phage particles to adsorb the bacterial cell, and/or calculated latent period, and/or calculated burst size were included in the review. Hence, if given, the information was collected about:

1. Phage designation – had to be stated explicitly in the text to be taken under consideration, if not – “no data”.
2. Host strain – had to be stated explicitly in the text to be taken under consideration, if not – “no data”.
3. Presence of ‘halo’ effect – had to be stated explicitly in the text or clearly visible on a photograph of an agar plate. Other information was classified as “no data”.
4. Type of phage morphology – had to be stated explicitly in the text in the form of, e.g., “myovirus type of morphology” or as by previous nomenclature, e.g., “*Myoviridae*” – both examples here defined as “myovirus” type of morphology (“*Podoviridae*” was defined as “podovirus”; “*Siphoviridae*” was defined as “siphovirus”, respectively). If other types of morphology were given, they were put unchanged in the table. If there was no information given – indicate “no data”.
5. Results of the studied multiplicity of infection (MOI; used in the experiment of one step growth curve, if not given, MOI used in the experiment of adsorption rate) – had to be stated explicitly in the text in the form of “MOI = x was used to…”, if not, information was classified as “no data”. There was no calculation made based on the given bacterial and phage titers used.
6. Adsorption time [s] – time needed for more or equal of 70% phage particles to adsorb to their bacterial host strain. This cut-off was selected based on its frequent use in the reviewed literature and its adequacy in indicating a successful phage-host interaction. Phages reaching or exceeding this level of adsorption were considered to exhibit efficient binding to bacterial cells, which is a prerequisite for effective infection and subsequent lytic activity. This parameter is derived from the text; graphs were not analyzed when collecting the information provided by researchers. If unavailable in the text – marked as “no data”.
7. Latent period [s] – had to be stated explicitly in the text; no graphs were analyzed while searching for the information in order to gather specific results given by researchers. If not available – marked as “no data”.
8. Lysis time [s] – had to be stated explicitly in the text; no graphs were analyzed while searching for the information to gather specific results given by researchers. If not reported – indicate “no data”.
9. Burst size [PFU/cell] – had to be stated explicitly in the text; no graphs were analyzed while searching for the information to gather specific results given by researchers. If not available – marked as “no data”.
10. Host range of the bacteriophage infecting bacteria belonging to the same species. Data was collected as a fraction of vulnerable strains/tested strains and as a percentage (which was sometimes calculated in order to obtain more accurate results). If not given – marked as “no data”.
11. Species specificity – analysis of polyvalence vs. species-specificity (collected as an exact number of tested other bacterial strains (if given) and defined as the ability for the phage to infect other bacterial strains or not). If not given – marked as “no data”.
12. Phage gene accession number (if not given in the paper, also checked using the phage name while searching the GenBank database).
13. Reference – an article from which data was collected.

All data were collected in the Supplementary tables (S1-S28).

While analyzing phages infecting *Enterococcus* spp. and *Enterobacter* spp., no host subspecies segregation (in terms of the host bacterium, which was designated as a host strain) was performed during the article acquisition process; however, diversification was applied in the “host species” column. Therefore, the lytic spectrum was also analyzed within the same bacterial species as a host strain.

All data after the exclusion criteria and initial analysis in terms of adsorption time needed for 70% or more phage particles to adsorb to bacterial host cell, latent period, burst size, and lysis time were subjected to basic descriptive statistical analysis, including range (minimum and maximum values), mean, standard deviation (SD), and standard error of the mean (SE).

## Results

### Exclusion criteria, data analysis, and descriptive statistics

#### Adsorption time needed for more or equal of 70% phage particles to adsorb to their bacterial host strain

As mentioned, the first step of the phage infection cycle is adsorption to bacterial cells, which involves the binding of viral proteins to specific receptors located on the bacterial surface (Olszak et al. 2017). This stage is essential for host recognition and the initiation of infection. Since it represents the initial phase of the phage replication cycle, it is crucial for successful phage amplification (Leprince and Mahillon 2023).

Adsorption time values exceeding 1800 s were considered extreme for the phage in question and were excluded from the analyses, as their infrequent occurrence and extreme magnitude could disproportionately influence the interpretation and visual presentation of the data.

After applying the inclusion criteria phages infecting *Enterococcus* spp. (*n* = 9) demonstrated a mean adsorption time of 633.33 s (Table 1). Notably, the shortest value (60 s; Buzikov et al. 2023) was comparable to values reported for phages infecting *S. aureus* (ϕMR11 bacteriophage (Matsuzaki et al. 2003)), suggesting that phages infecting these two pathogens may share similar adsorption dynamics (Matsuzaki et al. 2003). It was proven based on the average adsorption time of phages infecting *S. aureus* (665.74 s (Table 1)). Phages infecting Gram-negative pathogens generally displayed faster adsorption time, which was proven by the mean adsorption time of phages infecting *A. baumannii* (579.11 s); *P. aeruginosa* (551.14 s); *K. pneumoniae* (452.14 s) and *E. coli*, which exhibited the shortest mean adsorption time in terms of phages infecting analyzed pathogens – 420.16 s (Table 1). Surprisingly, the highest result of the mean adsorption time was obtained within the analysis of *Enterobacter*-specific phages – 720 s (Table 1).

**Table 1:** Summary of the most important biological features associated with the infection cycle of bacteriophages specific to bacteria belonging to the ESKAPE group and *E. coli*. Results are presented as means ± standard deviation (SD), and standard error of the mean (SE) is also included

Biological feature Specificity	Mean time needed for ≥ 70% phage particles to adsorb to bacterial host [s] ± SD; SE; number of analyzed phages	Mean latent period [s] ± SD; SE; number of analyzed phages	Mean lysis time [s] ± SD; SE; number of analyzed phages	Mean burst size [PFU/cell] ± SD; SE; number of analyzed phages
*Enterococcus* spp*.*	633.33 ± 426.5; 142.17; *n* = 9	1376.4 ± 900.65; 127.39; *n* = 50	2182.35 ± 1227.41; 297.92; *n* = 17	128.04 ± 150.46; 21.49; *n* = 49
*S. aureus*	665.74 ± 416.05; 60.65; *n* = 47	1720.25 ± 1003.59; 112.26; *n* = 80	2605.56 ± 1479.85; 349.02; *n* = 18	97.70 ± 117.70; 13.24; *n* = 79
*K. pneumoniae*	452.14 ± 251.17; 30.01; *n* = 70	1389.27 ± 861.02; 62.30; *n* = 191	2448.21 ± 1371.24; 183.32; *n* = 56	139.80 ± 132.10; 10.26; *n* = 166
*A. baumannii*	579.11 ± 362.6; 40.79; *n* = 79	1259.18 ± 745.06; 75.64; *n* = 97	2510.53 ± 1379.17; 316.32; *n* = 19	180.21 ± 133.69; 14.33; *n* = 87
*P. aeruginosa*	551.14 ± 401.22; 61.16; *n* = 43	1657.11 ± 947.82; 91.22; *n* = 108	2221.3 ± 1287.38; 189.88; *n* = 46	153.04 ± 169.28; 16.76; *n* = 102
*Enterobacter* spp.	720 ± 363.32; 137.10; *n* = 7	1178.28 ± 573.65; 106.43; *n* = 29	2236.36 ± 1583.84; 477.06; *n* = 11	118.15 ± 83.4; 16.04; *n* = 27
*E. coli*	420.16 ± 299.83; 49.31; *n* = 37	1282.82 ± 784.45; 72.5; *n* = 117	2006.04 ± 955.77; 131.29; *n* = 53	197 ± 221.96; 21.26; *n* = 109

#### Latent period

Latent period is defined as the time between phage adsorption and the initial release of progeny virions. During this stage, the viral genome is replicated and expressed, leading to the assembly of new phage particles inside the host bacterial cell (Olszak et al. 2017).

Latent period equal or exceeding 5400 s was considered an extreme value and was excluded from the statistical analyses, as its infrequent occurrence and large values could disproportionately influence the interpretation and visual presentation of the data.

The longest latent period (6000 s) was observed while analysing collected data regarding to phages infecting *K. pneumoniae* (Duarte et al. 2024). Interestingly, phages infecting Gram-positive pathogens within this analysis did not exceed 4800 s but the highest mean latent period was obtained analysing *S. aureus* 1720.25 s, followed by phages infecting *P. aeruginosa*, exhibiting also one of the longest mean latent period in this review (1657.11 s (Table 1)). Mean latent periods characterizing phages infecting *Enterobacter* spp.; *A. baumannii*; *E. coli*; *Enterococcus* spp.; *K. pneumoniae* did not exceed 1400 s (1178.28 s; 1259.18 s; 1282.82 s; 1376.4 s; 1389.27 s, respectively (Table 1)), which might suggest that latent period is not differentiated alongside to the Gram classification.

#### Lysis time

Lysis time refers to the period from phage infection until the bacterial cell is lysed by phage-encoded enzymes, resulting in the release of progeny virions (Olszak et al. 2017).

Lysis time exceeding 5400 s was considered an exceptional event for the phage in question and was excluded from the statistical analyses, as its infrequent occurrence and extreme magnitude could disproportionately influence the interpretation and visual presentation of the data.

Similarly to the mean and a maximum result within latent period, the longest lysis time was observed within the analysis of phages infecting *P. aeruginosa* (10500 s (Zhang et al. 2025a, b, c, d)). Nevertheless, the highest value in terms of the mean lysis time was exhibited by phages infecting *S. aureus* (2605.56 s (Table 1)). Interestingly, the second-highest record was observed for phages infecting *Enterococcus* spp. (2182.35 s (Table 1)), which could be suggested as a result of thick peptidoglycan layer of Gram-positive pathogen expressed by host species. Both *A. baumannii*-specific phages and *K. pneumoniae*-specific phages could be defined as phages exhibiting a second to the highest results in terms of the mean lysis time (2510.53 s and 2448.21 s, respectively (Table 1)). Interestingly, phages infecting *P. aeruginosa* reached the mean lysis time of 2221.3 s (Table 1), similarly to phages infecting *Enterobacter* spp. (2236.36 s (Table 1)), which may stand for some similarities between phages infecting these pathogens. The shortest lysis time was exhibited by phages infecting *E. coli* (2006.04 s (Table 1)), which is relatively low, comparing to mentioned ESKAPE pathogens acting as a host for analysed phages.

#### Burst size

The number of progeny phages released from a single infected bacterial cell following lysis is referred to as the burst size, i.e., the number of viral particles released per cell (Jończyk-Matysiak et al. 2019).

Burst size values lower than 2 PFU/cell and exceeding 1000 PFU/cell were considered extreme for the phage in question and were excluded from the statistical analyses, as their infrequent occurrence and extreme magnitude could disproportionately influence the interpretation and visual presentation of the data. Furthermore, studies reporting only approximate ranges of burst sizes were excluded from the analysis, as our assessment required precise numerical values rather than estimated intervals.

Descriptive statistics in terms of phages infecting analysed pathogens revealed one of the lowest burst sizes for *S. aureus* (97.70 PFU/cell) and *Enterococcus* spp. (128.04 PFU/cell), which suggest that phages infecting Gram-positive pathogens might exhibit lower burst sizes than phages specific to Gram-negative bacteria (Table 1). Nevertheless, second lowest burst size was exhibited by phages infecting *Enterobacter* spp. (118.15 PFU/cell). Results oscillating around 145 PFU/cell were observed within phages infecting *K. pneumoniae* and *P. aeruginosa* (139.80 PFU/cell and 153.04 PFU/cell respectively (Table 1)), whereas the highest burst sizes were observed while analyzing phages infecting *A. baumannii* (180.21 PFU/cell) and *E. coli* (197 PFU/cell). Interestingly, phages infecting *E. coli* not only exhibited the shortest lysis and adsorption time, but also the highest burst size.

In order to compare phage infection, cycle kinetic parameters between phages infecting different ESKAPE pathogens, the Kruskal–Wallis test was performed. The analyses were conducted only when at least 20 phages remained after exclusions, to ensure adequate relevance in terms of specific parameters, thereby maintaining the appropriate relevance of the obtained results. Hence, Enterococcus phages and Enterobacter phages were not analyzed in terms of adsorption; in terms of lysis time, only phages infecting *K. pneumoniae*; *P. aeruginosa* and *E. coli* were included in calculations; whereas the statistical analysis of burst size and latent period consisted of all analyzed phages. Furthermore, graphs were generated in order to visualize all the obtained results. Statistical analyses and graph generation were performed using Statistics Kingdom [web application].

#### Further statistical analysis of selected data (Kruskal–Wallis H test)

To verify the normal distribution within the analyzed groups (divided according to bacterial host species), the Shapiro–Wilk test was performed.

For the adsorption time, the Shapiro–Wilk test indicated a significant deviation from normality in all analysed groups:

- *S. aureus* – W(47) = 0.89, *p* < 0.001 (where “(47)” refers to the number of observations included in the analysis);
- *K. pneumoniae* – W(70) = 0.75, *p* < 0.001;
- *A. baumannii* – W(79) = 0.84, *p* < 0.001;
- *P. aeruginosa* – W(43) = 0.7, *p* < 0.001;
- *E. coli* – W(37) = 0.87, *p* < 0.001.

For the latent period parameter, a significant deviation from normality was observed in almost all groups, except for phages infecting *Enterobacter* spp. (W(29) = 0.94, *p* = 0.118):

- *Enterococcus* spp. – W(50) = 0.77, *p* < 0.001;
- *S. aureus* – W(80) = 0.88, *p* < 0.001;
- *K. pneumoniae* – W(191) = 0.89, *p* < 0.001;
- *A. baumannii* – W(97) = 0.91, *p* < 0.001;
- *P. aeruginosa* – W(108) = 0.91, *p* < 0.001;
- *E. coli –* W(117) = 0.9, *p* < 0.001.

With regard to lysis time, the Shapiro–Wilk test indicated a significant deviation from normality in three cases:

- *K. pneumoniae* – W(56) = 0.95, *p* = 0.017;
- *P. aeruginosa* – W(46) = 0.94, *p* = 0.013;
- *E. coli –* W(53) = 0.89, *p* < 0.001.

In terms of burst size, the Shapiro–Wilk test showed a significant deviation from normality considering all cases:

- *Enterococcus* spp. – W(49) = 0.64, *p* < 0.001;
- *S. aureus* – W(79) = 0.7, *p* < 0.001;
- *K. pneumoniae* – W(166) = 0.79, *p* < 0.001;
- *A. baumannii* – W(87) = 0.86, *p* < 0.001;
- *P. aeruginosa* – W(102) = 0.7, *p* < 0.001;
- *Enterobacter* spp. – W(27) = 0.86, *p* = 0.002;
- *E. coli* – W(109) = 0.74, *p* < 0.001.

Since the data did not meet the assumptions for parametric tests, the Kruskal–Wallis H test was applied to assess differences between the analyzed groups.

## Adsorption time

**Fig. 1:**
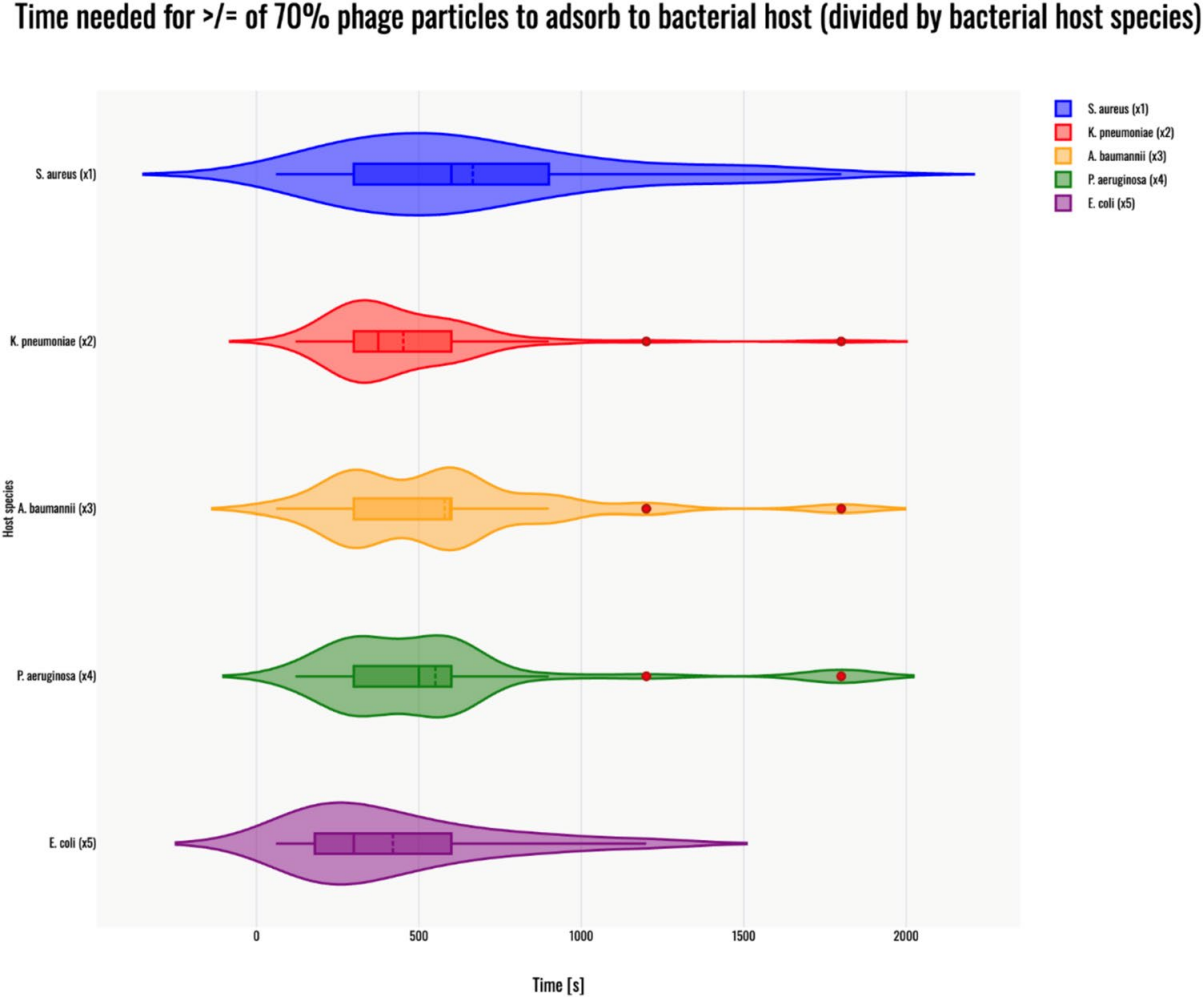
Violin plot of time needed for more or equal of 70% phage particles to adsorb to their bacterial host strain for phages infecting: *S. aureus* (blue); *K. pneumoniae* (red); *A. baumannii* (orange); *P. aeruginosa* (green); *E. coli* (purple). Red dots indicate outlier data points, which were included in the statistical analyses despite being identified as outliers. All host species are marked x(n), corresponding to the Kruskal–Wallis H test results above. The violin plot was generated with Statistics Kingdom – Violin plot generator (accessed – 09.12.2025) [web application]

The Kruskal–Wallis H test indicated a significant difference in the dependent variable between the different groups, χ^2^(4) = 16.6, *p* = 0.002, with a mean rank score of 164.67 for phages infecting *S. aureus* (x1), 123.46 for phages infecting *K. pneumoniae* (x2), 151.82 for phages infecting *A. baumannii* (x3), 138.33 for phages infecting *P. aeruginosa* (x4), 105.49 for phages infecting *E. coli* (x5). The post-hoc Dunn's test using a Bonferroni-corrected alpha level of 0.005 indicated that the mean ranks of the following pair differed significantly: x1-x5 and x3- x5. A violin plot was generated to visualize the results – Fig. 1.

## Latent period

**Fig. 2:**
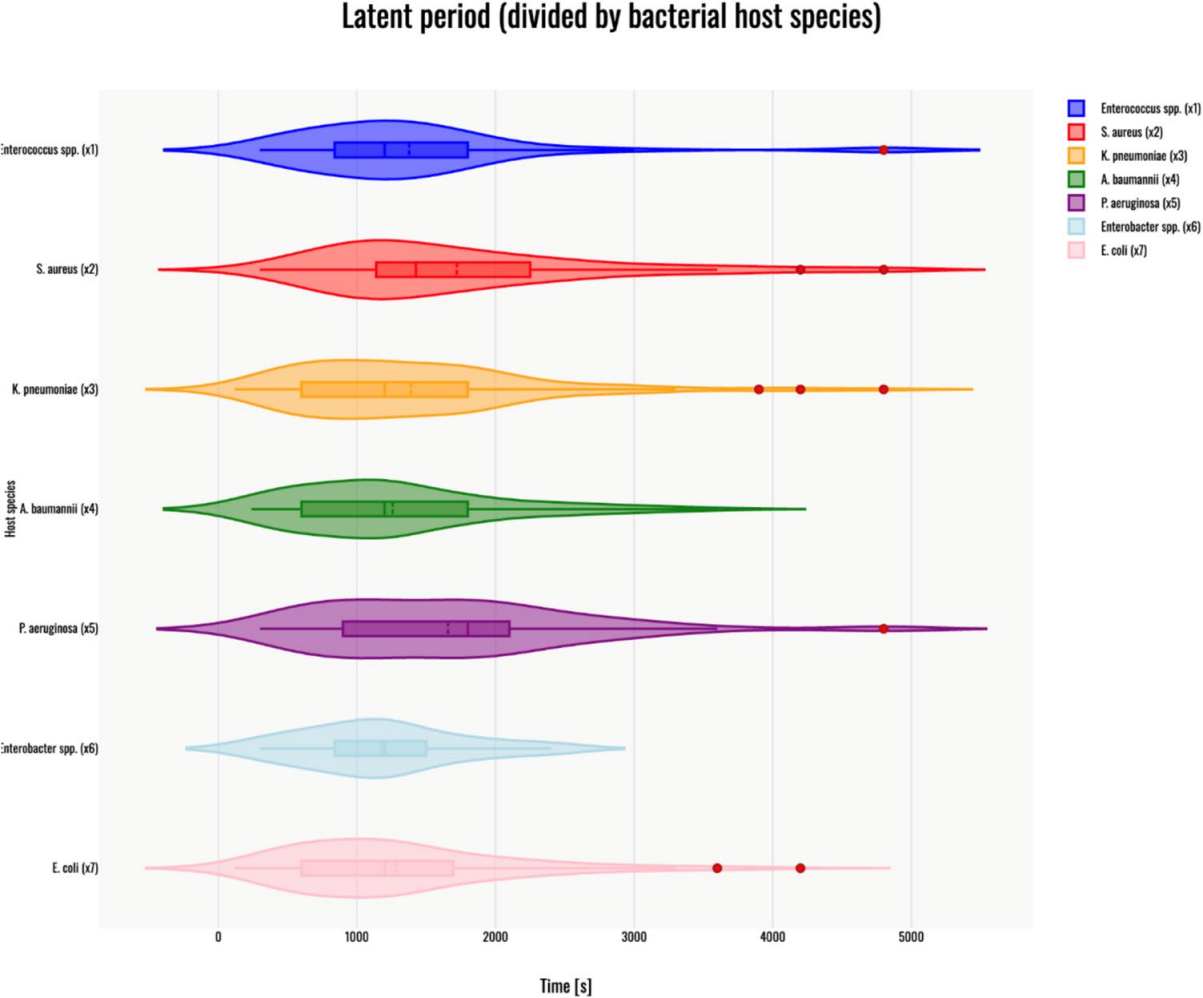
Violin plot of latent periods for phages infecting*: Enterococcus* spp. (dark blue); *S. aureus* (red); *K. pneumoniae* (orange); *A. baumannii* (green); *P. aeruginosa* (purple); *Enterobacter* spp. (light blue) and *E. coli* (pink). Red dots on the graph indicate outlier data points, which were included in the statistical analyses despite being identified as outliers. All host species are marked by x(n) corresponding to the Kruskal–Wallis H test results above. The violin plot was generated with Statistics Kingdom – Violin plot generator (accessed – 09.12.2025) [web application]

The Kruskal–Wallis H test indicated a significant difference in the dependent variable between the different groups, χ^2^(6) = 23.83, *p* < 0.001, with a mean rank score of 322.71 for phages infecting *Enterococcus* spp. (x1), 396.19 for phages infecting *S. aureus* (x2), 328.01 for phages infecting *K. pneumoniae* (x3), 301.24 for phages infecting *A. baumannii* (x4), 389.1 for phages infecting *P. aeruginosa* (x5), 296.36 for phages infecting *Enterobacter* spp. (x6), 306.07 for phages infecting *E. coli* (x7). The post-hoc Dunn's test using a Bonferroni-corrected alpha level of 0.0024 indicated that the mean ranks of the following pairs differ significantly: x2- x4, x2-x7, x4-x 5, and x5-x7. A violin plot was generated to visualize the results – Fig. 2.

## Lysis time

**Fig. 3:**
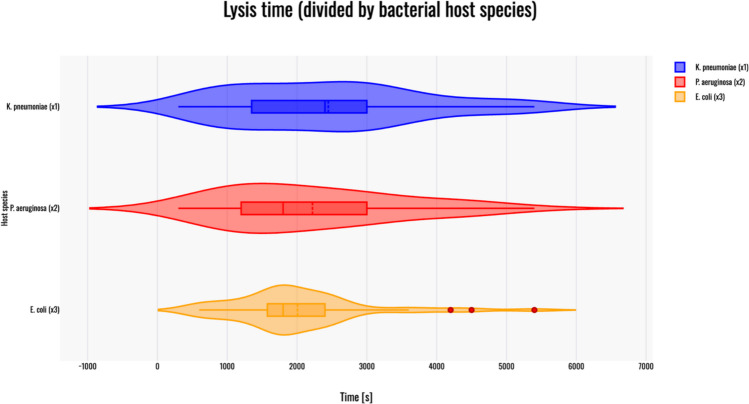
Violin plot of lysis times for phages infecting: *K. pneumoniae* (blue); *P. aeruginosa* (red) and *E. coli* (orange). Red dots on the graph indicate outlier data points that were included in the statistical analyses despite being identified as such. All host species are marked by x(n) corresponding to the Kruskal–Wallis H test results above. The violin plot was generated with Statistics Kingdom – Violin plot generator (accessed – 09.12.2025) [web application]

The Kruskal–Wallis H test indicated no significant difference in the dependent variable between the groups, χ^2(^2) = 2.7, *p* = 0.26, with a mean rank score of 85.21 for phages infecting *K. pneumoniae* (x1), 77.07 for phages infecting *P. aeruginosa* (x2), and 71.2 for phages infecting *E. coli* (x3). A violin plot was generated to visualize the results – Fig. 3.

## Burst size

**Fig. 4:**
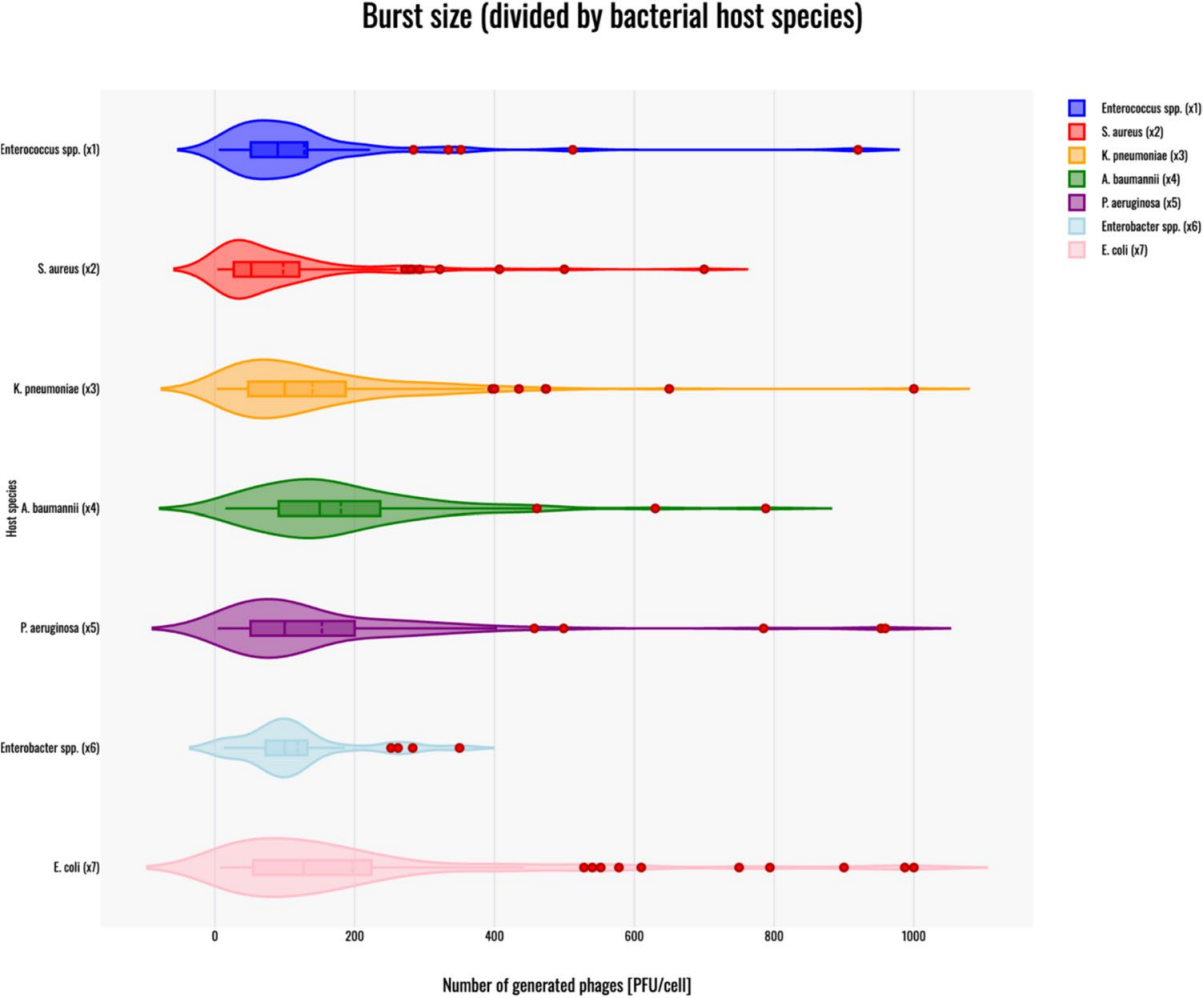
Violin plot of the burst size for phages infecting*: Enterococcus* spp. (dark blue); *S. aureus* (red); *K. pneumoniae* (orange); *A. baumannii* (green); *P. aeruginosa* (purple); *Enterobacter* spp. (light blue) and *E. coli* (pink). Red dots on the graph indicate outlier data points that were included in the statistical analyses despite being identified as such. All host species are marked by x(n) corresponding to the Kruskal–Wallis H test results above. The violin plot was generated with Statistics Kingdom - Violin plot generator (09.12.2025) [web application]

The Kruskal–Wallis H test indicated a significant difference in the dependent variable between the different groups, χ2(6) = 36.7, *p* < 0.001, with a mean rank score of 280.58 for phages infecting *Enterococcus* spp. (x1), 223.9 for phages infecting *S. aureus* (x2), 306.33 for phages infecting *K. pneumoniae* (x3), 381.36 for phages infecting *A. baumannii* (x4), 308.53 for phages infecting *P. aeruginosa* (x5), 294.96 for phages infecting *Enterobacter* spp. (x6), 339.35 for phages infecting *E. coli* (x7). The post-hoc Dunn's test using a Bonferroni-corrected alpha level of 0.0024 indicated that the mean ranks of the following pairs are significantly different: x1-x4, x2-x3, x2-x4, x2- x5, x2-x7, and x3-x4. A violin plot was generated to visualize results – Fig. 4.

## Distribution of data unqualified for statistical analysis

### Adsorption time

Since phages infecting *Enterococcus* spp. and *Enterobacter* spp. were excluded from further statistical analysis, the Shapiro–Wilk test was performed to assess normality of the data distribution within the tested groups. The Shapiro–Wilk test indicated no significant deviation from normality for either group. The W statistic measures how closely the ordered sample values match a normally distributed set with the same mean and variance. A W value close to 1 suggests that the data are approximately normally distributed. The p-value (*p* > 0.05) supports the acceptance of the null hypothesis (H_0_):

- phages infecting *Enterococcus* spp. did not show a significant departure from normality: W(9) = 0.94, *p* = 0.582;
- phages infecting *Enterobacter* spp. did not show a significant departure from normality: W(7) = 0.94, *p* = 0.687.

**Fig. 5:**
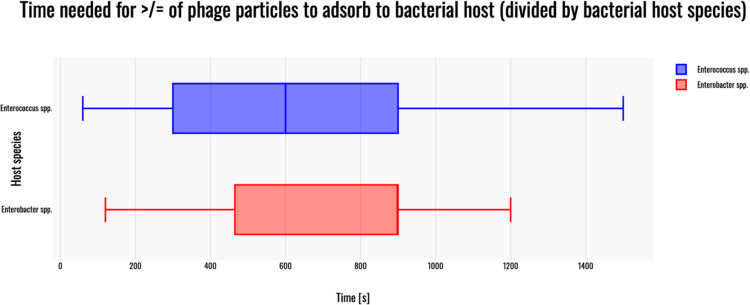
Boxplot of time needed for more or equal of 70% phage particles to adsorb to their bacterial host strain for phages infecting: *Enterococcus* spp. (blue) and *Enterobacter* spp. (red). The box plot was generated with Statistics Kingdom – Box plot generator (accessed – 09.12.2025) [web application]

Furthermore, a box plot was generated in order to visualize the results in terms of adsorption time (Fig. 5).

### Lysis time

Since phages infecting *Enterococcus* spp.; *S. aureus*; *A. baumannii* and *Enterobacter* spp. were not qualified for further statistical analysis, only the Shapiro–Wilk test was performed to assess the normality of data distribution within the tested groups. Among the analyzed groups, no significant deviation from normality was detected:

- phages infecting *Enterococcus* spp.: W(17) = 0.94, *p* = 0.312;
- phages infecting *A. baumannii*: W(19) = 0.93, *p* = 0.193;
- phages infecting *S. aureus*: W(18) = 0.9, *p* = 0.056;
- phages infecting *Enterobacter* spp.: W(11) = 0.9, *p* = 0.179.

**Fig. 6:**
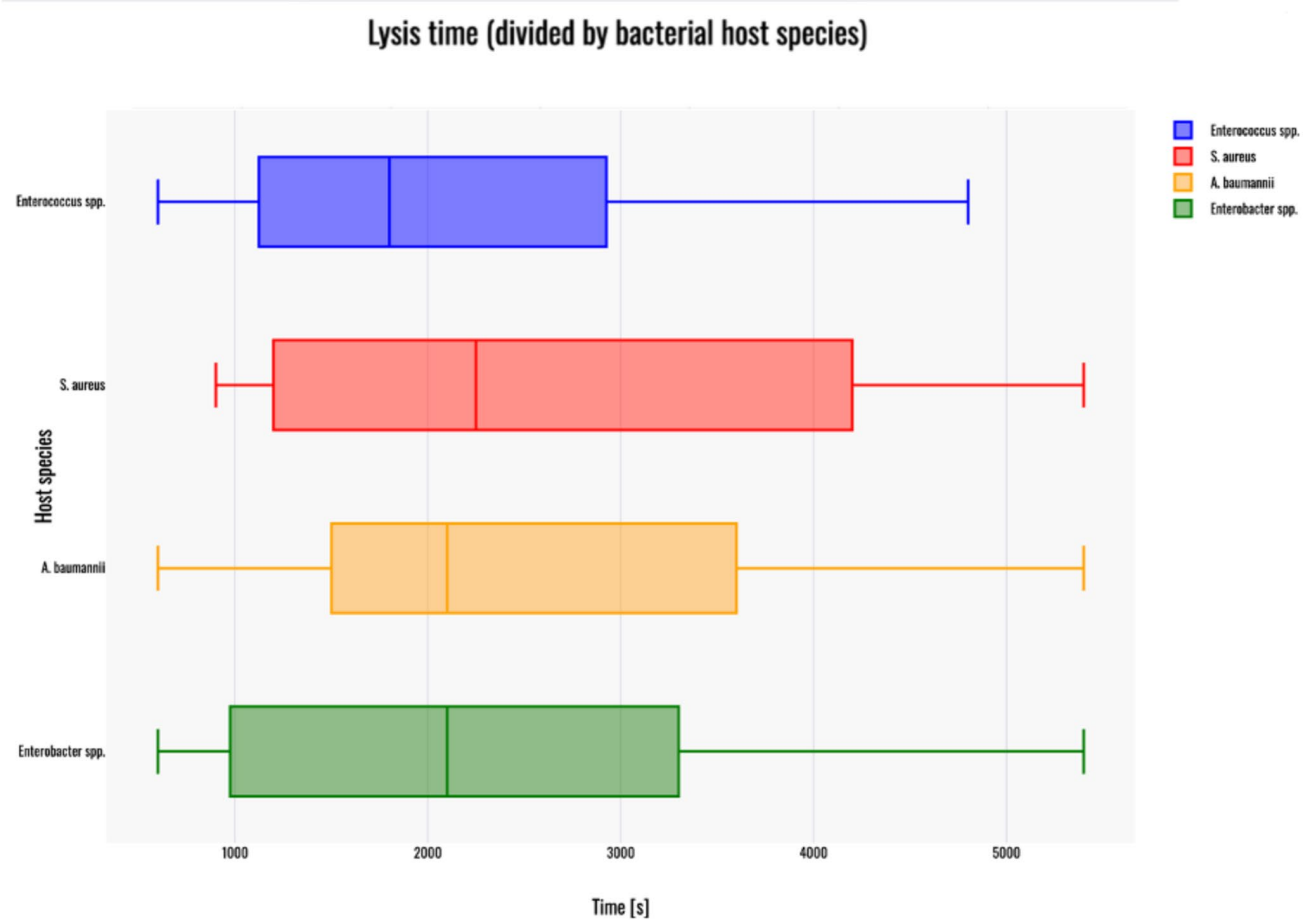
Boxplot of lysis times for phages infecting: *Enterococcus* spp. (blue); *S. aureus* (red); *A. baumannii* (yellow) and *Enterobacter* spp. (green). Red dots on the graph indicate outlier data points that were included in the statistical analyses despite being identified as such. The box plot was generated with Statistics Kingdom – Box plot generator (accessed – 09.12.2025) [web application]

Furthermore, a box plot was generated in order to visualize the results in terms of lysis time (Fig. 6).

### Host range and polyvalence

While analyzing the host range (lytic spectrum) of lytic phages infecting ESKAPE pathogens, it was necessary to exclude experiments that were maintained on fewer than 10 different bacterial strains in terms of the host range experiment, since there was a higher possibility of obtaining false results.

Furthermore, in this analysis, phages were classified as having a broad lytic spectrum if they were capable of infecting at least 60% of the tested bacterial strains (Table 2).

**Table 2:** Characterization of the lytic spectra of the analyzed phages specific for bacterial species from the ESKAPE group and *E. coli*, along with the analysis of their polyvalence

Host species	Number of analyzed phages characterized by broad lytic spectrum	Number of analyzed phages demonstrated specificity to other species (polyvalence)
*Enterococcus* spp.	21 out of 36 (58.33%)	10 out of 38 (26.31%)
*S. aureus*	56 out of 79 (70.89%)	18 out of 50 (36%)
*K. pneumoniae*	39 out of 141 (27.66%)	11 out of 76 (14.47%)
*A. baumannii*	14 out of 86 (16.28%)	5 out of 40 (11.11%)
*P. aeruginosa*	24 out of 79 (30.38%)	6 out of 35 (13.64%)
*Enterobacter* spp.	9 out of 16 (56.25%)	10 out of 20 (50%)
*E. coli*	20 out of 77 (25.97%)	28 out of 56 (50%)

Phages were considered capable of infecting a bacterial cell if any lytic activity was observed, regardless of the efficiency or intensity of lysis. The analysis involved both spot tests and efficiency of plating (EOP) experiments, and all results showing a slight lysis were considered as an indication of the phages' ability to infect the bacterial strain.

The analysis revealed that phages infecting *S. aureus* exhibited the broadest lytic spectrum reaching 70.89%. Nevertheless, these phages were not the most polyvalent phages in the analysed data (36% of *S. aureus*-specific phages were polyvalent (Table 2)). Phages infecting *E. coli* and *Enterobacter* spp. were the most frequently able to perform a cross-species infection (50% of the tested phages from both groups), whereas phages infecting *A. baumannii* were defined as least polyvalent (11.11% (Table 2)). Interestingly, phages infecting this pathogen displayed also the highest host specificity – only 16.28% phages exhibited broad lytic spectrum (Table 2).

### ‘Halo’ presence

The ‘halo’ effect is a clear zone surrounding a phage plaque, typically caused by virion-associated depolymerases that degrade bacterial capsules or extracellular polysaccharides (Pires et al. 2016). Its presence indicates enzymatic activity extending the lysis zone and may suggest that the phage encodes such enzymes. Phages possessing depolymerase activity demonstrate enhanced efficacy against their bacterial hosts, as these enzymes facilitate key processes including host recognition and attachment, cell wall penetration, bacterial lysis, and biofilm degradation.

Characterized lytic phages infecting ESKAPE pathogens differed in their ability to form a halo around plaques:

1. Enterococcus phages – 6 out of 37 (16.22%) formed a halo
2. S. aureus phages – 11 out of 59 (18.64%) formed a halo
3. K. pneumoniae phages – 120 out of 176 (68.18%) formed a halo
4. A. baumannii phages – 60 out of 98 (61.22%) formed a halo
5. P. aeruginosa phages – 31 out of 72 (43.06%) formed a halo
6. Enterobacter phages – 7 out of 19 (36.84%) formed a halo
7. E. coli phages – 26 out of 64 (40.63%) formed a halo

**Fig. 7:**
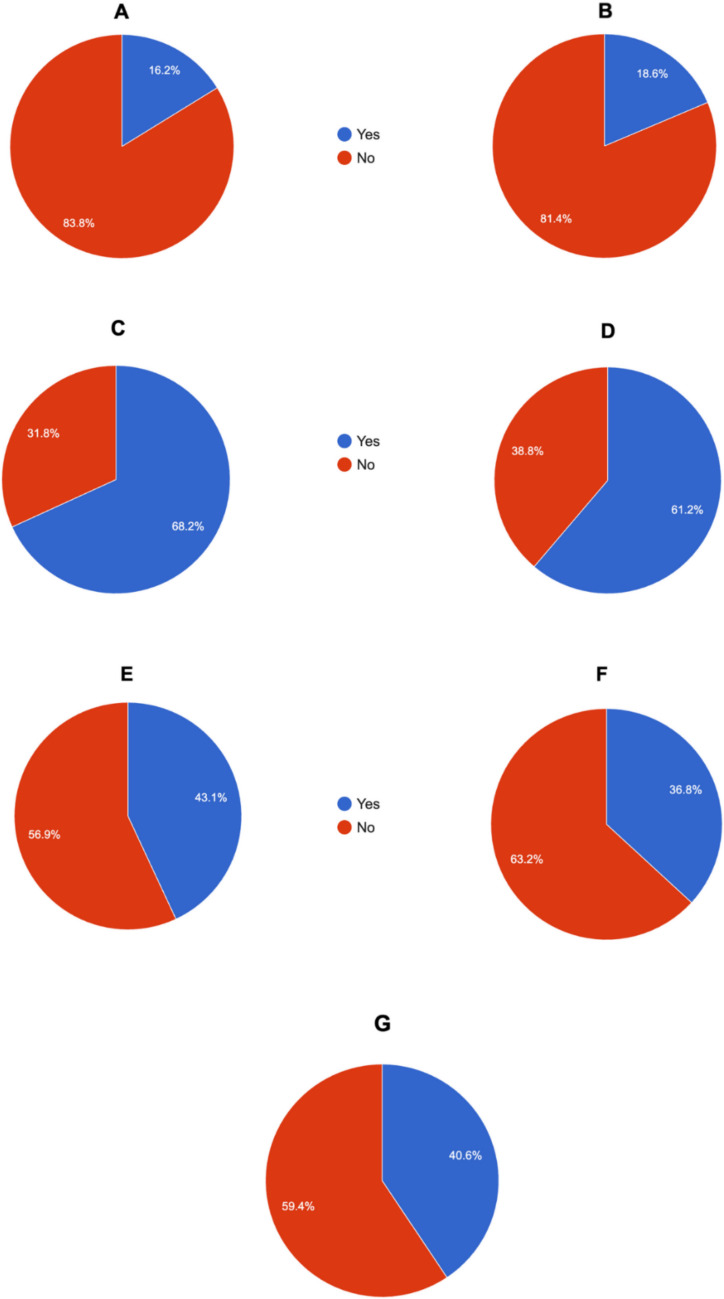
Pie charts showing the percentage distribution of the reported 'halo' effect around phage plaques for bacteriophages specific to pathogens from the ESKAPE group and for *E. coli*. The analyzed phages were: (**A**) *Enterococcus*-specific; (**B**) *S. aureus*-specific; (**C**) *K. pneumoniae*-specific; (**D**) *A. baumannii*-specific; (**E**) *P. aeruginosa*-specific; (**F**) *Enterobacter*-specific; and (**G**) *E. coli*-specific, where blue color – the presence of a halo around plaques, red color – the absence of a halo around plaques. Pie charts were generated with Statistics Kingdom – Pie chart generator (accessed – 09.12.2025) [web application]

As shown in Fig. 7, the halo effect was reported significantly more frequently for phages specific to Gram-negative bacteria, with *Klebsiella*-specific and *Acinetobacter*-specific phages being particularly significant – nearly 70% of these phages exhibited this effect.

### Morphology

**Fig. 8:**
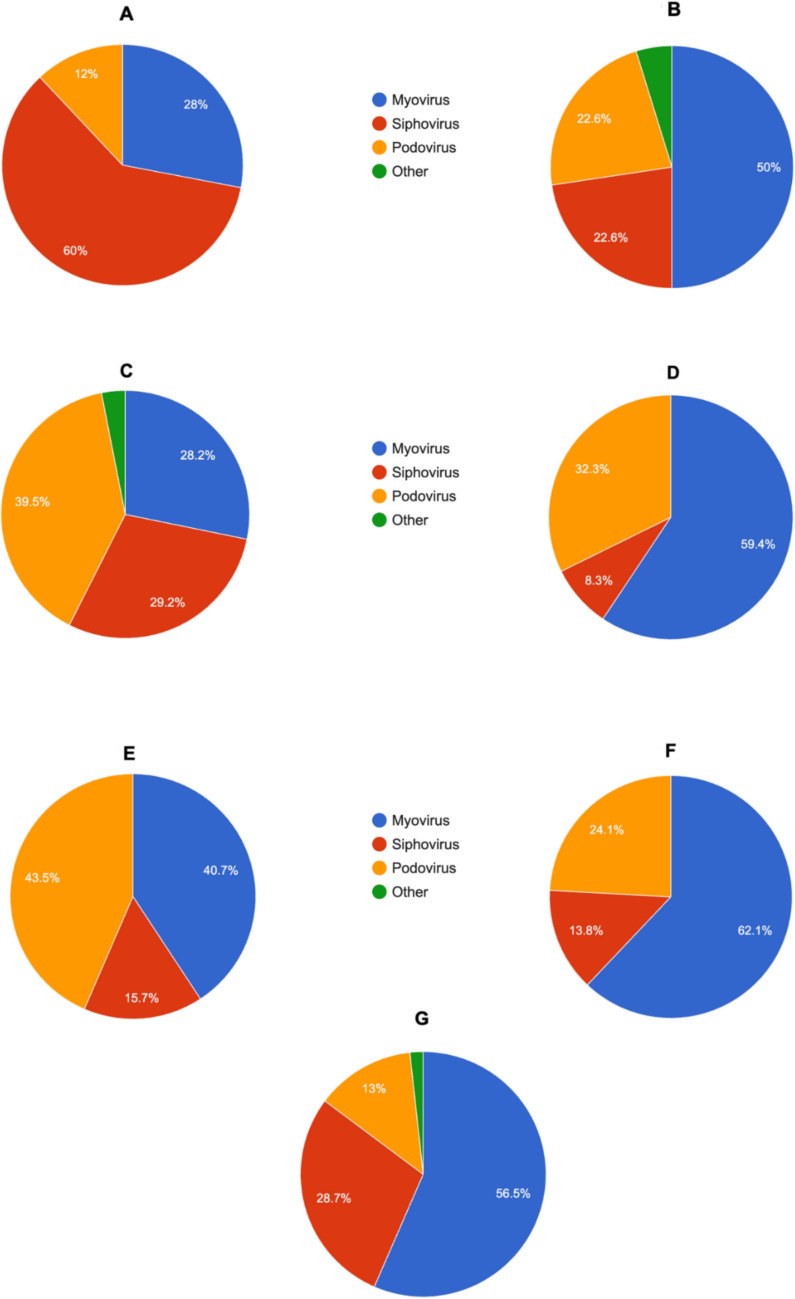
Pie charts showing the proportional representation of phage morphologies among the analyzed phages specific to pathogens from the ESKAPE group and for *E. coli*. The analyzed phages were: (**A**) *Enterococcus*-specific; (**B**) *S. aureus*-specific; (**C**) *K. pneumoniae*-specific; (**D**) *A. baumannii*-specific; (**E**) *P. aeruginosa*-specific; (**F**) *Enterobacter*-specific; and (**G**) *E. coli*-specific, where blue color – myovirus morphology type, red color- siphovirus morphology type, yellow color – podovirus morphology type, green color – other types of morphology. Pie charts were generated with Statistics Kingdom – Pie chart generator (accessed – 09.12.2025) [web application]

Lytic phages infecting ESKAPE pathogens exhibited differences in their morphology (Fig. 8):

1. Enterococcus phages – 14 myoviruses (28%), 30 siphoviruses (60%), 6 podoviruses (12%) of 50 analyzed viruses
2. S. aureus phages – 42 myoviruses (50%), 19 siphoviruses (22.62%), 19 podoviruses (22.62%), 4 others (three jumbo and one cystovirus; 4.76%) of 84 analyzed viruses
3. K. pneumoniae phages – 55 myoviruses (28.21%), 57 siphoviruses (29.23%), 77 podoviruses (39.49%), six others (three taipeivirus, two marfavirus, and one demerecvirus; 3.08%) of 195 analyzed viruses
4. A. baumannii phages – 57 myoviruses (59.38%), 8 siphoviruses (8.33%), 31 podoviruses (32.3%) of 96 analyzed viruses
5. P. aeruginosa phages – 44 myoviruses (40.74%), 17 siphoviruses (15.74%), 47 podoviruses (43.52%) of 108 analyzed viruses
6. Enterobacter phages – 18 myoviruses (62.07%), 4 siphoviruses (13.79%), 7 podoviruses (24.14%) of 29 analyzed viruses
7. E. coli phages – 65 myoviruses (56.52%), 33 siphoviruses (28.69%), 15 podoviruses (13.04%), 2 other (one tectivirus and one jumbo; 1.74%) of 93 analyzed viruses

## Discussion

The reviewed literature highlights a growing interest in bacteriophages as a potential tool to combat ESKAPE pathogens, which are known for their high level of antimicrobial resistance. The majority of studies focused on the biological properties of phages, including, among others, host range specificity, adsorption rate, burst size, and morphological type. Phages targeting *K. pneumoniae*, *A. baumannii*, and *P. aeruginosa* were among the most frequently characterized, reflecting the clinical urgency to find alternatives for MDR strains. In turn, the limited number of comprehensively characterized phages targeting bacterial species such as *Enterobacter* spp. highlights the need for further research in this area (Cieślik et al. 2025b).

This study provides the first consolidated overview of the cycle parameters of strictly lytic bacteriophages infecting ESKAPE pathogens and critical pathogens. There is variability among phages, both within a single bacterial host species and between different species. This variability may result from differences in phage biology, the diversity of bacterial strains, as well as the specific cultivation requirements of bacterial host strains. The expected therapeutic efficacy is difficult to define and varies significantly between individual phages. By establishing reference values for phage replication kinetics, this analysis offers a framework for evaluating newly isolated phages and supports their prioritization for therapeutic development. Beyond guiding phage cocktail design, these benchmarks also contribute to modeling phage-bacteria dynamics and highlight biological limits relevant for evolutionary optimization and engineering of phages.

The first step of the phage infection cycle – adsorption to bacterial cells – involves the binding of viral proteins to specific receptors on the bacterial surface (Olszak et al. 2017). This stage is essential for host recognition and the initiation of infection. It is crucial for successful phage amplification (Leprince and Mahillon 2023). After the analysis of phage adsorption, the shortest time needed for at least 70% of bacteriophages to adsorb to bacterial host, amongst all phages, was defined for phages infecting *K. pneumoniae* (420.16 s). In terms of phages infecting Gram-positive representatives of the ESKAPE group of pathogens, *Enterococcus*-specific phages exhibited a shorter time needed for adsorption. Nevertheless, these phages could not be analyzed using the Kruskal–Wallis H test to ensure adequate relevance. Therefore, a significant difference in the dependent variable could not be indicated. However, the Kruskal–Wallis H test indicated a significant difference between phages infecting *K. pneumoniae* and *S. aureus* in terms of the time needed for at least 70% of phage particles to adsorb to the bacterial host strain. It was proposed that phages encoding depolymerases are able to adsorb to their host strains faster (Leprince and Mahillon 2023). The analysis of ‘halo’ presence in this review indicated that only 16.22% of *Enterococcus*-specific phages and 18.64% of *S. aureus*-specific phages exhibited the ‘halo’ presence around their plaques, which may support the result of relatively long adsorption time for phages infecting these pathogens. Furthermore, phages infecting Gram-positive pathogens can use teichoic acid as their receptor, which is less accessible than, e.g., lipopolysaccharides, which are one of the most common receptors for phages infecting Gram-negative pathogens (Leprince and Mahillon 2023). Interestingly, the highest mean adsorption time was calculated for Enterobacter phages (almost 150 s longer than phages infecting *A. baumannii*, which exhibited the second-longest adsorption time in terms of phages infecting Gram-negative representatives of the ESKAPE group (Table 1)). This result diminishes a possible trend that phages infecting Gram-negative pathogens, amongst the analyzed group, adsorb to bacterial cells faster than phages infecting Gram-positive pathogens. It was suggested that some phages infecting *Enterobacter* spp. might use an auxiliary receptor while adsorbing to the bacterial cell (Fu et al. 2025), and the characteristics of this receptor might alter the adsorption process in terms of this pathogen. However, a similar trend was observed, e.g., in terms of phages infecting *E. coli* or *K. pneumoniae*, which in this review present the shortest adsorption time (Bertozzi Silva et al. 2016; Fu et al. 2025). Nevertheless, further analysis of the molecular structure of those receptors should be performed to understand these differences. Because shorter adsorption times directly accelerate the phage infection cycle, these results may provide a useful benchmark for evaluating future therapeutic candidates. Nevertheless, it is known that longer adsorption time is favored while fighting bacterial biofilm (Gallet et al. 2009); therefore, future candidates proposed for biofilm eradication agents, should not exceed the given benchmark, in order to exhibit more satisfying results regarding biofilm eradication.

Latent period is defined as the time between phage adsorption and the initial release of progeny virions. During this stage, the viral genome is replicated and expressed, resulting in the assembly of new phage particles within the host bacterial cell (Olszak et al. 2017). After the mean evaluation of analyzed phages infecting ESKAPE pathogens and *E. coli*, the shortest latent period was exhibited by Enterobacter phages (1178.28 s), with the lowest SD (573.65). This may be the cause of a prolonged adsorption time, which can be compensated by a shorter latent period in terms of phages infecting this pathogen (Table 1). The Kruskal–Wallis H test did not indicate that this group was statistically different from phages infecting other analyzed pathogens. Nevertheless, statistically different groups were proven to be phages infecting *A. baumannii* and phages infecting *E. coli*, to phages infecting *S. aureus*. Furthermore, a statistically significant difference was also observed between phages infecting *A. baumannii* and phages infecting *E. coli* to those infecting *P. aeruginosa*. Phages infecting *S. aureus* exhibited the longest latent period amongst all analyzed groups of phages (1720.25 s), which was also supported by a Kruskal–Wallis H test. Similarly, *P. aeruginosa*-specific phages exhibited a long latent period (1657.11 s), and this group was also defined to be statistically different from phages infecting *A. baumannii* and phages infecting *E. coli*. It is important to mention that phages exhibiting shorter latent period are described to amplify more efficiently (Abedon et al. 2003). Therefore, a given benchmark of a mean latent period may be useful while seeking a promising candidate for future phage therapy. For example, phages with short latent periods tend to infect bacteria more efficiently under conditions of high bacterial density (Abedon et al. 2001; Shao and Wang 2008); therefore, this feature can be exploited for the targeted design and recruitment of phages for therapy. Conversely, in low host availability, phages with longer latent and/or lysis times may be preferential, as their infection dynamics allow them to remain effective (expressing mostly higher values of burst size (Abedon and Culler 2007; Kannoly et al. 2023)) despite limited bacterial targets. However, further research is needed to investigate the potential correlation within a latent period of varying lengths. It could involve a comprehensive analysis of the complexity of phage particles and/or phage genome lengths (and/or their G+C content), which might indicate that more time is needed to replicate the viral DNA and assemble new phages active against ESKAPE pathogens.

Lysis time refers to the period from phage infection until the bacterial cell is lysed by phage-encoded enzymes, resulting in the release of progeny virions (Olszak et al. 2017). Although the highest mean in terms of lysis time was calculated for phages infecting *S. aureus* (2605.56 s), it was not qualified for the Kruskal–Wallis H test, as a result of too few data characterizing phages infecting this pathogen in terms of their lysis time. Hypothetically, phages infecting Gram-positive pathogens are facing a thick peptidoglycan layer (Zhydzetski et al. 2024), which may possibly lengthen the lysis period; however, *Enterococcus*-specific phages analyzed in this review exhibited a shorter lysis period (2182.35 s; Table 1) than K. pneumoniae phages (2448.21 s), A. baumannii phages (2510.53 s), Enterobacter phages (2236.36 s), and P. aeruginosa phages (2221.3 s). Regarding the qualified data for the Kruskal–Wallis H test (concerning phages infecting *K. pneumoniae*, *P. aeruginosa,* and *E. coli*; mentioned in previous section), no statistically significant differences were observed. Nonetheless, more phages infecting ESKAPE pathogens need to be characterized in order to evaluate and discuss those differences. However, since lysis of bacterial cells from within is caused by different phage enzymes destroying bacterial layers (Young 2013; Oliveira et al. 2022), the differences in lysis time duration may be correlated with the presence of genes encoding those enzymes. Furthermore, it was described that a short adsorption time can be associated with short lysis time, which can be supported by analysed phages infecting *E. coli*, exhibiting both shortest adsorption time and shortest lysis time within this review (Shao and Wang 2008). Nevertheless, more research regarding the genetic analysis of phage genomes, compared to the obtained lysis time, is proposed, in order to support these results.

The highest differences in this review (regarding to the Kruskal–Wallis H test) were evident while analyzing burst size, which is the number of progeny phages released from a single infected bacterial cell following lysis (Jończyk-Matysiak et al. 2019). Statistically significant diference was described for phages infecting *K. pneumoniae* (mean – 139.80 PFU/cell)*,* phages infecting *A. baumannii* (mean – 180.21 PFU/cell), phages infecting *P. aeruginosa* (mean – 153.04 PFU/cell), and phages infecting *E. coli* (mean – 197 PFU/cell), when compared to phages infecting *S. aureus* (mean burst size – 97.70 PFU/cell). Furthermore, a statistically significant difference was also observed between phages infecting *Enterococcus* spp. (mean – 128.04 PFU/cell) and phages infecting *K. pneumoniae* (mean – 139.80 PFU/cell) to phages infecting *A. baumannii* (mean – 180.21 PFU/cell). While analyzing solely the mean burst size, phages infecting *S. aureus* and phages infecting *Enterococcus* spp. gathered in this review, exhibited one of the lowest burst sizes amongst others, which was also supported by Kruskal–Wallis H test. This might be related to the fact that both pathogens are Gram-positive bacteria. The number of progeny phages is determined by several factors, as some of them require manipulating the host's translational machinery (Wolfram-Schauerte et al. 2023). Furthermore, phage burst size is also associated with the growth of the bacterial host strain and the lysis time (Hadas et al. 1997; Nabergoj et al. 2018; Kannoly et al. 2023), which is consistent with the results obtained in terms of the lysis time exhibited by phages infecting *S. aureus* within this review. Nevertheless, the most optimal lysis time, indicating the best fitness (growth rate) was defined for phages exhibiting an intermediate lysis time (Wang 2006) but more phages and host species ought to be added to this database in order to point out a defininte intermediate value. Some phages infecting Gram-negative pathogens may also cause lysis inhibition, which has been proven to increase the burst size (Abedon 2019; Hays and Seed 2020). Furthermore, it is known that *S. aureus* (unlike, for example, *E. coli*) does not undergo a multifork DNA replication, which may possibly lengthen its cell growth, potentially lowering the velocity of bacterial host strain growth (Barbuti et al. 2023).

Several publications reported phages with broad lytic spectra, active against bacterial isolates from different geographical regions. However, some studies emphasized the narrow host range of specific phages, which, while limiting in monotherapy, could support the development of targeted phage cocktails. Additionally, the high specificity of bacteriophages suggests they are less likely to disturb the natural microbiota when administered to patients (Olawade et al. 2024; Cieślik et al. 2025a, b). Given that 50% of phages infecting *E. coli* and *Enterobacter* spp. exhibited polyvalent activity, this observation underscores the practical value of routinely assessing these phages against additional clinically relevant hosts. Such cross-screening would allow phage therapy centers to identify phages that may retain lytic activity when primary, species-specific options fail – thereby expanding therapeutic flexibility in difficult or rapidly evolving infections.

Importantly, it was possible to observe that phages infecting Gram-negative pathogens could produce a ‘halo’ effect more often than phages infecting Gram-positive ESKAPE pathogens. This may be related to the depolymerases they encode, which can degrade bacterial extracellular polysaccharides (Pires et al. 2016). Furthermore, phage depolymerases have been shown to help phages penetrate through biofilm structure (Ferriol-González and Domingo-Calap 2020; Topka-Bielecka et al. 2020) and eradicate biofilm more efficiently. Moreover, newly isolated depolymerases can act independently as antimicrobial agents (Guo et al. 2017; Liu et al. 2019a, b; Topka-Bielecka et al. 2020).

The relationship between the morphology of phage virions and their infection cycles may involve, among other factors, the functional roles of specific tail proteins. Tailed bacteriophages that encode particular tail-associated proteins (e.g., gp9, gp26, expressed by P22 phage infecting *Salmonella enterica* serovar Typhimurium) may exhibit infection parameters influenced by these proteins, such as host recognition or the capacity of virions to navigate through their environment (Hu et al. 2013; Wang et al. 2019a, b). It is also worth noting that approximately 96% of the described known bacteriophages belong to the tailed viruses of the group classified as *Caudoviricetes,* and thus exhibit similar features, even at the molecular level (Fokine and Rossmann 2014; Turner et al. 2023).

In this publication, only lytic bacteriophages were considered. Overall, the biological diversity of phages described in the literature underlines their therapeutic potential but also highlights the need for standardization in phage selection and characterization protocols. It is crucial to conduct a detailed biological characterization of phages before their clinical application, particularly including the analysis of adsorption time and lysis time.

## Conclusions

This review provides a comparative analysis of the infection kinetics of strictly lytic bacteriophages targeting ESKAPE and other crucial pathogens (represented by *E. coli*), offering insights into differences in adsorption, latency, lysis time, and burst size among diverse phage-host systems. Despite recent advances, the current dataset remains limited, highlighting the pressing need for a broader collection of well-characterized phages. In particular, future research should include comparative analyses of phage genomic features, which may help to explain the observed variability in phage infection dynamics. Expanding the pool of phages with well-defined biological and genomic traits is crucial not only for enhancing our understanding of phage-bacteria interactions but also for strengthening the rationale for therapeutic applications against MDR pathogens.

## Supplementary Information

Below is the link to the electronic supplementary material.

Supplementary file1 (DOCX 54 KB)

Supplementary file2 (DOCX 87 KB)

Supplementary file3 (DOCX 133 KB)

Supplementary file4 (DOCX 81 KB)

Supplementary file5 (DOCX 91 KB)

Supplementary file6 (DOCX 41 KB)

Supplementary file7 (DOCX 96 KB)

## Data Availability

Data will be made available on request.

## Abbreviations

WHO: World Health Organization
MDR: Multidrug resistant
ESKAPE: *Enterococcus* spp., *Staphylococcus aureus*, *Klebsiella pneumoniae*, *Acinetobacter baumannii*, *Pseudomonas aeruginosa*, *Enterobacter* spp.
UTI: Urinary tract infection
HAI: Healthcare-associated infection
MRSA: Methicillin-resistant *Staphylococcus aureus*
CRAB: Carbapenem-resistant *Acinetobacter baumannii*
VAP: Ventilator-associated pneumonia
ICU: Intensive care unit
HGT: Horizontal gene transfer
RBP: Receptor binding protein
PFU: Plaque forming unit
MOI: Multiplicity of infection
SD: Standard deviation
SE: Standard error of the mean
