# Supplementary material for: A comprehensive analysis of the kinetics of infection of lytic bacteriophages specific to the ESKAPE and critical pathogens
Source: World J Microbiol Biotechnol. 2026 Feb 28;42(3):110. doi: 10.1007/s11274-025-04762-4 (PMC12950090; doi:10.1007/s11274-025-04762-4)
Supplement: Supplementary file 1 — Supplementary file1 (DOCX 54 KB) [file 11274_2025_4762_MOESM1_ESM.docx]

**Supplementary Table S1** – Collected data regarding to phages infecting *Enterococcus* spp. in terms of cycle parameters.

| **Phage designation** | **Host species** | **Host strain (source of isolation, if given)** | **Results of the studied multiplicity of infection (MOI)** | **Adsorption time [s]** | **Latent period [s]** | **Lysis time [s]** | **Burst size [PFU/cell]** | **Reference** |
| --- | --- | --- | --- | --- | --- | --- | --- | --- |
| SAM-E.f 12 | *E. faecalis* | VRE *E. faecalis* (clinical isolate) | 0.1 | 300 | 1200 | no data | 5.7 | Abed et al., 2024 |
| EFP6 | *E. faecalis* | EF6 (animal) | 0.001 | no data | 600 | no data | 127 | Ahmad et al., 2024 |
| SHEF2 | *E. faecalis* | OS16 (clinical) | 0.1 | no data | 1800 | 2900 | 9.3 | Al-Zubidi et al., 2019 |
| iF6 | *E. faceium* | FS86 (reference strain) | 0.1 | 60 | 4800 | 1800 | 31 | Buzikov et al., 2023 |
| EF-P29 | *E. faecalis* | GF29 (clinical isolate) | 0.01 | no data | 1800 | no data | 90 | Cheng et al., 2017 |
| vB_EfaH_EF1TV | *E. faecalis* | 1/1112 (clinical isolate) | 0.01 | no data | 900 | 900 | 27 | D'Andrea et al., 2020 |
| vB_ZEFP | *E. faecalis* | EF1, EF2, EF4, EF5, EF7, EF8, EF9, EF10, EF12 and EF13 (clinical isolates) | 0.1 | no data | 600 | no data | 110 | El-Telbany et al., 2021 |
| vB_EfKS5 | *E. faecalis* | 7 (clinical isolate) | 1 | no data | 1200 | no data | 183.33 | El-Telbany et al., 2023 |
| IME-EF5 | *E. faceium* | 4P-SA (clinical isolate) | 0.01 | no data | 1800 | no data | no data | Gong et al., 2016 |
| S2 | *E. faceium* | VREfm2 (no data) | 0.0001 | no data | 840 | 5700 | 200 | Goodarzi et al., 2021 |
| vB_EfaS_efap05-1 | *E. faecalis* | efa05 (clinical) | 10 | no data | 600 | 600 | 20 | Huang et al., 2022 |
| EF-N13 | *E. faecalis* | N13 (animal) | no data | no data | 300 | no data | 284 | Ji et al., 2023 |
| ZXL-01 | *E. faecalis* | HZB-01 (no data) | 0.1 | no data | 1200 | 4200 | 30 | Jiang et al., 2025 |
| vB_EfaS_WH1 | *E. faecalis* | EF01 (clinical) | 0.1 | no data | 600 | 2400 | 70 | Jin et al., 2023 |
| EFA1 | *E. faecalis* | no data (environmental) | 0.1 | no data | 1200 | 600 | 120 | Kabwe et al., 2021 |
| EF-M80 | *E. faceium* | *E. faecium* ATCC 1959 (reference strain) | 0.1 | no data | 1200 | no data | 12.6 | Khazani Asforooshani et al., 2024 |
| vB_EfaS_HEf13 | *E. faecalis* | KCOM 1162 (clinical) | 0.01 | no data | 1500 | 1800 | 352 | Lee et al., 2019 |
| VE1853 | *E. faecalis* | V583 (clinical) | 0.001 | no data | 900 | no data | 50 | Lossouarn et al., 2019 |
| vB_EfaS-Zip | *E. faceium* | C410 (no data) | no data | no data | 1500 | no data | 52 | Melo et al., 2021 |
| vB_EfaP-Max | *E. faecalis* | Efa1 (clinical) | no data | no data | 600 | no data | 38 | Melo et al., 2021 |
| vB_Efa29212_2e | *E. faecalis* | ATCC 29212 (reference strain) | no data | no data | 1500 | no data | 139 | Moryl et al., 2022 |
| vB_Efa29212_3e | *E. faecalis* | ATCC 29212 (reference strain) | no data | no data | 3000 | no data | 127 | Moryl et al., 2022 |
| PBEF129 | *E. faecalis* | CCARM5520 (reference strain) | no data | no data | 1800 | no data | 83 | Oh et al., 2021 |
| φ4D | *E. faecalis* | ATCC 51299 (reference strain) | 0.1 | no data | 1500 | no data | 36 | Parasion et al., 2012 |
| vB_EfaS-SRH2 | *E. faecalis* | ATCC 29212 (reference strain) | 1 | 600 | 900 | 900 | 125 | Pazhouhnia et al., 2022 |
| vB_EfaS_LOK1 | *E. faecalis* | IIH-74.4 (clinical) | 10 | no data | 1200 | no data | 72 | Plata Suarez et al., 2025 |
| vB_EfaH_163 | *E. faceium* | LMGY1 (animal) | 0.1 | no data | 4800 | no data | 155 | Pradal et al., 2023 |
| vB_Efm_LG62 | *E. faceium* | LG62 (clinical) | 10 | 3000 | 1200 | 6000 | 101 | Qu et al., 2023 |
| SRG1 | *E. faecalis* | KF448071 [gene accesion number] | no data | no data | 1260 | no data | 512 | Rahmat Ullah et al., 2017 |
| *E. faecalis*_phage-01 | *E. faecalis* | A.R.A.01 (clinical) | 0.01 | no data | 1800 | no data | 115.76 | Ramadan et al., 2024 |
| *E. faecalis*_phage-02 | *E. faecalis* | A.R.A.02 (clinical) | 0.01 | no data | 1500 | no data | 80,6 | Ramadan et al., 2024 |
| STH1 | *E. faceium* | 1969 (clinical) | no data | no data | 1080 | no data | 334 | Raza et al., 2018 |
| vB_GEC_EfS_9 | *E. faceium* | no data (clinical) | 0.1 | 900 | 1380 | no data | 222 | Rigvava et al., 2022 |
| RG1 | *E. faecium* | ATCC 35667 (reference strain) | 0.001 | no data | 600 | no data | 61 | Singh et al., 2025 |
| vB_EfaS-DELF1 | *E. faecalis* | ATCC 29212 (reference strain) | 1 | 1500 | 2400 | 2400 | 87 | Soleimani-Delfan et al., 2021 |
| vB_EfaS-SFQ1 | *E. faecalis* | EFS01 (clinical) | 0.01 | no data | 600 | 1800 | 110 | Song et al., 2023 |
| vB_EfaM_LG1 | *E. faecalis* | ef118 (clinical) | 0.001 | 900 | 600 | 1200 | 40 | Song et al., 2021 |
| vB_Efs8_KEN04 | *E. faecalis* | V583 (clinical) | 1 | 600 | 1200 | no data | 138.46 | Soro et al., 2024 |
| ENP2309 | E. faecalis | GZ16185 (animal) | 0.001 | no data | 2400 | 4800 | 920 | Tian et al., 2025 |
| GVEsP-1 | *E. faecalis* | CCUG 52538 (reference strain) | 0.01 | no data | 1200 | no data | 94 | Tkachev et al., 2022 |
| SSsP-1 | *E. faecalis* | Serg (clinical) | 0.01 | no data | 1080 | no data | 66 | Tkachev et al., 2022 |
| vB_EfaS-271 | *E. faecalis* | 271 (clinical) | 0.05 | no data | 480 | no data | 70 | Topka-Bielecka et al., 2020 |
| phiEF24C | *E. faecalis* | EF24 (clinical) | no data | 300 | 1800 | no data | 110 | Uchiyama et al., 2008 |
| Ef212 | *E. faecalis* | ATCC 29212 (reference strain) | 0.01 | no data | 300 | no data | 202 | Uskudar-Guclu & Yalcin, 2024 |
| vB_EfaP_IME195 | *E. faecalis* | 2003 (clinical) | 0.01 | no data | 1800 | no data | 120 | Wang et al., 2018 |
| vB_EfaP_IME199 | *E. faceium* | *E. faecium* 1007 (environmental) | 0.01 | no data | 1200 | 1800 | 30 | Xing et al., 2017 |
| vB_EfaS_PHB08 | *E. faecalis* | EF3964 (clinical) | 0.01 | no data | 600 | 3000 | 64 | Yang et al., 2020 |
| XWef1 | *E. faecium* | XW113 (no data) | 0.01 | no data | 1200 | 2400 | 57 | Yuan et al.., 2025 |
| BUCT630 | *E. faecium* | 2083 (no data) | 0.01 | 3600 | 1800 | 3600 | 130 | Zakirullah et al., 2025 |
| IME-EF1 | *E. faecalis* | *E. faecalis* 002 (clinical) | 0.1 | 540 | 1500 | no data | 60 | Zhang et al., 2013 |

**Supplementary Table S2** – Collected data regarding to phages infecting *Enterococcus* spp. in terms of presence of ‘halo’ effect, type of phage morphology, phage gene accesion number.

| **Phage designation** | **Host species** | **Host strain (source of isolation, if given)** | **Presence of 'halo' effect** | **Type of phage morphology** | **Phage gene accesion number** | **Reference** |
| --- | --- | --- | --- | --- | --- | --- |
| SAM-E.f 12 | *E. faecalis* | VRE *E. faecalis* (clinical isolate) | no | siphovirus | OR762047 | Abed et al., 2024 |
| EFP6 | *E. faecalis* | EF6 (animal) | no data | podovirus | PP836395.1 | Ahmad et al., 2024 |
| SHEF2 | *E. faecalis* | OS16 (clinical) | no | siphovirus | MF678788 | Al-Zubidi et al., 2019 |
| iF6 | *E. faceium* | FS86 (reference strain) | no | myovirus | MT909815 | Buzikov et al., 2023 |
| EF-P29 | *E. faecalis* | GF29 (clinical isolate) | no | siphovirus | GKY303907 | Cheng et al., 2017 |
| vB_EfaH_EF1TV | *E. faecalis* | 1/1112 (clinical isolate) | no | myovirus | MK268686.1 | D'Andrea et al., 2020 |
| vB_ZEFP | *E. faecalis* | EF1, EF2, EF4, EF5, EF7, EF8, EF9, EF10, EF12 and EF13 (clinical isolates) | no data | podovirus | MT747434 | El-Telbany et al., 2021 |
| vB_EfKS5 | *E. faecalis* | 7 (clinical isolate) | no data | siphovirus | OQ297175 | El-Telbany et al., 2023 |
| IME-EF5 | *E. faceium* | 4P-SA (clinical isolate) | no | siphovirus | KT588072 | Gong et al., 2016 |
| S2 | *E. faceium* | VREfm2 (no data) | no | siphovirus | no data | Goodarzi et al., 2021 |
| vB_EfaS_efap05-1 | *E. faecalis* | efa05 (clinical) | no | siphovirus | OL505085 | Huang et al., 2022 |
| EF-N13 | *E. faecalis* | N13 (animal) | no data | myovirus | ON352054 | Ji et al., 2023 |
| ZXL-01 | *E. faecalis* | HZB-01 (no data) | no | siphovius | ON113334 | Jiang et al., 2025 |
| vB_EfaS_WH1 | *E. faecalis* | EF01 (clinical) | no | siphovirus | PRJNA939409 | Jin et al., 2023 |
| EFA1 | *E. faecalis* | no data (environmental) | no | siphovirus | MT857001 | Kabwe et al., 2021 |
| EF-M80 | *E. faceium* | *E. faecium* ATCC 1959 (reference strain) | no data | myovirus | OR767211 | Khazani Asforooshani et al., 2024 |
| vB_EfaS_HEf13 | *E. faecalis* | KCOM 1162 (clinical) | no data | siphovirus | MH618488 | Lee et al., 2019 |
| VE1853 | *E. faecalis* | V583 (clinical) | no | podovirus | LT630001.1 | Lossouarn et al., 2019 |
| vB_EfaS-Zip | *E. faceium* | C410 (no data) | no data | siphovirus | MK360025 | Melo et al., 2021 |
| vB_EfaP-Max | *E. faecalis* | Efa1 (clinical) | no data | podovirus | MK360024 | Melo et al., 2021 |
| vB_Efa29212_2e | *E. faecalis* | ATCC 29212 (reference strain) | no data | siphovirus | OP559177 | Moryl et al., 2022 |
| vB_Efa29212_3e | *E. faecalis* | ATCC 29212 (reference strain) | no data | myovirus | OP559178 | Moryl et al., 2022 |
| PBEF129 | *E. faecalis* | CCARM5520 (reference strain) | no | siphovirus | MN854830.2 | Oh et al., 2021 |
| φ4D | *E. faecalis* | ATCC 51299 (reference strain) | no data | myovirus | no data | Parasion et al., 2012 |
| vB_EfaS-SRH2 | *E. faecalis* | ATCC 29212 (reference strain) | no | siphovirus | LC623721.1 | Pazhouhnia et al., 2022 |
| vB_EfaS_LOK1 | *E. faecalis* | IIH-74.4 (clinical) | yes | siphovius | PV780768 | Plata Suarez et al., 2025 |
| vB_EfaH_163 | *E. faceium* | LMGY1 (animal) | no | myovirus | CAJDKA010000002.1 | Pradal et al., 2023 |
| vB_Efm_LG62 | *E. faceium* | LG62 (clinical) | no | siphovirus | OP018674 | Qu et al., 2023 |
| SRG1 | *E. faecalis* | KF448071 [gene accesion number] | no | myovirus | no data | Rahmat Ullah et al., 2017 |
| *E. faecalis*_phage-01 | *E. faecalis* | A.R.A.01 (clinical) | yes | siphovirus | ON809698 | Ramadan et al., 2024 |
| *E. faecalis*_phage-02 | *E. faecalis* | A.R.A.02 (clinical) | yes | myovirus | ON809699 | Ramadan et al., 2024 |
| STH1 | *E. faceium* | 1969 (clinical) | no | myovirus | no data | Raza et al., 2018 |
| vB_GEC_EfS_9 | *E. faceium* | no data (clinical) | no data | siphovirus | MW672041 | Rigvava et al., 2022 |
| RG1 | *E. faecium* | ATCC 35667 (reference strain) | no | siphovius | PQ586903 | Singh et al., 2025 |
| vB_EfaS-DELF1 | *E. faecalis* | ATCC 29212 (reference strain) | no | siphovirus | LC513943.1 | Soleimani-Delfan et al., 2021 |
| vB_EfaS-SFQ1 | *E. faecalis* | EFS01 (clinical) | no | siphovirus | OQ831052 | Song et al., 2023 |
| vB_EfaM_LG1 | *E. faecalis* | ef118 (clinical) | no | myovirus | MZ420150 | Song et al., 2021 |
| vB_Efs8_KEN04 | *E. faecalis* | V583 (clinical) | no | siphovirus | MN854830.2 | Soro et al., 2024 |
| ENP2309 | E. faecalis | GZ16185 (animal) | no | myovirus | PQ787220 | Tian et al., 2025 |
| GVEsP-1 | *E. faecalis* | CCUG 52538 (reference strain) | yes | myovirus | MZ333462 | Tkachev et al., 2022 |
| SSsP-1 | *E. faecalis* | Serg (clinical) | yes | siphovirus | MZ333457 | Tkachev et al., 2022 |
| vB_EfaS-271 | *E. faecalis* | 271 (clinical) | no | siphovirus | MT520979.1 | Topka-Bielecka et al., 2020 |
| phiEF24C | *E. faecalis* | EF24 (clinical) | no | myovirus | AP009390.1 | Uchiyama et al., 2008 |
| Ef212 | *E. faecalis* | ATCC 29212 (reference strain) | yes | siphovirus | OR052631 | Uskudar-Guclu & Yalcin, 2024 |
| vB_EfaP_IME195 | *E. faecalis* | 2003 (clinical) | no | podovirus | KT932700 | Wang et al., 2018 |
| vB_EfaP_IME199 | *E. faceium* | *E. faecium* 1007 (environmental) | no data | podovirus | KT945995.1 | Xing et al., 2017 |
| vB_EfaS_PHB08 | *E. faecalis* | EF3964 (clinical) | no | siphovirus | MK570225.1 | Yang et al., 2020 |
| XWef1 | *E. faecium* | XW113 (no data) | no | siphovius | PV491271 | Yuan et al.., 2025 |
| BUCT630 | *E. faecium* | 2083 (no data) | no | siphovius | PP434460.1 | Zakirullah et al., 2025 |
| IME-EF1 | *E. faecalis* | *E. faecalis* 002 (clinical) | no | siphovirus | KF192053 | Zhang et al., 2013 |

**Supplementary Table S3** – Collected data regarding to phages infecting *Enterococcus* spp. in terms of host range and polyvalence.

| **Phage designation** | **Host species** | **Host strain (source of isolation, if given)** | **Host range of the bacteriophage against *Enterococcus* spp. strains (same as host species; vulnerable/tested)** | **Percentage of host range** | **Activity against other species** | **Tested other species (number of tested strains)** | **Reference** |
| --- | --- | --- | --- | --- | --- | --- | --- |
| SAM-E.f 12 | *E. faecalis* | VRE *E. faecalis* (clinical isolate) | 42/60 | 70% | no | *E. faecium* (no data);  *S. aureus* (no data);  *P. aeruginosa* (no data);  *E. coli* (no data) | Abed et al., 2024 |
| EFP6 | *E. faecalis* | EF6 (animal) | 4/10 | 40% | no | *A. baumannii* (2);  *Acidovorax* (1)  *B. bacterium* (1);  *E. coli* (12);  *K. pneumoniae* (2);  *P. mirabilis* (8);  *S. aureus* (2);  *S. argenteus* (6) | Ahmad et al., 2024 |
| SHEF2 | *E. faecalis* | OS16 (clinical) | 9/13 | 69.23% | no data |  | Al-Zubidi et al., 2019 |
| iF6 | *E. faceium* | FS86 (reference strain) | 5/11 | 45.45% | yes: *E. thailandicus* | *E. avium* (1);  *E. durans* (3);  *E. hirae* (1);  *E. faecalis* (3);  *E. thailandicus* (1);  *B. cereus* (1);  *P. syringae* (1);  *E. coli* (1) | Buzikov et al., 2023 |
| EF-P29 | *E. faecalis* | GF29 (clinical isolate) | 17/40 | 42.50% | no | *E. faecium* (2);  *P. aeruginosa* (3);  *E. coli* (3);  *S. aureus* (3);  *B. subtilis* (3); *Streptococcus sp.* (3);  *K. pneumoniae* (3) | Cheng et al., 2017 |
| vB_EfaH_EF1TV | *E. faecalis* | 1/1112 (clinical isolate) | 17/17 | 100% | no | *E. faecium* (6);  *S. aureus* (1) | D'Andrea et al., 2020 |
| vB_ZEFP | *E. faecalis* | EF1, EF2, EF4, EF5, EF7, EF8, EF9, EF10, EF12 and EF13 (clinical isolates) | 10/13 | 76.92% | no | *S. mutans* (1);  *E. gallinarum* (1);  *E. faecium* (1);  *E. coli* (1);  *P. aeruginosa* (1);  *S. aureus* (1) | El-Telbany et al., 2021 |
| vB_EfKS5 | *E. faecalis* | 7 (clinical isolate) | 22/29 | 75.86% | yes: *E. faecium* | *E. faecium* (7) | El-Telbany et al., 2023 |
| IME-EF5 | *E. faceium* | 4P-SA (clinical isolate) | 1/24 | 4.17% | no | *E. faecalis* (2);  *P. aeruginosa* (1);  *E. coli* (1);  *S. aureus* (1);  *B. subtilis* (1);  *Salmonella* sp. (1);  *K. pneumoniae* (1);  *Streptococcus* sp. (1) | Gong et al., 2016 |
| S2 | *E. faceium* | VREfm2 (no data) | 28/34 | 82.35% | no | *E. gallinarum* (1);  *E. faecalis* (13);  *E. coli* (6) | Goodarzi et al., 2021 |
| vB_EfaS_efap05-1 | *E. faecalis* | efa05 (clinical) | 5/10 | 50% | no data |  | Huang et al., 2022 |
| EF-N13 | *E. faecalis* | N13 (animal) | no data | no data | no data |  | Ji et al., 2023 |
| ZXL-01 | *E. faecalis* | HZB-01 (no data) | 15/30 | 50% | no data |  | Jiang et al., 2025 |
| vB_EfaS_WH1 | *E. faecalis* | EF01 (clinical) | 12/20 | 60% | no | *E. faecium* (1);  *C. perfringens* (1);  *S. aureus* (1);  *E. coli* (2);  *S. enterica* serovar Enteritidis (1);  *S. enterica* serovar Pullmorum (2);  *S. enterica* serovar Typhimurium (1);  *K. pneumoniae* (1) | Jin et al., 2023 |
| EFA1 | *E. faecalis* | no data (environmental) | no data | no data | no | *S. mutans* (no data);  *S. moorei* (no data);  *E. coli* (no data);  *A. hydrophila* (no data);  *L. casei* (no data);  *S. moorei* (no data);  *F. nucleatum* (no data) | Kabwe et al., 2021 |
| EF-M80 | *E. faceium* | *E. faecium* ATCC 1959 (reference strain) | 18/30 | 60% | no | *E. faecalis* (no data);  *E. coli* (no data);  *S. aureus* (no data);  *S. pneumoniae* (no data);  *K. pneumoniae* (no data);  *P. aeruginosa* (no data) | Khazani Asforooshani et al., 2024 |
| vB_EfaS_HEf13 | *E. faecalis* | KCOM 1162 (clinical) | 12/17 | 70.59% | no | *E. faecium* (2) | Lee et al., 2019 |
| VE1853 | *E. faecalis* | V583 (clinical) | no data | no data | no data |  | Lossouarn et al., 2019 |
| vB_EfaS-Zip | *E. faceium* | C410 (no data) | 3/13 | 23.08% | yes: *E. faecalis* | *E. gallinarum* (1);  *S. aureus* (2);  *S. epidermidis* (1);  *L. monocytogenes* (1);  *E. faecalis* (16) | Melo et al., 2021 |
| vB_EfaP-Max | *E. faecalis* | Efa1 (clinical) | 12/16 | 75% | yes: *E. faecium* | *E. gallinarum* (1);  *S. aureus* (2);  *S. epidermidis* (1);  *L. monocytogenes* (1);  *E. faecium* (13) | Melo et al., 2021 |
| vB_Efa29212_2e | *E. faecalis* | ATCC 29212 (reference strain) | no data | no data | no data |  | Moryl et al., 2022 |
| vB_Efa29212_3e | *E. faecalis* | ATCC 29212 (reference strain) | no data | no data | no data |  | Moryl et al., 2022 |
| PBEF129 | *E. faecalis* | CCARM5520 (reference strain) | 9/11 | 81.82% | no data |  | Oh et al., 2021 |
| φ4D | *E. faecalis* | ATCC 51299 (reference strain) | 29/34 | 85% | yes: *E. faecium* and *E. hirae* | *E. faecium* (1);  *E. hirae* (1) | Parasion et al., 2012 |
| vB_EfaS-SRH2 | *E. faecalis* | ATCC 29212 (reference strain) | 6/6 | 100% | no | *P. aeruginosa* (1);  *E. coli* (1);  *S. enterica* serovar Typhimurium (1);  *S. sonnei* (1);  *S. aureus* (1);  *S. mutans* (1);  *S. saliviarius* (1);  *B. cereus* (1) | Pazhouhnia et al., 2022 |
| vB_EfaS_LOK1 | E. faecalis | IIH-74.4 (clinical) | 1/8 | 12.5% | no | *S. aureus* (5);  *A. baumannii* (2);  *K. pneumoniae* (1);  *P. aeruginosa* (1);  *E. coli* (1);  *E. cloacae* (1);  *E. faecium* (11) | Plata Suarez et al., 2025 |
| vB_EfaH_163 | *E. faceium* | LMGY1 (animal) | 33/77 | 42.86% | yes: *E. faecalis* | *E. faecalis* (12) | Pradal et al., 2023 |
| vB_Efm_LG62 | *E. faceium* | LG62 (clinical) | 2/12 | 16.67% | no | *E. faecalis* (4) | Qu et al., 2023 |
| SRG1 | *E. faecalis* | KF448071 [gene accesion number] | 3/8 | 37.50% | no | *E. faecium* (3);  *E. coli* (3);  *Acinetobater* sp. (1);  *S. aureus* (2);  *P. aeruginosa* (1);  *Citrobacter* sp. (1) | Rahmat Ullah et al., 2017 |
| *E. faecalis*_phage-01 | *E. faecalis* | A.R.A.01 (clinical) | no data | no data | no | *E. faecium* (1);  *S. mutans* (1);  *S. aureus* (1);  *E. coli* (1);  *P. aeruginosa* (1) | Ramadan et al., 2024 |
| *E. faecalis*_phage-02 | *E. faecalis* | A.R.A.02 (clinical) | no data | no data | no | *E. faecium* (1);  *S. mutans* (1);  *S. aureus* (1);  *E. coli* (1);  *P. aeruginosa* (1) | Ramadan et al., 2024 |
| STH1 | *E. faceium* | 1969 (clinical) | 2/6 | 28.57% | no | *E. coli* (4);  *S. aureus* (5);  *P. aeruginosa* (3) | Raza et al., 2018 |
| vB_GEC_EfS_9 | *E. faceium* | no data (clinical) | 59/70 | 84.29% | no | *E. faecalis* (30);  *S. agalicticae* (20);  *S. mitis* (10);  *S. aureus* (10) | Rigvava et al., 2022 |
| RG1 | *E. faecium* | ATCC 35667 (reference strain) | 17/19 | 89.47% | no | *B. cepacia* (1);  *P. aeruginosa* (1);  *P. mirabilis* (1);  *K. oxytoca* (1);  *E. faecalis* (1);  *S. aureus* (1);  *D. radiodurans* (1) | Singh et al., 2025 |
| vB_EfaS-DELF1 | *E. faecalis* | ATCC 29212 (reference strain) | 7/7 | 100% | no | *E. mundtii* (1);  *E. faecium* (1);  *S. aureus* (2);  *S. agalactiae* (1);  *S. pyogenes* (1);  *E. coli* (1);  *S. epidermidis* (1);  *S. saprophyticus* (1);  *K. pneumoniae* (1);  *P. aeruginosa* (1);  *P. mirabilis* (1);  *L. delbrueckii subsp. bulgaricus* (1);  *S. salivarius subsp. thermophilus* (1);  *L. acidophilus* (1);  *C. albicans* (1);  *S. cerevisiae* (1) | Soleimani-Delfan et al., 2021 |
| vB_EfaS-SFQ1 | *E. faecalis* | EFS01 (clinical) | 6/10 | 60% | no | *E. faecium* (5) | Song et al., 2023 |
| vB_EfaM_LG1 | *E. faecalis* | ef118 (clinical) | 5/14 | 35.71% | no data |  | Song et al., 2021 |
| vB_Efs8_KEN04 | *E. faecalis* | V583 (clinical) | 26/26 | 100% | yes: *E. faecium* | *E. faecium* (11) | Soro et al., 2024 |
| ENP2309 | *E. faecalis* | GZ16185 (animal) | 13/17 | 81.25% | no | *E. gallinarum* (1);  *E. casseliflavus* (1);  *E. faecium* (1) | Tian et al., 2025 |
| GVEsP-1 | *E. faecalis* | CCUG 52538 (reference strain) | 14/39 | 35.90% | yes: *E. faecium* | *E. faecium* (24);  *E. gallinarum* (2);  *E. casseliflavus* (1);  *E. hirae* (1);  *E. durans* (1);  *S. aureus* (5);  *S. epidermidis* (1);  *S. agalactiae* (3);  *S. pyogenes* (4);  *B. longum* (1);  *E. coli* (1) | Tkachev et al., 2022 |
| SSsP-1 | *E. faecalis* | Serg (clinical) | 24/39 | 61.54% | yes: *E. faecium* | *E. faecium* (24);  *E. gallinarum* (2);  *E. casseliflavus* (1);  *E. hirae* (1);  *E. durans* (1);  *S. aureus* (5);  *S. epidermidis* (1);  *S. agalactiae* (3);  *S. pyogenes* (4);  *B. longum* (1);  *E. coli* (1) | Tkachev et al., 2022 |
| vB_EfaS-271 | *E. faecalis* | 271 (clinical) | 3/9 | 33.33% | no data |  | Topka-Bielecka et al., 2020 |
| phiEF24C | *E. faecalis* | EF24 (clinical) | 31/35 | 88.57% | no | *E. faecium* (10);  *S. aureus* (2);  *E. coli* (1) | Uchiyama et al., 2008 |
| Ef212 | *E. faecalis* | ATCC 29212 (reference strain) | 5/10 | 50% | no data |  | Uskudar-Guclu & Yalcin, 2024 |
| vB_EfaP_IME195 | *E. faecalis* | 2003 (clinical) | 2/26 | 7.69% | no | *E. faecium* (28);  *S. aureus* (5);  *E. coli* (5) | Wang et al., 2018 |
| vB_EfaP_IME199 | *E. faceium* | *E. faecium* 1007 (environmental) | no data | no data | no data |  | Xing et al., 2017 |
| vB_EfaS_PHB08 | *E. faecalis* | EF3964 (clinical) | 15/19 | 78.95% | no | *E. faecium* (10);  *E. coli* (4);  *Salmonella* sp. (3) | Yang et al., 2020 |
| XWef1 | *E. faecium* | XW113 (no data) | 3/16 | 18.75% | no | *E. faecalis* (4);  *L. acidophilus* (2);  *L. plantarum* (2);  *P. acidilactici* (2) | Yuan et al.., 2025 |
| BUCT630 | *E. faecium* | 2083 (no data) | 9/15 | 60% | no | *Enterobacter* spp. (1);  *E. coli* (1);  *E. faecalis* (1) | Zakirullah et al., 2025 |
| IME-EF1 | *E. faecalis* | *E. faecalis* 002 (clinical) | 3/12 | 25% | yes: *E. faecium* | *E. faecium* (10);  *S. aureus* (4) | Zhang et al., 2013 |

**Supplementary Table S4** – Bacterial strain species used in analyzed studies.

| **Bacterial strain species used in studies** |
| --- |
| *Acidovorax* |
| *Acinetobacter baumannii* |
| *Aeromonas hydrophila* |
| *Bacillus cereus* |
| *Bacillus subtilis* |
| *Bifidobacterium longum* |
| *Burkholderia cepacia* |
| *Candida albicans* |
| *Clostridium perfringens* |
| *Deinococcus radiodurans* |
| *Enterococcus avium* |
| *Enterococcus casseliflavus* |
| *Enterococcus durans* |
| *Enterococcus faecalis* |
| *Enterococcus faecium* |
| *Enterococcus gallinarum* |
| *Enterococcus hirae* |
| *Enterococcus mundtii* |
| *Enterococcus thailandicus* |
| *Escherichia coli* |
| *Fusobacterium nucleatum* |
| *Klebsiella oxytoca* |
| *Klebsiella pneumoniae* |
| *Lactobacillus acidophilus* |
| *Lactobacillus casei* |
| *Lactobacillus delbrueckii subsp. Bulgaricus* |
| *Lactobacillus plantarum* |
| *Listeria monocytogenes* |
| *Pediococcus acidilactici* |
| *Pseudomonas aeruginosa* |
| *Pseudomonas mirabilis* |
| *Pseudomonas syringae* |
| *Saccharomyces cerevisiae* |
| *Salmonella enterica* serovar Enteritidis |
| *Salmonella enterica* serovar Pullorum |
| *Salmonella enterica* serovar Typhimurium |
| *Salmonella* sp. |
| *Shigella sonnei* |
| *Solobacterium moorei* |
| *Staphylococcus argenteus* |
| *Staphylococcus aureus* |
| *Staphylococcus epidermitidis* |
| *Staphylococcus saprophyticus* |
| *Streptococcus agalactiae* |
| *Streptococcus mitis* |
| *Streptococcus mutans* |
| *Streptococcus pyogenes* |
| *Streptococcus pyogenes* |
| *Streptococcus salivarius* |
| *Streptococcus salivarius subsp. Thermophilus* |
| *Streptococcus* sp. |
