# Supplementary material for: A comprehensive analysis of the kinetics of infection of lytic bacteriophages specific to the ESKAPE and critical pathogens
Source: World J Microbiol Biotechnol. 2026 Feb 28;42(3):110. doi: 10.1007/s11274-025-04762-4 (PMC12950090; doi:10.1007/s11274-025-04762-4)
Supplement: Supplementary file 2 — Supplementary file2 (DOCX 87 KB) [file 11274_2025_4762_MOESM2_ESM.docx]

**Supplementary Table S5** – Collected data regarding to phages infecting *S. aureus* in terms of cycle parameters.

| **Phage designation** | **Host strain (source of isolation, if given)** | **Results of the studied multiplicity of infection (MOI)** | **Adsorption time [s]** | **Latent period [s]** | **Lysis time [s]** | **Burst size [PFU/cell]** | **Reference** |
| --- | --- | --- | --- | --- | --- | --- | --- |
| DRA88 | MSSA H476 (clinical) | 0.001 | 300 | 1500 | no data | 76 | Alves et al., 2014 |
| K | WBG8343 (clinical) | 0.001 | 300 | 3600 | no data | 125 | Alves et al., 2014 |
| vB_SauM_LM12 | 2093367 (clinical) | 0.01 | no data | 1200 | 3000 | 52 | Barros et al., 2019 |
| MSa | A170 (clinical) | no data | no data | 1800 | no data | 80 | Capparelli et al., 2007 |
| SA97 | RN4220 (clinical) | 0.01 | 600 | 1200 | no data | 32 | Chang et al., 2015 |
| OPT-SA02 | SAU18 (no data) | 0.01 | no data | 3000 | no data | 14 | Cho et al., 2025 |
| CapO46 | O46 (animal) | 0.01 | 600 | 1200 | 1800 | 30 | Cunha et al., 2025 |
| UPMK_1 | MRSA t127/4 (no data) | 0.01 | 900 | 1200 | no data | 32 | Dakheel et al., 2019 |
| UPMK_2 | MRSA t223/20 (no data) | 0.01 | 900 | 900 | no data | 67 | Dakheel et al., 2019 |
| vB_SauM-UFV_DC4 | MRSA 3059 (animal) | 0.00001 | 600 | 2400 | no data | 3,4 | da Silva et al., 2023 |
| SA46-CTH2 | SA46 (clinical) | 0.01 | no data | 900 | no data | 182 | Duc et al., 2020 |
| LH1 | ST352 (no data) | no data | no data | 4200 | no data | 5 | El Haddad et al., 2013 |
| LH1-MUT | ST352 (no data | no data | no data | 4800 | no data | 23 | El Haddad et al., 2013 |
| MRSA phage | no data (envrionmental) | 10 | 900 | 1800 | no data | 32 | Elsayed et al., 2024 |
| vB_SauP_ASUmrsa123 | mrsa123 (clinical) | 10 | no data | 3300 | no data | 167 | El-Tawab et al., 2024 |
| Huma | ATCC 29213 (reference strain) | 0.01 | 600 | 4800 | no data | 45 | Fatemeh Sharifi et al., 2024 |
| Simurgh | ATCC 29213 (reference strain) | 0.01 | 600 | 3600 | no data | 40 | Fatemeh Sharifi et al., 2024 |
| LSA2308 | ATCC 25923 (reference strain) | 0.1 | no data | 1200 | no data | 407 | Fei Ma et al., 2021 |
| LSA2366 | SA027 (reference strain) | 0.1 | no data | 600 | no data | 258 | Fei Ma et al., 2021 |
| SAJK-IND | MTCC (reference strain) | 0.01 | no data | 750 | 4200 | 45 | Ganaie et al., 2018 |
| MSP | MTCC (reference strain) | 0.02 | no data | 1600 | 5400 | 27 | Ganaie et al., 2018 |
| vB_SauS-phiIPLA35 | Sa9 (clinical) | no data | no data | 1800 | no data | 27 | García et al., 2009 |
| vB_SauS-phiIPLA88 | Sa9 (clinical) | no data | no data | 1350 | no data | 45 | García et al., 2009 |
| vB_SauP_phiAGO1.3 | phiAGO1.3 (clinical) | 0.01 | no data | 1800 | no data | 35 | Głowacka-Rutkowska et al., 2019 |
| phiIPLA-RODI | IPLA16 (einvironmental) | 0.1 | 900 | 900 | no data | 25 | Gutiérrez et al., 2015 |
| SAH-1 | N315 (animal) | 0.001 | no data | 1200 | 1200 | 100 | Han et al., 2013 |
| Stau2 | S23 (clinical) | 0.0005 | 180 | 1500 | no data | 100 | Hsieh et al., 2011 |
| vB_Sau-RP15 | NP01 (environmental) | 0.001 | 180 | 1800 | no data | no data | Imklin et al., 2023 |
| VB_SauS_SH-St 15644 | 644 (clinical) | 0.01 | 300 | 720 | no data | 13 | Ji et al., 2020 |
| WV | no data (clinical) | 0.1 | no data | 4200 | 1600 | no data | Jiang et al., 2021 |
| ɸSA012 | SA003 (animal) | 0.002 | 1200 | 2520 | no data | 41 | Keita Osada et al., 2017 |
| vB_SauM-515A1 | SA515 (clinical) | 0.1 | 900 | 2400 | no data | 185 | Kornienko et al., 2020 |
| vB_SauP-436A | 515A1 (clinical) | 0.1 | 1500 | 3000 | no data | 94 | Kornienko et al., 2020 |
| Psa1 | MRSA CC398 (reference strain) | 0.001 | no data | 2400 | no data | 19 | Kraushaar et al., 2013 |
| Psa2 | MRSA CC398 (reference strain) | 0.001 | no data | 2400 | no data | 19 | Kraushaar et al., 2013 |
| Psa3 | MRSA CC398 (reference strain) | 0.001 | no data | 2400 | no data | 20 | Kraushaar et al., 2013 |
| Sb-1 | N50 (clinical) | no data | 300 | no data | no data | no data | Kvachadze et al., 2011 |
| KMSP1 | ATCC 33593 (reference strain) | 0.01 | 900 | 3000 | no data | 48 | Kwak et al., 2023 |
| MSA6 | ATCC 25923 (reference strain) | 0.0001 | 300 | 900 | 2100 | 23 | Kwiatek et al., 2012 |
| PALS2 | ATCC 33593 (reference strain) | 0.1 | 600 | 1800 | no data | 12 | Lee et al., 2021 |
| vB_SauM_VL10 | MRSA ATCC 43300 (reference strain) | 0.1 | 300 | 2100 | no data | 126 | Lerdsittikul et al., 2024 |
| SPW | 39 (animal) | 0.01 | 300 | 720 | 3000 | 44 | Li & Zhang, 2014 |
| APTC-SA-2 | ATCC 25923 (reference strain) | 0.1 | no data | 600 | no data | 30 | Liu et al., 2022 |
| APTC-SA-4 | ATCC 51650 (reference strain) | 0.1 | no data | 600 | no data | 11 | Liu et al., 2022 |
| ATPC-SA-12 | RN4220 (reference strain) | 0.1 | no data | 1200 | no data | 14 | Liu et al., 2022 |
| ATPC-SA-13 | RN4220 (reference strain) | 0.1 | no data | 1200 | no data | 18 | Liu et al., 2022 |
| StAP1 | XN61 (clinical) | 0.5 | 600 | 1200 | no data | 260 | Lu et al., 2023 |
| vB_SauM-A | 203 (reference strain) | 0.05 | 480 | 1500 | no data | 500 | Łubowska et al., 2019 |
| vB_SauM-C | 343 (reference strain) | 0.05 | 1080 | 1080 | no data | 40 | Łubowska et al., 2019 |
| vB_SauM-D | 342 (reference strain) | 0.05 | 900 | 2100 | no data | 80 | Łubowska et al., 2019 |
| SPB | no data (animal) | 1 | no data | 600 | 3600 | 5.46 | Ma et al., 2025 |
| phiIPLA-LAVI | RN4220 (clinical) | 0.1 | no data | 300 | 1200 | 22 | Magdalena et al., 2025 |
| ϕMR11 | SA37 (clinical) | 0.00001 | 60 | 1500 | no data | 100 | Matsuzaki et al., 2003 |
| UHP46 | S46 (animal) | 0.01 | no data | 1200 | no data | 27 | Najeeb et al., 2025 |
| SK311 | SA 812 (clinical) | no data | 600 | 1800 | no data | 70 | Pantůcek et al., 1998 |
| φ812 | SA 812 (clinical) | no data | 600 | 2700 | no data | 12 | Pantůcek et al., 1998 |
| pS2 | ATCC 1260 (reference strain) | 1 | no data | no data | no data | 68 | Paul et al., 2025 |
| pS3 | ATCC 1260 (reference strain) | 1 | no data | no data | no data | 220 | Paul et al., 2025 |
| pS1 | ATCC 1260 (reference strain) | 1 | no data | no data | no data | 280 | Paul et al., 2025 |
| ɸMR003 | RN4220 (clinical) | 0.01 | 600 | 1980 | no data | 35 | Peng et al., 2019 |
| Phage 2 | no data (animal) | 0.1 | no data | 1200 | 4200 | no data | Pereira et al., 2025 |
| SAP-26 | WS-26 (clinical) | 0.0001 | 540 | 900 | no data | 107 | Rahman et al., 2011 |
| vB_SauM_ME126 | 1S (animal) | 10 | no data | 900 | 900 | 140 | Rasha Mohamed Ali Gharieb et al., 2020 |
| vB_SauM_ME18 | 1S (animal) | 10 | no data | 900 | 900 | 114 | Rasha Mohamed Ali Gharieb et al., 2020 |
| φCIFT_MFB_MRSA12 | MRSA‑12 (clinical) | 0.1 | 600 | no data | no data | 107 | Raveendran et al., 2025 |
| φCIFT_MFB_MRSA28 | MRSA‑28 (clinical) | 0.1 | 600 | no data | no data | 82 | Raveendran et al., 2025 |
| no data | ATCC 6538 (reference strain) | 10 | 600 | 1800 | 2400 | 57 | Rezaei et al., 2022 |
| SaGU1 | 159-B1 (clinical) | 0.001 | no data | 2400 | no data | 129 | Shimamori et al., 2021 |
| ɸSA039 | RN4220 (clinical) | no data | no data | no data | no data | no data | Synnott et al., 2009 |
| TSP | MR10 (clinical) | 0.1 | 450 | 1200 | no data | 104 | Tabassum et al., 2022 |
| ISP | ATCC 6538 (reference strain) | 10 | 1500 | 2400 | no data | no data | Vandersteegen et al., 2011 |
| Remus | PSPB (clinical) | 0.1 | 1500 | no data | no data | no data | Vandersteegen K, et al., 2013 |
| Romulus | PS47 (clinical) | 0.1 | 1500 | no data | no data | no data | Vandersteegen et al., 2013 |
| vB_SauS_SA2 | F2 (animal) | 0.1 | no data | 600 | no data | 293 | Wang et al., 2019 |
| SLPW | ATCC 25923 (reference strain) | 0.1 | no data | 600 | 7200 | 95.3 | Wang et al., 2016 |
| SapYZU11 | ATCC 29213 (reference strain) | 0.1 | no data | 1200 | no data | 152 | Wen-Yuan Zhou et al., 2023 |
| SapYZU15 | ATCC 29213 (reference strain) | 0.1 | no data | 600 | no data | 322 | Wen et al., 2023 |
| SapYZU01 | no data (no data) | 0.1 | no data | 1200 | no data | 40.26 | Wen et al., 2023 |
| SapYZU02 | no data (no data) | 0.1 | no data | 1200 | no data | 33.58 | Wen et al., 2023 |
| SapYZU03 | no data (no data) | 0.1 | no data | 900 | no data | 80.8 | Wen et al., 2023 |
| JPL-50 | rf50 (animal) | 0.01 | no data | 1200 | 4800 | 25 | Xiao et al., 2024 |
| vB_SauP_L1 | MRSA01 (clinical) | 0.1 | no data | 1800 | no data | 1670 | Yanmei et al., 2024 |
| vB_SaRS_FS19–1 | JP19 (food) | 0.01 | no data | 1200 | no data | 700 | Yao et al., 2025 |
| SP5 | ATCC 29213 (reference strain) | no data | 300 | no data | no data | no data | Yoon et al., 2013 |
| SP6 | ATCC 29213 (reference strain) | no data | 300 | no data | no data | no data | Yoon et al., 2013 |
| PK0S2-PH | no data (clinical) | 0.0001 | 1200 | 1800 | 1200 | no data | Zafar et al., 2024 |
| phage 3 | no data (clinical) | 0.0001 | 1800 | 1800 | 1200 | no data | Zafar et al., 2025 |
| PSK | MRSA SK1 (clinical) | 0.01 | 300 | 1200 | no data | 123 | Zanaty et al., 2025 |
| vB_StaM_SA1 | JTB1-3 (environmental) | 0.1 | 120 | 3600 | 4200 | 135 | Zhang et al., 2022 |
| vB_SauM_JS25 | ATCC 6538 (reference strain) | 0.2 | 300 | 1200 | no data | 21 | Zhang et al., 2015 |
| vB_SauS_IMEP5 | P5 (animal) | 0.001 | no data | 1800 | no data | 272 | Zhang et al., 2017 |

**Supplementary Table S6** – Collected data regarding to phages infecting *S. aureus* in terms of presence of ‘halo’ effect, type of phage morphology, phage gene accesion number.

| **Phage designation** | **Host strain (source of isolation, if given)** | **Presence of 'halo' effect** | **Type of phage morphology** | **Phage gene accesion number** | **Reference** |
| --- | --- | --- | --- | --- | --- |
| DRA88 | MSSA H476 (clinical) | no data | myovirus | KJ888149 | Alves et al., 2014 |
| K | WBG8343 (clinical) | no data | myovirus | NC_005880 | Alves et al., 2014 |
| vB_SauM_LM12 | 2093367 (clinical) | yes | no data | MG721208.1 | Barros et al., 2019 |
| MSa | A170 (clinical) | no data | no data | no data | Capparelli et al., 2007 |
| SA97 | RN4220 (clinical) | no data | siphovirus | KJ716334 | Chang et al., 2015 |
| OPT-SA02 | SAU18 (no data) | no | myovirus | PQ046923 | Cho et al., 2025 |
| CapO46 | O46 (animal) | yes | podovirus | PV007823 | Cunha et al., 2025 |
| UPMK_1 | MRSA t127/4 (no data) | yes | myovirus | MG543995 | Dakheel et al., 2019 |
| UPMK_2 | MRSA t223/20 (no data) | yes | podovirus | MG564297 | Dakheel et al., 2019 |
| vB_SauM-UFV_DC4 | MRSA 3059 (animal) | yes | jumbo | MZ779063 | da Silva et al., 2023 |
| SA46-CTH2 | SA46 (clinical) | yes | podovirus | MK764384 | Duc et al., 2020 |
| LH1 | ST352 (no data) | no | siphovirus | JX174275 | El Haddad et al., 2013 |
| LH1-MUT | ST352 (no data | no | siphovirus | no data | El Haddad et al., 2013 |
| MRSA phage | no data (envrionmental) | no data | podovirus | no data | Elsayed et al., 2024 |
| vB_SauP_ASUmrsa123 | mrsa123 (clinical) | no | podovirus | OR259390 | El-Tawab et al., 2024 |
| Huma | ATCC 29213 (reference strain) | yes | podovirus | OQ302592 | Fatemeh Sharifi et al., 2024 |
| Simurgh | ATCC 29213 (reference strain) | yes | podovirus | OQ302593 | Fatemeh Sharifi et al., 2024 |
| LSA2308 | ATCC 25923 (reference strain) | no data | myovirus | MW363798 | Fei Ma et al., 2021 |
| LSA2366 | SA027 (reference strain) | no data | podovirus | MW363799 | Fei Ma et al., 2021 |
| SAJK-IND | MTCC (reference strain) | no | myovirus | MG010123 | Ganaie et al., 2018 |
| MSP | MTCC (reference strain) | no | podovirus | no data | Ganaie et al., 2018 |
| vB_SauS-phiIPLA35 | Sa9 (clinical) | no | siphovirus | EU861004 | García et al., 2009 |
| vB_SauS-phiIPLA88 | Sa9 (clinical) | no | siphovirus | EU861005 | García et al., 2009 |
| vB_SauP_phiAGO1.3 | phiAGO1.3 (clinical) | no data | podovirus | MG766218 | Głowacka-Rutkowska et al., 2019 |
| phiIPLA-RODI | IPLA16 (einvironmental) | yes | myovirus | KP027446 | Gutiérrez et al., 2015 |
| SAH-1 | N315 (animal) | no | myovirus | no data | Han et al., 2013 |
| Stau2 | S23 (clinical) | no | myovirus | KP8813321.1 | Hsieh et al., 2011 |
| vB_Sau-RP15 | NP01 (environmental) | no | siphovirus | MZ643272 | Imklin et al., 2023 |
| VB_SauS_SH-St 15644 | 644 (clinical) | no | siphovirus | MG770897 | Ji et al., 2020 |
| WV | no data (clinical) | no | myovirus | MT787017 | Jiang et al., 2021 |
| ɸSA012 | SA003 (animal) | no | myovirus | AB903967 | Keita Osada et al., 2017 |
| vB_SauM-515A1 | SA515 (clinical) | no data | myovirus | MN047438.1 | Kornienko et al., 2020 |
| vB_SauP-436A | 515A1 (clinical) | no data | podovirus | MN150710.1 | Kornienko et al., 2020 |
| Psa1 | MRSA CC398 (reference strain) | no | podovirus | no data | Kraushaar et al., 2013 |
| Psa2 | MRSA CC398 (reference strain) | no | podovirus | no data | Kraushaar et al., 2013 |
| Psa3 | MRSA CC398 (reference strain) | no | podovirus | HF937074 | Kraushaar et al., 2013 |
| Sb-1 | N50 (clinical) | no | myovirus | HQ163896 | Kvachadze et al., 2011 |
| KMSP1 | ATCC 33593 (reference strain) | no data | myovirus | ON153212.1 | Kwak et al., 2023 |
| MSA6 | ATCC 25923 (reference strain) | no data | myovirus | NC_047726.1 | Kwiatek et al., 2012 |
| PALS2 | ATCC 33593 (reference strain) | no data | jumbo | MN091626 | Lee et al., 2021 |
| vB_SauM_VL10 | MRSA ATCC 43300 (reference strain) | no | myovirus | OP940114 | Lerdsittikul et al., 2024 |
| SPW | 39 (animal) | no | myovirus | no data | Li & Zhang, 2014 |
| APTC-SA-2 | ATCC 25923 (reference strain) | no data | myovirus | OL960567 | Liu et al., 2022 |
| APTC-SA-4 | ATCC 51650 (reference strain) | no data | myovirus | OL960568 | Liu et al., 2022 |
| ATPC-SA-12 | RN4220 (reference strain) | no data | myovirus | OL960569 | Liu et al., 2022 |
| ATPC-SA-13 | RN4220 (reference strain) | no data | myovirus | OL960570 | Liu et al., 2022 |
| StAP1 | XN61 (clinical) | no | myovirus | OQ025229 | Lu et al., 2023 |
| vB_SauM-A | 203 (reference strain) | no data | myovirus | MN539738 | Łubowska et al., 2019 |
| vB_SauM-C | 343 (reference strain) | no data | myovirus | MN539737 | Łubowska et al., 2019 |
| vB_SauM-D | 342 (reference strain) | no data | myovirus | MN539736 | Łubowska et al., 2019 |
| SPB | no data (animal) | no | myovirus | PQ777470 | Ma et al., 2025 |
| phiIPLA-LAVI | RN4220 (clinical) | yes | siphovirus | PV474729 | Magdalena et al., 2025 |
| ϕMR11 | SA37 (clinical) | no | siphovirus | NC_010147.1 | Matsuzaki et al., 2003 |
| UHP46 | S46 (animal) | no | siphovirus | PP995776 | Najeeb et al., 2025 |
| SK311 | SA 812 (clinical) | no | myovirus | no data | Pantůcek et al., 1998 |
| φ812 | SA 812 (clinical) | no | myovirus | EF136589.1 | Pantůcek et al., 1998 |
| pS2 | ATCC 1260 (reference strain) | no | no data | no data | Paul et al., 2025 |
| pS3 | ATCC 1260 (reference strain) | no | no data | no data | Paul et al., 2025 |
| pS1 | ATCC 1260 (reference strain) | yes | no data | no data | Paul et al., 2025 |
| ɸMR003 | RN4220 (clinical) | no data | myovirus | AP019522 | Peng et al., 2019 |
| Phage 2 | no data (animal) | no data | siphovirus | no data | Pereira et al., 2025 |
| SAP-26 | WS-26 (clinical) | no data | siphovirus | KCTC11665BP | Rahman et al., 2011 |
| vB_SauM_ME126 | 1S (animal) | no | myovirus | no data | Rasha Mohamed Ali Gharieb et al., 2020 |
| vB_SauM_ME18 | 1S (animal) | no | myovirus | no data | Rasha Mohamed Ali Gharieb et al., 2020 |
| φCIFT_MFB_MRSA32 | MRSA32 (clinical) | no | myovirus | no data | Raveendran et al., 2025 |
| φCIFT_MFB_MRSA12 | MRSA‑12 (clinical) | no | myovirus | no data | Raveendran et al., 2025 |
| φCIFT_MFB_MRSA28 | MRSA‑28 (clinical) | no | myovirus | no data | Raveendran et al., 2025 |
| no data | ATCC 6538 (reference strain) | no data | cystovirus | no data | Rezaei et al., 2022 |
| SaGU1 | 159-B1 (clinical) | no data | myovirus | LC574321 | Shimamori et al., 2021 |
| ɸSA039 | RN4220 (clinical) | no data | myovirus | AP018375 | Synnott et al., 2009 |
| TSP | MR10 (clinical) | no | podovirus | MW286254 | Tabassum et al., 2022 |
| ISP | ATCC 6538 (reference strain) | no data | myovirus | FR852584 | Vandersteegen et al., 2011 |
| Remus | PSPB (clinical) | no | myovirus | JX846613 | Vandersteegen K, et al., 2013 |
| Romulus | PS47 (clinical) | no | myovirus | JX846613 | Vandersteegen et al., 2013 |
| vB_SauS_SA2 | F2 (animal) | no | siphovirus | MH356730 | Wang et al., 2019 |
| SLPW | ATCC 25923 (reference strain) | no | podovirus | KU992911 | Wang et al., 2016 |
| SapYZU11 | ATCC 29213 (reference strain) | no data | podovirus | MW864250 | Wen-Yuan Zhou et al., 2023 |
| SapYZU15 | ATCC 29213 (reference strain) | no data | myovirus | MW864252 | Wen et al., 2023 |
| SapYZU01 | no data (no data) | no data | no data | no data | Wen et al., 2023 |
| SapYZU02 | no data (no data) | no data | no data | no data | Wen et al., 2023 |
| SapYZU03 | no data (no data) | no data | no data | no data | Wen et al., 2023 |
| JPL-50 | rf50 (animal) | no | siphovirus | MZ359091 | Xiao et al., 2024 |
| vB_SauP_L1 | MRSA01 (clinical) | no | podovirus | OR944503 | Yanmei et al., 2024 |
| vB_SaRS_FS19–1 | JP19 (food) | no | myovirus | no data | Yao et al., 2025 |
| SP5 | ATCC 29213 (reference strain) | no data | siphovirus | CP003194 | Yoon et al., 2013 |
| SP6 | ATCC 29213 (reference strain) | no data | siphovirus | CP003194 | Yoon et al., 2013 |
| PK0S2-PH | no data (clinical) | no | siphovirus | BankIt2877764 | Zafar et al., 2024 |
| phage 3 | no data (clinical) | no | siphovirus | no data | Zafar et al., 2025 |
| PSK | MRSA SK1 (clinical) | no | podovirus | PQ110032 | Zanaty et al., 2025 |
| vB_StaM_SA1 | JTB1-3 (environmental) | no | jumbo | MW218148.1 | Zhang et al., 2022 |
| vB_SauM_JS25 | ATCC 6538 (reference strain) | no | myovirus | no data | Zhang et al., 2015 |
| vB_SauS_IMEP5 | P5 (animal) | no | siphovirus | KX156762 | Zhang et al., 2017 |

**Supplementary Table S7** – Collected data regarding to phages infecting *S. aureus* in terms of host range and polyvalence.

| **Phage designation** | **Host strain (source of isolation, if given)** | **Host range of the bacteriophage against *S. aureus* strains (vulnerable/tested)** | **Percentage of host range** | **Activity against other species** | **Tested other species (number of tested strains)** | **Reference** |
| --- | --- | --- | --- | --- | --- | --- |
| DRA88 | MSSA H476 (clinical) | 57/95 | 60% | no | *S. xylosus* (1);  *S. sciuri subsp. sciuri* (1);  *S. chromogenes* (1);  *S.* hyicus (1);  *S. arlettae* (1);  *S. vitulinus* (1);  *S. simulans* (1);  *S. epidermidis* (1) | Alves et al., 2014 |
| K | WBG8343 (clinical) | 61/95 | 64.21% | yes: *S. simulans* and *S. hyicus* | *S. xylosus* (1);  *S. sciuri subsp. sciuri* (1);  *S. chromogenes* (1);  *S. hyicus* (1);  *S. arlettae* (1);  *S. vitulinus* (1);  *S. simulans* (1);  *S. epidermidis* (1) | Alves et al., 2014 |
| vB_SauM_LM12 | 2093367 (clinical) | 5/6 | 91% | no data |  | Barros et al., 2019 |
| MSa | A170 (clinical) | 7/19 | 36.84% | no data |  | Capparelli et al., 2007 |
| SA97 | RN4220 (clinical) | 12/19 | 63.16% | no | *S. xylosis* (1);  *S. cohnii* (1);  *S. warneri* (1);  *S. intermedius* (1);  *E. faecalis* (1);  *B. cereus* (1);  *B. subtilis* (1);  *L. monocytogenes* (1);  *S. enterica* serovar Typhimurium (1);  *E. coli* (2);  *C. sakazakii* (1);  *P. aeruginosa* (1) | Chang et al., 2015 |
| OPT-SA02 | SAU18 (no data) | 106/107 | 99.07% | yes: *S. borealis*; *S. chromogenes*; *S. epidermidis*; *S. haemolyticus*; *S. saprophyticus*; *S. sciuri*; *S. simulans*; *S. xylosus* | *S. borealis* (3);  *S. chromogenes* (3);  *S. epidermidis* (3);  *S. haemolyticus* (3);  *S. saprophyticus* (3);  *S. sciuri* (3);  *S. simulans* (3);  *S. xylosus* (3);  *B. cereus* (1);  *S. iniae* (1);  *E. faecalis* (1);  *E. faecium* (1);  *S. uberis* (1);  *S. dysgalactiae* (1);  *S. enterica* serovar Typhimurium (1);  *S. enterica* serovar Enteritidis (1);  *E. coli* (1);  *K. pneumoniae* (1) | Cho et al., 2025 |
| CapO46 | O46 (animal) | 31/31 | 100% | no | *S. chromogenes* (1);  *S. epidermidis* (1);  *S. equorum* (1);  *S. gallinarum* (1);  *S. haemolyticus* (1);  *S. sciuri* (1);  *S. warneri* (1);  *S. xylosus* (1) | Cunha et al., 2025 |
| UPMK_1 | MRSA t127/4 (no data) | 20/25 | 80% | no data |  | Dakheel et al., 2019 |
| UPMK_2 | MRSA t223/20 (no data) | 23/25 | 92% | no data |  | Dakheel et al., 2019 |
| vB_SauM-UFV_DC4 | MRSA 3059 (animal) | 11/26 | 42.31% | no data |  | da Silva et al., 2023 |
| SA46-CTH2 | SA46 (clinical) | 55/58 | 94.83% | no data |  | Duc et al., 2020 |
| LH1 | ST352 (no data) | 4/14 | 28.57% | no data |  | El Haddad et al., 2013 |
| LH1-MUT | ST352 (no data | 7/14 | 50% | no data |  | El Haddad et al., 2013 |
| MRSA phage | no data (envrionmental) | no data | no data | no data |  | Elsayed et al., 2024 |
| vB_SauP_ASUmrsa123 | mrsa123 (clinical) | 12/25 | 48% | no data |  | El-Tawab et al., 2024 |
| Huma | ATCC 29213 (reference strain) | 3/7 | 42.86% | no | *S. hemolyticus* (2);  *S. epidermidis* (4);  *S. agnetis* (1); *Staphylococcus* sp. (6);  *S. hyicus* (1); *S.chromogenes* (1);  *S. enterica* serovar Typhimurium (1);  *Y. enterocolitica* (1);  *L. monocytogenes* (1);  *B. subtilis* (2);  *E.coli* (2) | Fatemeh Sharifi et al., 2024 |
| Simurgh | ATCC 29213 (reference strain) | 3/7 | 42.86% | no | *S.hemolyticus* (2);  *S. epidermidis* (4);  *S. agnetis* (1); *Staphylococcus* sp. (6);  *S. hyicus* (1);  *S. chromogenes* (1);  *S. enterica* serovar Typhimurium (1);  *Y. enterocolitica* (1);  *L. monocytogenes* (1);  *B. subtilis* (2);  *E. coli* (2) | Fatemeh Sharifi et al., 2024 |
| LSA2308 | ATCC 25923 (reference strain) | 19/29 | 65.52% | no | *S.* *enterica* serovar Enteritidis (1);  *S.* *enterica* serovar Typhimurium (1);  *E. coli* (1);  *L. monocytogenes* (1) | Fei Ma et al., 2021 |
| LSA2366 | SA027 (reference strain) | 19/29 | 65.52% | no | *S. enterica* serovar Enteritidis (1);  *S. enterica* serovar Typhimurium (1);  *E. coli* (1);  *L. monocytogenes* (1) | Fei Ma et al., 2021 |
| SAJK-IND | MTCC (reference strain) | 120/120 | 100% | no | *E. coli* (no data);  *S. agalactiae* (no data);  *K. pneumoniae* (no data);  *P. aeruginosa* (no data) | Ganaie et al., 2018 |
| MSP | MTCC (reference strain) | 48/120 | 40% | no | *E. coli* (no data);  *S. agalactiae* (no data);  *K. pneumoniae* (no data);  *P. aeruginosa* (no data) | Ganaie et al., 2018 |
| vB_SauS-phiIPLA35 | Sa9 (clinical) | no data | no data | no data |  | García et al., 2009 |
| vB_SauS-phiIPLA88 | Sa9 (clinical) | no data | no data | no data |  | García et al., 2009 |
| vB_SauP_phiAGO1.3 | phiAGO1.3 (clinical) | 55/75 | 73% | no data |  | Głowacka-Rutkowska et al., 2019 |
| phiIPLA-RODI | IPLA16 (einvironmental) | 38/47 | 80.85% | yes: all tested strains except 8 strains of *S. epidermidis* | *S. epidermidis* (10);  *S. haemolyticus* (2);  *S. hominis* (2);  *S. arlettae* (2);  *S. lugdunensis* (1);  *S. gallinarum* (1);  *S. kloosii* (1);  *S. pasteuri* (1);  *S. xylosus* (1);  *S. saprophyticus* (1);  *M. caseolyticus* (1) | Gutiérrez et al., 2015 |
| SAH-1 | N315 (animal) | 37/39 | 94.87% | no | *S. sciuri* (3);  *S. cohnii* (3);  *E. faecalis* (3) | Han et al., 2013 |
| Stau2 | S23 (clinical) | 164/205 | 80% | no data |  | Hsieh et al., 2011 |
| vB_Sau-RP15 | NP01 (environmental) | 12/12 | 100% | no | *L. monocytogenes* (1);  *S. enterica* serovar Enteritidis (1);  *S. enterica* serovar Typhimurium (1) | Imklin et al., 2023 |
| VB_SauS_SH-St 15644 | 644 (clinical) | 12/37 | 32.43% | no data |  | Ji et al., 2020 |
| WV | no data (clinical) | 4/12 | 33.33% | no data |  | Jiang et al., 2021 |
| ɸSA012 | SA003 (animal) | 21/28 | 75% | no data |  | Keita Osada et al., 2017 |
| vB_SauM-515A1 | SA515 (clinical) | 64/75 | 85.33% | no data |  | Kornienko et al., 2020 |
| vB_SauP-436A | 515A1 (clinical) | 51/75 | 68% | no data |  | Kornienko et al., 2020 |
| Psa1 | MRSA CC398 (reference strain) | 76/120 | 63.30% | no | *S. epidermidis* (1);  *B. subtilis* (1);  *B. cereus* (1);  *B. thuringiensis* (1);  *S. agalactiae* (1);  *E. coli* (1) | Kraushaar et al., 2013 |
| Psa2 | MRSA CC398 (reference strain) | 76/120 | 63% | no | *S. epidermidis* (1);  *B. subtilis* (1);  *B. cereus* (1);  *B. thuringiensis* (1);  *S. agalactiae* (1);  *E. coli* (1) | Kraushaar et al., 2013 |
| Psa3 | MRSA CC398 (reference strain) | 76/120 | 63.30% | no | *S. epidermidis* (1);  *B. subtilis* (1);  *B. cereus* (1);  *B. thuringiensis* (1);  *S. agalactiae* (1);  *E. coli* (1) | Kraushaar et al., 2013 |
| Sb-1 | N50 (clinical) | 25/27 | 92.59% | no data |  | Kvachadze et al., 2011 |
| KMSP1 | ATCC 33593 (reference strain) | 11/20 | 55% | yes: *S. warneri*, *S. cohnii* and *S. intermedius* | *S. warneri* (1);  *S. cohnii* (1);  *S. intermedius* (1);  *S. xylosus* (1);  *B. cereus* (1);  *B. subtilis* (1);  *L. monocytogenes* (1);  *E. coli* (2);  *C. sakazakii* (1);  *P. aeruginosa* (1) | Kwak et al., 2023 |
| MSA6 | ATCC 25923 (reference strain) | 26/27 | 96.30% | no | *S. saprophyticus* (2);  *S. epidermidis* (4) | Kwiatek et al., 2012 |
| PALS2 | ATCC 33593 (reference strain) | 14/14 | 100% | yes: *S. haemolyticus*, *S. epidermidis*, *S. hominis*, *S. warneri*, *S. xylosus*, *S. saprophyticus*, *S. captitis* and *S. cohnii* | *S. haemolyticus* (1);  *S. epidermidis* (3);  *S. hominis* (1);  *S. warneri* (1);  *S. xylosus* (1);  *S. saprophyticus* (1);  *S. captitis* (1);  *S. cohnii* (1);  *E. faecalis* (1);  *B. cereus* (1);  *B. subtilis* (1);  *L. monocytogenes* (1);  *S. enterica* serovar Typhimurium (1);  *E. coli* (1);  *C. sakazakii* (1);  *P. aeruginosa* (1) | Lee et al., 2021 |
| vB_SauM_VL10 | MRSA ATCC 43300 (reference strain) | 34/43 | 79.06% | no | *S. pseudintermedius* (5); Coagulase-negative *Staphylococci* (4) | Lerdsittikul et al., 2024 |
| SPW | 39 (animal) | 4/4 | 100% | yes: *E. coli* | *E. coli* (1) | Li & Zhang, 2014 |
| APTC-SA-2 | ATCC 25923 (reference strain) | 44/51 | 86% | yes: *S. epidermidis* | *S. epidermidis* (9);  *S. pneumoniae* (10);  *P. aeruginosa* (10);  *A. baumannii* (1);  *K. pneumoniae* (3);  *E. coli* (2);  *P. mirabilis* (1);  *E. faecalis* (1);  *E. faecium* (1) | Liu et al., 2022 |
| APTC-SA-4 | ATCC 51650 (reference strain) | 48/51 | 94% | yes: *S. epidermidis* | *S. epidermidis* (9);  *S. pneumoniae* (10);  *P. aeruginosa* (10);  *A. baumannii* (1);  *K. pneumoniae* (3);  *E. coli* (2);  *P. mirabilis* (1);  *E. faecalis* (1);  *E. faecium* (1) | Liu et al., 2022 |
| ATPC-SA-12 | RN4220 (reference strain) | no data | no data | yes: *S. epidermidis* | *S. epidermidis* (9);  *S. pneumoniae* (10);  *P. aeruginosa* (10);  *A. baumannii* (1);  *K. pneumoniae* (3);  *E. coli* (2);  *P. mirabilis* (1);  *E. faecalis* (1);  *E. faecium* (1) | Liu et al., 2022 |
| ATPC-SA-13 | RN4220 (reference strain) | no data | no data | yes: *S. epidermidis* | *S. epidermidis* (9);  *S. pneumoniae* (10);  *P. aeruginosa* (10);  *A. baumannii* (1);  *K. pneumoniae* (3);  *E. coli* (2);  *P. mirabilis* (1);  *E. faecalis* (1);  *E. faecium* (1) | Liu et al., 2022 |
| StAP1 | XN61 (clinical) | 75/162 | 46.30% | no data |  | Lu et al., 2023 |
| vB_SauM-A | 203 (reference strain) | 41/69 | 59.42% | yes: *S. epidermidis* | *S. epidermidis* (2);  *S. intermedius* (1);  *E. coli* (1);  *S. enterica* serovar Typhimurium (1);  *S. enterica* serovar Enteritidis (1);  *S. flexner*i (1);  *S. sonnei* (1);  *P. vulgaris* (1);  *P. mirabilis* (1);  *Y. enterocolitica* (1);  *P. aeruginosa* (1);  *E. faecalis* (1);  *L. lactis* (1);  *L. gasseri* (1);  *L. acidophilus* (1);  *B. cereus* (1);  *S. agalacticae* (1);  *L. monocytogenes* (1) | Łubowska et al., 2019 |
| vB_SauM-C | 343 (reference strain) | 58/69 | 84.06% | yes: *S. epidermidis* | *S. epidermidis* (2);  *S. intermedius* (1);  *E. coli* (1);  *S. enterica* serovar Typhimurium (1);  *S. enterica* serovar Enteritidis (1);  *S. flexner*i (1);  *S. sonnei* (1);  *P. vulgaris* (1);  *P. mirabilis* (1);  *Y. enterocolitica* (1);  *P. aeruginosa* (1);  *E. faecalis* (1);  *L. lactis* (1);  *L. gasseri* (1);  *L. acidophilus* (1);  *B. cereus* (1);  *S. agalacticae* (1);  *L. monocytogenes* (1) | Łubowska et al., 2019 |
| vB_SauM-D | 342 (reference strain) | 46/69 | 66.67% | yes: *S. epidermidis* | *S. epidermidis* (2);  *S. intermedius* (1);  *E. coli* (1);  *S. enterica* serovar Typhimurium (1);  *S. enterica* serovar Enteritidis (1);  *S. flexner*i (1);  *S. sonnei* (1);  *P. vulgaris* (1);  *P. mirabilis* (1);  *Y. enterocolitica* (1);  *P. aeruginosa* (1);  *E. faecalis* (1);  *L. lactis* (1);  *L. gasseri* (1);  *L. acidophilus* (1);  *B. cereus* (1);  *S. agalacticae* (1);  *L. monocytogenes* (1) | Łubowska et al., 2019 |
| SPB | no data (animal) | 46/47 | 97.87% | no data | no data | Ma et al., 2025 |
| phiIPLA-LAVI | RN4220 (clinical) | 36/43 | 83.72% | no | *S. epidermidis* (3);  *S. hominis* (2);  *S. haemolyticus* (1);  *S. lugdunensis* (1);  *S. arlettae* (1);  *S. kloosii* (1);  *S. pasteuri* (1);  *S. gallinarum* (1);  *S. saprophyticus* (1);  *M. caseolyticus* (1) | Magdalena et al., 2025 |
| ϕMR11 | SA37 (clinical) | 30/75 | 40% | no data |  | Matsuzaki et al., 2003 |
| UHP46 | S46 | 4/29 | 13.79% | no data | no data | Najeeb et al., 2025 |
| SK311 | SA 812 (clinical) | no data | no data | yes: *S. aureus subsp. aureus*; *S. aureus subsp. anaerobius*; *S. capitis subsp. capitis*; *S. capitis subsp. ureolyticus*; *S. carnosus*; *S. felis*; *S. haemolyticus*; *S. hominis*; *S. hyicus*; *S. intermedius*; *S. kloosii*; *S. lugdunensis*; *S. pasteuri*; *S. pulvereri*; *S. schleiferi subsp. schleiferi*; *S. warneri; S. simulans* | *S. arlettae* (1);  *S. aureus subsp. aureus* (1);  *S. aureus subsp. anaerobius* (1);  *S. auricularis* (2);  *"S. bovicus’’* (2);  *S. capitis subsp. capitis* (2);  *S. capitis subsp. ureolyticus* (2);  *S. caprae* (1);  *S. carnosus* (8);  *‘‘S. carouselicus’’* (2);  *S. caseolyticus* (1);  *S. chromogenes* (3);  *S. cohnii subsp. cohnii* (2);  *S. cohnii subsp. urealyticum* (3);  *S. delphini* (2);  *S. epidermidis* (6);  *S. equipercicus’* (2);  *S. equorum* (2);  *S. felis* (3);  *S. gallinarum* (2);  *S. haemolyticus* (3);  *S. hominis* (3);  *S. hyicus* (3);  *S. intermedius* (3);  *S. kloosii* (2);  *S. lentus* (2);  *S. lugdunensis* (2);  *S. lutrae* (1);  *S. muscae* (4);  *S. pasteuri* (2);  *S. piscifermentans* (5);  *S. pulvereri* (1);  *S. saprophyticus* (4);  *S. saprophyticus subsp. bovis* (1);  *S. schleiferi subsp. coagulans* (2);  *S. schleiferi subsp. schleiferi* (2);  *S. sciuri subsp. rodentium* (2);  *S. simulans* (3);  *S. vitulus* (1);  *S. warneri* (3);  *S. xylosus* (4) | Pantůcek et al., 1998 |
| φ812 | SA 812 (clinical) | 99/141 | 70% | yes: *S. aureus subsp. aureus*; *S. aureus subsp. anaerobius*; *S. capitis subsp. capitis*; *S. capitis subsp. ureolyticus*; *S. carnosus*; *S. epidermidis*; *S. felis*; *S. haemolyticus*; *S. hominis*; *S. hyicus*; *S. intermedius*; *S. kloosii*; *S. lugdunensis*; *S. pasteuri*; *S. pulvereri*; *S. saprophyticus*; *S. schleiferi subsp. schleiferi*; *S. warneri* | *S. arlettae* (1);  *S. aureus subsp. aureus* (1);  *S. aureus subsp. anaerobius* (1);  *S. auricularis* (2);  *"S. bovicus’’* (2);  *S. capitis subsp. capitis* (2);  *S. capitis subsp. ureolyticus* (2);  *S. caprae* (1);  *S. carnosus* (8);  *‘‘S. carouselicus’’* (2);  *S. caseolyticus* (1);  *S. chromogenes* (3);  *S. cohnii subsp. cohnii* (2);  *S. cohnii subsp. urealyticum* (3);  *S. delphini* (2);  *S. epidermidis* (6);  *S. equipercicus’* (2);  *S. equorum* (2);  *S. felis* (3);  *S. gallinarum* (2);  *S. haemolyticus* (3);  *S. hominis* (3);  *S. hyicus* (3);  *S. intermedius* (3);  *S. kloosii* (2);  *S. lentus* (2);  *S. lugdunensis* (2);  *S. lutrae* (1);  *S. muscae* (4);  *S. pasteuri* (2);  *S. piscifermentans* (5);  *S. pulvereri* (1);  *S. saprophyticus* (4);  *S. saprophyticus subsp. bovis* (1);  *S. schleiferi subsp. coagulans* (2);  *S. schleiferi subsp. schleiferi* (2);  *S. sciuri subsp. rodentium* (2);  *S. simulans* (3);  *S. vitulus* (1);  *S. warneri* (3);  *S. xylosus* (4) | Pantůcek et al., 1998 |
| pS2 | ATCC 1260 (reference strain) | 11/18 | 61.11% | no | *S. chromogenes* (1);  *S. haemolyticus* (1);  *E. coli* (1);  *S. uberis* (1);  *S. agalactia* (1);  *P. aeruginosa* (1);  *E. faecalis* (1);  *K. pneumoniae* (1) | Paul et al., 2025 |
| pS3 | ATCC 1260 (reference strain) | 13/18 | 72.22% | no | *S. chromogenes* (1);  *S. haemolyticus* (1);  *E. coli* (1);  *S. uberis* (1);  *S. agalactia* (1);  *P. aeruginosa* (1);  *E. faecalis* (1);  *K. pneumoniae* (1) | Paul et al., 2025 |
| pS1 | ATCC 1260 (reference strain) | 18/18 | 100% | yes: *S. chromogenes*; *S. haemolyticus* | *S. chromogenes* (1);  *S. haemolyticus* (1);  *E. coli* (1);  *S. uberis* (1);  *S. agalactia* (1);  *P. aeruginosa* (1);  *E. faecalis* (1);  *K. pneumoniae* (1) | Paul et al., 2025 |
| ɸMR003 | RN4220 (clinical) | 26/28 | 92.86% | no data |  | Peng et al., 2019 |
| Phage 2 | no data (animal) | 56/90 | 62.22% | no | *S. xylosus* (1);  *S. epidermidis* (1);  *S. haemolyticus* (1);  *S. saprophyticus* (1);  *S. hyicus* (1);  *S. intermedius* (1);  *S. enterica* serovar Typhimurium (1);  *S. agalactiae* (1);  *S. dysgalactiae subsp. equisimilis* (1);  *S. uberis* (1);  *L. monocytogenes* (1);  *E. coli* (1);  *C. sakazakii* (1) | Pereira et al., 2025 |
| SAP-26 | WS-26 (clinical) | 171/171 | 100.00% | no data |  | Rahman et al., 2011 |
| vB_SauM_ME126 | 1S (animal) | 4/40 | 10% | no data |  | Rasha Mohamed Ali Gharieb et al., 2020 |
| vB_SauM_ME18 | 1S (animal) | 4/40 | 10% | no data |  | Rasha Mohamed Ali Gharieb et al., 2020 |
| φCIFT_MFB_MRSA32 | MRSA32 (clinical) | 28/39 | 71.79% | no data | no data | Raveendran et al., 2025 |
| φCIFT_MFB_MRSA12 | MRSA‑12 (clinical) | 30/39 | 76.92% | no data | no data | Raveendran et al., 2025 |
| φCIFT_MFB_MRSA28 | MRSA‑28 (clinical) | 32/39 | 82.05% | no data | no data | Raveendran et al., 2025 |
| no data | ATCC 6538 (reference strain) | 16/20 | 80% | no data |  | Rezaei et al., 2022 |
| SaGU1 | 159-B1 (clinical) | 14/16 | 87.5% | no | *S. epidermidis* (7);  *L. innocua* (1);  *S. enterica* serovar Typhimurium (1);  *B. subtilis* (1);  *E. coli*;  *P. aeruginosa* (1) | Shimamori et al., 2021 |
| ɸSA039 | RN4220 (clinical) | 13/15 | 86.67% | no data |  | Synnott et al., 2009 |
| TSP | MR10 (clinical) | 27/40 | 67.5% | no | *S. epidermidis* (4);  *E. coli* (1);  *K. pneumoniae* (1);  *S. marcescens* (1);  *P. aeruginosa* (1);  *A. baumannii* (1);  *E. cloacae* (1) | Tabassum et al., 2022 |
| ISP | ATCC 6538 (reference strain) | 74/85 | 87.06% | no | *S. haemolyticus* (9) | Vandersteegen et al., 2011 |
| Remus | PSPB (clinical) | 61/90 | 68% | no | *S. haemolyticus* (9);  *S. epidermidis* (1) | Vandersteegen K, et al., 2013 |
| Romulus | PS47 (clinical) | 62/90 | 69% | no | *S. haemolyticus* (9);  *S. epidermidis* (1) | Vandersteegen et al., 2013 |
| vB_SauS_SA2 | F2 (animal) | 7/19 | 36.84% | yes: *S. saprophyticus* | *S. saprophyticus* (13);  *S. gallinarum* (5);  *S. cohnii* (4);  *S. sciuri* (3);  *S. lentus* (7);  *S. xylosus* (3) | Wang et al., 2019 |
| SLPW | ATCC 25923 (reference strain) | 36/40 | 90% | no | *S. epidermidis* (1);  *B. subtilis* (1);  *S. zooepidemicus* (1);  *E. coli* (1);  *S. suis* (4) | Wang et al., 2016 |
| SapYZU11 | ATCC 29213 (reference strain) | 35/53 | 66.04% | no | *S. epidermidis* (2) | Wen-Yuan Zhou et al., 2023 |
| SapYZU15 | ATCC 29213 (reference strain) | 48/53 | 90.57% | no | *S. epidermidis* (3);  *S. caprae* (1);  *E. coli* (1);  *E. hormaechei* (1); *Salmonella* (1);  *L. monocytogenes* (1);  *v. mimicus* (1) | Wen et al., 2023 |
| SapYZU01 | no data (no data) | 34/53 | 64.15% | no | *S. epidermidis* (3);  *S. caprae* (1);  *E. coli* (1);  *E. hormaechei* (1);  *Salmonella* (1);  *L. monocytogenes* (1);  *v. mimicus* (1) | Wen et al., 2023 |
| SapYZU02 | no data (no data) | 28/53 | 52.83% | no | *S. epidermidis* (3);  *S. caprae* (1);  *E. coli* (1);  *E. hormaechei* (1);  *Salmonella* (1);  *L. monocytogenes* (1);  *v. mimicus* (1) | Wen et al., 2023 |
| SapYZU03 | no data (no data) | 25/53 | 47.17% | no | *S. epidermidis* (3);  *S. caprae* (1);  *E. coli* (1);  *E. hormaechei* (1);  *Salmonella* (1);  *L. monocytogenes* (1);  *v. mimicus* (1) | Wen et al., 2023 |
| JPL-50 | rf50 (animal) | 168/200 | 84% | no data |  | Xiao et al., 2024 |
| vB_SauP_L1 | MRSA01 (clinical) | 18/35 | 51.40% | no data |  | Yanmei et al., 2024 |
| vB_SaRS_FS19–1 | JP19 (food) | 43/50 | 86% | no data | no data | Yao et al., 2025 |
| SP5 | ATCC 29213 (reference strain) | no data | no data | no data |  | Yoon et al., 2013 |
| SP6 | ATCC 29213 (reference strain) | no data | no data | no data |  | Yoon et al., 2013 |
| PK0S2-PH | no data (clinical) | 10/20 | 50% | no data |  | Zafar et al., 2024 |
| phage 3 | no data (clinical) | 10/20 | 50% | no data | no data | Zafar et al., 2025 |
| PSK | MRSA SK1 (clinical) | 4/10 | 40% | no data | no data | Zanaty et al., 2025 |
| vB_StaM_SA1 | JTB1-3 (environmental) | 6/12 | 50% | yes: *S. epidermidis* and *S. haemolyticus* | *S. epidermidis* (10);  *S. haemolyticus* (7);  *S. lentus* (1) | Zhang et al., 2022 |
| vB_SauM_JS25 | ATCC 6538 (reference strain) | 51/56 | 91.10% | no | *S. epidermidis* (4) | Zhang et al., 2015 |
| vB_SauS_IMEP5 | P5 (animal) | no data | no data | no data |  | Zhang et al., 2017 |

**Supplementary Table S8** – Bacterial strain species used in analyzed studies.

| **Bacterial strain species used in analyzed studies** |
| --- |
| *‘‘Staphylococcus carouselicus’’* |
| *"Stahylococcus bovicus’’* |
| *Acinetobacter baumannii* |
| *Bacillus cereus* |
| *Bacillus subtilis* |
| *Cronobacter sakazakii* |
| *Enterobacter cloacae* |
| *Enterobacter hormaechei* |
| *Enterococcus faecalis* |
| *Enterococcus faecium* |
| *Escherichia coli* |
| *Klebsiella pneumoniae* |
| *Lactobacillus acidophilus* |
| *Lactobacillus gasseri* |
| *Lactococcus lactis* |
| *Listeria monocytogenes* |
| *Macrococcus caseolyticus* |
| *Proteus mirabilis* |
| *Proteus vulgaris* |
| *Pseudomonas aeruginosa* |
| *Salmonella enterica* serovar Enteritidis |
| *Salmonella enterica* serovar Typhimurium |
| *Serratia marcescens* |
| *Shigella flexneri* |
| *Shigella sonnei* |
| *Staphylococcus agnetis* |
| *Staphylococcus arlettae* |
| *Staphylococcus aureus subsp. anaerobius* |
| *Staphylococcus aureus subsp. aureus* |
| *Staphylococcus auricularis* |
| *Staphylococcus borealis* |
| *Staphylococcus capitis subsp. capitis* |
| *Staphylococcus capitis subsp. ureolyticus* |
| *Staphylococcus caprae* |
| *Staphylococcus captitis* |
| *Staphylococcus carnosus* |
| *Staphylococcus caseolyticus* |
| *Staphylococcus chromogenes* |
| *Staphylococcus cohnii* |
| *Staphylococcus cohnii subsp. cohnii* |
| *Staphylococcus cohnii subsp. urealyticum* |
| *Staphylococcus delphini* |
| *Staphylococcus epidermidis* |
| *Staphylococcus equipercicus* |
| *Staphylococcus equorum* |
| *Staphylococcus felis* |
| *Staphylococcus gallinarum* |
| *Staphylococcus hemolyticus* |
| *Staphylococcus hominis* |
| *Staphylococcus hyicus* |
| *Staphylococcus iniae* |
| *Staphylococcus intermedius* |
| *Staphylococcus kloosii* |
| *Staphylococcus lentus* |
| *Staphylococcus lugdunensis* |
| *Staphylococcus lutrae* |
| *Staphylococcus muscae* |
| *Staphylococcus pasteuri* |
| *Staphylococcus piscifermentans* |
| *Staphylococcus pulvereri* |
| *Staphylococcus saprophyticus* |
| *Staphylococcus saprophyticus subsp. bovis* |
| *Staphylococcus schleiferi subsp. coagulans* |
| *Staphylococcus schleiferi subsp. schleiferi* |
| *Staphylococcus sciuri* |
| *Staphylococcus sciuri subsp. rodentium* |
| *Staphylococcus simulans* |
| *Staphylococcus* sp. |
| *Staphylococcus vitulinus* |
| *Staphylococcus vitulus* |
| *Staphylococcus warneri* |
| *Staphylococcus xylosis* |
| *Staphylococcus xylosus* |
| *Staphylococcus zooepidemicus* |
| *Streptococcus agalactiae* |
| *Streptococcus dysgalactiae subsp. equisimilis* |
| *Streptococcus pneumoniae* |
| *Streptococcus suis* |
| *Streptococcus uberis* |
| *Vibrio mimicus* |
| *Yersinia enterocolitica* |
