# Supplementary material for: A comprehensive analysis of the kinetics of infection of lytic bacteriophages specific to the ESKAPE and critical pathogens
Source: World J Microbiol Biotechnol. 2026 Feb 28;42(3):110. doi: 10.1007/s11274-025-04762-4 (PMC12950090; doi:10.1007/s11274-025-04762-4)
Supplement: Supplementary file 3 — Supplementary file3 (DOCX 133 KB) [file 11274_2025_4762_MOESM3_ESM.docx]

**Supplementary Table S9** – Collected data regarding to phages infecting *K. pneumoniae* in terms of cycle parameters.

| **Phage designation** | **Host strain (source of isolation, if given)** | **Results of the studied multiplicity of infection (MOI)** | **Adsorption time [s]** | **Latent period [s]** | **Lysis time [s]** | **Burst size [PFU/cell]** | **Reference** |
| --- | --- | --- | --- | --- | --- | --- | --- |
| vB_KpnP_kP17 | CCASU-2024-75 (clinical) | 0.1 | no data | 1200 | 2400 | 331 | Abdel-Razek et al., 2025 |
| NK20 | Kp20 (clinical) | 0.01 | 600 | 1800 | 3000 | 138 | Al-Madboly et al., 2023 |
| VTCCBPA43 | MTCC109 (reference strain) | 0.001 | no data | 4200 | 4200 | 172 | Anand et al., 2020 |
| BM7 | KP-7 (clinical) | 0.01 | no data | 600 | no data | 85 | Ananna et al., 2024 |
| BU9 | KP9 (clinical) | 0.01 | no data | 1500 | no data | 12 | Ananna et al., 2024 |
| A¥L | ATCC 700603 (reference strain) | 0.001 | 300 | 600 | no data | 166 | Asghar et al., 2022 |
| A¥M | ATCC 700603 (reference strain) | 0.001 | 300 | 1200 | no data | 15 | Asghar et al., 2022 |
| JKP2 | Kp-8890 (clinical) | 0.1 | no data | 2700 | no data | 70 | Asif et al., 2023 |
| KpnM | S32 (clinical) | 0.1 | 360 | 1080 | no data | 230 | Aslam et al., 2022 |
| UPM2146 | ATCC BAA-2146 (reference strain) | 0.001 | 240 | 1200 | 300 | 20 | Assafiri et al., 2021 |
| vB_kpnM_17-11 | Kp 17-11 (no data) | 0.001 | no data | 1800 | 3600 | 31.7 | Bai et al., 2022 |
| Kpn31 | CCCD-K001 (reference strain) | 0.001 | no data | 900 | no data | 9.12 | Balcão et al., 2022 |
| K2a | KP2 (clinical) | 0.01 | no data | 300 | no data | 116 | Baqer et al., 2022 |
| K2b | KP2 (clinical) | 0.01 | no data | 1200 | no data | 41 | Baqer et al., 2022 |
| K2w5 | KP2 (clinical) | 0.01 | no data | 1200 | no data | 354 | Baqer et al., 2022 |
| K2w6 | KP2 (clinical) | 0.1 | no data | 1500 | no data | 106 | Baqer et al., 2022 |
| Kp99 | KP9 (clinical) | 0.1 | no data | 1200 | no data | 214 | Baqer et al., 2022 |
| K9w5 | KP9 (clinical) | 0.001 | no data | 1800 | no data | 66 | Baqer et al., 2022 |
| K9w6 | KP9 (clinical) | 0.1 | no data | 600 | no data | 130 | Baqer et al., 2022 |
| K9coc | KP9 (clinical) | 0.01 | no data | 600 | no data | 210 | Baqer et al., 2022 |
| k3w7 | KP2 (clinical) | no data | no data | 1200 | no data | 220 | Baqer et al., 2023 |
| vB_KpnS-VAC35 | K3574 (clinical) | 0.01 | 300 | 600 | no data | 45.52 | Bleriot et al., 2023 |
| vB_KpnS-VAC36 | K3573 (clinical) | 0.01 | 120 | 480 | no data | 2.71 | Bleriot et al., 2023 |
| vB_KpnP_FBKp16 | K6310 (clinical) | 0.001 | no data | 600 | no data | 113 | Bonilla et al., 2021 |
| vB_KpnP_FBKp27 | L923 (clinical) | 0.001 | no data | 1500 | no data | 175 | Bonilla et al., 2021 |
| vB_KpnM_FBKp34 | K6453 (clinical) | 0.001 | no data | 1800 | no data | 12 | Bonilla et al., 2021 |
| vB_KpnM_FBKp24 | K6592 (clinical) | 0.001 | no data | 3000 | no data | 169 | Bonilla et al., 2021 |
| vB_KpnS_GH-K3 | K7 (clinical) | 0.0000001 | no data | 1200 | no data | 291 | Cai et al., 2019 |
| phage 1513 | KP 1513 (clinical) | 0.1 | no data | 1800 | 3000 | 264 | Cao et al., 2015 |
| SAKp02 | B3768 (clinical) | 0.1 | 300 | 1200 | no data | 202 | Chakraborty et al., 2024 |
| vB_KpP_HS106 | 106 (environmental) | 0.001 | 360 | 600 | no data | 183 | Chen et al., 2023 |
| P929 | Kp325 (clinical) | 0.1 | no data | 1500 | no data | 156 | Chen et al., 2022 |
| SS | B5055 (clinical) | no data | 600 | 1200 | 2400 | 98 | Chhibber et al., 2008 |
| vB_Kpn_F48 | 12C47 (clinical) | 1 | no data | 600 | 2400 | 72 | Ciacci et al., 2018 |
| KPAФ1 | ATCC 27736 (reference strain) | 0.01 | no data | 2100 | no data | 218 | Dandekar et al., 2025 |
| KP149Ф1 | 149 (clinical) | 0.01 | no data | 3000 | no data | 171 | Dandekar et al., 2025 |
| KP149Ф2 | 149 (clinical) | 0.01 | no data | 1800 | no data | 152 | Dandekar et al., 2025 |
| φBO1E | CG258 clade II (clinical) | 0.01 | no data | 600 | 1200 | 300 | D'Andrea et al., 2017 |
| MKP-1 | ATCC 618 (reference strain) | 0.1 | 300 | 2400 | 3000 | 260 | Das et al., 2024 |
| KP34 | 77 (clinical) | 0.005 | 300 | 900 | no data | 45 | Drulis-Kawa et al., 2011 |
| VB_KPM_KP1LMA | Scc 24 (no data) | 0.001 | 600 | 6000 | no data | 8 | Duarte et al., 2024 |
| TUN1 | K64 Kp (clnical) | 1 | no data | 600 | no data | 76 | Eckstein et al., 2021 |
| vB_KpnA_SCNJ1-Z | SCNJ1 (clinical) | 0.1 | no data | 540 | no data | 9 | Fang et al., 2023 |
| vB_KpnS_SCNJ1-C | SCNJ1 (clinical) | 0.1 | no data | 420 | no data | 7 | Fang et al., 2023 |
| vB_KpnM_SCNJ1-Y | SCNJ1 (clinical) | 0.1 | no data | 420 | no data | 15 | Fang et al., 2023 |
| P13 | ST11 (clinical) | 0.01 | 360 | 1200 | no data | 167 | Fang et al., 2022 |
| Trimon | Kp ATCC BAA-2146 (reference strain) | 0.001 | 300 | 900 | no data | 69 | Fausti et al., 2025 |
| Olmo | Kp ATCC BAA-2146 (reference strain) | 0.001 | no data | 900 | no data | 22 | Fausti et al., 2025 |
| Kilian | Kp ATCC BAA-2146 (reference strain) | 0.001 | 300 | 300 | no data | 474 | Fausti et al., 2025 |
| Jurek | Kp ATCC BAA-2146 (reference strain) | 0.001 | 300 | 600 | no data | 196 | Fausti et al., 2025 |
| ZCKP2 | KP/08 (clinical) | 0.1 | no data | 1500 | 600 | 650 | Fayez et al., 2023 |
| vB_KpnS_SXFY507 | SXFY507 (clinical) | 0.001 | no data | 1200 | no data | 246 | Feng et al., 2023 |
| pK3-24 | Kpn 3–24 (clinical) | 0.01 | 120 | 3000 | 4800 | 50 | Feng et al., 2024 |
| BUCT556A | 3128 (clinical) | 0.001 | no data | 2400 | no data | 91 | Feng et al., 2021 |
| vB_KpnM_IME346 | KP576 (clinical) | 0.01 | no data | 1200 | no data | 27 | Gao et al., 2022 |
| vB_KpnP_IME337 | 2390 (clinical) | 0.001 | no data | 600 | no data | 317 | Gao et al., 2020 |
| hvKpP3 | hvKpLS8 (clinical) | 10 | 600 | 1800 | no data | 94 | Geng et al., 2023 |
| KL-2146 | ATCC 13883 (reference strain) | 0.026 | no data | 1800 | no data | 142 | Gilcrease et al., 2023 |
| vB_KpnP_Dlv622 | Kp-9068 (clinical) | 0.01 | 390 | 1800 | no data | 66 | Gorodnichev et al., 2021 |
| vB_KpnM_Seu621 | Kp-9068 (clinical) | 0.01 | 390 | 1800 | no data | 85 | Gorodnichev et al., 2021 |
| KpS8 | KPi4275 (clinical) | 0.01 | 390 | 1800 | no data | 96 | Gorodnichev et al., 2021 |
| vB_KpnP_Klyazma | L2-1B (clinical) | 0.01 | 360 | 2100 | 1200 | 64 | Gorodnichev et al., 2023 |
| vB_KpnP_XY3 | Kpn32416 (clinical) | 0.1 | no data | 1200 | 2400 | 340 | Guo et al., 2025 |
| vB_KpnP_XY4 | Kpn31109 (clinical) | 0.1 | no data | 300 | 2100 | 126 | Guo et al., 2025 |
| BUCT631 | K7 (clinical) | 0.01 | 900 | 960 | no data | 303 | Han et al., 2023 |
| HHU1 | 1301 (clinical) | no data | 480 | 600 | 3000 | 134.17 | Han et al., 2025 |
| ΦSRD2021 | CRKP A1806 (clinical) | 0.01 | 600 | 600 | no data | 80 | Hao et al., 2021 |
| AM.K1 | ATCC 33495 (reference strain) | 0.01 | no data | 600 | no data | 148 | Hari et al., 2025 |
| AM.K2 | ATCC 33495 (reference strain) | 0.01 | no data | 600 | no data | 12 | Hari et al., 2025 |
| AM.K3 | ATCC 33495 (reference strain) | 0.01 | no data | 600 | no data | 28 | Hari et al., 2025 |
| AM.K4 | ATCC 33495 (reference strain) | 0.01 | no data | 900 | no data | 23 | Hari et al., 2025 |
| AM.K5 | ATCC 33495 (reference strain) | 0.01 | no data | 900 | no data | 24 | Hari et al., 2025 |
| AM.K6 | ATCC 33495 (reference strain) | 0.01 | no data | 900 | no data | 47 | Hari et al., 2025 |
| Pharr | ST258 (clinical) | 1 | no data | 960 | no data | no data | Hesse et al., 2021 |
| ϕKpNIH-2 | ST258 (clinical) | 1 | no data | 1440 | no data | no data | Hesse et al., 2021 |
| vB_KpnS_Kp13 | 533 (clinical) | 0.1 | no data | 1080 | 600 | 220 | Horváth et al., 2020 |
| Kpph1 | NUHL30457 (reference strain) | 0.01 | no data | 960 | 780 | 400 | Huang et al., 2025 |
| Kpph9 | NUHL30457 (reference strain) | 0.01 | no data | 660 | 900 | 25 | Huang et al., 2025 |
| φKp5130 | Kp5137 (clinical) | 0.1 | no data | 3900 | 1800 | 57 | Hu et al., 2023 |
| φKp9438 | Kp9310 (clinical) | 0.1 | no data | 3900 | 1500 | 31 | Hu et al., 2023 |
| ΦK2046 | FK2046 (clinical) | 0.001 | 1800 | 480 | 5400 | 5623 | Hu et al., 2025 |
| φNK5 | NK-5 (clinical) | no data | 300 | 600 | no data | 82 | Hung et al., 2011 |
| Z | M (clinical) | no data | no data | 1440 | no data | 320 | Jamal et al., 2015 |
| vB_KpnM_JYSS3 | 21AA2216 (clinical) | 0.001 | 360 | 300 | 2700 | 230 | Jiao et al., 2025 |
| K14-2 | KCTC 12385 (reference strain) | no data | no data | 1200 | 3000 | 32.9 | Kang et al., 2025 |
| vB_Klp_5 | KP33 (clinical) | 0.1 | 420 | 1500 | no data | 90 | Karumidze et al., 2013 |
| vB_Klp_1 | KP1 (clinical) | 0.1 | 420 | 900 | no data | 62.5 | Karumidze et al., 2013 |
| vB_Klp_3 | KP80 (clinical) | 0.1 | 420 | 600 | no data | 60 | Karumidze et al., 2013 |
| vB_Klp_4 | KP163 (clinical) | 0.1 | 420 | 900 | no data | 300 | Karumidze et al., 2013 |
| vB_Klp_6 | KP26 (clinical) | 0.1 | 420 | 900 | no data | 75 | Karumidze et al., 2013 |
| KpTRp1 | B5055 (reference strain) | 0.0001 | 1200 | 1800 | 9000 | 6 | Kazdaghli et al., 2025 |
| vB_KpnM_KP15 | no data (clinical) | 0.005 | 300 | 1500 | no data | 12.5 | Kęsik-Szeloch et al., 2013 |
| vB_KpnM_KP27 | no data (clinical) | 0.005 | 300 | 1500 | no data | 12.5 | Kęsik-Szeloch et al., 2013 |
| vB_KpnS_KP16 | no data (clinical) | 0.005 | 300 | 900 | no data | 55 | Kęsik-Szeloch et al., 2013 |
| vB_KpnS_KP36 | no data (clinical) | 0.005 | 300 | 900 | no data | 55 | Kęsik-Szeloch et al., 2013 |
| vB_KpnP_KP32 | no data (clinical) | 0.005 | 300 | 900 | no data | 55 | Kęsik-Szeloch et al., 2013 |
| vB_KpnP_KP34 | no data (clinical) | 0.005 | 300 | 900 | no data | 55 | Kęsik-Szeloch et al., 2013 |
| KP1 | K16-KPN-13-022 (no data) | 0.1 | no data | 1200 | no data | 197 | Kim et al., 2023 |
| KP12 | K16-KPN-13-022 (no data) | 0.1 | no data | 1200 | no data | 312 | Kim et al., 2023 |
| vB_KpnS_LmqsRe28-1 | 2 (animal) | 0.02 | no data | 1800 | no data | 1.34 | Köhne et al., 2025 |
| vB_KpnM_LmqsRe27-1 | 12 (animal) | 0.02 | no data | 1800 | no data | 47.69 | Köhne et al., 2025 |
| vB_KpnS_LmqsRe28-2 | 3 (animal) | 0.02 | no data | 1200 | no data | 0.13 | Köhne et al., 2025 |
| Kpn5 | B5055 (clinical) | no data | 300 | 1200 | no data | 130 | Kumari et al., 2010 |
| Kpn12 | B5055 (clinical) | no data | 420 | 1500 | no data | 140 | Kumari et al., 2010 |
| KPn13 | B5055 (clinical) | no data | 480 | 1500 | no data | 120 | Kumari et al., 2010 |
| Kpn17 | B5055 (clinical) | no data | 600 | 2100 | no data | 100 | Kumari et al., 2010 |
| Kpn22 | B5055 (clinical) | no data | 540 | 1800 | no data | 110 | Kumari et al., 2010 |
| CTF-1 | no data (clinical) | no data | no data | 2400 | no data | 92 | Kurt et al., 2025 |
| vB_KpnP_K3-ULINTkp1 | QAMH 130326/0185 (clinical) | 0.1 | 240 | 600 | no data | no data | Laforêt et al., 2022 |
| vB_KpnP_K3-ULINTkp2 | QAMH 130326/0185 (clinical) | 0.1 | 360 | 900 | no data | no data | Laforêt et al., 2022 |
| M198 | 198 (clinical) | 0.1 | 600 | 1200 | 6000 | 107.5 | Leshkasheli et al., 2025 |
| P01 | 135080 (clinical) | 0.1 | no data | 1800 | no data | 143 | Li et al., 2024 |
| P545 | KP4 (clinical) | 0.1 | no data | 1200 | no data | 82 | Li et al., 2020 |
| vB_KpnP_ZX1 | 111-2 (clinical) | 0.1 | 600 | 1800 | no data | 125 | Li et al., 2022 |
| P1011 | B16 (animal) | 0.1 | no data | 360 | no data | 41 | Li et al., 2024 |
| IME184 | 1558 (clinical) | 0.1 | 600 | 1800 | no data | no data | Li et al., 2022 |
| P509 | Kp30 (clinical) | 0.1 | no data | 300 | no data | 85 | Li et al., 2020 |
| 175008 | 135080 (clinical) | 0.01 | 2100 | 2250 | no data | 25 | Li et al., 2025 |
| phiA85 | A85 (clinical) | 0.001 | no data | 3000 | 7800 | no data | Li et al., 2025 |
| Henu2_3 | Kp1049 (clinical) | 0.001 | 360 | 600 | no data | 215 | Li et al., 2025 |
| CM_Kpn_HB132952 | KPHB132952 (clinical) | 1 | no data | 3000 | no data | no data | Liang et al., 2022 |
| CM_Kpn_HB143742 | KPHB143742 (clinical) | 1 | no data | 1800 | no data | no data | Liang et al., 2022 |
| vB_KpnM_KpVB3 | CRKP7 (no data) | 0.00001 | no data | 1200 | 3600 | 150 | Liu et al., 2024 |
| N22 | KP-ASM (no data) | 0.01 | no data | 1800 | 900 | 898000000 | Liu et al., 2025 |
| φKp-lyy15 | K1 (clinical) | 0.001 | no data | 600 | 1800 | 65 | Lu et al., 2015 |
| HZJ33 | KP703 (clinical) | 0.01 | no data | 600 | 3600 | 46500 | Lu et al., 2025 |
| vB_Kpn_B01 | 18 (animal) | 0.01 | no data | 2400 | no data | 40 | Luo et al., 2021 |
| myPSH1235 | no data (clinical) | 0.001 | no data | 2400 | no data | 120 | Manohar et al., 2019 |
| vB_KquU_φKuK6 | ATCC 700603 (reference strain) | 0.01 | no data | 1200 | no data | 435 | Miller et al., 2024 |
| vB_KshKPC-M | Kp100 (clinical) | 0.01 | 300 | 1200 | 1200 | 260 | Mohammadi et al., 2023 |
| vB_KpnM_KP1 | ATCC 13883 (reference strain) | 0.1 | no data | 600 | 1500 | 126 | Molina-López et al., 2025 |
| KP8 | CEMTC 356 (clinical) | 0.01 | no data | 900 | no data | 40 | Morozova et al., 2019 |
| PG14 | G14 (clinical) | 0.01 | 720 | 1200 | 1800 | 47 | Mulani et al., 2022 |
| vB_kpnP_KPYAP-1 | ST45 (clinical) | 0.001 | 240 | 1200 | 900 | 473 | Natarajan et al., 2024 |
| KA | KP1 (clinical) | 1 | no data | 2400 | no data | 80 | Nawaz et al., 2025 |
| IME268 | 1733 (clinical) | 0.001 | 600 | 1800 | no data | no data | Nazir et al., 2022 |
| LASTA | Ni9 (clinical) | 0.01 | 300 | 4800 | 3600 | 187 | Obradović et al., 2023 |
| SJM3 | Ni9 (clinical) | 0.01 | 300 | 4800 | 3000 | 155 | Obradović et al., 2023 |
| vB_KpnM-VAC66 | K3320 (clinical) | 0.01 | 720 | 300 | no data | 31.49 | Pacios et al., 2021 |
| vB_KpnM-VAC13 | K2534 (clinical) | 0.01 | 240 | 900 | no data | 380 | Pacios et al., 2021 |
| vB_KPnM_KPDP1 | yvu3 (no data) | no data | no data | 1200 | no data | 76 | Pallavali et al., 2021 |
| vB_KleS-HSE3 | 1025 (clinical) | 1 | no data | 1800 | no data | 277 | Peng et al., 2020 |
| vB_KleM_KB2 | 0915 (clinical) | 1 | no data | 600 | no data | 63 | Peng et al., 2023 |
| vB_Kp_XP4 | P4 (clinical) | 0.1 | no data | 600 | 2400 | 387 | Peng et al., 2025 |
| B1 | 52145 (clinical) | 0.01 | no data | 540 | 720 | 2200 | Pertics et al., 2021 |
| 731 | 52145 (clinical) | 0.01 | 600 | 600 | no data | 1000 | Pertics et al., 2023 |
| vB_KpS_GP-1 | EuSCAPE_IT395 (clinical) | 0.01 | no data | 3000 | 3000 | 150 | Ponsecchi et al., 2024 |
| vB_KpP_GP-2 | KP411 (clinical) | 0.01 | no data | 900 | no data | 9 | Ponsecchi et al., 2024 |
| vB_KpP_GP-4 | KP263 (clinical) | 0.01 | no data | 900 | no data | 53 | Ponsecchi et al., 2024 |
| vB_KpP_GP-5 | KP20-LU (clinical) | 0.01 | no data | 600 | no data | 87 | Ponsecchi et al., 2024 |
| BUCT610 | ST893 (clinical) | 0.01 | no data | 1800 | no data | no data | Pu et al., 2022 |
| BUCT541 | S-2007 (clinical) | 0.01 | no data | 1800 | 3000 | no data | Pu et al., 2022 |
| KP168 | UA168 (clinical) | 0.005 | 240 | 600 | 2400 | no data | Qi et al., 2020 |
| PSKP16 | BhvKp (clinical) | 0.1 | no data | 600 | 1500 | 180 | Rahimi et al., 2023 |
| P12L | ATCC 43816 (reference strain) | 0.001 | 600 | 900 | no data | 183 | Rollon et al., 2025 |
| KZag1 | K9 (clinical) | 0.01 | no data | 1200 | 2100 | 83 | Saqr et al., 2024 |
| vB_Kpn_F13 | KP5 (clinical) | 0.01 | no data | 1800 | no data | 56 | Senhaji-Kacha et al., 2024 |
| vB_Kpn_F14 | KP5 (clinical) | 0.01 | no data | 1200 | no data | 87 | Senhaji-Kacha et al., 2024 |
| kpssk3 | CRKP NY03 (clinical) | 10 | no data | 600 | no data | 200 | Shi et al., 2020 |
| vB_LZ 2044 | NTUH-K2044 (clinical) | 0.01 | no data | 1800 | 4200 | 396.7 | Shi et al., 2025 |
| ΦKpnBHU1 | KpnBHU09 (clinical) | no data | no data | 1800 | no data | 76 | Singh et al., 2024 |
| ΦKpnBHU2 | KpnBHU09 (clinical) | no data | no data | 4200 | no data | 43 | Singh et al., 2024 |
| ΦKpnBHU3 | KpnBHU09 (clinical) | no data | no data | 1500 | no data | 83 | Singh et al., 2024 |
| KPP-5 | CFS17 (food) | 100 | no data | 1500 | no data | 236 | Sofy et al., 2021 |
| vB_KpnM-Teh.1 | ATCC 10031 (reference strain) | 1 | 600 | 2760 | no data | 22 | Soleimani Sasani & Eftekhar, 2020 |
| TSK1 | ShA2 (environmental) | 0.6 | no data | 1800 | no data | 113 | Tabassum et al., 2018 |
| ZCKP1 | KP/01 (clinical) | 100 | no data | 1800 | 600 | 110 | Taha et al., 2018 |
| ΦFK1979 | FK1979 (clinical) | 0.0001 | no data | 300 | 900 | 292 | Tang et al., 2023 |
| PKp-V1 | ST258 (animal) | 0.01 | 570 | 1620 | no data | 197 | Tariq et al., 2024 |
| Henu1 | K2044 (clinical) | no data | no data | 1800 | no data | 95 | Teng et al., 2019 |
| K751 | ST258 (clinical) | no data | no data | 2100 | no data | 6 | Tisalema-Guanopatín et al., 2023 |
| T751 | ST258 (clinical) | no data | no data | 2700 | no data | 10 | Tisalema-Guanopatín et al., 2023 |
| T765 | ST258 (clinical) | no data | no data | 2100 | no data | 44 | Tisalema-Guanopatín et al., 2023 |
| PɸBw-Kp1 | Iaufa_lad2 (clinical) | no data | no data | 1200 | 4800 | no data | Torabi et al., 2021 |
| PɸBw-Kp2 | Iaufa_lad2 (clinical) | no data | no data | 3300 | 4800 | no data | Torabi et al., 2021 |
| PɸBw-Kp3 | Iaufa_lad2 (clinical) | no data | no data | 2400 | 5400 | no data | Torabi et al., 2021 |
| KPO1K2 | B5055 (clinical) | 0.001 |  | 900 | no data | 140 | Verma et al., 2009 |
| vB_KpnP_23 | JNKPN23 (clinical) | 0.1 | no data | 600 | 5400 | no data | Wang et al., 2024 |
| Kpp-9 | kp09 (clinical) | 0.1 | no data | 1500 | 6300 | 4800 | Wang et al., 2025 |
| KP1801 | ESBL-KP PW006 (clinical) | no data | 210 | 2100 | no data | 300 | Wintachai et al., 2020 |
| vKPPW7 | KPPW67 (clinical) | 0.1 | 420 | 1200 | 3000 | 106 | Wintachai et al., 2025 |
| Kpp95 | ATCC 23357 (reference strain) | 0.003 | no data | 1800 | no data | 100 | Wu et al., 2007 |
| KlebP_265 | CEMTC 5232 (reference strain) | 0.001 | 720 | 900 | no data | 26 | Yakubovskij et al., 2025 |
| vB_Kpn_HF0522 | KP1049 (clinical) | 0.01 | no data | 900 | 2700 | no data | Yan et al., 2025 |
| vB_Kpn_ZCKp20p | K20 (clinical) | 0.1 | no data | 1800 | no data | 100 | Zaki et al., 2023 |
| vB_KpnP_Bp5 | GXKP-J05 (animal) | 0.001 | no data | 300 | no data | 24 | Zhang et al., 2021 |
| vB_KpnP_IME279 | 530 (clinical) | 0.1 | no data | 1200 | no data | 140 | Zhang et al., 2020 |
| Kpn_PImp2 | IMP66 (clinical) | 1 | no data | 600 | no data | no data | Zhang et al., 2025 |
| Kpn_PImp3 | IMP66 (clinical) | 1 | no data | 180 | no data | no data | Zhang et al., 2025 |
| ZK1 | Wkp26 (clinical | 0.1 | no data | 120 | no data | 161 | Zhao et al., 2024 |
| p9676 | ST11 (clinical) | 0.001 | no data | 300 | no data | 40 | Zhao et al., 2025 |
| HZJ31 | KPZ2 (clinical) | 0.001 | 900 | 600 | 3600 | 374 | Zhu et al., 2025 |
| HS37 | KP 37 (clinical) | 0.01 | no data | 600 | 3000 | 93 | Zhu et al., 2026 |
| LAPAZ | KP01 (clinical) | 0.01 | 540 | 1800 | no data | 27 | Ziller et al., 2024 |
| vB_KpnS_FZ10 | Kl 327 (clinical) | 0.01 | no data | 1800 | 1800 | 81 | Zurabov et al., 2021 |
| vB_KpnS_FZ41 | Kl 325 (clinical) | 0.01 | no data | 2100 | 1500 | 120 | Zurabov et al., 2021 |
| vB_KpnP_FZ12 | Kl 315 (clinical) | 0.01 | no data | 1800 | 1500 | 83 | Zurabov et al., 2021 |
| vB_KpnM_FZ14 | Kl 263 (clinical) | 0.01 | no data | 1800 | 600 | 122 | Zurabov et al., 2021 |

**Supplementary Table S10** – Collected data regarding to phages infecting *K. pneumoniae* in terms of presence of ‘halo’ effect, type of phage morphology, phage gene accesion number.

| **Phage designation** | **Host strain** | **Presence of 'halo' effect** | **Type of phage morphology** | **Phage gene accesion number** | **Reference** |
| --- | --- | --- | --- | --- | --- |
| vB_KpnP_kP17 | CCASU-2024-75 (clinical) | yes | podovirus | PP096838 | Abdel-Razek et al., 2025 |
| NK20 | Kp20 (clinical) | yes | podovirus | OP013026 | Al-Madboly et al., 2023 |
| VTCCBPA43 | MTCC109 (reference strain) | no | myovirus | no data | Anand et al., 2020 |
| BM7 | KP-7 (clinical) | yes | marfavirus | OR145792.1 | Ananna et al., 2024 |
| BU9 | KP9 (clinical) | yes | marfavirus | OR145793.1 | Ananna et al., 2024 |
| A¥L | ATCC 700603 (reference strain) | yes | myovirus | OM103621 | Asghar et al., 2022 |
| A¥M | ATCC 700603 (reference strain) | yes | siphovirus | OM328157 | Asghar et al., 2022 |
| JKP2 | Kp-8890 (clinical) | yes | podovirus | ON165415.1 | Asif et al., 2023 |
| KpnM | S32 (clinical) | yes | myovirus | no data | Aslam et al., 2022 |
| UPM2146 | ATCC BAA-2146 (reference strain) | no | siphovirus | MN478483 | Assafiri et al., 2021 |
| vB_kpnM_17-11 | Kp 17-11 (no data) | yes | myovirus | MW239157 | Bai et al., 2022 |
| Kpn31 | CCCD-K001 (reference strain) | no | siphovirus | ON637170 | Balcão et al., 2022 |
| K2a | KP2 (clinical) | yes | podovirus | no data | Baqer et al., 2022 |
| K2b | KP2 (clinical) | yes | podovirus | no data | Baqer et al., 2022 |
| K2w5 | KP2 (clinical) | yes | podovirus | no data | Baqer et al., 2022 |
| K2w6 | KP2 (clinical) | yes | podovirus | no data | Baqer et al., 2022 |
| Kp99 | KP9 (clinical) | yes | podovirus | no data | Baqer et al., 2022 |
| K9w5 | KP9 (clinical) | yes | myovirus | no data | Baqer et al., 2022 |
| K9w6 | KP9 (clinical) | yes | podovirus | no data | Baqer et al., 2022 |
| K9coc | KP9 (clinical) | yes | podovirus | no data | Baqer et al., 2022 |
| k3w7 | KP2 (clinical) | no data | myovirus | no data | Baqer et al., 2023 |
| vB_KpnS-VAC35 | K3574 (clinical) | no data | Demerecvirus | MZ571828.1 | Bleriot et al., 2023 |
| vB_KpnS-VAC36 | K3573 (clinical) | no data | myovirus | MZ571829.1 | Bleriot et al., 2023 |
| vB_KpnP_FBKp16 | K6310 (clinical) | yes | podovirus | MW394389 | Bonilla et al., 2021 |
| vB_KpnP_FBKp27 | L923 (clinical) | yes | podovirus | MW394388 | Bonilla et al., 2021 |
| vB_KpnM_FBKp34 | K6453 (clinical) | no | myovirus | MW394390 | Bonilla et al., 2021 |
| vB_KpnM_FBKp24 | K6592 (clinical) | no | myovirus | MW394391 | Bonilla et al., 2021 |
| vB_KpnS_GH-K3 | K7 (clinical) | yes | siphovirus | MH844531 | Cai et al., 2019 |
| phage 1513 | KP 1513 (clinical) | yes | siphovirus | KP658157 | Cao et al., 2015 |
| SAKp02 | B3768 (clinical) | yes | myovirus | OR290970.1 | Chakraborty et al., 2024 |
| vB_KpP_HS106 | 106 (environmental) | yes | myovirus | NC_055919.1 | Chen et al., 2023 |
| P929 | Kp325 (clinical) | yes | podovirus | OK562429 | Chen et al., 2022 |
| SS | B5055 (clinical) | no data | podovirus | no data | Chhibber et al., 2008 |
| vB_Kpn_F48 | 12C47 (clinical) | no | myovirus | MG746602 | Ciacci et al., 2018 |
| KPAФ1 | ATCC 27736 (reference strain) | yes | myovirus | SRR27947663 | Dandekar et al., 2025 |
| KP149Ф1 | 149 (clinical) | yes | podovirus | SRR29283123 | Dandekar et al., 2025 |
| KP149Ф2 | 149 (clinical) | yes | podovirus | SRR27947661 | Dandekar et al., 2025 |
| φBO1E | CG258 clade II (clinical) | yes | podovirus | KM576124 | D'Andrea et al., 2017 |
| MKP-1 | ATCC 618 (reference strain) | no | siphovirus | PP627509 | Das et al., 2024 |
| KP34 | 77 (clinical) | yes | podovirus | GQ413938 | Drulis-Kawa et al., 2011 |
| VB_KPM_KP1LMA | Scc 24 (no data) | no | myovirus | PP002985 | Duarte et al., 2024 |
| TUN1 | K64 Kp (clnical) | yes | podovirus | HG994092.1 | Eckstein et al., 2021 |
| vB_KpnA_SCNJ1-Z | SCNJ1 (clinical) | yes | podovirus | OQ689084 | Fang et al., 2023 |
| vB_KpnS_SCNJ1-C | SCNJ1 (clinical) | yes | siphovirus | OQ718882 | Fang et al., 2023 |
| vB_KpnM_SCNJ1-Y | SCNJ1 (clinical) | yes | myovirus | OQ689083 | Fang et al., 2023 |
| P13 | ST11 (clinical) | yes | podovirus | MW042787 | Fang et al., 2022 |
| Trimon | Kp ATCC BAA-2146 (reference strain) | yes | siphovirus | PV340598.1 | Fausti et al., 2025 |
| Olmo | Kp ATCC BAA-2146 (reference strain) | yes | myovirus | PV425437.1 | Fausti et al., 2025 |
| Kilian | Kp ATCC BAA-2146 (reference strain) | yes | siphovirus | PV340597.1 | Fausti et al., 2025 |
| Jurek | Kp ATCC BAA-2146 (reference strain) | yes | siphovirus | PV360689.1 | Fausti et al., 2025 |
| ZCKP2 | KP/08 (clinical) | no | siphovirus | NC_071151 | Fayez et al., 2023 |
| vB_KpnS_SXFY507 | SXFY507 (clinical) | yes | siphovirus | OK019720 | Feng et al., 2023 |
| pK3-24 | Kpn 3–24 (clinical) | yes | podovirus | PP915726 | Feng et al., 2024 |
| BUCT556A | 3128 (clinical) | no | siphovirus | MZ172460 | Feng et al., 2021 |
| vB_KpnM_IME346 | KP576 (clinical) | no data | myovirus | MK685667 | Gao et al., 2022 |
| vB_KpnP_IME337 | 2390 (clinical) | no | podovirus | MN176573 | Gao et al., 2020 |
| hvKpP3 | hvKpLS8 (clinical) | yes | myovirus | MT559528 | Geng et al., 2023 |
| KL-2146 | ATCC 13883 (reference strain) | no data | siphovirus | MN379832 | Gilcrease et al., 2023 |
| vB_KpnP_Dlv622 | Kp-9068 (clinical) | yes | podovirus | MT939252 | Gorodnichev et al., 2021 |
| vB_KpnM_Seu621 | Kp-9068 (clinical) | no | myovirus | MT939253 | Gorodnichev et al., 2021 |
| KpS8 | KPi4275 (clinical) | yes | myovirus | MT178275 | Gorodnichev et al., 2021 |
| vB_KpnP_Klyazma | L2-1B (clinical) | yes | podovirus | OP125547.1 | Gorodnichev et al., 2023 |
| vB_KpnP_XY3 | Kpn32416 (clinical) | yes | siphovirus | PV296009 | Guo et al., 2025 |
| vB_KpnP_XY4 | Kpn31109 (clinical) | yes | siphovirus | PV296010 | Guo et al., 2025 |
| BUCT631 | K7 (clinical) | yes | podovirus | OP852425 | Han et al., 2023 |
| HHU1 | 1301 (clinical) | yes | myovirus | PQ438797.1 | Han et al., 2025 |
| ΦSRD2021 | CRKP A1806 (clinical) | no data | siphovirus | MZ208805 | Hao et al., 2021 |
| AM.K1 | ATCC 33495 (reference strain) | no | myovirus | no data | Hari et al., 2025 |
| AM.K2 | ATCC 33495 (reference strain) | no | myovirus | no data | Hari et al., 2025 |
| AM.K3 | ATCC 33495 (reference strain) | no | myovirus | no data | Hari et al., 2025 |
| AM.K4 | ATCC 33495 (reference strain) | no | myovirus | no data | Hari et al., 2025 |
| AM.K5 | ATCC 33495 (reference strain) | no | myovirus | no data | Hari et al., 2025 |
| AM.K6 | ATCC 33495 (reference strain) | no | siphovirus | no data | Hari et al., 2025 |
| Pharr | ST258 (clinical) | no data | podovirus | NC_048175.1 | Hesse et al., 2021 |
| ϕKpNIH-2 | ST258 (clinical) | no data | siphovirus | NC_049845.1 | Hesse et al., 2021 |
| vB_KpnS_Kp13 | 533 (clinical) | yes | siphovirus | MK170446 | Horváth et al., 2020 |
| Kpph1 | NUHL30457 (reference strain) | yes | siphovirus | OR983331 | Huang et al., 2025 |
| Kpph9 | NUHL30457 (reference strain) | yes | podovirus | OR983332 | Huang et al., 2025 |
| φKp9438 | Kp9310 (clinical) | yes | myovirus | no data | Hu et al., 2023 |
| ΦK2046 | FK2046 (clinical) | no | myovirus | PP736830 | Hu et al., 2025 |
| φNK5 | NK-5 (clinical) | yes | podovirus | no data | Hung et al., 2011 |
| Z | M (clinical) | yes | siphovirus | no data | Jamal et al., 2015 |
| vB_KpnM_JYSS3 | 21AA2216 (clinical) | yes | myovirus | PQ589211 | Jiao et al., 2025 |
| K14-2 | KCTC 12385 (reference strain) | no | myovirus | PP978606 | Kang et al., 2025 |
| vB_Klp_5 | KP33 (clinical) | yes | podovirus | no data | Karumidze et al., 2013 |
| vB_Klp_1 | KP1 (clinical) | yes | siphovirus | no data | Karumidze et al., 2013 |
| vB_Klp_3 | KP80 (clinical) | yes | siphovirus | no data | Karumidze et al., 2013 |
| vB_Klp_4 | KP163 (clinical) | yes | siphovirus | no data | Karumidze et al., 2013 |
| vB_Klp_6 | KP26 (clinical) | yes | podovirus | no data | Karumidze et al., 2013 |
| KpTRp1 | B5055 (reference strain) | yes | myovirus | PP954958 | Kazdaghli et al., 2025 |
| vB_KpnM_KP15 | no data (clinical) | no | myovirus | GU295964 | Kęsik-Szeloch et al., 2013 |
| vB_KpnM_KP27 | no data (clinical) | no | myovirus | HQ918180 | Kęsik-Szeloch et al., 2013 |
| vB_KpnS_KP16 | no data (clinical) | yes | siphovirus | no data | Kęsik-Szeloch et al., 2013 |
| vB_KpnS_KP36 | no data (clinical) | yes | siphovirus | JQ267364 | Kęsik-Szeloch et al., 2013 |
| vB_KpnP_KP32 | no data (clinical) | yes | podovirus | GQ413937 | Kęsik-Szeloch et al., 2013 |
| vB_KpnP_KP34 | no data (clinical) | yes | podovirus | GQ413938 | Kęsik-Szeloch et al., 2013 |
| KP1 | K16-KPN-13-022 (no data) | no | myovirus | MG751100.1 | Kim et al., 2023 |
| KP12 | K16-KPN-13-022 (no data) | no | myovirus | no data | Kim et al., 2023 |
| vB_KpnS_LmqsRe28-1 | 2 (animal) | yes | siphovirus | PV660645 | Köhne et al., 2025 |
| vB_KpnM_LmqsRe27-1 | 12 (animal) | yes | podovirus | PV660644 | Köhne et al., 2025 |
| vB_KpnS_LmqsRe28-2 | 3 (animal) | yes | siphovirus | PV759140 | Köhne et al., 2025 |
| Kpn5 | B5055 (clinical) | yes | podovirus | no data | Kumari et al., 2010 |
| Kpn12 | B5055 (clinical) | yes | podovirus | no data | Kumari et al., 2010 |
| KPn13 | B5055 (clinical) | yes | podovirus | OQ790081.1 | Kumari et al., 2010 |
| Kpn17 | B5055 (clinical) | yes | podovirus | OQ790080.1 | Kumari et al., 2010 |
| Kpn22 | B5055 (clinical) | yes | podovirus | no data | Kumari et al., 2010 |
| CTF-1 | no data (clinical) | no data | no data | PV550976 | Kurt et al., 2025 |
| vB_KpnP_K3-ULINTkp1 | QAMH 130326/0185 (clinical) | yes | podovirus | no data | Laforêt et al., 2022 |
| vB_KpnP_K3-ULINTkp2 | QAMH 130326/0185 (clinical) | yes | podovirus | no data | Laforêt et al., 2022 |
| M198 | 198 (clinical) | no data | myovirus | PQ182780.1 | Leshkasheli et al., 2025 |
| P01 | 135080 (clinical) | no | myovirus | OR387546 | Li et al., 2024 |
| P545 | KP4 (clinical) | no | myovirus | MN781108 | Li et al., 2020 |
| vB_KpnP_ZX1 | 111-2 (clinical) | yes | podovirus | MW722080 | Li et al., 2022 |
| P1011 | B16 (animal) | yes | siphovirus | OR492660 | Li et al., 2024 |
| IME184 | 1558 (clinical) | no | siphovirus | MZ398244.1 | Li et al., 2022 |
| P509 | Kp30 (clinical) | yes | podovirus | MT542697 | Li et al., 2020 |
| 175008 | 135080 (clinical) | no | myovirus | PQ360875 | Li et al., 2025 |
| phiA85 | A85 (clinical) | no | myovirus | PQ678658.1 | Li et al., 2025 |
| Henu2_3 | Kp1049 (clinical) | no | podovirus | PQ394119.1 | Li et al., 2025 |
| CM_Kpn_HB132952 | KPHB132952 (clinical) | yes | siphovirus | no data | Liang et al., 2022 |
| CM_Kpn_HB143742 | KPHB143742 (clinical) | no | podovirus | no data | Liang et al., 2022 |
| vB_KpnM_KpVB3 | CRKP7 (no data) | yes | myovirus | OR771714.1 | Liu et al., 2024 |
| N22 | KP-ASM (no data) | no | no data | no data | Liu et al., 2025 |
| φKp-lyy15 | K1 (clinical) | no data | siphovirus | no data | Lu et al., 2015 |
| HZJ33 | KP703 (clinical) | yes | podovirus | PQ793761 | Lu et al., 2025 |
| vB_Kpn_B01 | 18 (animal) | no | siphovirus | MT380195.1 | Luo et al., 2021 |
| myPSH1235 | no data (clinical) | no data | podovirus | MG972768 | Manohar et al., 2019 |
| vB_KquU_φKuK6 | ATCC 700603 (reference strain) | no | myovirus | PP874908 | Miller et al., 2024 |
| vB_KshKPC-M | Kp100 (clinical) | yes | siphovirus | ON489264.2 | Mohammadi et al., 2023 |
| vB_KpnM_KP1 | ATCC 13883 (reference strain) | no data | myovirus | PP782000.1 | Molina-López et al., 2025 |
| KP8 | CEMTC 356 (clinical) | no | podovirus | MG922974 | Morozova et al., 2019 |
| PG14 | G14 (clinical) | yes | siphovirus | OM964875 | Mulani et al., 2022 |
| vB_kpnP_KPYAP-1 | ST45 (clinical) | no | podovirus | OQ417518 | Natarajan et al., 2024 |
| KA | KP1 (clinical) | no | podovirus | OR809313 | Nawaz et al., 2025 |
| IME268 | 1733 (clinical) | no | siphovirus | MZ398242 | Nazir et al., 2022 |
| LASTA | Ni9 (clinical) | no | podovirus | NC_054965.1 | Obradović et al., 2023 |
| SJM3 | Ni9 (clinical) | no | podovirus | MT251348.1 | Obradović et al., 2023 |
| vB_KpnM-VAC66 | K3320 (clinical) | no | myovirus | MZ612130 | Pacios et al., 2021 |
| vB_KpnM-VAC13 | K2534 (clinical) | no data | myovirus | MZ322895 | Pacios et al., 2021 |
| vB_KPnM_KPDP1 | yvu3 (no data) | no data | myovirus | no data | Pallavali et al., 2021 |
| vB_KleS-HSE3 | 1025 (clinical) | no | siphovirus | MT075871 | Peng et al., 2020 |
| vB_KleM_KB2 | 0915 (clinical) | yes | myovirus | MT757392.1 | Peng et al., 2023 |
| vB_Kp_XP4 | P4 (clinical) | yes | siphovirus | PP663283 | Peng et al., 2025 |
| B1 | 52145 (clinical) | yes | siphovirus | MW672037 | Pertics et al., 2021 |
| 731 | 52145 (clinical) | no | siphovirus | OQ404738 | Pertics et al., 2023 |
| vB_KpS_GP-1 | EuSCAPE_IT395 (clinical) | yes | siphovirus | PP454752 | Ponsecchi et al., 2024 |
| vB_KpP_GP-2 | KP411 (clinical) | yes | podovirus | PP454753 | Ponsecchi et al., 2024 |
| vB_KpP_GP-4 | KP263 (clinical) | yes | podovirus | PP454754 | Ponsecchi et al., 2024 |
| vB_KpP_GP-5 | KP20-LU (clinical) | yes | podovirus | PP454755 | Ponsecchi et al., 2024 |
| BUCT610 | ST893 (clinical) | no | siphovirus | MZ318367.1 | Pu et al., 2022 |
| BUCT541 | S-2007 (clinical) | yes | siphovirus | MZ836210.1 | Pu et al., 2022 |
| KP168 | UA168 (clinical) | yes | podovirus | MN585130 | Qi et al., 2020 |
| PSKP16 | BhvKp (clinical) | yes | siphovirus | OW251746.1 | Rahimi et al., 2023 |
| P12L | ATCC 43816 (reference strain) | yes | podovirus | PQ287567 | Rollon et al., 2025 |
| KZag1 | K9 (clinical) | no data | myovirus | OR502445 | Saqr et al., 2024 |
| vB_Kpn_F13 | KP5 (clinical) | no data | siphovirus | PP341282 | Senhaji-Kacha et al., 2024 |
| vB_Kpn_F14 | KP5 (clinical) | no data | siphovirus | PP341283 | Senhaji-Kacha et al., 2024 |
| kpssk3 | CRKP NY03 (clinical) | yes | podovirus | MK134560 | Shi et al., 2020 |
| vB_LZ 2044 | NTUH-K2044 (clinical) | yes | no data | OP785155.1 | Shi et al., 2025 |
| ΦKpnBHU1 | KpnBHU09 (clinical) | yes | podovirus | OL979478 | Singh et al., 2024 |
| ΦKpnBHU2 | KpnBHU09 (clinical) | yes | podovirus | OL979479 | Singh et al., 2024 |
| ΦKpnBHU3 | KpnBHU09 (clinical) | yes | podovirus | OL976437 | Singh et al., 2024 |
| KPP-5 | CFS17 (food) | no | podovirus | MW600722 | Sofy et al., 2021 |
| vB_KpnM-Teh.1 | ATCC 10031 (reference strain) | no | myovirus | no data | Soleimani Sasani & Eftekhar, 2020 |
| TSK1 | ShA2 (environmental) | yes | siphovirus | MH688453 | Tabassum et al., 2018 |
| ZCKP1 | KP/01 (clinical) | no data | myovirus | MH252123 | Taha et al., 2018 |
| ΦFK1979 | FK1979 (clinical) | yes | myovirus | ON146449 | Tang et al., 2023 |
| PKp-V1 | ST258 (animal) | no | myovirus | no data | Tariq et al., 2024 |
| Henu1 | K2044 (clinical) | yes | podovirus | MK203841.1 | Teng et al., 2019 |
| K751 | ST258 (clinical) | yes | Taipeivirus | ON202820 | Tisalema-Guanopatín et al., 2023 |
| T751 | ST258 (clinical) | yes | Taipeivirus | ON323462 | Tisalema-Guanopatín et al., 2023 |
| T765 | ST258 (clinical) | yes | Taipeivirus | ON399185 | Tisalema-Guanopatín et al., 2023 |
| PɸBw-Kp1 | Iaufa_lad2 (clinical) | no | podovirus | no data | Torabi et al., 2021 |
| PɸBw-Kp2 | Iaufa_lad2 (clinical) | no | podovirus | no data | Torabi et al., 2021 |
| PɸBw-Kp3 | Iaufa_lad2 (clinical) | no | siphovirus | no data | Torabi et al., 2021 |
| KPO1K2 | B5055 (clinical) | yes | podovirus | no data | Verma et al., 2009 |
| vB_KpnP_23 | JNKPN23 (clinical) | yes | podovirus | PP542034.1 | Wang et al., 2024 |
| Kpp-9 | kp09 (clinical) | yes | podovirus | PQ285366 | Wang et al., 2025 |
| KP1801 | ESBL-KP PW006 (clinical) | yes | siphovirus | MN783016.1 | Wintachai et al., 2020 |
| vKPPW7 | KPPW67 (clinical) | yes | podovirus | PQ409239 | Wintachai et al., 2025 |
| Kpp95 | ATCC 23357 (reference strain) | no | myovirus | no data | Wu et al., 2007 |
| KlebP_265 | CEMTC 5232 (reference strain) | yes | siphovirus | PQ480913 | Yakubovskij et al., 2025 |
| vB_Kpn_HF0522 | KP1049 (clinical) | yes | podovirus | PP836776.1 | Yan et al., 2025 |
| vB_Kpn_ZCKp20p | K20 (clinical) | no | siphovirus | OP373729 | Zaki et al., 2023 |
| vB_KpnP_Bp5 | GXKP-J05 (animal) | no | podovirus | MN116494.1 | Zhang et al., 2021 |
| vB_KpnP_IME279 | 530 (clinical) | no data | podovirus | MF614100 | Zhang et al., 2020 |
| Kpn_PImp2 | IMP66 (clinical) | yes | siphovirus | PRJNA1220278 | Zhang et al., 2025 |
| Kpn_PImp3 | IMP66 (clinical) | no | siphovirus | PRJNA1220278 | Zhang et al., 2025 |
| ZK1 | Wkp26 (clinical | yes | podovirus | OK625527 | Zhao et al., 2024 |
| p9676 | ST11 (clinical) | no data | podovirus | PP646059 | Zhao et al., 2025 |
| HZJ31 | KPZ2 (clinical) | yes | podovirus | OR050820.1 | Zhu et al., 2025 |
| HS37 | KP 37 (clinical) | no | podovirus | OQ515479.1 | Zhu et al., 2026 |
| LAPAZ | KP01 (clinical) | yes | siphovirus | PP706156 | Ziller et al., 2024 |
| vB_KpnS_FZ10 | Kl 327 (clinical) | yes | siphovirus | MK521904 | Zurabov et al., 2021 |
| vB_KpnS_FZ41 | Kl 325 (clinical) | no | siphovirus | MK521907 | Zurabov et al., 2021 |
| vB_KpnP_FZ12 | Kl 315 (clinical) | yes | podovirus | MK521906 | Zurabov et al., 2021 |
| vB_KpnM_FZ14 | Kl 263 (clinical) | yes | myovirus | MK521905 | Zurabov et al., 2021 |

**Supplementary Table S11** – Collected data regarding to phages infecting *K. pneumoniae* in terms of host range and polyvalence.

| **Phage designation** | **K-type of the bacterial strains that phage can lyse** | **Host strain** | **Host range of the bacteriophage against *K. pneumoniae* strains (vulnerable/tested)** | **Percentage of host range** | **Activity against other species** | **Tested other species (number of tested strains)** | **Reference** |
| --- | --- | --- | --- | --- | --- | --- | --- |
| vB_KpnP_kP17 | no data | CCASU-2024-75 (clinical) | 7/40 | 17.5% | no | *E. coli* (4);  *P. aeruginosa* (3);  *S. aureus* (2);  *S. marcescens* (1);  *S. enterica* serovar Typhimurium (1);  *B. cereus* (1) | Abdel-Razek et al., 2025 |
| NK20 | no data | Kp20 (clinical) | 19/38 | 50% | no data |  | Al-Madboly et al., 2023 |
| VTCCBPA43 | no data | MTCC109 (reference strain) | 6/10 | 60% | no data |  | Anand et al., 2020 |
| BM7 | no data | KP-7 (clinical) | 2/30 | 6.67% | no data |  | Ananna et al., 2024 |
| BU9 | no data | KP9 (clinical) | 3/30 | 10% | no data |  | Ananna et al., 2024 |
| A¥L | no data | ATCC 700603 (reference strain) | 8/11 | 72.73% | no | *S. aureus* (1);  *S. enterica* serovar Typhimurium (1);  *A. baumannii* (1);  *P. aeruginosa* (1);  *E. coli* (1) | Asghar et al., 2022 |
| A¥M | no data | ATCC 700603 (reference strain) | 7/11 | 63.64% | no | *S. aureus* (1);  *S. enterica* serovar Typhimurium (1);  *A. baumannii* (1);  *P. aeruginosa* (1);  *E. coli* (1) | Asghar et al., 2022 |
| JKP2 | K17 | Kp-8890 (clinical) | 5/11 | 45.45% | no | *E. coli* (5); *Enterobacter* sp. (3);  *Pseudomonas* sp. (5);  *S. aureus* (5) | Asif et al., 2023 |
| KpnM | no data | S32 (clinical) | 53/67 | 79% | no data |  | Aslam et al., 2022 |
| UPM2146 | no data | ATCC BAA-2146 (reference strain) | 5/22 | 22.72% | no | *S. aureus* (1);  *E. coli* (2) | Assafiri et al., 2021 |
| vB_kpnM_17-11 | K19 | Kp 17-11 (no data) | 4/96 | 4.17% | no data |  | Bai et al., 2022 |
| Kpn31 | no data | CCCD-K001 (reference strain) | 12/27 | 44.44% | no | *E. coli* (1);  *S. enterica* (1);  *P. aeruginosa* (1); *P. mirabilis* (1);  *E. faecalis* (1);  *B. subtilis* (1);  *S. epidermidis* (1);  *S. aureus* (1);  *A. baumannii* (1) | Balcão et al., 2022 |
| K2a | no data | KP2 (clinical) | no data | no data | no data |  | Baqer et al., 2022 |
| K2b | no data | KP2 (clinical) | no data | no data | no data |  | Baqer et al., 2022 |
| K2w5 | no data | KP2 (clinical) | no data | no data | no data |  | Baqer et al., 2022 |
| K2w6 | no data | KP2 (clinical) | no data | no data | no data |  | Baqer et al., 2022 |
| Kp99 | no data | KP9 (clinical) | no data | no data | no data |  | Baqer et al., 2022 |
| K9w5 | no data | KP9 (clinical) | no data | no data | no data |  | Baqer et al., 2022 |
| K9w6 | K2; K57; K20 | KP9 (clinical) | no data | no data | no data |  | Baqer et al., 2022 |
| K9coc | no data | KP9 (clinical) | no data | no data | no data |  | Baqer et al., 2022 |
| k3w7 | no data | KP2 (clinical) | no data | no data | no data |  | Baqer et al., 2023 |
| vB_KpnS-VAC35 | K30 | K3574 (clinical) | 17/47 | 36.17% | no data |  | Bleriot et al., 2023 |
| vB_KpnS-VAC36 | K15;K51;K52 | K3573 (clinical) | 18/47 | 38.30% | no data |  | Bleriot et al., 2023 |
| vB_KpnP_FBKp16 | K110 | K6310 (clinical) | 1/7 | 14.29% | no | *S. aureus* (1);  *P. aeruginosa* (1);  *A. baumannii* (1);  *E. coli* (2) | Bonilla et al., 2021 |
| vB_KpnP_FBKp27 | K30 | L923 (clinical) | 1/7 | 14.29% | no | *S. aureus* (1);  *P. aeruginosa* (1);  *A. baumannii* (1);  *E. coli* (2) | Bonilla et al., 2021 |
| vB_KpnM_FBKp34 | K38 | K6453 (clinical) | 1/7 | 14.29% | no | *S. aureus* (1);  *P. aeruginosa* (1);  *A. baumannii* (1);  *E. coli* (2) | Bonilla et al., 2021 |
| vB_KpnM_FBKp24 | K64 | K6592 (clinical) | 3/7 | 42.86% | no | *S. aureus* (1);  *P. aeruginosa* (1);  *A. baumannii* (1);  *E. coli* (2) | Bonilla et al., 2021 |
| vB_KpnS_GH-K3 | no data | K7 (clinical) | 9/72 | 12.50% | no data |  | Cai et al., 2019 |
| phage 1513 | no data | KP 1513 (clinical) | 5/10 | 50% | no | *P. aeruginosa* (1);  *S. aureus* (1);  *E. coli* (1) | Cao et al., 2015 |
| SAKp02 | no data | B3768 (clinical) | 43/72 | 59.72% | no data |  | Chakraborty et al., 2024 |
| vB_KpP_HS106 | K2 | 106 (environmental) | 25/41 | 60.98% | no data |  | Chen et al., 2023 |
| P929 | K19 | Kp325 (clinical) | 21/84 | 25% | no data |  | Chen et al., 2022 |
| SS | no data | B5055 (clinical) | 7/20 | 35% | no data |  | Chhibber et al., 2008 |
| vB_Kpn_F48 | K17 | 12C47 (clinical) | 14/61 | 23% | no |  | Ciacci et al., 2018 |
| φBO1E | K154 | CG258 clade II (clinical) | 4/83 | 4.82% | no data |  | D'Andrea et al., 2017 |
| KPAФ1 | no data | ATCC 27736 (reference strain) | 21/87 | 24.13% | no data |  | Dandekar et al., 2025 |
| KP149Ф1 | no data | 149 (clinical) | 5/87 | 5.74% | no data |  | Dandekar et al., 2025 |
| KP149Ф2 | no data | 149 (clinical) | 4/87 | 4.59% | no data |  | Dandekar et al., 2025 |
| MKP-1 | no data | ATCC 618 (reference strain) | 3/3 | 100% | no | *E. coli* (1);  *P. aeruginosa* (1);  *S. enterica* serovar Typhi (1) | Das et al., 2024 |
| KP34 | no data | 77 (clinical) | 42/101 | 41.58% | no | *K. pneumoniae subsp. ozaenae* (2);  *K. pneumoniae subsp. rhinoscleromatis* (2);  *K. oxytoca* (72);  *E. cloacae* (50);  *E. agglomerans* (1);  *E. aerogenes* (3);  *C. (Enterobacter) sakazakii* (1);  *E. coli* (100) | Drulis-Kawa et al., 2011 |
| VB_KPM_KP1LMA | no data | Scc 24 (no data) | 1/6 | 16.67% | yes: *E. coli* | *E. coli* (36);  *C. freundii* (1);  *E. cloacae* (1);  *Providencia* sp. (1);  *S. enterica* serovar Enteriditis (5);  *S. enterica* serovar Typhimurium (2);  *S. flexneri* (1) | Duarte et al., 2024 |
| TUN1 | K64 | K64 Kp (clnical) | 57/82 | 69.51% | no data |  | Eckstein et al., 2021 |
| vB_KpnA_SCNJ1-Z | K54 | SCNJ1 (clinical) | 1/32 | 3.13% | no | *A. baumannii* (1);  *E. cloacae* (1);  *K. cryocrescens* (1);  *C. braakii* (1);  *R. ornithinolytica* (1);  *K. gyiorum* (1);  *M. morganii* (1);  *P. alcalifaciens* (1);  *E. coli* (6);  *Providencia* sp. (2);  *Citrobacter* sp. (1);  *K. variicola* (1) | Fang et al., 2023 |
| vB_KpnS_SCNJ1-C | K54 | SCNJ1 (clinical) | 1/32 | 3.13% | no | *A. baumannii* (1);  *E. cloacae* (1);  *K. cryocrescens* (1);  *C. braakii* (1);  *R. ornithinolytica* (1);  *K. gyiorum* (1);  *M. morganii* (1);  *P. alcalifaciens* (1);  *E. coli* (6);  *Providencia* sp. (2);  *Citrobacter* sp. (1);  *K. variicola* (1) | Fang et al., 2023 |
| vB_KpnM_SCNJ1-Y | K54 | SCNJ1 (clinical) | 1/32 | 3.13% | no | *A. baumannii* (1);  *E. cloacae* (1);  *K. cryocrescens* (1);  *C. braakii* (1);  *R. ornithinolytica* (1);  *K. gyiorum* (1);  *M. morganii* (1);  *P. alcalifaciens* (1);  *E. coli* (6);  *Providencia* sp. (2);  *Citrobacter* sp. (1);  *K. variicola* (1) | Fang et al., 2023 |
| P13 | K47 | ST11 (clinical) | 1/18 | 5.56% | no data |  | Fang et al., 2022 |
| Trimon | no data | Kp ATCC BAA-2146 (reference strain) | 4/89 | 4.49% | no data |  | Fausti et al., 2025 |
| Olmo | no data | Kp ATCC BAA-2146 (reference strain) | 5/89 | 5.62% | no data |  | Fausti et al., 2025 |
| Kilian | no data | Kp ATCC BAA-2146 (reference strain) | 4/89 | 4.49% | no data |  | Fausti et al., 2025 |
| Jurek | no data | Kp ATCC BAA-2146 (reference strain) | 3/89 | 3.37% | no data |  | Fausti et al., 2025 |
| ZCKP2 | no data | KP/08 (clinical) | 7/30 | 23.33% | no data |  | Fayez et al., 2023 |
| vB_KpnS_SXFY507 | no data | SXFY507 (clinical) | 23/27 | 85.19% | no data |  | Feng et al., 2023 |
| pK3-24 | no data | Kpn 3–24 (clinical) | 8/95 | 8.42% | no data |  | Feng et al., 2024 |
| BUCT556A | no data | 3128 (clinical) | 15/74 | 20.27% | no | *E. coli* (10) | Feng et al., 2021 |
| vB_KpnM_IME346 | K63 | KP576 (clinical) | 4/12 | 33.33% | no data |  | Gao et al., 2022 |
| vB_KpnP_IME337 | K2 | 2390 (clinical) | 1/30 | 3.33% | no data |  | Gao et al., 2020 |
| hvKpP3 | K2 | hvKpLS8 (clinical) | 8/12 | 66.67% | no data |  | Geng et al., 2023 |
| KL-2146 | no data | ATCC 13883 (reference strain) | no data | no data | no data |  | Gilcrease et al., 2023 |
| vB_KpnP_Dlv622 | K23 | Kp-9068 (clinical) | 4/83 | 4.82% | no data |  | Gorodnichev et al., 2021 |
| vB_KpnM_Seu621 | K23 | Kp-9068 (clinical) | 4/83 | 4.82% | no data |  | Gorodnichev et al., 2021 |
| KpS8 | K23 | KPi4275 (clinical) | 4/83 | 4.82% | no data |  | Gorodnichev et al., 2021 |
| vB_KpnP_Klyazma | K20 | L2-1B (clinical) | 11/180 | 6.11% | no data |  | Gorodnichev et al., 2023 |
| vB_KpnP_XY3 | no data | Kpn32416 (clinical) | 3/26 | 11.54% | no | *A. baumannii* (15);  *S. aureus* (25) | Guo et al., 2025 |
| vB_KpnP_XY4 | no data | Kpn31109 (clinical) | 3/26 | 11.54% | no | *A. baumannii* (15);  *S. aureus* (25) | Guo et al., 2025 |
| BUCT631 | K1 | K7 (clinical) | 7/30 | 23.33% | no data |  | Han et al., 2023 |
| HHU1 | K2 | 1301 (clinical) | 7/33 | 21.21% | no data |  | Han et al., 2025 |
| AM.K1 | no data | ATCC 33495 (reference strain) | 52/60 | 87% | no | *A. baumannii* (1);  *P. aeruginosa* (2);  *S. aureus* (1);  *K. oxytoca* (1);  *E. coli* (1) | Hari et al., 2025 |
| AM.K2 | no data | ATCC 33495 (reference strain) | 52/60 | 87% | no | *A. baumannii* (1);  *P. aeruginosa* (2);  *S. aureus* (1);  *K. oxytoca* (1);  *E. coli* (1) | Hari et al., 2025 |
| AM.K3 | no data | ATCC 33495 (reference strain) | 45/60 | 75% | no | *A. baumannii* (1);  *P. aeruginosa* (2);  *S. aureus* (1);  *K. oxytoca* (1);  *E. coli* (1) | Hari et al., 2025 |
| AM.K4 | no data | ATCC 33495 (reference strain) | 36/60 | 60% | no | *A. baumannii* (1);  *P. aeruginosa* (2);  *S. aureus* (1);  *K. oxytoca* (1);  *E. coli* (1) | Hari et al., 2025 |
| AM.K5 | no data | ATCC 33495 (reference strain) | 35/60 | 58% | no | *A. baumannii* (1);  *P. aeruginosa* (2);  *S. aureus* (1);  *K. oxytoca* (1);  *E. coli* (1) | Hari et al., 2025 |
| AM.K6 | no data | ATCC 33495 (reference strain) | 47/60 | 78% | no | *A. baumannii* (1);  *P. aeruginosa* (2);  *S. aureus* (1);  *K. oxytoca* (1);  *E. coli* (1) | Hari et al., 2025 |
| ΦSRD2021 | K47 | CRKP A1806 (clinical) | 2/47 | 4.26% | no data |  | Hao et al., 2021 |
| Pharr | no data | ST258 (clinical) | no data | no data | no data |  | Hesse et al., 2021 |
| ϕKpNIH-2 | no data | ST258 (clinical) | no data | no data | no data |  | Hesse et al., 2021 |
| vB_KpnS_Kp13 | K24 | 533 (clinical) | 40/89 | 44.94% | no data |  | Horváth et al., 2020 |
| Kpph1 | K2 | NUHL30457 (reference strain) | 2/15 | 13.33% | no data |  | Huang et al., 2025 |
| Kpph9 | K2 | NUHL30457 (reference strain) | 3/15 | 20% | no data |  | Huang et al., 2025 |
| φKp5130 | K2; K5; K24; K38; K62 | Kp5137 (clinical) | 6/22 | 27.27% | no data |  | Hu et al., 2023 |
| φKp9438 | K2; K12; K15; K19; K20; K23; K24; K30; K38; K47; K62; K64; K158 | Kp9310 (clinical) | 21/22 | 95.45% | no data |  | Hu et al., 2023 |
| ΦK2046 | no data | FK2046 (clinical) | no data | no data | no data |  | Hu et al., 2025 |
| φNK5 | no data | NK-5 (clinical) | no data | no data | no data |  | Hung et al., 2011 |
| Z | no data | M (clinical) | 4/16 | 25% | no | *E. coli* (6);  *P. aeruginosa* (7);  *S. aureus* (4);  *A. xylosoxidans* (1) | Jamal et al., 2015 |
| vB_KpnM_JYSS3 | K2 | 21AA2216 (clinical) | 1/96 | 1.04% | no data |  | Jiao et al., 2025 |
| K14-2 | no data | KCTC 12385 (reference strain) | 4/4 | 100% | yes: *K. pneumoniae subsp. ozaenae*; *K. alba*; *K. michiganensis*; *R. ornithinolytica*; *R. planticola*; *E. coli* | *K. pneumoniae subsp. ozaenae* (4);  *K. alba* (1);  *K. michiganensis* (3);  *R. ornithinolytica* (5);  *R. planticola* (1);  *E. coli* (1);  *A. baumannii* (1);  *P. aeruginosa* (1);  *S. aureus* (1) | Kang et al., 2025 |
| vB_Klp_5 | no data | KP33 (clinical) | no data | no data | no data |  | Karumidze et al., 2013 |
| vB_Klp_1 | no data | KP1 (clinical) | no data | no data | no data |  | Karumidze et al., 2013 |
| vB_Klp_3 | no data | KP80 (clinical) | no data | no data | no data |  | Karumidze et al., 2013 |
| vB_Klp_4 | no data | KP163 (clinical) | no data | no data | no data |  | Karumidze et al., 2013 |
| vB_Klp_6 | no data | KP26 (clinical) | no data | no data | no data |  | Karumidze et al., 2013 |
| KpTRp1 | K2 | B5055 (reference strain) | 1/5 | 20% | no | *K. oxytoca* (1);  *P. aeruginosa* (2);  *P. putida* (2);  *S. aureus* (2);  *S. xylosus* (1) | Kazdaghli et al., 2025 |
| vB_KpnM_KP15 | no data | no data (clinical) | no data | no data | no | *K. pneumoniae subsp. ozaenae* (2);  *K. pneumoniae subsp. rhinoscleromatis* (2);  *K. oxytoca* (48);  *E. aerogenes* (3);  *E. cloacae* (50);  *E. agglomerans* (1);  *C. sakazakii* (1);  *E. coli* (50) | Kęsik-Szeloch et al., 2013 |
| vB_KpnM_KP27 | no data | no data (clinical) | no data | no data | no | *K. pneumoniae subsp. ozaenae* (2);  *K. pneumoniae subsp. rhinoscleromatis* (2);  *K. oxytoca* (48);  *E. aerogenes* (3);  *E. cloacae* (50);  *E. agglomerans* (1);  *C. sakazakii* (1);  *E. coli* (50) | Kęsik-Szeloch et al., 2013 |
| vB_KpnS_KP16 | no data | no data (clinical) | no data | no data | no | *K. pneumoniae subsp. ozaenae* (2);  *K. pneumoniae subsp. rhinoscleromatis* (2);  *K. oxytoca* (48);  *E. aerogenes* (3);  *E. cloacae* (50);  *E. agglomerans* (1);  *C. sakazakii* (1);  *E. coli* (50) | Kęsik-Szeloch et al., 2013 |
| vB_KpnS_KP36 | no data | no data (clinical) | no data | no data | no | *K. pneumoniae subsp. ozaenae* (2);  *K. pneumoniae subsp. rhinoscleromatis* (2);  *K. oxytoca* (48);  *E. aerogenes* (3);  *E. cloacae* (50);  *E. agglomerans* (1);  *C. sakazakii* (1);  *E. coli* (50) | Kęsik-Szeloch et al., 2013 |
| vB_KpnP_KP32 | no data | no data (clinical) | no data | no data | no | *K. pneumoniae subsp. ozaenae* (2);  *K. pneumoniae subsp. rhinoscleromatis* (2);  *K. oxytoca* (48);  *E. aerogenes* (3);  *E. cloacae* (50);  *E. agglomerans* (1);  *C. sakazakii* (1);  *E. coli* (50) | Kęsik-Szeloch et al., 2013 |
| vB_KpnP_KP34 | no data | no data (clinical) | no data | no data | no | *K. pneumoniae subsp. ozaenae* (2);  *K. pneumoniae subsp. rhinoscleromatis* (2);  *K. oxytoca* (48);  *E. aerogenes* (3);  *E. cloacae* (50);  *E. agglomerans* (1);  *C. sakazakii* (1);  *E. coli* (50) | Kęsik-Szeloch et al., 2013 |
| KP1 | no data | K16-KPN-13-022 (no data) | 9/11 | 81.82% | no | *A. baumannii* (1);  *C. freundii* (1);  *C. sakazakii* (1);  *E. coli* (2);  *P. mirabilis* (1);  *P. aeruginosa* (1);  *S. enterica* serovar Typhimurium (1);  *S. enterica* serovar Enteritis (1) | Kim et al., 2023 |
| KP12 | no data | K16-KPN-13-022 (no data) | 8/11 | 72.73% | no | *A. baumannii* (1);  *C. freundii* (1);  *C. sakazakii* (1);  *E. coli* (2);  *P. mirabilis* (1);  *P. aeruginosa* (1);  *S. enterica* serovar Typhimurium (1);  *S. enterica* serovar Enteritis (1) | Kim et al., 2023 |
| vB_KpnS_LmqsRe28-1 | no data | 2 (animal) | 10/26 | 38.46% | no data |  | Köhne et al., 2025 |
| vB_KpnM_LmqsRe27-1 | no data | 12 (animal) | 2/26 | 7.69% | no data |  | Köhne et al., 2025 |
| vB_KpnS_LmqsRe28-2 | no data | 3 (animal) | 8/26 | 30.77% | no data |  | Köhne et al., 2025 |
| Kpn5 | no data | B5055 (clinical) | no data | no data | no data |  | Kumari et al., 2010 |
| Kpn12 | no data | B5055 (clinical) | no data | no data | no data |  | Kumari et al., 2010 |
| KPn13 | no data | B5055 (clinical) | no data | no data | no data |  | Kumari et al., 2010 |
| Kpn17 | no data | B5055 (clinical) | no data | no data | no data |  | Kumari et al., 2010 |
| Kpn22 | no data | B5055 (clinical) | no data | no data | no data |  | Kumari et al., 2010 |
| CTF-1 | no data | no data (clinical) | 22/25 | 88% | no data |  | Kurt et al., 2025 |
| vB_KpnP_K3-ULINTkp1 | K3 | QAMH 130326/0185 (clinical) | 1/24 | 4.16% | yes: *K. oxytoca* | *E. coli* (8);  *K. oxytoca* (3);  *K. variicola* (3);  *K. quasipneumoniae* (1) | Laforêt et al., 2022 |
| vB_KpnP_K3-ULINTkp2 | K3 | QAMH 130326/0185 (clinical) | 3/24 | 12.50% | yes: *K. oxytoca* | *E. coli* (8);  *K. oxytoca* (3);  *K. variicola* (3);  *K. quasipneumoniae* (1) | Laforêt et al., 2022 |
| M198 | no data | 198 (clinical) | 60/101 | 59.41% | yes: *K. oxytoca* | *K. oxytoca* (2) | Leshkasheli et al., 2025 |
| P01 | K64 | 135080 (clinical) | 10/42 | 23.8% | no data |  | Li et al., 2024 |
| P545 | no data | KP4 (clinical) | 52/54 | 96.30% | no data |  | Li et al., 2020 |
| vB_KpnP_ZX1 | K57 | 111-2 (clinical) | 1/7 | 14.29% | no data |  | Li et al., 2022 |
| P1011 | K5 | B16 (animal) | 5/38 | 13.16% | no data |  | Li et al., 2024 |
| IME184 | no data | 1558 (clinical) | 19/74 | 25.68% | no | *E. coli* (10) | Li et al., 2022 |
| P509 | K64; K47 | Kp30 (clinical) | 35/54 | 64.81% | no | *P. aeruginosa* (9);  *A. baumannii* (10);  *E. coli* (3) | Li et al., 2020 |
| 175008 | no data | 135080 (clinical) | 10/30 | 33.33% | no | *E. coli* (11) | Li et al., 2025 |
| phiA85 | no data | A85 (clinical) | 22/50 | 44% | no | *P. aeruginosa* (2);  *E. coli* (2);  *A. baumannii* (2) | Li et al., 2025 |
| Henu2_3 | K1 | Kp1049 (clinical) | 6/15 | 40% | no | *E. coli* (1);  *A. baumannii* (1);  *P. aeruginosa* (1) | Li et al., 2025 |
| CM_Kpn_HB132952 | no data | KPHB132952 (clinical) | 30/31 | 96.77% | no | *S. aureus* (1);  *S. dysgalacticae* (1);  *E. coli* (3);  *S. agalacticae* (1) | Liang et al., 2022 |
| CM_Kpn_HB143742 | no data | KPHB143742 (clinical) | 30/31 | 96.77% | no | *S. aureus* (1);  *S. dysgalacticae* (1);  *E. coli* (3);  *S. agalacticae* (1) | Liang et al., 2022 |
| vB_KpnM_KpVB3 | K14; K64 | CRKP7 (no data) | 10/32 | 31.25% | no data |  | Liu et al., 2024 |
| N22 | no data | KP-ASM (no data) | no data | no data | no data |  | Liu et al., 2025 |
| φKp-lyy15 | no data | K1 (clinical) | 11/25 | 44% | no | *E. coli* (1);  *P. aeruginosa* (1) | Lu et al., 2015 |
| HZJ33 | no data | KP703 (clinical) | 12/30 | 40% | no data |  | Lu et al., 2025 |
| vB_Kpn_B01 | no data | 18 (animal) | no data | no data | no data |  | Luo et al., 2021 |
| myPSH1235 | K1; K2; K5 | no data (clinical) | 23/44 | 52.27% | no data |  | Manohar et al., 2019 |
| vB_KquU_φKuK6 | K6 | ATCC 700603 (reference strain) | 1/2 | 50% | no | *K. oxytoca* (1);  *C. freundii* (1);  *E. coli* (3);  *P. mirabilis* (1);  *P. aeruginosa* (2);  *S. enterica* serovar Tennessee (1);  *S. marcescens* (1);  *S. flexneri* (1);  *S. sonnei* (1) | Miller et al., 2024 |
| vB_KshKPC-M | no data | Kp100 (clinical) | 44/45 | 97.70% | no data |  | Mohammadi et al., 2023 |
| vB_KpnM_KP1 | no data | ATCC 13883 (reference strain) | 24/31 | 77.42% | yes: *K. oxytoca*; *K. aerogenes*; *K. quasipneumoniae* | *A. baumannii* (1);  *E. faecalis* (1);  *E. coli* (4);  *K. aerogenes* (1);  *K. oxytoca* (1);  *K. quasipneumoniae* (1);  *P. mirabilis* (1);  *P. aeruginosa* (1);  *S. enterica* serovar Typhimurium (1);  *S. enterica* serovar Typhi (1);  *S. enterica* serovar Anatum (1);  *S. flexneri* (1);  *Y. enterocolitica* (1) | Molina-López et al., 2025 |
| KP8 | no data | CEMTC 356 (clinical) | 1/50 | 2% | no | *C. freundii* (1);  *C. braakii* (1);  *Enterobacter* sp. (1);  *E. coli* (2);  *K. aerogenes* (2);  *K. oxytoca* (9);  *K. quasipneumoniae* (1);  *K. varicola* (1);  *P. vulgaris* (1);  *P. mirabilis* (1);  *Raoultella* sp. (3);  *S. enterica* (1) | Morozova et al., 2019 |
| PG14 | no data | G14 (clinical) | 4/4 | 100% | no | *S. aureus* (4);  *A. baumannii* (2);  *E. coli* (1);  *P. aeruginosa* (2) | Mulani et al., 2022 |
| vB_kpnP_KPYAP-1 | K62 | ST45 (clinical) | no data | no data | no data |  | Natarajan et al., 2024 |
| KA | no data | KP1 (clinical) | 7/15 | 46.67% | no | *P. aeruginosa*;  *E. coli* | Nawaz et al., 2025 |
| IME268 | no data | 1733 (clinical) | 19/74 | 25.68% | no | *E. coli* (10) | Nazir et al., 2022 |
| LASTA | K15 | Ni9 (clinical) | 5/140 | 3.57% | no data |  | Obradović et al., 2023 |
| SJM3 | K15 | Ni9 (clinical) | 5/140 | 3.57% | no data |  | Obradović et al., 2023 |
| vB_KpnM-VAC66 | no data | K3320 (clinical) | 30/48 | 62.50% | no data |  | Pacios et al., 2021 |
| vB_KpnM-VAC13 | no data | K2534 (clinical) | 12/16 | 75% | no data |  | Pacios et al., 2021 |
| vB_KPnM_KPDP1 | no data | yvu3 (no data) | no data | no data | no data |  | Pallavali et al., 2021 |
| vB_KleS-HSE3 | no data | 1025 (clinical) | 1/4 | 25% | yes: *Y. pseudotuberculosis* | *Y. pseudotuberculosis* (1);  *E. coli* (1);  *S. aureus* (1);  *A. baumannii* (1) | Peng et al., 2020 |
| vB_KleM_KB2 | no data | 0915 (clinical) | 2/4 | 50% | no | *E. coli* (3);  *A. baumannii* (5);  *Y. pseudotuberculosis* (1);  *S. aureus* (1);  *B. pumillus* (1) | Peng et al., 2023 |
| vB_Kp_XP4 | K1; K19 | P4 (clinical) | 2/21 | 9.52% | no data |  | Peng et al., 2025 |
| B1 | K2 | 52145 (clinical) | 3/21 | 14.29% | no data |  | Pertics et al., 2021 |
| 731 | K33; K21; K24 | 52145 (clinical) | 12/105 | 11.43% | no data |  | Pertics et al., 2023 |
| vB_KpS_GP-1 | no data | EuSCAPE_IT395 (clinical) | 10/36 | 27.78% | no data |  | Ponsecchi et al., 2024 |
| vB_KpP_GP-2 | no data | KP411 (clinical) | 6/36 | 16.67% | no data |  | Ponsecchi et al., 2024 |
| vB_KpP_GP-4 | no data | KP263 (clinical) | 11/36 | 30.56% | no data |  | Ponsecchi et al., 2024 |
| vB_KpP_GP-5 | no data | KP20-LU (clinical) | 3/36 | 8.33% | no data |  | Ponsecchi et al., 2024 |
| BUCT610 | no data | ST893 (clinical) | 1/20 | 5% | no data |  | Pu et al., 2022 |
| BUCT541 | K1 | S-2007 (clinical) | 7/30 | 23.33% | no data |  | Pu et al., 2022 |
| KP168 | no data | UA168 (clinical) | 13/20 | 65% | no data |  | Qi et al., 2020 |
| PSKP16 | K1; K2; K54 | BhvKp (clinical) | 4/30 | 13.33% | no data |  | Rahimi et al., 2023 |
| P12L | K2 | ATCC 43816 (reference strain) | 2/6 | 33.33% | no | *E. coli* (1);  *C. jejuni* (1);  *S. enterica* serovar Enteritidis (1);  *V. parahaemolyticus* (1);  *B. cereus* (1);  *E. durans* (1);  *E. faecalis* (1);  *L. monocytogenes* (1) | Rollon et al., 2025 |
| KZag1 | no data | K9 (clinical) | 12/15 | 80% | no | *S. aureus* (1);  *E. coli* (2);  *S. typhi* (1);  *P. aeruginosa* (1) | Saqr et al., 2024 |
| vB_Kpn_F13 | no data | KP5 (clinical) | 19/47 | 40.42% | no data |  | Senhaji-Kacha et al., 2024 |
| vB_Kpn_F14 | no data | KP5 (clinical) | 19/47 | 40.42% | no data |  | Senhaji-Kacha et al., 2024 |
| kpssk3 | no data | CRKP NY03 (clinical) | 25/57 | 43.86% | no data | *A. baumannii* (5);  *P. aeruginosa* (5);  *K. oxytoca* (5);  *E. coli* (5) | Shi et al., 2020 |
| vB_LZ 2044 | K1 | NTUH-K2044 (clinical) | 8/15 | 53.33% | no | *Salmonella* (2);  *P. aeruginosa* (2);  *E. coli* (2);  *S. aureus* (1) | Shi et al., 2025 |
| ΦKpnBHU1 | no data | KpnBHU09 (clinical) | 54/70 | 77.10% | no | *E. coli* (no data);  *P. aeruginosa* (no data);  *E. cloacae* (no data);  *A. lwoffii* (no data);  *S. enterica* serovar Typhi (no data);  *S. aureus* (no data);  *E. faecalis* (no data) | Singh et al., 2024 |
| ΦKpnBHU2 | no data | KpnBHU09 (clinical) | 50/70 | 71.40% | no | *E. coli* (no data);  *P. aeruginosa* (no data);  *E. cloacae* (no data);  *A. lwoffii* (no data);  *S. enterica* serovar Typhi (no data);  *S. aureus* (no data);  *E. faecalis* (no data) | Singh et al., 2024 |
| ΦKpnBHU3 | no data | KpnBHU09 (clinical) | 50/70 | 71.10% | no | *E. coli* (no data);  *P. aeruginosa* (no data);  *E. cloacae* (no data);  *A. lwoffii* (no data);  *S. enterica* serovar Typhi (no data);  *S. aureus* (no data);  *E. faecalis* (no data) | Singh et al., 2024 |
| KPP-5 | no data | CFS17 (food) | 19/19 | 100% | no | *E. coli* (6);  *S. enterica* serovar Typhimurium (3);  *P. aeruginosa* (3) | Sofy et al., 2021 |
| vB_KpnM-Teh.1 | no data | ATCC 10031 (reference strain) | 7/38 | 18.42% | no data |  | Soleimani Sasani & Eftekhar, 2020 |
| TSK1 | no data | ShA2 (environmental) | 4/8 | 50% | no | *E. coli* (3);  *P. aeruginosa* (1);  *E. cloacae* (2);  *A. baumannii* (1);  *S. aureus* (1) | Tabassum et al., 2018 |
| ZCKP1 | no data | KP/01 (clinical) | 15/21 | 71.43% | yes: *E. coli* and *P. mirabilis* | *P. mirabilis* (18);  *E. coli* (30) | Taha et al., 2018 |
| ΦFK1979 | K2 | FK1979 (clinical) | 5/35 | 14.29% | no data |  | Tang et al., 2023 |
| PKp-V1 | K1 | ST258 (animal) | 15/17 | 88.24% | no data |  | Tariq et al., 2024 |
| Henu1 | K1; K2; K57 | K2044 (clinical) | 18/176 | 10.23% | no | *Y. enterocolitica* (1);  *A. baumannii* (1);  *S. dysenteriae* (1);  *E. coli* (1);  *P. agglomerans* (1);  *P. dispersa* (1);  *E. cloacae* (1);  *S. marcescens* (1);  *P. aeruginosa* (1) | Teng et al., 2019 |
| K751 | no data | ST258 (clinical) | no data | no data | no data |  | Tisalema-Guanopatín et al., 2023 |
| T751 | no data | ST258 (clinical) | no data | no data | no data |  | Tisalema-Guanopatín et al., 2023 |
| T765 | no data | ST258 (clinical) | no data | no data | no data |  | Tisalema-Guanopatín et al., 2023 |
| PɸBw-Kp1 | no data | Iaufa_lad2 (clinical) | 1/6 | 16.67% | no data |  | Torabi et al., 2021 |
| PɸBw-Kp2 | no data | Iaufa_lad2 (clinical) | 3/6 | 50% | no data |  | Torabi et al., 2021 |
| PɸBw-Kp3 | no data | Iaufa_lad2 (clinical) | 1/6 | 16.67% | no data |  | Torabi et al., 2021 |
| KPO1K2 | no data | B5055 (clinical) | 7/25 | 28% | yes: *E. coli* | *E. coli* (no data);  *P. aeruginosa* (no data) | Verma et al., 2009 |
| vB_KpnP_23 | K64; K116; K107; K52; K47; K112; K17; K54; K149 | JNKPN23 (clinical) | 38/57 | 66.67% | no data |  | Wang et al., 2024 |
| Kpp-9 | K2 | kp09 (clinical) | 8/81 | 9.88% | no data |  | Wang et al., 2025 |
| KP1801 | no data | ESBL-KP PW006 (clinical) | 10/20 | 50% | no | *E. coli* (1);  *A. baumannii* (1);  *P. aeruginosa* (1);  *S. aureus* (1) | Wintachai et al., 2020 |
| vKPPW7 | K24 | KPPW67 (clinical) | 18/20 | 90% | no | *A. baumannii* (1);  *S. aureus* (1);  *E. coli* (1) | Wintachai et al., 2025 |
| Kpp95 | no data | ATCC 23357 (reference strain) | 65/107 | 60.75% | yes: *E. agglomerans;* *K. oxytoca;* *S. marcescens* | *A. baumannii* (10);  *E. agglomerans* (10);  *E. cloacae* (12);  *E. coli* (14);  *K. oxytoca* (14);  *P. mirabilis* (14);  *P. aeruginosa* (7);  *S. marcescens* (5) | Wu et al., 2007 |
| KlebP_265 | no data | CEMTC 5232 (reference strain) | 25/158 | 14.56% | yes: *K. oxytoca*; *K. aerogenes* | *K. oxytoca* (27);  *K. aerogenes* (8) | Yakubovskij et al., 2025 |
| vB_Kpn_HF0522 | K1 | KP1049 (clinical) | 9/47 | 19.15% | no data |  | Yan et al., 2025 |
| vB_Kpn_ZCKp20p | no data | K20 (clinical) | 12/32 | 37.50% | no data |  | Zaki et al., 2023 |
| vB_KpnP_Bp5 | K20 | GXKP-J05 (animal) | 1/36 | 2.78% | no |  | Zhang et al., 2021 |
| vB_KpnP_IME279 | K11; K37; K375 | 530 (clinical) | 10/20 | 50% | no data |  | Zhang et al., 2020 |
| Kpn_PImp2 | no data | IMP66 (clinical) | 4/12 | 33.33% | no | *E. coli* (2);  *V. parahaemolyticus* (1);  *Salmonella* sp. (1);  *S. aureus* (1) | Zhang et al., 2025 |
| Kpn_PImp3 | no data | IMP66 (clinical) | 6/12 | 50% | no | *E. coli* (2);  *V. parahaemolyticus* (1);  *Salmonella* sp. (1);  *S. aureus* (1) | Zhang et al., 2025 |
| ZK1 | K1 | Wkp26 (clinical | 45/95 | 47.37% | no data |  | Zhao et al., 2024 |
| p9676 | KL64 | ST11 (clinical) | 18/24 | 75% | no data |  | Zhao et al., 2025 |
| HZJ31 | no data | KPZ2 (clinical) | 7/30 | 23.33% | no data |  | Zhu et al., 2025 |
| HS37 | no data | KP 37 (clinical) | 12/18 | 66.67% | no | *V. parahaemolyticus* (5);  *E. coli* (3);  *S. aureus* (4);  *L. monocytogenes* (4) | Zhu et al., 2026 |
| LAPAZ | K30; K38; K58; K2 | KP01 (clinical) | no data | 30% | no data |  | Ziller et al., 2024 |
| vB_KpnS_FZ10 | no data | Kl 327 (clinical) | 8/14 | 57.14% | no data |  | Zurabov et al., 2021 |
| vB_KpnS_FZ41 | no data | Kl 325 (clinical) | 4/14 | 28.57% | no data |  | Zurabov et al., 2021 |
| vB_KpnP_FZ12 | no data | Kl 315 (clinical) | 10/14 | 71.43% | no data |  | Zurabov et al., 2021 |
| vB_KpnM_FZ14 | no data | Kl 263 (clinical) | 4/14 | 28.57% | no data |  | Zurabov et al., 2021 |

**Supplementary Table S12** – Bacterial strain species used in analyzed studies.

| **Bacterial strain species used in studies** |
| --- |
| *Achromobacter xylosoxidans* |
| *Acinetobacter baumannii* |
| *Acinetobacter lwoffii* |
| *Bacillus pumillus* |
| *Campylobacter jejuni* |
| *Citrobacter braakii* |
| *Citrobacter freundii* |
| *Citrobacter* sp. |
| *Cronobacter (Enterobacter) sakazakii* |
| *Enterobacter aerogenes* |
| *Enterobacter agglomerans* |
| *Enterobacter cloacae* |
| *Enterobacter* sp. |
| *Enterococcus durans* |
| *Enterococcus faecalis* |
| *Escherichia coli* |
| *Kerstersia gyiorum* |
| *Klebsiella aerogenes* |
| *Klebsiella alba* |
| *Klebsiella michiganensis* |
| *Klebsiella oxytoca* |
| *Klebsiella pneumoniae subsp. ozaenae* |
| *Klebsiella pneumoniae subsp. rhinoscleromatis* |
| *Klebsiella quasipneumoniae* |
| *Klebsiella variicola* |
| *Kluyvera cryocrescens* |
| *Morganella morganii* |
| *Pantoea agglomerans* |
| *Pantoea dispersa* |
| *Proteus mirabilis* |
| *Proteus vulgaris* |
| *Providencia alcalifaciens* |
| *Providencia* sp. |
| *Pseudomonas aeruginosa* |
| *Raoultella ornithinolytica* |
| *Raoultella planticola* |
| *Raoultella* sp. |
| *Salmonella enterica* |
| *Salmonella enterica* serovar Anatum |
| *Salmonella enterica* serovar Enteriditis |
| *Salmonella enterica* serovar Tennessee |
| *Salmonella enterica* serovar Typhi |
| *Salmonella enterica* serovar Typhimurium |
| *Serratia marcescens* |
| *Shigella dysenteriae* |
| *Shigella flexneri* |
| *Shigella sonnei* |
| *Staphylococcus agalacticae* |
| *Staphylococcus aureus* |
| *Staphylococcus epidermidis* |
| *Staphylococcus galacticae* |
| *Staphylococcus xylososus* |
| *Vibrio parahaemolyticus* |
| *Yersinia enterocolitica* |
| *Yersinia pseudotuberculosis* |
