# Supplementary material for: A comprehensive analysis of the kinetics of infection of lytic bacteriophages specific to the ESKAPE and critical pathogens
Source: World J Microbiol Biotechnol. 2026 Feb 28;42(3):110. doi: 10.1007/s11274-025-04762-4 (PMC12950090; doi:10.1007/s11274-025-04762-4)
Supplement: Supplementary file 4 — Supplementary file4 (DOCX 81 KB) [file 11274_2025_4762_MOESM4_ESM.docx]

**Supplementary Table S13** – Collected data regarding to phages infecting *A. baumannii* in terms of cycle parameters.

| **Phage designation** | **Host strain (source of isolation, if given)** | **Results of the studied multiplicity of infection (MOI)** | **Adsorption time [s]** | **Latent period [s]** | **Lysis time [s]** | **Burst size [PFU/cell]** | **Reference** |
| --- | --- | --- | --- | --- | --- | --- | --- |
| PMK34 | MK34 (clinical isolate, sputum) | 0.0001 | 240 | 1800 | no data | 128 | [Abdelkader Gutiérrez et al., 2020](https://doi.org/10.1128/AEM.01311-20) |
| TAC1 | AC5 (clinical isolate, sputum) | 1 | no data | 900 | no data | 454 | [Asif et al., 2020](https://doi.org/10.1007/s00705-019-04483-8) |
| Acba_6 | Ab_3940 (clinical isolate) | 0.001 | 900 | no data | no data | no data | [Bagińska et al., 2023](https://doi.org/10.3390/v15010231) |
| PBAB08 | AB28 (clinical isolate) | 0.01 | 300 | no data | 1500 | 215 | [Cha et al., 2018](https://doi.org/10.3389/fmicb.2018.00696) |
| PBAB25 | AB28 (clinical isolate) | 0.01 | 300 | no data | 1500 | 630 | Cha et al., 2018 |
| vB_AbaP_W8 | ATCC17978 (reference strain) | 0.01 | 60 | 300 | no data | 164 | [Choi et al., 2024](https://doi.org/10.3390/antibiotics13070610) |
| vB_AbaSi_W9 | ATCC17978 (reference strain) | 0.01 | 60 | 1200 | no data | 117 | [Choi et al., 2024](https://doi.org/10.3390/antibiotics13070680) |
| vB_AbaSt_W16 | KBN10P02782 (clinical isolate, blood) | 0.01 | 180 | 600 | no data | 1102 | [Choi et al., 2024](https://doi.org/10.3390/antibiotics13070610) |
| Acba_19 | 3940 (no data) | 0.1 | 600 | 300 | 4200 | 172 | Cieślik et al., 2025 |
| Acba_21 | 14 (clinical) | 0.1 | 750 | 1200 | 4500 | 49 | Cieślik et al., 2025 |
| P11B | TAB11B (clinical) | 0.1 | no data | 600 | 1800 | 118 | Deng et al., 2025 |
| AP22 | Ab1053 (clinical isolate) | 5 | 300 | 2400 | no data | 240 | [Dubrovin et al., 2012](https://doi.org/10.1371/journal.pone.0047348) |
| vB_AbaM_HB02 | ATCC19606 (reference strain) | 0.1 | 600 | 1200 | no data | 198 | [Erol et al., 2024](https://doi.org/10.1002/cbf.3966) |
| vB_AbaP_HB01 | ATCC19606 (reference strain) | 0.1 | 900 | 1800 | no data | 126 | [Erol et al., 2024](https://doi.org/10.1002/cbf.3966) |
| vB_AbaI-TMU2 | TMU2 (clinical isolate) | 0.1 | 480 | 1200 | no data | 80 | [Esmaeili-Fard-Barzegar et al., 2022](https://doi.org/10.18502/ijm.v14i5.10966) |
| Brutus | MAR15-3273 (no data) | 0.1 | 300 | 900 | no data | 77 | [Evseev et al., 2024](https://doi.org/10.3390/ijms25042074) |
| Scipio | LUH5534 (no data) | 0.1 | 300 | 600 | no data | 47 | [Evseev et al., 2024](https://doi.org/10.3390/ijms25042074) |
| vB‐AbauM‐Arak1 | XDR Ab (clinical isolate) | 0.01 | 600 | 1800 | no data | 200 | [Ghaznavi-Rad et al., 2022](https://doi.org/10.1002/jcla.24497) |
| øFG02 | AB900 (clinical) | 1 | 1200 | no data | no data | no data | [Gordillo Altamirano et al., 2022](https://doi.org/10.1016/j.ebiom.2022.104045) |
| ΦFG02 | AB900 (clinical) | 0.01 | no data | 2400 | no data | 15 | [Gordillo Altamirano et al., 2021](https://doi.org/10.1038/s41564-020-00830-7) |
| ΦCO01 | A9844 (clinical) | 0.01 | no data | 2460 | no data | 30 | Gordillo Altamirano et al., 2021 |
| vB_AbaP_AGC01 | ATCC 16909 (reference strain) | 0.1 | 300 | 1200 | no data | 317 | [Grygorcewicz et al., 2020](https://doi.org/10.3390/ijms21124390) |
| vB_Ab4_Hep4 | Ab4 (clinical) | 0.001 | 1200 | 3600 | no data | 110 | [He et al., 2024](https://doi.org/10.3389/fcimb.2024.1301089) |
| Petty | TP1/TP2/TP3 (clinical) | 0.01 | 600 | 1500 | no data | 240 | [Hernandez-Morales ACLessor et al., 2018](https://doi.org/10.1128/JVI.01064-17) |
| SH-Ab 15519 | Ab_15519 (clinical) | 10 | 600 | 600 | no data | 60 | [Hua et al., 2018](https://doi.org/10.3389/fmicb.2017.02659) |
| Abp1 | AB1 (clinical) | 0.001 | no data | 600 | 900 | 350 | [Huang et al., 2013](https://doi.org/10.1007/s00284-013-0308-7) |
| Abp95 | AB2013-95 (clinical) | no data | 600 | 1200 | 2400 | 167 | [Huang et al., 2023](https://doi.org/10.1038/s41598-022-26696-9) |
| KARL-1 | AB01 (clinical) | 0.1 | 720 | 1800 | no data | 42 | [Jansen et al., 2018](https://doi.org/10.1038/s41598-018-32344-y) |
| Bϕ-C62 | YMC13/01/C62 (clinical) | 10 | 60 | 1200 | no data | 76 | [Jeon et al., 2016](https://doi.org/10.1128/AEM.00526-16) |
| Βϕ-R2315 | YMC11/12/R2315 (clinical) | 0.1 | 300 | 2400 | no data | 78 | Jeon et al., 2016 |
| Βϕ-R3177 | YMC11/11/R3177 (clinical) | 10 | 300 | 3300 | no data | 286 | [Jeon et al., 2015](https://doi.org/10.1007/s00705-015-2604-y) |
| Βϕ-R1215 | YMC11/12/R1215 (clinical) | 0.1 | 300 | 1800 | no data | 43 | [Jeon et al., 2016](https://doi.org/10.1111/jam.13134) |
| Bϕ-R2096 | YMC13/03/R2096 (clinical) | 10 | 300 | 3000 | no data | 142 | [Jeon et al., 2019](https://doi.org/10.1186/s12866-019-1443-5) |
| Abp9 | ABZY9 (clinical) | no data | 900 | 1800 | no data | 158 | [Jiang et al., 2020](https://doi.org/10.3389/fmicb.2020.506068) |
| vB_AbaM-SHI | SL (clinical) | 1 | 600 | 3000 | no data | 155 | [Jiang et al., 2024](https://doi.org/10.3389/fcimb.2024.1351993) |
| ZZ1 | AB09V (clinical | 10 | 300 | 540 | no data | 200 | [Jin et al., 2012](https://doi.org/10.1186/1471-2180-12-156) |
| ØABP-02 | ATCC 19606 (reference strain) | 0.001 | no data | 1200 | no data | 120 | [Kitti et al., 2014](https://doi.org/10.1007/s12088-014-0472-x) |
| ØABP-04 | ATCC 19606 (reference strain) | 0.001 | no data | 1200 | no data | 150 | Kitti et al., 2014 |
| ØABP-01 | ATCC 19606 (reference strain) | 0.001 | no data | 900 | no data | 110 | [Kitti et al., 2014](https://doi.org/10.1007/s12088-014-0472-x) |
| vB-GEC_Ab-M-G7 | G7 (clinical) | 0.1 | 420 | 1200 | no data | 120 | [Kusradze et al., 2016](https://doi.org/10.3389/fmicb.2016.01590) |
| ϕAB1 | M68316 (clinical) | 0.0001 | 300 | no data | no data | no data | [Lai et al., 2016](https://doi.org/10.1371/journal.pone.0153361) |
| ϕAB6 | Ab 54149 (clinical) | 0.0001 | 300 | no data | no data | no data | [Lai et al., 2016](https://doi.org/10.1371/journal.pone.0153361) |
| Abp53 | Ab53 (clinical) | 0.1 | 360 | 600 | no data | 150 | [Lee et al., 2011](https://doi.org/10.1128/AEM.05116-11) |
| vB_AbaM_3054 | FER (clinical) | 100 | 600 | no data | no data | no data | [Leshkasheli et al., 2019](https://doi.org/10.1016/j.jgar.2019.05.005) |
| vB_AbaM_3090 | FER (clinical) | 100 | 600 | no data | no data | no data | [Leshkasheli et al., 2019](https://doi.org/10.1016/j.jgar.2019.05.005) |
| vChT04 | CR57 (clinical) | 0.001 | 600 | 600 | no data | 280 | [Leungtongkam et al., 2023](https://doi.org/10.1007/s00705-023-05862-y) |
| vB_AbaM_P1 | CGMCC 1.90331 (reference strain) | 0.1 | 720 | 1800 | no data | 788 | [Li et al., 2024](https://doi.org/10.1007/s00253-024-13208-0) |
| φAB2 | ATCC 17978 (reference strain) | 0.0001 | 480 | 600 | no data | 200 | [Lin et al., 2010](https://doi.org/10.1016/j.resmic.2010.03.007) |
| pB3074 | Bm3074 (clinical) | 0.01 | 600 | 1800 | no data | 85 | [Luo et al., 2023](https://doi.org/10.1128/spectrum.00341-23) |
| YC#06 | B.m#4015 (clinical) | 0.001 | no data | 2400 | 6600 | 116 | [Luo et al., 2022](https://doi.org/10.1128/spectrum.00096-22) |
| P425 | AB25 (clinical) | 0.1 | 600 | 600 | 3600 | 184 | Lin et al., 2025 |
| BUCT775 | 3808 (clinical) | 0.01 | no data | 600 | 3600 | 335.7 | Liu et al., 2025 |
| vB_AbaS_TCUP2199 | TV2199 (clinical) | 0.01 | 1200 | 1800 | no data | 196 | [Mardiana et al., 2022](https://doi.org/10.3390/v14061240) |
| vB_AbaM_Acibel004 | 070517/0072 (clinical) | 0.01 | 900 | 1620 | no data | 125 | [Merabishvili et al., 2014](https://doi.org/10.1371/journal.pone.0104853) |
| vB_AbaP_Acibel007 | 070517/0072 (clinical) | 0.01 | 600 | 1260 | no data | 145 | [Merabishvili et al., 2014](https://doi.org/10.1371/journal.pone.0104853) |
| vAbBal23 | AB12 (clinical) | no data | no data | 900 | no data | 2000 | Ndiaye et al., 2024 |
| vAbAbd25 | AB12 (clinical) | no data | no data | 900 | no data | 270 | Ndiaye et al., 2024 |
| vAbaIN10 | AB6 (clinical) | 0.01 | no data | 1500 | 900 | 4780 | Ndiaye et al., 2025 |
| vB_AbaM-IME-AB2 | MDR-AB2 (clinical) | 0.1 | 540 | 1200 | no data | 62 | [Peng et al., 2014](https://doi.org/10.1186/1471-2180-14-181) |
| vB_AbaM-DLP1 | AB5075 (clinical) | 0.001 | no data | 1200 | no data | 239 | [Peters et al., 2023](https://doi.org/10.3390/v15030739) |
| vB_AbaM-DLP2 | AB5075 (clinical) | 0.001 | no data | 1200 | no data | 24 | Peters et al., 2023 |
| vB_AbaP_AS11 | Ab 28 (clinical) | 0.001 | 300 | 1200 | no data | 150 | [Popova et al., 2017](https://doi.org/10.3390/v9070188) |
| vB_AbaP_AS12 | Ab 1432 (clinical) | 0.001 | 300 | 900 | no data | 300 | Popova et al., 2017 |
| AM24 | SCPM-Obolensk (clinical) | no data | 300 | 2700 | no data | 180 | [Popova et al., 2019](https://doi.org/10.1007/s00705-019-04208-x) |
| Pɸ-Bw-Ab | IAU_FAL101 (clinical) | 10 | 600 | 3000 | no data | 69 | [Rahimzadeh Torabi et al., 2021](https://doi.org/10.22038/ijbms.2021.57772.12850) |
| vB_AbaS_SA1 | Ab8 (clinical) | 0.1 | no data | 1200 | no data | 250 | Rastegar et al., 2024 |
| vB_Aba_QH4 | NO.424 (clinical) | 0.001 | 360 | 600 | 2400 | 460.5 | Ruizhe et al. 2025 |
| TaPaz | NIPH601 (clinical) | 0.0015 | 900 | 1800 | no data | 110 | [Shchurova et al., 2021](https://doi.org/10.3390/v13060978) |
| pIsf-AB02 | MDR-AB02 (clinical) | 0.01 | 600 | 1800 | 4200 | 120 | [Sisakhtpour et al., 2022](https://doi.org/10.1186/s12941-022-00492-9) |
| P1033 | ABMYH-1033 (clinical) | no data | no data | 1200 | no data | no data | [Soontarach et al., 2022](https://doi.org/10.3390/ph15040443) |
| T17 | ABJNSP-17 (clinical) | no data | 600 | 240 | no data | no data | [Soontarach et al., 2022](https://doi.org/10.3390/ph15040443) |
| T92 | ABJNH-92 (clinical) | no data | 600 | 360 | no data | no data | [Soontarach et al., 2022](https://doi.org/10.3390/ph15040443) |
| P521 | ABMASP-521 (clinical) | no data | 600 | 240 | no data | no data | [Soontarach et al., 2022](https://doi.org/10.3390/ph15040443) |
| P1051 | ABMYH-1051 (clinical) | no data | no data | 600 | no data | no data | [Soontarach et al., 2022](https://doi.org/10.3390/ph15040443) |
| P245 | ABMYSP-245 (clinical) | no data | no data | 2400 | no data | no data | [Soontarach et al., 2022](https://doi.org/10.3390/ph15040443) |
| T1245 | ABMYH-1245 (clinical) | no data | 120 | 240 | no data | no data | [Soontarach et al., 2022](https://doi.org/10.3390/ph15040443) |
| T444 | ABMSP-444 (clinical) | no data | 600 | 360 | no data | no data | [Soontarach et al., 2022](https://doi.org/10.3390/ph15040443) |
| T515 | ABMASP-515 (clinical) | no data | 360 | 240 | no data | no data | [Soontarach et al., 2022](https://doi.org/10.3390/ph15040443) |
| vB_AbaM_AB4P2 | AB4 (clinical) | 1 | no data | 1200 | 6600 | 61 | Su et al., 2025 |
| vB_AbaM_AB3P2 | AB3 (clinical) | 1 | no data | 600 | no data | 24 | Tan et al., 2024 |
| AB7-IBB2 | AIIMS 7 (clinical) | 0.001 | 240 | 1200 | 600 | 22 | [Thawal et al., 2012](https://doi.org/10.1007/s00284-012-0127-2) |
| Abgy202141 | GY-4 (clinical) | 1 | 300 | 300 | no data | 189 | [Tian et al., 2024](https://doi.org/10.3389/fmicb.2024.1379400) |
| Aristophanes | KZ1098 (clinical) | 0.0015 | 1200 | 1200 | no data | 15 | [Timoshina et al., 2021](https://doi.org/10.3390/v13091688) |
| Loki | ATCC 17978 (reference strain) | 0.1 | 300 | 2400 | 1800 | 43 | [Turner et al., 2017](https://doi.org/10.1371/journal.pone.0172303) |
| vB_AbaM_A72 | A72 (clinical) | 0.1 | no data | 720 | no data | 154 | Vera-Jauregui et al. 2025 |
| vB_AbaM_ISTD | 6077/12 (clinical) | 0.01 | 1800 | 1200 | no data | 114 | [Vukotic et al., 2020](https://doi.org/10.3389/fmed.2020.00426) |
| vB_AbaM_NOVI | 6077/12 (clinical) | 0.01 | 1800 | 1800 | no data | 55 | [Vukotic et al., 2020](https://doi.org/10.3389/fmed.2020.00426) |
| IME285 | Ab387 (clinical) | 0.001 | no data | 600 | no data | 450 | [Wang et al., 2020](https://doi.org/10.3389/fmicb.2020.01407) |
| HZY2308 | AB48 (clinical | 0.01 | no data | 1200 | 3000 | 4000000 | Wang et al., 2024 |
| Ab_WF01 | CRAB (clinical) | 0.001 | 900 | 600 | no data | 151 | [Wang et al., 2024](https://doi.org/10.3389/fcimb.2024.1382145) |
| vB_AbaM_ ABPW7 | ATCC 17978 (reference strain) | 0.1 | 900 | 1200 | no data | 147 | [Wintachai et al., 2022](https://doi.org/10.3390/v14112561) |
| vWUPSU | NPRCOE 160519 (clinical) | 0.01 | 600 | 1500 | no data | 153 | [Wintachai et al., 2022](https://doi.org/10.3390/ph15030291) |
| vB _AbP_ABWU2101 | ABPW0185 (clinical) | 0.01 | 900 | 1200 | no data | 283 | [Wintachai et al., 2022](https://doi.org/10.3390/v14020194) |
| vB_AbaP_WU2001 | CRAB ABPW052 (clinical | 0.1 | 600 | 1200 | 1800 | 230 | [Wintachai et al., 2022](https://doi.org/10.1038/s41598-022-06582-0) |
| AB1801 | NPRC AB11 (clinical) | 1 | 600 | 1200 | no data | 212 | [Wintachai et al., 2019](https://doi.org/10.1099/jmm.0.001002) |
| PD-6A3 | AB32 (clinical) | 1 | 300 | 1200 | no data | 129 | [Wu et al., 2019](https://doi.org/10.3389/fmicb.2018.03302) |
| AbTJ | ATCC 19606 (reference strain) | 0.01 | no data | 5400 | no data | 70 | [Xu et al., 2020](https://doi.org/10.3390/v12020205) |
| φAbp1 | ATCC 17978 (reference strain) | 0.1 | 300 | 600 | 1500 | no data | [Yang et al., 2019](https://doi.org/10.1128/mSystems.00068-19) |
| AB1 | KD311 (clinical) | 0.0001 | 1800 | 1060 | no data | 409 | [Yang et al., 2010](https://doi.org/10.1186/1471-2180-10-131) |
| φAbp2 | MDRAB (clinical) | 0.1 | 600 | 900 | 2100 | 222 | [Yang et al., 2019](https://doi.org/10.1007/s00705-019-04213-0) |
| AB7-IBB1 | AIIMS 7 (clinical) | 0.001 | 300 | 1800 | no data | 125 | [Yele et al., 2012](https://doi.org/10.1007/s00705-012-1320-0) |
| Phab24 | ATCC 17978 (reference strain) | 0.01 | no data | no data | 5400 | 350 | [Zhang et al., 2022](https://doi.org/10.1016/j.virusres.2022.198889) |
| MRABP9 | MRAB11 (clinical) | 0.001 | 900 | 300 | no data | 396 | [Zhang et al., 2024](https://doi.org/10.1016/j.virol.2024.110098) |
| P1068 | ZWAb014 (clinical) | 1 | no data | 600 | no data | 280 | [Zheng et al., 2024](https://doi.org/10.1016/j.virs.2024.08.002) |
| WCHABP1 | Ab1186 (clinical) | 0.1 | 600 | 600 | no data | 136 | [Zhou et al., 2018](https://doi.org/10.3389/fmicb.2018.00850) |
| WCHABP12 | Ab1262 (clinical) | 0.1 | 600 | 1200 | no data | 175 | [Zhou et al., 2018](https://doi.org/10.3389/fmicb.2018.00850) |
| BUCT628 | XDR Ab (no data) | 0.1 | no data | 1200 | no data | 260 | [Zhu et al., 2022](https://doi.org/10.1007/s00705-022-05425-7) |

**Supplementary Table S14** – Collected data regarding to phages infecting *A. baumannii* in terms of presence of ‘halo’ effect, type of phage morphology, phage gene accesion number.

| **Phage designation** | **Host strain** | **Presence of 'halo' effect** | **Type of phage morphology** | **Phage gene accesion number** | **Reference** |
| --- | --- | --- | --- | --- | --- |
| PMK34 | MK34 (clinical isolate, sputum) | yes | podovirus | MN433707 | [Abdelkader Gutiérrez et al., 2020](https://doi.org/10.1128/AEM.01311-20) |
| TAC1 | AC5 (clinical isolate, sputum) | no | myovirus | MK170160 | [Asif et al., 2020](https://doi.org/10.1007/s00705-019-04483-8) |
| Acba_6 | Ab_3940 (clinical isolate) | yes | podovirus | OQ101251 | [Bagińska et al., 2023](https://doi.org/10.3390/v15010231) |
| PBAB08 | AB28 (clinical isolate) | no data | myovirus | MG366114 | [Cha et al., 2018](https://doi.org/10.3389/fmicb.2018.00696) |
| PBAB25 | AB28 (clinical isolate) | no data | myovirus | MG366115 | Cha et al., 2018 |
| vB_AbaP_W8 | ATCC17978 (reference strain) | yes | podovirus | PP174318 | [Choi et al., 2024](https://doi.org/10.3390/antibiotics13070610) |
| vB_AbaSi_W9 | ATCC17978 (reference strain) | no | myovirus | PP146379.1 | [Choi et al., 2024](https://doi.org/10.3390/antibiotics13070680) |
| vB_AbaSt_W16 | KBN10P02782 (clinical isolate, blood) | no | myovirus | PP174317 | [Choi et al., 2024](https://doi.org/10.3390/antibiotics13070610) |
| Acba_19 | 3940 (no data) | yes | podovirus | PV067698 | Cieślik et al., 2025 |
| Acba_21 | 14 (clinical) | yes | myovirus | PV067692 | Cieślik et al., 2025 |
| P11B | TAB11B (clinical) | yes | myovirus | PV851422 | Deng et al., 2025 |
| AP22 | Ab1053 (clinical isolate) | yes | myovirus | HE806280.1 | [Dubrovin et al., 2012](https://doi.org/10.1371/journal.pone.0047348) |
| vB_AbaM_HB02 | ATCC19606 (reference strain) | no | myovirus | no data | [Erol et al., 2024](https://doi.org/10.1002/cbf.3966) |
| vB_AbaP_HB01 | ATCC19606 (reference strain) | yes | podovirus | OP917929.1 | [Erol et al., 2024](https://doi.org/10.1002/cbf.3966) |
| vB_AbaI-TMU2 | TMU2 (clinical isolate) | no | myovirus | no data | [Esmaeili-Fard-Barzegar et al., 2022](https://doi.org/10.18502/ijm.v14i5.10966) |
| Brutus | MAR15-3273 (no data) | yes | myovirus | ON036882 | [Evseev et al., 2024](https://doi.org/10.3390/ijms25042074) |
| Scipio | LUH5534 (no data) | yes | myovirus | ON036883 | [Evseev et al., 2024](https://doi.org/10.3390/ijms25042074) |
| vB‐AbauM‐Arak1 | XDR Ab (clinical isolate) | no | myovirus | no data | [Ghaznavi-Rad et al., 2022](https://doi.org/10.1002/jcla.24497) |
| øFG02 | AB900 (clinical) | no data | no data | no data | [Gordillo Altamirano et al., 2022](https://doi.org/10.1016/j.ebiom.2022.104045) |
| ΦFG02 | AB900 (clinical) | yes | myovirus | MT648818 | [Gordillo Altamirano et al., 2021](https://doi.org/10.1038/s41564-020-00830-7) |
| ΦCO01 | A9844 (clinical) | yes | myovirus | MT648819 | Gordillo Altamirano et al., 2021 |
| vB_AbaP_AGC01 | ATCC 16909 (reference strain) | yes | podovirus | MT263719 | [Grygorcewicz et al., 2020](https://doi.org/10.3390/ijms21124390) |
| vB_Ab4_Hep4 | Ab4 (clinical) | yes | podovirus | OP019135 | [He et al., 2024](https://doi.org/10.3389/fcimb.2024.1301089) |
| Petty | TP1/TP2/TP3 (clinical) | yes | podovirus | KF669656.1 | [Hernandez-Morales ACLessor et al., 2018](https://doi.org/10.1128/JVI.01064-17) |
| SH-Ab 15519 | Ab_15519 (clinical) | yes | podovirus | KY082667 | [Hua et al., 2018](https://doi.org/10.3389/fmicb.2017.02659) |
| Abp1 | AB1 (clinical) | yes | podovirus | JX658790 | [Huang et al., 2013](https://doi.org/10.1007/s00284-013-0308-7) |
| Abp95 | AB2013-95 (clinical) | yes | myovirus | MZ618622.1 | [Huang et al., 2023](https://doi.org/10.1038/s41598-022-26696-9) |
| KARL-1 | AB01 (clinical) | no | myovirus | MH713599 | [Jansen et al., 2018](https://doi.org/10.1038/s41598-018-32344-y) |
| Bϕ-C62 | YMC13/01/C62 (clinical) | no data | myovirus | KJ817802 | [Jeon et al., 2016](https://doi.org/10.1128/AEM.00526-16) |
| Βϕ-R2315 | YMC11/12/R2315 (clinical) | no | myovirus | KP861229 | Jeon et al., 2016 |
| Βϕ-R3177 | YMC11/11/R3177 (clinical) | no | siphovirus | KP861230 | [Jeon et al., 2015](https://doi.org/10.1007/s00705-015-2604-y) |
| Βϕ-R1215 | YMC11/12/R1215 (clinical) | no | myovirus | KP861231 | [Jeon et al., 2016](https://doi.org/10.1111/jam.13134) |
| Bϕ-R2096 | YMC13/03/R2096 (clinical) | yes | myovirus | KM672662 | [Jeon et al., 2019](https://doi.org/10.1186/s12866-019-1443-5) |
| Abp9 | ABZY9 (clinical) | yes | myovirus | MN166083 | [Jiang et al., 2020](https://doi.org/10.3389/fmicb.2020.506068) |
| vB_AbaM-SHI | SL (clinical) | yes | myovirus | ON480525 | [Jiang et al., 2024](https://doi.org/10.3389/fcimb.2024.1351993) |
| ZZ1 | AB09V (clinical | no | myovirus | HQ698922 | [Jin et al., 2012](https://doi.org/10.1186/1471-2180-12-156) |
| ØABP-02 | ATCC 19606 (reference strain) | yes | myovirus | no data | [Kitti et al., 2014](https://doi.org/10.1007/s12088-014-0472-x) |
| ØABP-04 | ATCC 19606 (reference strain) | yes | myovirus | no data | Kitti et al., 2014 |
| ØABP-01 | ATCC 19606 (reference strain) | no | podovirus | no data | [Kitti et al., 2014](https://doi.org/10.1007/s12088-014-0472-x) |
| vB-GEC_Ab-M-G7 | G7 (clinical) | no | myovirus | no data | [Kusradze et al., 2016](https://doi.org/10.3389/fmicb.2016.01590) |
| ϕAB1 | M68316 (clinical) | yes | podovirus | HQ186308 | [Lai et al., 2016](https://doi.org/10.1371/journal.pone.0153361) |
| ϕAB6 | Ab 54149 (clinical) | yes | podovirus | KT339321 | [Lai et al., 2016](https://doi.org/10.1371/journal.pone.0153361) |
| Abp53 | Ab53 (clinical) | no | myovirus | JF317274 | [Lee et al., 2011](https://doi.org/10.1128/AEM.05116-11) |
| vB_AbaM_3054 | FER (clinical) | yes | myovirus | no data | [Leshkasheli et al., 2019](https://doi.org/10.1016/j.jgar.2019.05.005) |
| vB_AbaM_3090 | FER (clinical) | no | myovirus | no data | [Leshkasheli et al., 2019](https://doi.org/10.1016/j.jgar.2019.05.005) |
| vChT04 | CR57 (clinical) | no data | podovirus | OQ858591 | [Leungtongkam et al., 2023](https://doi.org/10.1007/s00705-023-05862-y) |
| vB_AbaM_P1 | CGMCC 1.90331 (reference strain) | no | myovirus | OL960030 | [Li et al., 2024](https://doi.org/10.1007/s00253-024-13208-0) |
| φAB2 | ATCC 17978 (reference strain) | no data | podovirus | no data | [Lin et al., 2010](https://doi.org/10.1016/j.resmic.2010.03.007) |
| P425 | AB25 (clinical) | yes | podovirus | PQ211117 | Lin et al., 2025 |
| BUCT775 | 3808 (clinical) | no | podovirus | PV277657 | Liu et al., 2025 |
| pB3074 | Bm3074 (clinical) | yes | no data | OQ730192.1 | [Luo et al., 2023](https://doi.org/10.1128/spectrum.00341-23) |
| YC#06 | B.m#4015 (clinical) | yes | myovirus | ON391949.1 | [Luo et al., 2022](https://doi.org/10.1128/spectrum.00096-22) |
| vB_AbaS_TCUP2199 | TV2199 (clinical) | no | siphovirus | ON323491 | [Mardiana et al., 2022](https://doi.org/10.3390/v14061240) |
| vB_AbaM_Acibel004 | 070517/0072 (clinical) | no data | myovirus | NC_025462.1 | [Merabishvili et al., 2014](https://doi.org/10.1371/journal.pone.0104853) |
| vB_AbaP_Acibel007 | 070517/0072 (clinical) | no data | podovirus | KJ473423.1 | [Merabishvili et al., 2014](https://doi.org/10.1371/journal.pone.0104853) |
| vAbBal23 | AB12 (clinical) | yes | no data | ERR12951254 | Ndiaye et al., 2024 |
| vAbAbd25 | AB12 (clinical) | no | no data | ERR12951255 | Ndiaye et al., 2024 |
| vAbaIN10 | AB6 (clinical) | yes | no data | no data | Ndiaye et al., 2025 |
| vB_AbaM-IME-AB2 | MDR-AB2 (clinical) | no | myovirus | JX976549 | [Peng et al., 2014](https://doi.org/10.1186/1471-2180-14-181) |
| vB_AbaM-DLP1 | AB5075 (clinical) | yes | myovirus | OP946501.1 | [Peters et al., 2023](https://doi.org/10.3390/v15030739) |
| vB_AbaM-DLP2 | AB5075 (clinical) | no | myovirus | OP946502.1 | Peters et al., 2023 |
| vB_AbaP_AS11 | Ab 28 (clinical) | yes | podovirus | NC_041915.1 | [Popova et al., 2017](https://doi.org/10.3390/v9070188) |
| vB_AbaP_AS12 | Ab 1432 (clinical) | yes | podovirus | NC_041914.1 | Popova et al., 2017 |
| AM24 | SCPM-Obolensk (clinical) | yes | myovirus | KY000079 | [Popova et al., 2019](https://doi.org/10.1007/s00705-019-04208-x) |
| Pɸ-Bw-Ab | IAU_FAL101 (clinical) | yes | siphovirus | no data | [Rahimzadeh Torabi et al., 2021](https://doi.org/10.22038/ijbms.2021.57772.12850) |
| vB_AbaS_SA1 | Ab8 (clinical) | no | siphovirus | PP236949 | Rastegar et al., 2024 |
| vB_Aba_QH4 | NO.424 (clinical) | yes | myovirus | PQ227708 | Ruizhe et al. 2025 |
| TaPaz | NIPH601 (clinical) | yes | myovirus | MZ043613 | [Shchurova et al., 2021](https://doi.org/10.3390/v13060978) |
| pIsf-AB02 | MDR-AB02 (clinical) | no | myovirus | no data | [Sisakhtpour et al., 2022](https://doi.org/10.1186/s12941-022-00492-9) |
| P1033 | ABMYH-1033 (clinical) | no | myovirus | no data | [Soontarach et al., 2022](https://doi.org/10.3390/ph15040443) |
| T17 | ABJNSP-17 (clinical) | yes | no data | no data | [Soontarach et al., 2022](https://doi.org/10.3390/ph15040443) |
| T92 | ABJNH-92 (clinical) | yes | no data | no data | [Soontarach et al., 2022](https://doi.org/10.3390/ph15040443) |
| P521 | ABMASP-521 (clinical) | yes | no data | no data | [Soontarach et al., 2022](https://doi.org/10.3390/ph15040443) |
| P1051 | ABMYH-1051 (clinical) | no | no data | no data | [Soontarach et al., 2022](https://doi.org/10.3390/ph15040443) |
| P245 | ABMYSP-245 (clinical) | no | no data | no data | [Soontarach et al., 2022](https://doi.org/10.3390/ph15040443) |
| T1245 | ABMYH-1245 (clinical) | yes | podovirus | no data | [Soontarach et al., 2022](https://doi.org/10.3390/ph15040443) |
| T444 | ABMSP-444 (clinical) | yes | podovirus | no data | [Soontarach et al., 2022](https://doi.org/10.3390/ph15040443) |
| T515 | ABMASP-515 (clinical) | yes | podovirus | no data | [Soontarach et al., 2022](https://doi.org/10.3390/ph15040443) |
| vB_AbaM_AB4P2 | AB4 (clinical) | yes | myovirus | OR544125 | Su et al., 2025 |
| vB_AbaM_AB3P2 | AB3 (clinical) | yes | myovirus | OR526523 | Tan et al., 2024 |
| AB7-IBB2 | AIIMS 7 (clinical) | yes | podovirus | no data | [Thawal et al., 2012](https://doi.org/10.1007/s00284-012-0127-2) |
| Abgy202141 | GY-4 (clinical) | yes | myovirus | OR770645 | [Tian et al., 2024](https://doi.org/10.3389/fmicb.2024.1379400) |
| Aristophanes | KZ1098 (clinical) | no | podovirus | MT783706 | [Timoshina et al., 2021](https://doi.org/10.3390/v13091688) |
| Loki | ATCC 17978 (reference strain) | no | siphovirus | LN890663 | [Turner et al., 2017](https://doi.org/10.1371/journal.pone.0172303) |
| vB_AbaM_A72 | A72 (clinical) | yes | myovirus | PV437273.1 | Vera-Jauregui et al. 2025 |
| vB_AbaM_ISTD | 6077/12 (clinical) | yes | myovirus | no data | [Vukotic et al., 2020](https://doi.org/10.3389/fmed.2020.00426) |
| vB_AbaM_NOVI | 6077/12 (clinical) | yes | myovirus | no data | [Vukotic et al., 2020](https://doi.org/10.3389/fmed.2020.00426) |
| IME285 | Ab387 (clinical) | yes | myovirus | MH853786 | [Wang et al., 2020](https://doi.org/10.3389/fmicb.2020.01407) |
| HZY2308 | AB48 (clinical | yes | myovirus | OR730450 | Wang et al., 2024 |
| Ab_WF01 | CRAB (clinical) | yes | podovirus | OQ848592 | [Wang et al., 2024](https://doi.org/10.3389/fcimb.2024.1382145) |
| vB_AbaM_ ABPW7 | ATCC 17978 (reference strain) | yes | myovirus | OP562383 | [Wintachai et al., 2022](https://doi.org/10.3390/v14112561) |
| vWUPSU | NPRCOE 160519 (clinical) | yes | myovirus | OL743187 | [Wintachai et al., 2022](https://doi.org/10.3390/ph15030291) |
| vB _AbP_ABWU2101 | ABPW0185 (clinical) | yes | podovirus | OK546191.1 | [Wintachai et al., 2022](https://doi.org/10.3390/v14020194) |
| vB_AbaP_WU2001 | CRAB ABPW052 (clinical | yes | podovirus | MZ099557.1 | [Wintachai et al., 2022](https://doi.org/10.1038/s41598-022-06582-0) |
| AB1801 | NPRC AB11 (clinical) | yes | siphovirus | no data | [Wintachai et al., 2019](https://doi.org/10.1099/jmm.0.001002) |
| PD-6A3 | AB32 (clinical) | yes | podovirus | KY388102.1 | [Wu et al., 2019](https://doi.org/10.3389/fmicb.2018.03302) |
| AbTJ | ATCC 19606 (reference strain) | yes | podovirus | MK340941 | [Xu et al., 2020](https://doi.org/10.3390/v12020205) |
| φAbp1 | ATCC 17978 (reference strain) | no data | no data | NC_021316.1 | [Yang et al., 2019](https://doi.org/10.1128/mSystems.00068-19) |
| AB1 | KD311 (clinical) | yes | siphovirus | HM368260.1 | [Yang et al., 2010](https://doi.org/10.1186/1471-2180-10-131) |
| φAbp2 | MDRAB (clinical) | no | myovirus | no data | [Yang et al., 2019](https://doi.org/10.1007/s00705-019-04213-0) |
| AB7-IBB1 | AIIMS 7 (clinical) | no | siphovirus | no data | [Yele et al., 2012](https://doi.org/10.1007/s00705-012-1320-0) |
| Phab24 | ATCC 17978 (reference strain) | no data | myovirus | MZ477002 | [Zhang et al., 2022](https://doi.org/10.1016/j.virusres.2022.198889) |
| MRABP9 | MRAB11 (clinical) | yes | podovirus | OP727261.1 | [Zhang et al., 2024](https://doi.org/10.1016/j.virol.2024.110098) |
| P1068 | ZWAb014 (clinical) | yes | myovirus | OQ689089 | [Zheng et al., 2024](https://doi.org/10.1016/j.virs.2024.08.002) |
| WCHABP1 | Ab1186 (clinical) | yes | myovirus | KY829116 | [Zhou et al., 2018](https://doi.org/10.3389/fmicb.2018.00850) |
| WCHABP12 | Ab1262 (clinical) | yes | myovirus | KY670595 | [Zhou et al., 2018](https://doi.org/10.3389/fmicb.2018.00850) |
| BUCT628 | XDR Ab (no data) | yes | myovirus | MZ593728 | [Zhu et al., 2022](https://doi.org/10.1007/s00705-022-05425-7) |

**Supplementary Table S15** – Collected data regarding to phages infecting *A. baumannii* in terms of host range and polyvalence.

| **Phage designation** | **Host strain** | **Host range of the bacteriophage against *A. baumannii* strains (vulnerable/tested)** | **Percentage of host range** | **Activity against other species** | **Tested other species (number of tested strains)** | **Reference** |
| --- | --- | --- | --- | --- | --- | --- |
| PMK34 | MK34 (clinical isolate, sputum) | 1/10 | 10% | no data |  | [Abdelkader Gutiérrez et al., 2020](https://doi.org/10.1128/AEM.01311-20) |
| TAC1 | AC5 (clinical isolate, sputum) | 21/32 | 65.63% | no | *K. pneumoniae* (4);  *P. aeruginosa* (5);  *S. aureus* (5);  *E. coli* (4);  *E. cloacae* (3);  *B. cepacia* (2);  *S. marcescens* (1) | [Asif et al., 2020](https://doi.org/10.1007/s00705-019-04483-8) |
| Acba_6 | Ab_3940 (clinical isolate) | 10/53 | 18.87% | no | *P. aeruginosa* (2);  *K. pneumoniae* (1);  *E. coli* (2);  *E. cloacae* (1) | [Bagińska et al., 2023](https://doi.org/10.3390/v15010231) |
| PBAB08 | AB28 (clinical isolate) | no data | no data | no data |  | [Cha et al., 2018](https://doi.org/10.3389/fmicb.2018.00696) |
| PBAB25 | AB28 (clinical isolate) | no data | no data | no data |  | Cha et al., 2018 |
| vB_AbaP_W8 | ATCC17978 (reference strain) | 11/29 | 37.93% | no | *E. coli* (1);  *S. aureus* (1);  *K. pneumoniae* (1);  *P. aeruginosa* (1);  *E. faecalis* (1);  *E. faecium* (1) | [Choi et al., 2024](https://doi.org/10.3390/antibiotics13070610) |
| vB_AbaSi_W9 | ATCC17978 (reference strain) | 26/29 | 89.66% | no | *E. coli* (1);  *S. aureus* (1);  *K. pneumoniae* (1);  *P. aeruginosa* (1);  *E. faecalis* (1);  *E. faecium* (1) | [Choi et al., 2024](https://doi.org/10.3390/antibiotics13070680) |
| vB_AbaSt_W16 | KBN10P02782 (clinical isolate, blood) | 12/29 | 41.38% | no | *E. coli* (1);  *S. aureus* (1);  *K. pneumoniae* (1);  *P. aeruginosa* (1);  *E. faecalis* (1);  *E. faecium* (1) | [Choi et al., 2024](https://doi.org/10.3390/antibiotics13070610) |
| Acba_19 | 3940 (no data) | 3/59 | 5.08% | no | *Enterobacter* spp. (3);  *A. johnsonii* (1);  *E. coli* (1);  *P. aeruginosa* (5);  *S. aureus* (4); *Enterococcus* sp. (2) | Cieślik et al., 2025 |
| Acba_21 | 14 (clinical) | 8/59 | 13.56% | yes: *A. johnsonnii* | *Enterobacter* spp. (3);  *A. johnsonii* (1);  *E. coli* (1);  *P. aeruginosa* (5);  *S. aureus* (4);  *Enterococcus* spp. (2) | Cieślik et al., 2025 |
| P11B | TAB11B (clinical) | 13/144 | 9.03% | no data | no data | Deng et al., 2025 |
| AP22 | Ab1053 (clinical isolate) | no data | no data | no data |  | [Dubrovin et al., 2012](https://doi.org/10.1371/journal.pone.0047348) |
| vB_AbaM_HB02 | ATCC19606 (reference strain) | 39/50 | 78% | no data |  | [Erol et al., 2024](https://doi.org/10.1002/cbf.3966) |
| vB_AbaP_HB01 | ATCC19606 (reference strain) | 16/50 | 32% | no data |  | [Erol et al., 2024](https://doi.org/10.1002/cbf.3966) |
| vB_AbaI-TMU2 | TMU2 (clinical isolate) | 2/10 | 20% | yes: *P. aeruginosa* | *P. aeruginosa* (5);  *K. pneumoniae* (no data) | [Esmaeili-Fard-Barzegar et al., 2022](https://doi.org/10.18502/ijm.v14i5.10966) |
| Brutus | MAR15-3273 (no data) | no data | no data | no data |  | [Evseev et al., 2024](https://doi.org/10.3390/ijms25042074) |
| Scipio | LUH5534 (no data) | no data | no data | no data |  | [Evseev et al., 2024](https://doi.org/10.3390/ijms25042074) |
| vB‐AbauM‐Arak1 | XDR Ab (clinical isolate) | 12/19 | 63.16% | no | *P. aeruginosa* (2);  *E. coli* (2);  *P. mirabilis* (1);  *K. pneumoniae* (1);  *S. sonnei* (1);  *S. flexneri* (1);  *A. hydrophila* (1);  *B. cereus* (1);  *S. pneumoniae* (1);  *E. faecalis* (1);  *S. aureus* (1);  *S. saprophyticus* (1) | [Ghaznavi-Rad et al., 2022](https://doi.org/10.1002/jcla.24497) |
| øFG02 | AB900 (clinical) | no data | no data | no data |  | [Gordillo Altamirano et al., 2022](https://doi.org/10.1016/j.ebiom.2022.104045) |
| ΦFG02 | AB900 (clinical) | 2/9 | 22.22% | no data |  | [Gordillo Altamirano et al., 2021](https://doi.org/10.1038/s41564-020-00830-7) |
| ΦCO01 | A9844 (clinical) | 4/9 | 44.44% | no data |  | Gordillo Altamirano et al., 2021 |
| vB_AbaP_AGC01 | ATCC 16909 (reference strain) | 93/185 | 50.27% | no | *E. coli* (no data);  *Klebsiella* spp. (no data);  *Enterobacter* spp. (no data);  *Pseudomonas* spp. (no data) | [Grygorcewicz et al., 2020](https://doi.org/10.3390/ijms21124390) |
| vB_Ab4_Hep4 | Ab4 (clinical) | 3/22 | 13.64% | no data |  | [He et al., 2024](https://doi.org/10.3389/fcimb.2024.1301089) |
| Petty | TP1/TP2/TP3 (clinical) | 4/38 | 10.53% | no | Acinetobacter genospecies 16 (1);  *A. baylyi* (1) | [Hernandez-Morales ACLessor et al., 2018](https://doi.org/10.1128/JVI.01064-17) |
| SH-Ab 15519 | Ab_15519 (clinical) | no data | no data | no data |  | [Hua et al., 2018](https://doi.org/10.3389/fmicb.2017.02659) |
| Abp1 | AB1 (clinical) | 2/80 | 2.50% | no | *P. aeruginosa* (2);  *E.coli* (4) | [Huang et al., 2013](https://doi.org/10.1007/s00284-013-0308-7) |
| Abp95 | AB2013-95 (clinical) | 58/200 | 29% | no data |  | [Huang et al., 2023](https://doi.org/10.1038/s41598-022-26696-9) |
| KARL-1 | AB01 (clinical) | 16/20 | 80% | no data |  | [Jansen et al., 2018](https://doi.org/10.1038/s41598-018-32344-y) |
| Bϕ-C62 | YMC13/01/C62 (clinical) | 16/45 | 35% | no | *P. aeruginosa* (no data);  *E. coli* (no data) | [Jeon et al., 2016](https://doi.org/10.1128/AEM.00526-16) |
| Βϕ-R2315 | YMC11/12/R2315 (clinical) | 22/50 | 44% | yes: *S. aureus* | *P. aeruginosa* (5);  *E. coli* (5);  *B. subtilis* (5);  *S. aureus* (5) | Jeon et al., 2016 |
| Βϕ-R3177 | YMC11/11/R3177 (clinical) | 11/45 | 24.44% | no data |  | [Jeon et al., 2015](https://doi.org/10.1007/s00705-015-2604-y) |
| Βϕ-R1215 | YMC11/12/R1215 (clinical) | 22/50 | 44% | yes: *S. aureus* | *P. aeruginosa* (5);  *E. coli* (5);  *B. subtilis* (5);  *S. aureus* (5) | [Jeon et al., 2016](https://doi.org/10.1111/jam.13134) |
| Bϕ-R2096 | YMC13/03/R2096 (clinical) | 17/34 | 50% | no | *P. aeruginosa* (3);  *E. coli* (3) | [Jeon et al., 2019](https://doi.org/10.1186/s12866-019-1443-5) |
| Abp9 | ABZY9 (clinical) | 12/97 | 12% | no | *E. coli* (2);  *P. aeruginosa* (1) | [Jiang et al., 2020](https://doi.org/10.3389/fmicb.2020.506068) |
| vB_AbaM-SHI | SL (clinical) | no data | no data | no data |  | [Jiang et al., 2024](https://doi.org/10.3389/fcimb.2024.1351993) |
| ZZ1 | AB09V (clinical | 3/23 | 13.04% | no data |  | [Jin et al., 2012](https://doi.org/10.1186/1471-2180-12-156) |
| ØABP-02 | ATCC 19606 (reference strain) | 6/12 | 50% | no | *E. faecalis* (1);  *K. pneumoniae* (1);  *P. mirabilis* (1);  *P. vulgaris* (1);  *P. fluorescens* (1);  *S. typhil* (1);  *S. typhimurium* (1);  *S. flexneri* (1);  *V. parahaemolyticus* (1);  *B. cereus* (1);  *B. subtilis* (1);  *E. aerogenes* (1);  *E. coli* (1);  *P. aeruginosa* (1) | [Kitti et al., 2014](https://doi.org/10.1007/s12088-014-0472-x) |
| ØABP-04 | ATCC 19606 (reference strain) | 6/12 | 50% | no | *E. faecalis* (1);  *K. pneumoniae* (1);  *P. mirabilis* (1);  *P. vulgaris* (1);  *P. fluorescens* (1);  *S. typhil* (1);  *S. typhimurium* (1);  *S. flexneri* (1);  *V. parahaemolyticus* (1);  *B. cereus* (1);  *B. subtilis* (1);  *E. aerogenes* (1);  *E. coli* (1);  *P. aeruginosa* (1) | Kitti et al., 2014 |
| ØABP-01 | ATCC 19606 (reference strain) | 12/12 | 100% | no | *E. faecalis* (1);  *K. pneumoniae* (1);  *P. mirabilis* (1);  *P. vulgaris* (1);  *P. fluorescens* (1);  *S. typhil* (1);  *S. typhimurium* (1);  *S. flexneri* (1);  *V. parahaemolyticus* (1);  *B. cereus* (1);  *B. subtilis* (1);  *E. aerogenes* (1);  *E. coli* (1);  *P. aeruginosa* (1) | [Kitti et al., 2014](https://doi.org/10.1007/s12088-014-0472-x) |
| vB-GEC_Ab-M-G7 | G7 (clinical) | 168/200 | 68% | no data |  | [Kusradze et al., 2016](https://doi.org/10.3389/fmicb.2016.01590) |
| ϕAB1 | M68316 (clinical) | 191/832 | 22.96% | no data |  | [Lai et al., 2016](https://doi.org/10.1371/journal.pone.0153361) |
| ϕAB6 | Ab 54149 (clinical) | 483/832 | 58.05% | no data |  | [Lai et al., 2016](https://doi.org/10.1371/journal.pone.0153361) |
| Abp53 | Ab53 (clinical) | 16/26 | 61.54% | no | *E. coli* (2);  *S. maltophilia* (6);  *K. pneumoniae* (1);  *P. aeruginosa* (1);  *X. campestris* (2);  *X. oryzae* (1);  *X. vesicatoria* (1) | [Lee et al., 2011](https://doi.org/10.1128/AEM.05116-11) |
| vB_AbaM_3054 | FER (clinical) | 9/83 | 10.84% | no data |  | [Leshkasheli et al., 2019](https://doi.org/10.1016/j.jgar.2019.05.005) |
| vB_AbaM_3090 | FER (clinical) | 12/83 | 14.46% | no data |  | [Leshkasheli et al., 2019](https://doi.org/10.1016/j.jgar.2019.05.005) |
| vChT04 | CR57 (clinical) | 52/150 | 34.67% | no data |  | [Leungtongkam et al., 2023](https://doi.org/10.1007/s00705-023-05862-y) |
| vB_AbaM_P1 | CGMCC 1.90331 (reference strain) | 9/35 | 25.71% | no data |  | [Li et al., 2024](https://doi.org/10.1007/s00253-024-13208-0) |
| φAB2 | ATCC 17978 (reference strain) | 25/127 | 19.69% | no | *A. calcoaceticus* (1);  *E. coli* (10);  *K. pneumoniae* (6);  *P. aeruginosa* (3) | [Lin et al., 2010](https://doi.org/10.1016/j.resmic.2010.03.007) |
| P425 | AB25 (clinical) | 39/49 | 79.59% | no data | no data | Lin et al., 2025 |
| BUCT775 | 3808 (clinical) | 4/15 | 26.67% | no data | no data | Liu et al., 2025 |
| pB3074 | Bm3074 (clinical) | 14/34 | 41.18% | no | *E. coli* (1);  *P. aeruginosa* (1);  *S. pneumoniae* (1) | [Luo et al., 2023](https://doi.org/10.1128/spectrum.00341-23) |
| YC#06 | B.m#4015 (clinical) | 5/20 | 25% | no | *E. coli* (1);  *P. aeruginosa* (1);  *S. pneumoniae* (1) | [Luo et al., 2022](https://doi.org/10.1128/spectrum.00096-22) |
| vB_AbaS_TCUP2199 | TV2199 (clinical) | 189/208 | 90.86% | no data |  | [Mardiana et al., 2022](https://doi.org/10.3390/v14061240) |
| vB_AbaM_Acibel004 | 070517/0072 (clinical) | 15/28 | 44.12% | no | *A. pittii* (4);  *A. nosocomialis* (2) | [Merabishvili et al., 2014](https://doi.org/10.1371/journal.pone.0104853) |
| vB_AbaP_Acibel007 | 070517/0072 (clinical) | 15/28 | 44.12% | no | *A. pittii* (4);  *A. nosocomialis* (2) | [Merabishvili et al., 2014](https://doi.org/10.1371/journal.pone.0104853) |
| vAbBal23 | AB12 (clinical) | no data | no data | no data |  | Ndiaye et al., 2024 |
| vAbAbd25 | AB12 (clinical) | no data | no data | no data |  | Ndiaye et al., 2024 |
| vAbaIN10 | AB6 (clinical) | 3/11 | 27.27% | no data |  | Ndiaye et al., 2025 |
| vB_AbaM-IME-AB2 | MDR-AB2 (clinical) | no data | no data | no data |  | [Peng et al., 2014](https://doi.org/10.1186/1471-2180-14-181) |
| vB_AbaM-DLP1 | AB5075 (clinical) | 15/107 | 14.02% | no data |  | [Peters et al., 2023](https://doi.org/10.3390/v15030739) |
| vB_AbaM-DLP2 | AB5075 (clinical) | 21/107 | 19.63% | no data |  | Peters et al., 2023 |
| vB_AbaP_AS11 | Ab 28 (clinical) | 4/100 | 4% | no data |  | [Popova et al., 2017](https://doi.org/10.3390/v9070188) |
| vB_AbaP_AS12 | Ab 1432 (clinical) | 6/100 | 6% | no data |  | Popova et al., 2017 |
| AM24 | SCPM-Obolensk (clinical) | 14/105 | 13.33% | no data |  | [Popova et al., 2019](https://doi.org/10.1007/s00705-019-04208-x) |
| Pɸ-Bw-Ab | IAU_FAL101 (clinical) | 5/16 | 31.25% | no | *E. coli* (9);  *K. pneumoniae* (6) | [Rahimzadeh Torabi et al., 2021](https://doi.org/10.22038/ijbms.2021.57772.12850) |
| vB_AbaS_SA1 | Ab8 (clinical) | 10/30 | 33.33% | no | *K. pneumoniae* (5);  *E. coli* (5);  *P. aeruginosa* (5) | Rastegar et al., 2024 |
| vB_Aba_QH4 | NO.424 (clinical) | 2/80 | 2.5% | no | *P. aeruginosa* (20) | Ruizhe et al. 2025 |
| TaPaz | NIPH601 (clinical) | 1/56 | 1.79% | no data |  | [Shchurova et al., 2021](https://doi.org/10.3390/v13060978) |
| pIsf-AB02 | MDR-AB02 (clinical) | 27/48 | 56.25% | no | *P. aeruginosa* (1);  *E. coli* (1);  *K. pneumoniae* (1) | [Sisakhtpour et al., 2022](https://doi.org/10.1186/s12941-022-00492-9) |
| P1033 | ABMYH-1033 (clinical) | 23/49 | 46.94% | no data |  | [Soontarach et al., 2022](https://doi.org/10.3390/ph15040443) |
| T17 | ABJNSP-17 (clinical) | 2/49 | 4.08% | no data |  | [Soontarach et al., 2022](https://doi.org/10.3390/ph15040443) |
| T92 | ABJNH-92 (clinical) | 2/49 | 4.08% | no data |  | [Soontarach et al., 2022](https://doi.org/10.3390/ph15040443) |
| P521 | ABMASP-521 (clinical) | 9/49 | 18.37% | no data |  | [Soontarach et al., 2022](https://doi.org/10.3390/ph15040443) |
| P1051 | ABMYH-1051 (clinical) | 12/49 | 24.49% | no data |  | [Soontarach et al., 2022](https://doi.org/10.3390/ph15040443) |
| P245 | ABMYSP-245 (clinical) | 23/49 | 46.94% | no data |  | [Soontarach et al., 2022](https://doi.org/10.3390/ph15040443) |
| T1245 | ABMYH-1245 (clinical) | 9/49 | 18.37% | no data |  | [Soontarach et al., 2022](https://doi.org/10.3390/ph15040443) |
| T444 | ABMSP-444 (clinical) | 3/49 | 6.12% | no data |  | [Soontarach et al., 2022](https://doi.org/10.3390/ph15040443) |
| T515 | ABMASP-515 (clinical) | 2/49 | 4.08% | no data |  | [Soontarach et al., 2022](https://doi.org/10.3390/ph15040443) |
| vB_AbaM_AB4P2 | AB4 (clinical) | 2/7 | 28.57% | no | *Salmonella* sp. (4);  *E. coli* (5);  *P. aeruginosa* (2);  *S. enterica* (no data) | Su et al., 2025 |
| vB_AbaM_AB3P2 | AB3 (clinical) | 2/5 | 40% | no | *E. coli* (5);  *P. aeruginosa* (2) | Tan et al., 2024 |
| AB7-IBB2 | AIIMS 7 (clinical) | 19/39 | 48.72% | no | *A. haemolyticus* (1);  *A. calcoaceticus* (1);  *A. lwoffii* (1) | [Thawal et al., 2012](https://doi.org/10.1007/s00284-012-0127-2) |
| Abgy202141 | GY-4 (clinical) | no data | no data |  |  | [Tian et al., 2024](https://doi.org/10.3389/fmicb.2024.1379400) |
| Aristophanes | KZ1098 (clinical) | 1/56 | 1.79% | no data |  | [Timoshina et al., 2021](https://doi.org/10.3390/v13091688) |
| Loki | ATCC 17978 (reference strain) | 34/37 | 91.98% | yes: all despite one strain of *A. baumannii-calcoaceticus*) | *A. baylyi* (1);  *A. lwoffii* (1);  *A. calcoaceticus* (1);  *A. baumannii-calcoaceticus* (2) | [Turner et al., 2017](https://doi.org/10.1371/journal.pone.0172303) |
| vB_AbaM_A72 | A72 (clinical) | 2/19 | 10.53% | no | *S. aureus* (2);  *E. coli* (1);  *S. enterica* (1);  *P. aeruginosa* (1);  *L. monocytogenes* (1) | Vera-Jauregui et al. 2025 |
| vB_AbaM_ISTD | 6077/12 (clinical) | 37/103 | 35.92% | no data |  | [Vukotic et al., 2020](https://doi.org/10.3389/fmed.2020.00426) |
| vB_AbaM_NOVI | 6077/12 (clinical) | 23/103 | 22.33% | no data |  | [Vukotic et al., 2020](https://doi.org/10.3389/fmed.2020.00426) |
| IME285 | Ab387 (clinical) | 9/49 | 18.37% | no data |  | [Wang et al., 2020](https://doi.org/10.3389/fmicb.2020.01407) |
| HZY2308 | AB48 (clinical | 17/43 | 39.53% | no data |  | Wang et al., 2024 |
| Ab_WF01 | CRAB (clinical) | no data | no data | no | *E. faecium* (1)*;*  *S. aureus* (1);  *K. pneumoniae* (1)*;*  *P. aeruginosa* (1);  *E. coli* (1) | [Wang et al., 2024](https://doi.org/10.3389/fcimb.2024.1382145) |
| vB_AbaM_ ABPW7 | ATCC 17978 (reference strain) | 9/21 | 42.86% | no | *E. coli* (1);  *K. pneumoniae* (1);  *S. aureus* (1);  *P. aeruginosa* (1) | [Wintachai et al., 2022](https://doi.org/10.3390/v14112561) |
| vWUPSU | NPRCOE 160519 (clinical) | 16/30 | 53.30% | no | *E. coli* (1);  *K. pneumoniae* (1);  *S. aureus* (1);  *P. aeruginosa* (1) | [Wintachai et al., 2022](https://doi.org/10.3390/ph15030291) |
| vB _AbP_ABWU2101 | ABPW0185 (clinical) | 14/20 | 70% | no | *K. pneumoniae* (1);  *S. aureus* (1) | [Wintachai et al., 2022](https://doi.org/10.3390/v14020194) |
| vB_AbaP_WU2001 | CRAB ABPW052 (clinical | 24/43 | 55.81% | no | *E. coli* (1);  *K. pneumoniae* (1);  *S. aureus* (1);  *P. aeruginosa* (1) | [Wintachai et al., 2022](https://doi.org/10.1038/s41598-022-06582-0) |
| AB1801 | NPRC AB11 (clinical) | 7/10 | 70% | no | *E. coli* (1);  *K. pneumoniae* (1);  *S. aureus* (1);  *P. aeruginosa* (1) | [Wintachai et al., 2019](https://doi.org/10.1099/jmm.0.001002) |
| PD-6A3 | AB32 (clinical) | 179/552 | 32.40% | no data | no data | [Wu et al., 2019](https://doi.org/10.3389/fmicb.2018.03302) |
| AbTJ | ATCC 19606 (reference strain) | 5/5 | 100% | no | *A. haemolyticus* (4);  *B. subtilis* (1);  *S. aureus* (1);  *E. coli* (8);  *P. pastoris* (1) | [Xu et al., 2020](https://doi.org/10.3390/v12020205) |
| φAbp1 | ATCC 17978 (reference strain) | no data | no data | no data |  | [Yang et al., 2019](https://doi.org/10.1128/mSystems.00068-19) |
| AB1 | KD311 (clinical) | 1/4 | 25% | no | *S. maltophilia* (1);  *P. aeruginosa* (3) | [Yang et al., 2010](https://doi.org/10.1186/1471-2180-10-131) |
| φAbp2 | MDRAB (clinical) | 16/60 | 26.67% | no data |  | [Yang et al., 2019](https://doi.org/10.1007/s00705-019-04213-0) |
| AB7-IBB1 | AIIMS 7 (clinical) | 23/39 | 58.97% | no | *A. haemolyticus* (1);  *A. calcoaceticus* (1);  *A. lwoffii* (1) | [Yele et al., 2012](https://doi.org/10.1007/s00705-012-1320-0) |
| Phab24 | ATCC 17978 (reference strain) | no data | no data | no data |  | [Zhang et al., 2022](https://doi.org/10.1016/j.virusres.2022.198889) |
| MRABP9 | MRAB11 (clinical) | 3/11 | 27.27% | no | *K. pneumoniae* (4);  *E.coli* (1) | [Zhang et al., 2024](https://doi.org/10.1016/j.virol.2024.110098) |
| P1068 | ZWAb014 (clinical) | 18/94 | 19.15% | no data |  | [Zheng et al., 2024](https://doi.org/10.1016/j.virs.2024.08.002) |
| WCHABP1 | Ab1186 (clinical) | 9/18 | 50% | no data |  | [Zhou et al., 2018](https://doi.org/10.3389/fmicb.2018.00850) |
| WCHABP12 | Ab1262 (clinical) | 12/18 | 66.67% | no data |  | [Zhou et al., 2018](https://doi.org/10.3389/fmicb.2018.00850) |
| BUCT628 | XDR Ab (no data) | no data | no data | no data |  | [Zhu et al., 2022](https://doi.org/10.1007/s00705-022-05425-7) |

**Supplementary Table S16** – Bacterial strain species used in analyzed studies.

| **Bacterial strain species used in studies** |
| --- |
| *Acinetobacter baumannii* |
| *Acinetobacter baylyi* |
| *Acinetobacter calcoaceticus* |
| *Acinetobacter haemolyticus* |
| *Acinetobacter lwoffii* |
| *Acinetobacter nosocomialis* |
| *Acinetobacter pittii* |
| *Aeromonas hydrophila* |
| *Bacillus cereus* |
| *Bacillus subtilis* |
| *Burkholderia cepacia* |
| *Enterobacter aerogenes* |
| *Enterococcus faecalis* |
| *Enterococcus faecium* |
| *Escherichia coli* |
| *Klebsiella pneumoniae* |
| *Pichia pastoris* |
| *Proteus mirabilis* |
| *Proteus vulgaris* |
| *Pseudomonas aeruginosa* |
| *Pseudomonas fluorescens* |
| *Salmonella typhi* |
| *Salmonella typhimurium* |
| *Salmonella enterica* serovar Enteritis |
| *Salmonella enterica* serovar Indiana |
| *Salmonella enterica* serovar London |
| *Salmonella enterica* serovar Typhimurium |
| *Salmonella enterica* serovar Weltevreden |
| *Shigella flexneri* |
| *Shigella sonnei* |
| *Staphylococcus aureus* |
| *Staphylococcus saprophyticus* |
| *Stenotrophomonas maltophilia* |
| *Streptococcus pneumoniae* |
| *Vibrio parahaemolyticus* |
| *Xanthomonas campestris* |
| *Xanthomonas oryzae* |
| *Xanthomonas vesicatoria* |
