# Supplementary material for: A comprehensive analysis of the kinetics of infection of lytic bacteriophages specific to the ESKAPE and critical pathogens
Source: World J Microbiol Biotechnol. 2026 Feb 28;42(3):110. doi: 10.1007/s11274-025-04762-4 (PMC12950090; doi:10.1007/s11274-025-04762-4)
Supplement: Supplementary file 5 — Supplementary file5 (DOCX 91 KB) [file 11274_2025_4762_MOESM5_ESM.docx]

**Supplementary Table S17** – Collected data regarding to phages infecting *P. aeruginosa* in terms of cycle parameters.

| **Phage designation** | **Host strain (source of isolation, if given)** | **Results of the studied multiplicity of infection (MOI)** | **Adsorption time [s]** | **Latent period [s]** | **Lysis time [s]** | **Burst size [PFU/cell]** | **Reference** |
| --- | --- | --- | --- | --- | --- | --- | --- |
| vB_PaeM_PS3 | PS3 (no data) | 0.1 | no data | 600 | no data | 132 | Abdelghafar et al., 2023 |
| vB_PaeP_PS28 | PS28 (no data) | 0.1 | no data | 900 | 1500 | 210 | Abdelghafar et al., 2023 |
| phPS127 | PS700 (clinical) | 0.01 | 300 | 1200 | no data | 356 | Abo Kamer et al., 2022 |
| MA-1 | PA2949 (clinical) | no data | no data | 1800 | no data | 330 | Adnan et al., 2020 |
| BVPaP-3 | HS6 (clinical) | 0.07 | 420 | 1200 | no data | 44 | Ahiwale et al., 2012 |
| Ps12on-D | CN573 (clinical) | 0.01 | 600 | 600 | 1200 | 115 | Akremi et al., 2022 |
| ZC03 | PA14 (clinical) | 0.01 | no data | 3000 | no data | 10 | Amgarten et al., 2017 |
| vB-PaeP-007 | Pae30 (no data) | 0.01 | 480 | 600 | no data | 93 | Barazandeh et al., 2021 |
| PEV2 | PAV237 (animal) | no data | no data | 1500 | no data | 125 | Ceyssens et al., 2010 |
| Banzai | PAO1 (clinical) | 0.001 | 900 | 3000 | 3000 | 104 | Chaplin et al., 2025 |
| PpY1 | ATCC 15692 (reference strain) | 0.01 | no data | 900 | 2700 | no data | Cong et al., 2024 |
| vB_PaeP_ASP23 | L64 (animal) | no data | no data | 600 | no data | 140 | Cui et al., 2023 |
| C11 | TJC422 (clinical) | 0.01 | no data | 1080 | no data | 11 | Cui et al., 2016 |
| Phage I | 1950 (clinical) | 0.1 | 300 | 1200 | 2400 | 270 | Dai et al., 2023 |
| Phage II | 1950 (clinical) | 0.1 | 600 | 2700 | 1200 | 220 | Dai et al., 2023 |
| Phage III | 1950 (clinical) | 0.1 | 600 | 1800 | 1200 | 60 | Dai et al., 2023 |
| Phage IV | 1950 (clinical) | 0.1 | 600 | 1500 | 1800 | 70 | Dai et al., 2023 |
| vB_PaeM_KT28 | ATCC 15692 (reference strain) | no data | no data | 2100 | no data | 64 | Danis-Wlodarczyk et al., 2015 |
| vB_PaeM_KTN6 | ATCC 15692 (reference strain) | no data | no data | 2100 | no data | 96 | Danis-Wlodarczyk et al., 2015 |
| ϕPSZ1 | no data (clinical) | <0.1 | no data | 720 | 900 | 100 | El Didamony et al., 2015 |
| ϕPSZ2 | no data (clinical) | <0.1 | no data | 900 | 1200 | 100 | El Didamony et al., 2015 |
| PaBG | ATCC 15692 (reference strain) | 0.01 | 600 | 4800 | no data | 35 | Evseev et al., 2020 |
| Φ4_ZP1 | MA4 (clinical) | 0.001 | no data | 600 | 600 | 8.5 | Fiscarelli et al., 2021 |
| Φ9_ZP2 | MA4 (clinical) | 0.001 | no data | 1800 | 900 | 11 | Fiscarelli et al., 2021 |
| Φ14_OBG | MA4 (clinical) | 0.001 | no data | 2100 | 600 | 12.7 | Fiscarelli et al., 2021 |
| Φ17_OBG | ATCC 15692 (reference strain) | 0.001 | no data | 1200 | 1800 | 34 | Fiscarelli et al., 2021 |
| Φ19_OBG | ATCC 15692 (reference strain) | 0.001 | no data | 2400 | 300 | 4 | Fiscarelli et al., 2021 |
| JG024 | ATCC 15692 (reference strain) | 0.16 | no data | 3000 | no data | 180 | Garbe et al., 2010 |
| JG004 | no data (clinical) | no data | no data | 1860 | no data | 13 | Garbe et al., 2011 |
| φDCL-PA6 | PA14 (clinical) | 0.01 | 600 | 600 | no data | 80 | García-Cruz et al., 2024 |
| vB_PaeM_SCUT-S1 | ATCC 15692 (reference strain) | 0.01 | no data | 2400 | 600 | 134 | Guo et al., 2019 |
| vB_PaeM_SCUT-S2 | ATCC 15692 (reference strain) | 0.01 | no data | 1500 | 1500 | 40 | Guo et al., 2019 |
| ph0031 | DSM19880 (reference strain) | 0.001 | 1800 | 1500 | no data | 20 | Harada et al., 2022 |
| ph0034 | DSM19880 (reference strain) | 0.001 | 1800 | 600 | no data | 28 | Harada et al., 2022 |
| FMD5 | no data (animal) | 0.01 | no data | 300 | 3300 | 200 | Hu et al., 2025 |
| H24-1 | no data (animal) | 0.1 | no data | 600 | 3000 | 150 | Hu et al., 2025 |
| Ka2 | PAO1 (clinical) | 0.001 | no data | no data | 2400 | 300 | Ilyina et al., 2025 |
| MIJ3 | ATCC 15692 (reference strain) | 0.01 | 600 | 2700 | no data | 68 | Imam et al., 2019 |
| AZ1 | 2995 (clinical) | no data | no data | 1980 | no data | 326 | Jamal et al., 2017 |
| Bϕ-R656 | YMC11/02/R656 (clinical) | 0.001 | 300 | 1800 | no data | 253 | Jeon et al., 2019 |
| Bϕ-R1836 | YMC11/11/R1836 (clinical) | 0.001 | 600 | 1800 | no data | 499 | Jeon et al., 2019 |
| PA-YS35 | YS35 (clinical | 0.01 | no data | 540 | 1260 | 380 | Jiang et al., 2020 |
| vB_PaeP_TUMS_P121 | ATCC 27853 (reference strain) | 0.01 | no data | 2400 | 3600 | 92 | Kamyab et al., 2022 |
| vB_PaeS_TUMS_P81 | ATCC 27853 (reference strain) | 0.001 | 420 | 1800 | 2400 | 104 | Kamyab et al., 2023 |
| PhPa-4 | no data (clinical) | 0.001 | 1200 | 2100 | no data | 97 | Kanwar et al., 2025 |
| PhPa-6 | no data (clinical) | 0.001 | 1800 | 1800 | no data | 67 | Kanwar et al., 2025 |
| PSPa | ATCC 15692 (reference strain) | 0.001 | no data | 1200 | no data | 165 | Karthika et al., 2023 |
| APPa | ATCC 15692 (reference strain) | 0.001 | no data | 1800 | no data | 278 | Karthika et al., 2023 |
| vB_Pae-Kakheti25 | PA25 (clinical) | 0.1 | no data | 1200 | no data | 130 | Karumidze et al., 2012 |
| vB_Pae-TbilisiM32 | PA32 (clinical) | 0.1 | no data | 1200 | no data | 210 | Karumidze et al., 2012 |
| PA1Ø | ATCC 15692 (reference strain) | no data | 120 | 600 | 900 | 261 | Kim et al., 2012 |
| vB_Pae_HB2107-3I | PA14 (clinical) | 0.01 | no data | 600 | 3000 | 1100 | Kong et al., 2023 |
| vB_PaeS_LmqsRe25-1 | 29 (animal) | 0.002 | no data | 2400 | no data | 0.03 | Köhne et al., 2025 |
| PSA-KC1 | no data (no data) | no data | no data | 2100 | 2100 | 85 | Kurt et al., 2025 |
| vB_PaeM_MAG1 | Pa6, Pa21, Pa14, Pa18, Pa26 and Pa31/2 (clinical) | 0.1 | 300 | 900 | no data | 26 | Kwiatek et al., 2017 |
| vB_PaeP_MAG4 | Pa6, Pa21, Pa14, Pa18, Pa26 and Pa31/2 (clinical) | 0.1 | 300 | 900 | no data | 33 | Kwiatek et al., 2017 |
| pPa_SNUABM_DT01 | no data (clinical) | 0.01 | 300 | 2400 | no data | 147.91 | Kwon et al., 2021 |
| vB_PaeP_Tr60_Ab31 | PA14 (reference strain) | 0.01 | 240 | no data | no data | 40 | Latino et al., 2014 |
| SL1 | MDR-PA1 (clinical) | 0.01 | no data | 2700 | no data | 100 | Latz et al., 2017 |
| SL2 | MDR-PA2 (clinical) | 0.01 | no data | 1800 | no data | 22 | Latz et al., 2017 |
| SL4 | MDR-PA4 (clinical) | 0.01 | no data | 3000 | no data | 16 | Latz et al., 2017 |
| φKMV | ATCC 15692 (reference strain) | no data | no data | 750 | no data | 27 | Lavigne et al., 2003 |
| vB_PaeS_VL1 | ATCC 27853 (reference strain) | 0.001 | 600 | 1800 | no data | 404 | Lerdsittikul et al., 2022 |
| vB_PaP_HN01 | ATCC 27853 (reference strain) | 0.1 | 600 | 900 | 4200 | 3370 | Li et al., 2024 |
| vB_PaP_HN01 | ATCC 27853 (reference strain) | 0.1 | 600 | 900 | 4200 | 3370 | Li et al., 2025 |
| vB_PaeP_PZH3 | PA18 (clinical) | 0.01 | no data | 600 | 4200 | 959 | Ma et al., 2025 |
| PPaMa1/18 | Isolate 7 (no data) | 0.01 | no data | 1200 | no data | 58 | Majdani et al., 2022 |
| Sem-1 | ATCC 27853 (reference strain) | 0.01 | no data | 840 | 420 | 100 | Majlesain et al., 2025 |
| PaB1 | no data (clinical) | 0.1 | 540 | 2298 | no data | 240 | Marashi et al., 2022 |
| PaBa2 | no data (clinical) | 0.1 | 519,16 | 2202 | no data | 250 | Marashi et al., 2022 |
| PaBa3 | no data (clinical) | 0.1 | 499,8 | 2298 | no data | 220 | Marashi et al., 2022 |
| AM.P2 | ATCC 15692 (reference strain) | 0.1 | no data | 1200 | 1200 | no data | Menon et al., 2021 |
| Pseudomonas phage_AUS034 | AUS34 (clinical) | 0.01 | 300 | 3000 | no data | 143 | Namonyo et al., 2022 |
| Pseudomonas phage_AUS260 | AUS260 (clinical) | 0.01 | 300 | 3600 | no data | 86 | Namonyo et al., 2022 |
| Pseudomonas phage_AUS301 | AUS301 (clinical) | 0.01 | 300 | 4800 | no data | 121 | Namonyo et al., 2022 |
| Pseudomonas phage_AUS391 | AUS391 (clinical) | 0.01 | 300 | 5400 | no data | 51 | Namonyo et al., 2022 |
| PAA | PAZMYU (clinical) | 1 | 600 | 1800 | no data | 47 | Nawaz et al., 2025 |
| PAM | PAZMYU (clinical) | 1 | 300 | 2400 | no data | 83 | Nawaz et al., 2025 |
| AA17 | no data (clinical) | 0.001 | no data | 1200 | no data | 27 | Nour El-Din et al., 2025 |
| U17 | no data (clinical) | 0.001 | no data | 900 | no data | 185 | Nour El-Din et al., 2025 |
| AC20 | no data (clinical) | 0.001 | no data | 900 | no data | 96 | Nour El-Din et al., 2025 |
| AA20 | no data (clinical) | 0.001 | no data | 1800 | no data | 311 | Nour El-Din et al., 2025 |
| ΦPA01 | ATCC 15692 (reference strain) | 0.01 | no data | 1800 | no data | 32 | Ong et al., 2020 |
| ΦPA02 | ATCC 15692 (reference strain) | 0.01 | no data | 2100 | no data | 49 | Ong et al., 2020 |
| vB_PAnP_PADP4 | yvu1 (no data) | no data | no data | 1200 | no data | 102 | Pallavali et al., 2021 |
| vB_PaeM_CEB_DP1 | ATCC 15692 (reference strain) | no data | no data | 3000 | 3000 | 70 | Pires et al., 2015 |
| vB_PaS-HSN4 | ATCC 15442 (reference strain) | 0.01 | 300 | 1200 | 1800 | 119 | Rafiei et al., 2024 |
| DRL-P1 | MTCC 1688 (reference strain) | 0.1 | 300 | 1800 | no data | 100 | Sharma et al., 2021 |
| Phage_Pae01 | Pa021 (animal) | 0.01 | no data | 1800 | 1800 | no data | Shi et al., 2024 |
| vB_PaeP_LP14 | L7 (environmental) | 0.1 | no data | 300 | 4500 | 785 | Shi et al., 2020 |
| Pa_WF01 | CRPA (clinical) | 0.0001 | no data | 600 | 2400 | 154 | Sun et al., 2025 |
| vB_PaeM-AL | PA2 (clinical) | 0.001 | no data | 4800 | 2400 | 188 | Sutnu et al., 2024 |
| PUTH1 | B-I-1 (clinical) | 10 | 360 | 1800 | 3000 | 54.5 | Tan et al., 2025 |
| PA_LZ01 | ATCC 15692 (reference strain) | no data | no data | 2400 | 1200 | 15 | Wang et al., 2023 |
| PA_LZ02 | PA14 (clinical) | no data | no data | 3600 | 3000 | 50 | Wang et al., 2023 |
| JJ01 | ATCC 15692 (reference strain) | 0.01 | 600 | 1800 | no data | 109 | Wannasrichan et al., 2022 |
| vB_Pae_LC3I3 | PA14 (no data) | 0.0001 | no data | 1800 | 9000 | 1120 | Xuan et al., 2023 |
| L5 | PAO1r (laboratory strain) | 0.01 | no data | 1200 | 4200 | no data | Yang et al., 2022 |
| vB_PaeP_Lx18 | Pae-M3 (animal) | 1 | no data | 1200 | 6000 | 91 | Yin et al., 2022 |
| PX1 | ATCC 15692 (reference strain) | 0.01 | no data | 3000 | no data | 60 | Yu et al., 2015 |
| PEf1 | ATCC 15692 (reference strain) | 0.01 | no data | 2400 | no data | 72 | Yu et al., 2015 |
| PAXYB1 | ATCC 15692 (reference strain) | 0.001 | 180 | 1800 | 6000 | 141 | Yu et al., 2017 |
| vB_PaeM_LS1 | DLG (animal | 0.01 | no data | 1800 | no data | 98 | Yuan et al., 2019 |
| PPAT | ATCC 15692 (reference strain) | 0.2 | 120 | 1200 | 1800 | 953 | Yuanyuan et al., 2022 |
| PPAY | ATCC 15692 (reference strain) | 0.2 | 600 | 1200 | 1800 | 457 | Yuanyuan et al., 2022 |
| vB_PaeP_YL1 | PA27 (clinical) | 0.1 | no data | 600 | 5400 | 125 | Zhang et al., 2024 |
| vB_PaeP_YL2 | PA27 (clinical) | 0.1 | no data | 1200 | 4800 | 94 | Zhang et al., 2024 |
| O4 | ATCC 15692 (reference strain) | 0.001 | no data | 1800 | 1500 | 100 | Zhang et al., 2018 |
| vB_PaeP_YZ2 | no data | 0.01 | no data | 300 | 10500 | 265 | Zhang et al., 2025 |
| vB_PaeP_YQZQ | no data | 0.01 | no data | 300 | 8400 | 266 | Zhang et al., 2025 |
| vB_PaeP_QSZH | no data | 0.01 | no data | 600 | 8400 | 352 | Zhang et al., 2025 |

**Supplementary Table S18** – Collected data regarding to phages infecting *P. aeruginosa* in terms of presence of ‘halo’ effect, type of phage morphology, phage gene accesion number.

| **Phage designation** | **Host strain (source of isolation, if given)** | **Presence of 'halo' effect** | **Type of phage morphology** | **Phage gene accesion number** | **Reference** |
| --- | --- | --- | --- | --- | --- |
| vB_PaeM_PS3 | PS3 (no data) | yes | myovirus | OQ411628 | Abdelghafar et al., 2023 |
| vB_PaeP_PS28 | PS28 (no data) | no | podovirus | OQ134474.1 | Abdelghafar et al., 2023 |
| phPS127 | PS700 (clinical) | yes | siphovirus | no data | Abo Kamer et al., 2022 |
| MA-1 | PA2949 (clinical) | no | myovirus | no data | Adnan et al., 2020 |
| BVPaP-3 | HS6 (clinical) | no data | podovirus | no data | Ahiwale et al., 2012 |
| Ps12on-D | CN573 (clinical) | yes | no data | OM870967 | Akremi et al., 2022 |
| ZC03 | PA14 (clinical) | no | podovirus | KU356690 | Amgarten et al., 2017 |
| vB-PaeP-007 | Pae30 (no data) | no | podovirus | no data | Barazandeh et al., 2021 |
| PEV2 | PAV237 (animal) | no data | podovirus | NC_031063.1 | Ceyssens et al., 2010 |
| Banzai | PAO1 (clinical) | no data | myovirus | PV661111 | Chaplin et al., 2025 |
| PpY1 | ATCC 15692 (reference strain) | yes | podovirus | PQ463998 | Cong et al., 2024 |
| vB_PaeP_ASP23 | L64 (animal) | yes | podovirus | MN602045 | Cui et al., 2023 |
| C11 | TJC422 (clinical) | no data | myovirus | KT804923 | Cui et al., 2016 |
| Phage I | 1950 (clinical) | no data | siphovirus | no data | Dai et al., 2023 |
| Phage II | 1950 (clinical) | no data | myovirus | no data | Dai et al., 2023 |
| Phage III | 1950 (clinical) | no data | myovirus | no data | Dai et al., 2023 |
| Phage IV | 1950 (clinical) | no data | siphovirus | no data | Dai et al., 2023 |
| vB_PaeM_KT28 | ATCC 15692 (reference strain) | yes | myovirus | KP340287 | Danis-Wlodarczyk et al., 2015 |
| vB_PaeM_KTN6 | ATCC 15692 (reference strain) | yes | myovirus | KP340288 | Danis-Wlodarczyk et al., 2015 |
| ϕPSZ1 | no data (clinical) | no data | podovirus | no data | El Didamony et al., 2015 |
| ϕPSZ2 | no data (clinical) | no data | podovirus | no data | El Didamony et al., 2015 |
| PaBG | ATCC 15692 (reference strain) | yes | myovirus | KF147891 | Evseev et al., 2020 |
| Φ4_ZP1 | MA4 (clinical) | yes | podovirus | no data | Fiscarelli et al., 2021 |
| Φ9_ZP2 | MA4 (clinical) | yes | podovirus | no data | Fiscarelli et al., 2021 |
| Φ14_OBG | MA4 (clinical) | yes | podovirus | no data | Fiscarelli et al., 2021 |
| Φ17_OBG | ATCC 15692 (reference strain) | no | podovirus | no data | Fiscarelli et al., 2021 |
| Φ19_OBG | ATCC 15692 (reference strain) | no | podovirus | no data | Fiscarelli et al., 2021 |
| JG024 | ATCC 15692 (reference strain) | no data | myovirus | GU815091 | Garbe et al., 2010 |
| JG004 | no data (clinical) | no data | myovirus | GU988610 | Garbe et al., 2011 |
| φDCL-PA6 | PA14 (clinical) | no data | myovirus | OR436899 | García-Cruz et al., 2024 |
| vB_PaeM_SCUT-S1 | ATCC 15692 (reference strain) | no | myovirus | MK340760 | Guo et al., 2019 |
| vB_PaeM_SCUT-S2 | ATCC 15692 (reference strain) | no | myovirus | MK340761 | Guo et al., 2019 |
| ph0031 | DSM19880 (reference strain) | yes | myovirus | MW526258 | Harada et al., 2022 |
| ph0034 | DSM19880 (reference strain) | yes | myovirus | MW526259 | Harada et al., 2022 |
| FMD5 | no data (animal) | yes | podovirus | PP107937 | Hu et al., 2025 |
| H24‑1 | no data (animal) | no | myovirus | PP504668 | Hu et al., 2025 |
| Ka2 | PAO1 (clinical) | no | myovirus | ON529291 | Ilyina et al., 2025 |
| MIJ3 | ATCC 15692 (reference strain) | no | myovirus | LR588166 | Imam et al., 2019 |
| AZ1 | 2995 (clinical) | no | siphovirus | no data | Jamal et al., 2017 |
| Bϕ-R656 | YMC11/02/R656 (clinical) | no | siphovirus | KT968831.1 | Jeon et al., 2019 |
| Bϕ-R1836 | YMC11/11/R1836 (clinical) | no | siphovirus | KT968832.1 | Jeon et al., 2019 |
| PA-YS35 | YS35 (clinical | no | myovirus | MF974178.1 | Jiang et al., 2020 |
| vB_PaeP_TUMS_P121 | ATCC 27853 (reference strain) | yes | podovirus | MZ955867 | Kamyab et al., 2022 |
| vB_PaeS_TUMS_P81 | ATCC 27853 (reference strain) | no | podovirus | OL519844 | Kamyab et al., 2023 |
| PhPa‑4 | no data | no data | podovirus | no data | Kanwar et al., 2025 |
| PhPa‑6 | no data | no data | siphovirus | no data | Kanwar et al., 2025 |
| PSPa | ATCC 15692 (reference strain) | no | myovirus | MN131143.1 | Karthika et al., 2023 |
| APPa | ATCC 15692 (reference strain) | no | siphovirus | MT118302.1 | Karthika et al., 2023 |
| vB_Pae-Kakheti25 | PA25 (clinical) | no | siphovirus | JQ307387 | Karumidze et al., 2012 |
| vB_Pae-TbilisiM32 | PA32 (clinical) | no | podovirus | JQ307386 | Karumidze et al., 2012 |
| PA1Ø | ATCC 15692 (reference strain) | no data | siphovirus | no data | Kim et al., 2012 |
| vB_Pae_HB2107-3I | PA14 (clinical) | no data | podovirus | ON778006 | Kong et al., 2023 |
| vB_PaeS_LmqsRe25‑1 | 29 (animal) | no | siphovirus | no data | Köhne et al., 2025 |
| PSA‑KC1 | no data (no data) | no | no data | OQ412632 | Kurt et al., 2025 |
| vB_PaeM_MAG1 | Pa6, Pa21, Pa14, Pa18, Pa26 and Pa31/2 (clinical) | no data | myovirus | KR052143 | Kwiatek et al., 2017 |
| vB_PaeP_MAG4 | Pa6, Pa21, Pa14, Pa18, Pa26 and Pa31/2 (clinical) | no data | podovirus | KR052142 | Kwiatek et al., 2017 |
| pPa_SNUABM_DT01 | no data (clinical) | no data | myovirus | MW735835 | Kwon et al., 2021 |
| vB_PaeP_Tr60_Ab31 | PA14 (reference strain) | no data | podovirus | HG798806 | Latino et al., 2014 |
| SL1 | MDR-PA1 (clinical) | no data | myovirus | MF768470 | Latz et al., 2017 |
| SL2 | MDR-PA2 (clinical) | no data | myovirus | MF805716 | Latz et al., 2017 |
| SL4 | MDR-PA4 (clinical) | no data | podovirus | MF768469 | Latz et al., 2017 |
| φKMV | ATCC 15692 (reference strain) | no data | podovirus | AJ505558.22262835 | Lavigne et al., 2003 |
| vB_PaeS_VL1 | ATCC 27853 (reference strain) | no | podovirus | OK665488 | Lerdsittikul et al., 2022 |
| vB_PaP_HN01 | ATCC 27853 (reference strain) | yes | myovirus | PP100125 | Li et al., 2024 |
| vB_PaP_HN01 | ATCC 27853 (reference strain) | yes | podovirus | PP100125 | Li et al., 2025 |
| vB_PaeP_PZH3 | PA18 (clinical) | no | podovirus | PQ562891 | Ma et al., 2025 |
| PPaMa1/18 | Isolate 7 (no data) | yes | myovirus | no data | Majdani et al., 2022 |
| Sem‑1 | ATCC 27853 (reference strain) | no data | no data | OQ326580.1 | Majlesain et al., 2025 |
| PaB1 | no data (clinical) | no | myovirus | PP931177.1 | Marashi et al., 2022 |
| PaBa2 | no data (clinical) | no | myovirus | no data | Marashi et al., 2022 |
| PaBa3 | no data (clinical) | no | podovirus | no data | Marashi et al., 2022 |
| AM.P2 | ATCC 15692 (reference strain) | no | podovirus | MT416090 | Menon et al., 2021 |
| Pseudomonas phage_AUS034 | AUS34 (clinical) | yes | podovirus | MW512831 | Namonyo et al., 2022 |
| Pseudomonas phage_AUS260 | AUS260 (clinical) | no data | siphovirus | MW512832 | Namonyo et al., 2022 |
| Pseudomonas phage_AUS301 | AUS301 (clinical) | yes | myovirus | MW512833 | Namonyo et al., 2022 |
| Pseudomonas phage_AUS391 | AUS391 (clinical) | no data | myovirus | MW512834 | Namonyo et al., 2022 |
| PAA | PAZMYU (clinical) | no | siphovirus | no data | Nawaz et al., 2025 |
| PAM | PAZMYU (clinical) | no | siphovirus | no data | Nawaz et al., 2025 |
| AA17 | no data (clinical) | no data | siphovirus | PP916319 | Nour El‑Din et al., 2025 |
| U17 | no data (clinical) | no data | podovirus | PP916318 | Nour El‑Din et al., 2025 |
| AC20 | no data (clinical) | no data | myovirus | PP916317 | Nour El‑Din et al., 2025 |
| AA20 | no data (clinical) | no data | myovirus | PP916316 | Nour El‑Din et al., 2025 |
| ΦPA01 | ATCC 15692 (reference strain) | no data | myovirus | AP019535 | Ong et al., 2020 |
| ΦPA02 | ATCC 15692 (reference strain) | no data | myovirus | AP019418 | Ong et al., 2020 |
| vB_PAnP_PADP4 | yvu1 (no data) | no data | podovirus | no data | Pallavali et al., 2021 |
| vB_PaeM_CEB_DP1 | ATCC 15692 (reference strain) | no data | myovirus | KR869157 | Pires et al., 2015 |
| vB_PaS-HSN4 | ATCC 15442 (reference strain) | no | podovirus | LC648443.1 | Rafiei et al., 2024 |
| DRL-P1 | MTCC 1688 (reference strain) | no | myovirus | MN564818 | Sharma et al., 2021 |
| Phage_Pae01 | Pa021 (animal) | no | myovirus | OR858750 | Shi et al., 2024 |
| vB_PaeP_LP14 | L7 (environmental) | no | podovirus | MH356729 | Shi et al., 2020 |
| vB_PaeM-AL | PA2 (clinical) | yes | myovirus | no data | Sutnu et al., 2024 |
| Pa_WF01 | CRPA (clinical) | no | podovirus | OQ848593 | Sun et al., 2025 |
| PUTH1 | B‑I‑1 (clinical) | no data | podovirus | PV521973 | Tan et al., 2025 |
| PA_LZ01 | ATCC 15692 (reference strain) | no | myovirus | OM953790.1 | Wang et al., 2023 |
| PA_LZ02 | PA14 (clinical) | no | myovirus | OQ646789 | Wang et al., 2023 |
| JJ01 | ATCC 15692 (reference strain) | yes | myovirus | ON324181.1 | Wannasrichan et al., 2022 |
| vB_Pae_LC3I3 | PA14 (no data) | yes | siphovirus | ON778007 | Xuan et al., 2023 |
| L5 | PAO1r (laboratory strain) | no | podovirus | OL754589 | Yang et al., 2022 |
| vB_PaeP_Lx18 | Pae-M3 (animal) | yes | podovirus | MN692672 | Yin et al., 2022 |
| PX1 | ATCC 15692 (reference strain) | yes | podovirus | no data | Yu et al., 2015 |
| PEf1 | ATCC 15692 (reference strain) | yes | siphovirus | no data | Yu et al., 2015 |
| PAXYB1 | ATCC 15692 (reference strain) | no data | podovirus | KY618819 | Yu et al., 2017 |
| vB_PaeM_LS1 | DLG (animal | yes | myovirus | MG897799 | Yuan et al., 2019 |
| PPAT | ATCC 15692 (reference strain) | no | podovirus | MZ727332.1 | Yuanyuan et al., 2022 |
| PPAY | ATCC 15692 (reference strain) | yes | podovirus | MZ727202.1 | Yuanyuan et al., 2022 |
| vB_PaeP_YL1 | PA27 (clinical) | no | podovirus | OQ992204 | Zhang et al., 2024 |
| vB_PaeP_YL2 | PA27 (clinical) | no | podovirus | OQ992205 | Zhang et al., 2024 |
| O4 | ATCC 15692 (reference strain) | no data | podovirus | NC_031274.1 | Zhang et al., 2018 |
| vB_PaeP_YZ2 | no data | yes | myovirus | no data | Zhang et al., 2025 |
| vB_PaeP_YQZQ | no data | yes | podovirus | no data | Zhang et al., 2025 |
| vB_PaeP_QSZH | no data | yes | podovirus | no data | Zhang et al., 2025 |

**Supplementary Table S19** – Collected data regarding to phages infecting *P. aeruginosa* in terms of host range and polyvalence.

| **Phage designation** | **Host strain (source of isolation, if given)** | **Host range of the bacteriophage against *P. aeruginosa* strains (vulnerable/tested)** | **Percentage of host range** | **Activity against other species** | **Tested other species (number of tested strains)** | **Reference** |
| --- | --- | --- | --- | --- | --- | --- |
| vB_PaeM_PS3 | PS3 (no data) | 10/18 | 55.55% | no | *E. coli* (4);  *S. enterica* serovar Typhimurium (1);  *S. aureus* (2);  *K. pneumoniae* (1);  *S. marcescens* (1) | Abdelghafar et al., 2023 |
| vB_PaeP_PS28 | PS28 (no data) | 13/18 | 72.22% | no | *E. coli* (4);  *S. enterica* serovar Typhimurium (1);  *S. aureus* (2);  *K. pneumoniae* (1);  *S. marcescens* (1) | Abdelghafar et al., 2023 |
| phPS127 | PS700 (clinical) | 9/12 | 75% | no data |  | Abo Kamer et al., 2022 |
| MA-1 | PA2949 (clinical) | 6/20 | 30% | no data |  | Adnan et al., 2020 |
| BVPaP-3 | HS6 (clinical) | 1/1 | 100% | no | *E. coli* (1);  *P. vulgaris* (1);  *P. fluorescens* (1);  *S. enterica serovar Paratyphi A* (1);  *S. enterica serovar Typhimurium* (1);  *V. cholerae* (1);  *K. pneumoniae* (1);  *E. aerogenes* (1);  *C. koseri* (1);  *S. sonnei* (1) | Ahiwale et al., 2012 |
| Ps12on-D | CN573 (clinical) | 37/140 | 26.43% | no data |  | Akremi et al., 2022 |
| ZC03 | PA14 (clinical) | 3/20 | 15% | no | *B. subtilis* (1);  *C. violaceum* (2);  *E. coli* (1);  *E. faecalis* (1);  *K. pneumoniae* (1);  *S. marcescens* (1);  *S. aureus* (1);  *S. maltophilia* (1);  *X. axonopodis pv. citri* (1) | Amgarten et al., 2017 |
| vB-PaeP-007 | Pae30 (no data) | 47/70 | 67.14% | no | *S. dysenteriae* (1);  *S. sonnei* (2);  *S. flexneri* (1);  *E. coli* (2);  *K. pneumoniae* (1);  *S. pneumoniae* (1);  *E. faecalis* (1);  *S. enterica serovar* Typhimurium (1);  *P. mirabilis* (1);  *S. saprophyticus* (1);  *S. epidermidis* (1);  *S. aureus* (2);  *B. cereus* (1);  *B subtilis* (1);  *E. aerogenes* (1);  *S. pyogenes* (1);  *P. mirabilis* (1) | Barazandeh et al., 2021 |
| PEV2 | PAV237 (animal) | no data | no data | no data |  | Ceyssens et al., 2010 |
| Banzai | PAO1 (clinical) | 13/30 | 43.33% | no data |  | Chaplin et al., 2025 |
| PpY1 | ATCC 15692 (reference strain) | 9/26 | 34.62% | no data |  | Cong et al., 2024 |
| vB_PaeP_ASP23 | L64 (animal) | 16/23 | 69.57% | no | *S. aureus* (1);  *S. abortus* (1);  *E. coli* (1) | Cui et al., 2023 |
| C11 | TJC422 (clinical) | no data | no data | no data |  | Cui et al., 2016 |
| Phage I | 1950 (clinical) | 42/58 | 72.41% | no data |  | Dai et al., 2023 |
| Phage II | 1950 (clinical) | 35/58 | 60.34% | no data |  | Dai et al., 2023 |
| Phage III | 1950 (clinical) | 25/58 | 43.10% | no data |  | Dai et al., 2023 |
| Phage IV | 1950 (clinical) | 28/58 | 48.28% | no data |  | Dai et al., 2023 |
| vB_PaeM_KT28 | ATCC 15692 (reference strain) | 34/58 | 58.62% | no data |  | Danis-Wlodarczyk et al., 2015 |
| vB_PaeM_KTN6 | ATCC 15692 (reference strain) | 39/58 | 67.24% | no data |  | Danis-Wlodarczyk et al., 2015 |
| ϕPSZ1 | no data (clinical) | 7/9 | 77.78% | no | *E. coli* (1);  *S. aureus* (1) | El Didamony et al., 2015 |
| ϕPSZ2 | no data (clinical) | 6/9 | 66.67% | no | *E. coli* (1);  *S. aureus* (1) | El Didamony et al., 2015 |
| PaBG | ATCC 15692 (reference strain) | 20/125 | 16% | no data |  | Evseev et al., 2020 |
| Φ4_ZP1 | MA4 (clinical) | 7/32 | 21.88% | no data |  | Fiscarelli et al., 2021 |
| Φ9_ZP2 | MA4 (clinical) | 7/32 | 21.88% | no data |  | Fiscarelli et al., 2021 |
| Φ14_OBG | MA4 (clinical) | 3/32 | 9.38% | no data |  | Fiscarelli et al., 2021 |
| Φ17_OBG | ATCC 15692 (reference strain) | 5/32 | 15.63% | no data |  | Fiscarelli et al., 2021 |
| Φ19_OBG | ATCC 15692 (reference strain) | 5/32 | 15.63% | no data |  | Fiscarelli et al., 2021 |
| JG024 | ATCC 15692 (reference strain) | 84/100 | 84% | no data |  | Garbe et al., 2010 |
| JG004 | no data (clinical) | 9/19 | 47.37% | no data |  | Garbe et al., 2011 |
| φDCL-PA6 | PA14 (clinical) | 9/11 | 81.81% | no data |  | García-Cruz et al., 2024 |
| vB_PaeM_SCUT-S1 | ATCC 15692 (reference strain) | 6/7 | 85.71% | no data |  | Guo et al., 2019 |
| vB_PaeM_SCUT-S2 | ATCC 15692 (reference strain) | 6/7 | 85.71% | no data |  | Guo et al., 2019 |
| ph0031 | DSM19880 (reference strain) | 9/11 | 81.82% | no | *E. coli* (1);  *S. enterica* (1);  *P. mirabilis* (1);  *E. faecalis* (1);  *B. subtilis* (1);  *S. epidermidis* (1);  *S. aureus* (1);  *A. baumannii* (1);  *K. pneumoniae* (1) | Harada et al., 2022 |
| ph0034 | DSM19880 (reference strain) | 4/11 | 36.36% | no | *E. coli* (1);  *S. enterica* (1);  *P. mirabilis* (1);  *E. faecalis* (1);  *B. subtilis* (1);  *S. epidermidis* (1);  *S. aureus* (1);  *A. baumannii* (1);  *K. pneumoniae* (1) | Harada et al., 2022 |
| FMD5 | no data (animal) | 61/167 | 36.53% | no | *K. pneumoniae* (1);  *E. coli* (1); *Streptococcus* sp. (1); *Pasteurella* sp. (1);  *S. aureus* (1);  *Salmonella* sp. (1) | Hu et al., 2025 |
| H24‑1 | no data (animal) | 94/167 | 56.29% | no | *K. pneumoniae* (1);  *E. coli* (1); *Streptococcus* sp. (1); *Pasteurella* sp. (1);  *S. aureus* (1);  *Salmonella* sp. (1) | Hu et al., 2025 |
| Ka2 | PAO1 (clinical) | 21/30 | 70% | no | *Pseudomonas* spp.;  *E. coli*;  *K. pneumoniae*;  *S. enterica* | Ilyina et al., 2025 |
| MIJ3 | ATCC 15692 (reference strain) | 35/59 | 59.32% | no data |  | Imam et al., 2019 |
| AZ1 | 2995 (clinical) | 4/19 | 21.05% | yes: *E. coli* and *A. xylosoxidans* | *E. coli* (7);  *S. aureus* (3);  *A. xylosoxidans* (1);  *K. pneumoniae* (2);  *E. faecium* (1);  *E. faecalis* (1) | Jamal et al., 2017 |
| Bϕ-R656 | YMC11/02/R656 (clinical) | 18/28 | 64.29% | no data |  | Jeon et al., 2019 |
| Bϕ-R1836 | YMC11/11/R1836 (clinical) | 14/28 | 50% | no data |  | Jeon et al., 2019 |
| PA-YS35 | YS35 (clinical | 21/30 | 70% | no data |  | Jiang et al., 2020 |
| vB_PaeP_TUMS_P121 | ATCC 27853 (reference strain) | no data | no data | no data |  | Kamyab et al., 2022 |
| vB_PaeS_TUMS_P81 | ATCC 27853 (reference strain) | 28/83 | 33.73% | yes: *P. syringae* | *C. freundii* (1);  *E. aerogenes* (1);  *E. faecalis* (1);  *E. coli* (2);  *K. pneumoniae* (1);  *S. aureus* (5);  *P. mirabilis* (1);  *P. putida* (1);  *P. syringae* (1);  *S. enterica* (1);  *S. enterica* serovar Enteritidis (1);  *S. enterica* serovar Typhimurium (1);  *S. enterica* serovar Paratyphi (1);  *S. enterica* serovar Paratyphi C (1);  *S. enterica* serovar Typhi (1);  *S. marcescens* (1);  *S. flexneri* (1);  *S. epidermidis* (1);  *S. saprophyticus* (1) | Kamyab et al., 2023 |
| PhPa‑4 | no data (no data) | 11/18 | 60% | no data |  | Kanwar et al., 2025 |
| PhPa‑6 | no data (no data) | 16/18 | 88% | no data |  | Kanwar et al., 2025 |
| PSPa | ATCC 15692 (reference strain) | 2/2 | 100% | no | *P. otitidis* (1);  *K. aerogenes* (1);  *S. aureus* (1);  *K. pneumoniae* (1);  *A. baumannii* (1);  *E. faecium* (1);  *E. coli* (1);  *S. enterica* serovar Typhi (1);  *P. mirabilis* (1);  *V. alginolyticus* (1);  *C. violaceum* (1) | Karthika et al., 2023 |
| APPa | ATCC 15692 (reference strain) | 2/2 | 100% | no | *P. otitidis* (1);  *K. aerogenes* (1);  *S. aureus* (1);  *K. pneumoniae* (1);  *A. baumannii* (1);  *E. faecium* (1);  *E. coli* (1);  *S. enterica* serovar Typhi (1);  *P. mirabilis* (1);  *V. alginolyticus* (1);  *C. violaceum* (1) | Karthika et al., 2023 |
| vB_Pae-Kakheti25 | PA25 (clinical) | 140/200 | 70% | no | *Klebsiella* sp. (12);  *E. coli* (10);  *Staphylococcus* sp. (11);  *Enterococcus* sp. (12);  *Streptococcus* sp. (15) | Karumidze et al., 2012 |
| vB_Pae-TbilisiM32 | PA32 (clinical) | 140/200 | 70% | no | *Klebsiella* sp. (12);  *E. coli* (10);  *Staphylococcus* sp. (11);  *Enterococcus* sp. (12);  *Streptococcus* sp. (15) | Karumidze et al., 2012 |
| PA1Ø | ATCC 15692 (reference strain) | 9/9 | 100% | yes: *S. sonnei*; *S. aureus*; *S. epidermidis*; *S. hominis*; *S. pneumoniae*; *S. salivarius*; *S. gordonii*; *L. monocytogenes* | *S. sonnei* (7);  *E. coli* (5);  *S. marcescens* (1);  *A. baumannii* (1);  *E. aerogenes* (1);  *S. aureus* (7);  *S. epidermidis* (3);  *S. hominis* (2);  *S. pneumoniae* (3);  *S. salivarius* (4);  *S. gordonii* (1);  *S. agalactiae* (1);  *L. monocytogenes* (1) | Kim et al., 2012 |
| vB_Pae_HB2107-3I | PA14 (clinical) | no data | no data | no data |  | Kong et al., 2023 |
| vB_PaeS_LmqsRe25‑1 | 29 (animal) | 14/33 | 42.42% | no data |  | Köhne et al., 2025 |
| PSA‑KC1 | no data | 17/25 | 68% | no data |  | Kurt et al., 2025 |
| vB_PaeM_MAG1 | Pa6, Pa21, Pa14, Pa18, Pa26 and Pa31/2 (clinical) | 22/37 | 59.46% | no data |  | Kwiatek et al., 2017 |
| vB_PaeP_MAG4 | Pa6, Pa21, Pa14, Pa18, Pa26 and Pa31/2 (clinical) | 20/37 | 54.05% | no data |  | Kwiatek et al., 2017 |
| pPa_SNUABM_DT01 | no data (clinical) | no data | no data | no data |  | Kwon et al., 2021 |
| vB_PaeP_Tr60_Ab31 | PA14 (reference strain) | 7/36 | 19.44% | no data |  | Latino et al., 2014 |
| SL1 | MDR-PA1 (clinical) | no data | no data | no data |  | Latz et al., 2017 |
| SL2 | MDR-PA2 (clinical) | no data | no data | no data |  | Latz et al., 2017 |
| SL4 | MDR-PA4 (clinical) | no data | no data | no data |  | Latz et al., 2017 |
| φKMV | ATCC 15692 (reference strain) | no data | no data | no data |  | Lavigne et al., 2003 |
| vB_PaeS_VL1 | ATCC 27853 (reference strain) | 34/60 | 56.67% | no | *E. coli* (1);  *B. thailandensis* (1);  *S. aureus* (1);  *E. faecalis* (1) | Lerdsittikul et al., 2022 |
| vB_PaP_HN01 | ATCC 27853 (reference strain) | 27/42 | 64.29% | no | *S. aureus* (1);  *B. pseudomallei* (1) | Li et al., 2024 |
| vB_PaP_HN01 | ATCC 27853 (reference strain) | 28/42 | 66.67% | no data |  | Li et al., 2025 |
| vB_PaeP_PZH3 | PA18 (clinical) | 10/18 | 55.56% | no | *A. baumannii* (1);  *K. pneumoniae* (2);  *S. aureus* (5) | Ma et al., 2025 |
| PPaMa1/18 | Isolate 7 (no data) | 12/15 | 80% | no data |  | Majdani et al., 2022 |
| Sem‑1 | ATCC 27853 (reference strain) | 1/1 | 100% | yes | *P. vulgaris*;  *S. enterica* serovar Typhimurium;  *E. coli*;  *Shigella* sp.;  *K. pneumoniae*;  *B. subtilis*;  *B. cereus* | Majlesain et al., 2025 |
| PaB1 | no data (clinical) | 25/40 | 62.50% | no | *A. baumannii* (1);  *E. coli* (1);  *S. aureus* (1);  *E. faecalis* (1);  *S. flexneri* (1) | Marashi et al., 2022 |
| PaBa2 | no data (clinical) | 14/40 | 35% | no | *A. baumannii* (1);  *E. coli* (1);  *S. aureus* (1);  *E. faecalis* (1);  *S. flexneri* (1) | Marashi et al., 2022 |
| PaBa3 | no data (clinical) | 11/40 | 26.50% | no | *A. baumannii* (1);  *E. coli* (1);  *S. aureus* (1);  *E. faecalis* (1);  *S. flexneri* (1) | Marashi et al., 2022 |
| AM.P2 | ATCC 15692 (reference strain) | 1/1 | 100% | no | *E. coli* (2);  *K. pneumoniae* (2);  *A. baumannii* (1);  *S. aureus* (4) | Menon et al., 2021 |
| Pseudomonas phage_AUS034 | AUS34 (clinical) | 6/11 | 54.55% | no | *P. putida* (1) | Namonyo et al., 2022 |
| Pseudomonas phage_AUS260 | AUS260 (clinical) | 5/11 | 45.45% | no | *P. putida* (1) | Namonyo et al., 2022 |
| Pseudomonas phage_AUS301 | AUS301 (clinical) | 7/11 | 63.64% | no | *P. putida* (1) | Namonyo et al., 2022 |
| Pseudomonas phage_AUS391 | AUS391 (clinical) | 7/11 | 63.64% | no | *P. putida* (1) | Namonyo et al., 2022 |
| PAA | PAZMYU (clinical) | 10/25 | 40% | no data |  | Nawaz et al., 2025 |
| PAM | PAZMYU (clinical) | 16/25 | 64% | no data |  | Nawaz et al., 2025 |
| AA17 | no data (clinical) | 14/56 | 25% | no data |  | Nour El‑Din et al., 2025 |
| U17 | no data (clinical) | 9/56 | 16.07% | no data |  | Nour El‑Din et al., 2025 |
| AC20 | no data (clinical) | 12/56 | 21.43% | no data |  | Nour El‑Din et al., 2025 |
| AA20 | no data (clinical) | 33/56 | 58.93% | no data |  | Nour El‑Din et al., 2025 |
| ΦPA01 | ATCC 15692 (reference strain) | 21/58 | 36.21% | no data |  | Ong et al., 2020 |
| ΦPA02 | ATCC 15692 (reference strain) | 27/58 | 46.55% | no data |  | Ong et al., 2020 |
| vB_PAnP_PADP4 | yvu1 (no data) | no data | no data | no data |  | Pallavali et al., 2021 |
| vB_PaeM_CEB_DP1 | ATCC 15692 (reference strain) | 17/30 | 56.67% | no data |  | Pires et al., 2015 |
| vB_PaS-HSN4 | ATCC 15442 (reference strain) | 49/91 | 53.85% | no | *E. faecium* (1);  *S. aureus* (1);  *S. agalactiae* (1);  *S. pyogenes* (1);  *E. coli* (1);  *S. epidermidis* (1);  *S. saprophyticus* (1);  *K. pneumoniae* (1);  *P. mirabilis* (1) | Rafiei et al., 2024 |
| DRL-P1 | MTCC 1688 (reference strain) | 10/10 | 100% | no | *E. coli* (1);  *V. cholerae* (1);  *B. megaterium* (1);  *S. flexneri* (1);  *B. subtilis* (1);  *S. enterica* serovar Typhimurium (1);  *S. pyogenes* (1);  *K. pneumoniae* (1) | Sharma et al., 2021 |
| Phage_Pae01 | Pa021 (animal) | 87/104 | 83.65% | no data |  | Shi et al., 2024 |
| vB_PaeP_LP14 | L7 (environmental) | 7/17 | 41.18% | no data |  | Shi et al., 2020 |
| Pa_WF01 | CRPA (clinical) | no data |  | no | *A. baumannii* (1);  *K. pneumoniae* (2);  *S. aureus* (1);  *E. cloacae* (1);  *E. faecalis* (1) | Sun et al., 2025 |
| vB_PaeM-AL | PA2 (clinical) | 10/17 | 58.82% | no | *K. pneumoniae* (5);  *S. aureus* (4);  *E. faecalis* (3);  *E. coli* (1) | Sutnu et al., 2024 |
| PUTH1 | B‑I‑1 (clinical) | no data |  | no data |  | Tan et al., 2025 |
| PA_LZ01 | ATCC 15692 (reference strain) | no data | no data | no data |  | Wang et al., 2023 |
| PA_LZ02 | PA14 (clinical) | no data | no data | no data |  | Wang et al., 2023 |
| JJ01 | ATCC 15692 (reference strain) | 3/4 | 75% | no | *P. chlororaphis* (1);  *P. stutzeri* (2);  *P. mendocina* (1);  *P. fluorescens* (1);  *P. putida* (2);  *V. cholerae* (1);  *E. coli* (1);  *B. thailandensis* (1);  *A. baumannii* (1) | Wannasrichan et al., 2022 |
| vB_Pae_LC3I3 | PA14 (no data) | no data | no data | no data |  | Xuan et al., 2023 |
| L5 | PAO1r (laboratory strain) | 13/41 | 31.71% | no data |  | Yang et al., 2022 |
| vB_PaeP_Lx18 | Pae-M3 (animal) | 21/33 | 63.64% | no | *Salmonella* sp. (1);  *E. coli* (4);  *S. aureus* (1) | Yin et al., 2022 |
| PX1 | ATCC 15692 (reference strain) | 2/2 | 100% | yes: *P. syringae*, *P. putida* | *P. syringae* (1);  *P. putida* (1);  *E. coli* (1) | Yu et al., 2015 |
| PEf1 | ATCC 15692 (reference strain) | 1/2 | 50% | yes: *P. putida*, *E. coli* | *P. syringae* (1);  *P. putida* (1);  *E. coli* (1) | Yu et al., 2015 |
| PAXYB1 | ATCC 15692 (reference strain) | 14/20 | 70% | no | *E. coli* (1) | Yu et al., 2017 |
| vB_PaeM_LS1 | DLG (animal | 19/23 | 82.61% | no | *A. baumannii* (1);  *E. coli* (1);  *L. monocytogenes* (1);  *S. aureus* (1) | Yuan et al., 2019 |
| PPAT | ATCC 15692 (reference strain) | 1/7 | 14.29% | no data |  | Yuanyuan et al., 2022 |
| PPAY | ATCC 15692 (reference strain) | 1/7 | 14.29% | no data |  | Yuanyuan et al., 2022 |
| vB_PaeP_YL1 | PA27 (clinical) | 67/165 | 40.60% | no | *E. coli* (2);  *Salmonella* sp. (2);  *S. aureus* (1) | Zhang et al., 2024 |
| vB_PaeP_YL2 | PA27 (clinical) | 53/165 | 32.12% | no | *E. coli* (2);  *Salmonella* sp. (2);  *S. aureus* (1) | Zhang et al., 2024 |
| O4 | ATCC 15692 (reference strain) | no data | no data | no data |  | Zhang et al., 2018 |
| vB_PaeP_YZ2 | no data (no data) | 167/246 | 67.89% | no data |  | Zhang et al., 2025 |
| vB_PaeP_YQZQ | no data (no data) | 176/246 | 71.54% | no data |  | Zhang et al., 2025 |
| vB_PaeP_QSZH | no data (no data) | 178/246 | 72.36% | no data |  | Zhang et al., 2025 |

**Supplementary Table S20** – Bacterial strain species used in analyzed studies.

| **Bacterial strain species used in studies** |
| --- |
| *Achromobacter xylosoxidans* |
| *Acinetobacter baumannii* |
| *Bacillus cereus* |
| *Bacillus megaterium* |
| *Bacillus subtilis* |
| *Burkholderia pseudomallei* |
| *Burkholderia thailandensis* |
| *Chromobacterium violaceum* |
| *Citrobacter freundii* |
| *Citrobacter koseri* |
| *Enterobacter aerogenes* |
| *Enterococcus faecalis* |
| *Enterococcus faecium* |
| *Enterococcus* sp. |
| *Escherichia coli* |
| *Klebsiella aerogenes* |
| *Klebsiella pneumoniae* |
| *Klebsiella* sp. |
| *Listeria monocytogenes* |
| *Proteus mirabilis* |
| *Proteus vulgaris* |
| *Pseudomonas aeruginosa* |
| *Pseudomonas chlororaphis* |
| *Pseudomonas fluorescens* |
| *Pseudomonas mendocina* |
| *Pseudomonas otitidis* |
| *Pseudomonas putida* |
| *Pseudomonas* sp. |
| *Pseudomonas stutzeri* |
| *Pseudomonas syringae* |
| *Salmonella abortus* |
| *Salmonella enterica* |
| *Salmonella enterica* serovar Enteritidis |
| *Salmonella enterica* serovar Paratyphi |
| *Salmonella enterica* serovar Paratyphi A |
| *Salmonella enterica* serovar Paratyphi C |
| *Salmonella enterica* serovar Typhi |
| *Salmonella enterica* serovar Typhi |
| *Salmonella enterica* serovar Typhimurium |
| *Salmonella* sp. |
| *Serratia marcescens* |
| *Shigella dysenteriae* |
| *Shigella flexneri* |
| *Shigella sonnei* |
| *Staphylococcus aureus* |
| *Staphylococcus epidermidis* |
| *Staphylococcus hominis* |
| *Staphylococcus saprophyticus* |
| *Staphylococcus* sp. |
| *Stenotrophomonas maltophilia* |
| *Streptococcus agalactiae* |
| *Streptococcus gordonii* |
| *Streptococcus pneumoniae* |
| *Streptococcus pyogenes* |
| *Streptococcus salivarius* |
| *Streptococcus* sp. |
| *Vibrio alginolyticus* |
| *Vibrio cholerae* |
| *Xanthomonas axonopodis pv. Citri* |
