# Supplementary material for: A comprehensive analysis of the kinetics of infection of lytic bacteriophages specific to the ESKAPE and critical pathogens
Source: World J Microbiol Biotechnol. 2026 Feb 28;42(3):110. doi: 10.1007/s11274-025-04762-4 (PMC12950090; doi:10.1007/s11274-025-04762-4)
Supplement: Supplementary file 6 — Supplementary file6 (DOCX 41 KB) [file 11274_2025_4762_MOESM6_ESM.docx]

**Supplementary Table S21** – Collected data regarding to phages infecting *Enterobacter* spp. in terms of cycle parameters.

| **Phage designation** | **Host species** | **Host strain (source of isolation, if given)** | **Results of the studied multiplicity of infection (MOI)** | **Adsorption time [s]** | **Latent period [s]** | **Lysis time [s]** | **Burst size [PFU/cell]** | **Reference** |
| --- | --- | --- | --- | --- | --- | --- | --- | --- |
| vB_EclM_ECLFM1 | *E. cloacae* | ECL73134 (clinical) | 0.001 | 120 | 900 | 1200 | 75 | Ali et al., 2024 |
| EBP | *E. cloacae* | E44 (clinical) | 0.01 | no data | 1200 | no data | 252 | Asif et al., 2021 |
| P.A-5 | *E. hormaechei* | no data (no data) | 10 | no data | 1200 | 4200 | 262 | Chen et al., 2021 |
| Ehp-YZU08 | *E. hormaechei* | Eh-YZU05 (veterinary reference strain) | 10 | no data | 300 | no data | 74 | Chen et al., 2022 |
| Ehp-YZU10 | *E. hormaechei* | Eh-YZU05 (veterinary reference strain) | 10 | no data | 300 | no data | 70 | Chen et al., 2022 |
| Ehp-YZU9-1 | *E. hormaechei* | Eh-YZU05 (veterinary reference strain) | 10 | no data | 2400 | no data | 105 | Chen et al., 2022 |
| Ehp-YZU9-2 | *E. hormaechei* | Eh-YZU05 (veterinary reference strain) | 10 | no data | 1200 | no data | 283 | Chen et al., 2022 |
| Ehp-YZU9-3 | *E. hormaechei* | Eh-YZU05 (veterinary reference strain) | 10 | no data | 1200 | no data | 186 | Chen et al., 2022 |
| IME278 | *E. hormaechei* | *E. hormaechei* 529 (clinical) | 0.1 | no data | 2400 | no data | no data | Cheng et al., 2023 |
| Entb_43 | *E. cloacae* | 30345 (clinical) | 0.1 | 1200 | no data | no data | no data | Cieślik et al., 2022 |
| Entb_45 | *E. cloacae* | 29796 (clinical) | 0.1 | 600 | no data | no data | no data | Cieślik et al., 2022 |
| ФECL22 | *E. cloacae* | 22 (no data) | 0.01 | 900 | 900 | 900 | 19 | Fu et al., 2025 |
| vB_EclS_AS5 | *E. cloacae* | ATCC 13047 (reference strain) | 0.1 | 900 | 1200 | 2100 | 76 | González-Gómez et al., 2024 |
| vB_EclM_AS6 | *E. cloacae* | ATCC 13047 (reference strain) | 0.1 | 900 | 1200 | 2400 | 105 | González-Gómez et al., 2024 |
| vB-Ea-5 | *E. aerogenes* | EIa2 (clinical) | 0.1 | no data | 1500 | 2400 | 13 | Habibinava et al., 2021 |
| vB_ECC_CW742 | *E. hormaechei* | ST 742 (clinical) | 0.1 | no data | 900 | no data | 19 | Imanaka et al., 2025 |
| vB_ECC_MY742 | *E. hormaechei* | ST 742 (clinical) | 0.1 | no data | 900 | no data | 14 | Imanaka et al., 2025 |
| vB_ECC_YI837 | *E. cloacae* | ST 837 (clinical) | 0.1 | no data | 900 | no data | 69 | Imanaka et al., 2025 |
| MJ2 | *E. cloacae* | no data (clinical) | no data | no data | 1260 | no data | 350 | Jamal et al., 2019 |
| phiEap-2 | *E. aerogenes* | 3-SP (clinical) | no data | no data | 1500 | 3600 | 100 | Li et al., 2016 |
| PZJ0206 | *E. cloacae* | ZJ0206 (environmental) | 0.1 | 420 | 1350 | no data | 124 | Li et al., 2022 |
| vB_Ent31 | *E. cloacae* | Ent31 (no data) | 0.01 | no data | 600 | 600 | 99 | Li et al., 2025 |
| vB_EhoP_ZX13 | *E. hormaechei* | Eho13 (no data) | 1 | no data | 300 | no data | 100 | Liu et al., 2025 |
| myPSH1140 | *E. cloacae* | no data (clinical) | 0.001 | no data | 660 | no data | 135 | Manohar et al., 2019 |
| F20 | *E. aerogenes* | ATCC 13048 (reference strain) | no data | no data | 2100 | 5400 | 72 | Mishra et al., 2012 |
| vB_EclM-EP1 | *E. cloacae* | 6AS1 (food) | no data | no data | 1800 | no data | 100 | Nasr-Eldin et al., 2023 |
| vB_EclM-EP2 | *E. cloacae* | 6AS1 (food) | no data | no data | 1800 | no data | 142 | Nasr-Eldin et al., 2023 |
| vB_EclM_HK6 | *E. cloacae* | EC21 (food) | 0.01 | no data | 600 | no data | 115 | Temsaah et al., 2024 |
| EspM4VN | *Enterobacter* spp. | M4 (environmental) | 1 | no data | 1200 | 600 | 122 | Thanh et al., 2020 |
| vB_EaeM_φEap-3 | *E. aerogenes* | 3-SP (clinical) | 0.01 | no data | 600 | 1200 | 109 | Zhao et al., 2019 |
| EcpYZU01 | *E. cloacae* | J01 (environmental) | 10 | no data | 1800 | no data | no data | Zheng et al., 2020 |

**Supplementary Table S22** – Collected data regarding to phages infecting *Enterobacter* spp. in terms of presence of ‘halo’ effect, type of phage morphology, phage gene accesion number.

| **Phage designation** | **Host species** | **Host strain (source of isolation, if given)** | **Presence of 'halo' effect** | **Type of phage morphology** | **Phage gene accesion number** | **Reference** |
| --- | --- | --- | --- | --- | --- | --- |
| vB_EclM_ECLFM1 | *E. cloacae* | ECL73134 (clinical) | no | myovirus | OQ411233 | Ali et al., 2024 |
| EBP | *E. cloacae* | E44 (clinical) | no | myovirus | MT341500 | Asif et al., 2021 |
| P.A-5 | *E. hormaechei* | no data (no data) | no | myovirus | no data | Chen et al., 2021 |
| Ehp-YZU08 | *E. hormaechei* | Eh-YZU05 (veterinary reference strain) | yes | podovirus | no data | Chen et al., 2022 |
| Ehp-YZU10 | *E. hormaechei* | Eh-YZU05 (veterinary reference strain) | yes | podovirus | no data | Chen et al., 2022 |
| Ehp-YZU9-1 | *E. hormaechei* | Eh-YZU05 (veterinary reference strain) | no data | myovirus | no data | Chen et al., 2022 |
| Ehp-YZU9-2 | *E. hormaechei* | Eh-YZU05 (veterinary reference strain) | no data | podovirus | no data | Chen et al., 2022 |
| Ehp-YZU9-3 | *E. hormaechei* | Eh-YZU05 (veterinary reference strain) | no data | podovirus | no data | Chen et al., 2022 |
| IME278 | *E. hormaechei* | *E. hormaechei* 529 (clinical) | yes | siphovirus | MW748991.1 | Cheng et al., 2023 |
| Entb_43 | *E. cloacae* | 30345 (clinical) | no | myovirus | ON585039 | Cieślik et al., 2022 |
| Entb_45 | *E. cloacae* | 29796 (clinical) | no | myovirus | ON630910 | Cieślik et al., 2022 |
| ФECL22 | *E. cloacae* | 22 (no data) | no | myovirus | PQ227830 | Fu et al., 2025 |
| vB_EclS_AS5 | *E. cloacae* | ATCC 13047 (reference strain) | no data | no data | OR753409 | González-Gómez et al., 2024 |
| vB_EclM_AS6 | *E. cloacae* | ATCC 13047 (reference strain) | no data | no data | OR753410 | González-Gómez et al., 2024 |
| vB-Ea-5 | *E. aerogenes* | EIa2 (clinical) | no data | myovirus | no data | Habibinava et al., 2021 |
| vB_ECC_CW742 | *E. hormaechei* | ST 742 (clinical) | no data | myovirus | PV019367 | Imanaka et al., 2025 |
| vB_ECC_MY742 | *E. hormaechei* | ST 742 (clinical) | no data | myovirus | PV593737 | Imanaka et al., 2025 |
| vB_ECC_YI837 | *E. cloacae* | ST 837 (clinical) | no data | myovirus | PV593738 | Imanaka et al., 2025 |
| MJ2 | *E. cloacae* | no data (clinical) | no data | podovirus | no data | Jamal et al., 2019 |
| phiEap-2 | *E. aerogenes* | 3-SP (clinical) | no | siphovirus | KT287080 | Li et al., 2016 |
| PZJ0206 | *E. cloacae* | ZJ0206 (environmental) | no data | podovirus | MT625440 | Li et al., 2022 |
| vB_Ent31 | *E. cloacae* | Ent31 (no data) | no | siphovirus | no data | Li et al., 2025 |
| vB_EhoP_ZX13 | *E. hormaechei* | Eho13 (no data) | no | myovirus | PQ932598 | Liu et al., 2025 |
| myPSH1140 | *E. cloacae* | no data (clinical) | no data | myovirus | MG999954 | Manohar et al., 2019 |
| F20 | *E. aerogenes* | ATCC 13048 (reference strain) | no | siphovirus | JN672684 | Mishra et al., 2012 |
| vB_EclM-EP1 | *E. cloacae* | 6AS1 (food) | yes | myovirus | no data | Nasr-Eldin et al., 2023 |
| vB_EclM-EP2 | *E. cloacae* | 6AS1 (food) | yes | myovirus | no data | Nasr-Eldin et al., 2023 |
| vB_EclM_HK6 | *E. cloacae* | EC21 (food) | no | myovirus | PP337149 | Temsaah et al., 2024 |
| EspM4VN | *Enterobacter* spp. | M4 (environmental) | yes | myovirus | LC373201 | Thanh et al., 2020 |
| vB_EaeM_φEap-3 | *E. aerogenes* | 3-SP (clinical) | no | myovirus | KT321315 | Zhao et al., 2019 |
| EcpYZU01 | *E. cloacae* | J01 (environmental) | yes | podovirus | MK033137 | Zheng et al., 2020 |

**Supplementary Table S23** – Collected data regarding to phages infecting *Enterobacter* spp. in terms of host range and polyvalence.

| **Phage designation** | **Host species** | **Host strain (source of isolation, if given)** | **Host range of the bacteriophage against *Enterobacter* spp. strains (same as host species; vulnerable/tested)** | **Percentage of host range** | **Activity against other species** | **Tested other species (number of tested strains)** | **Reference** |
| --- | --- | --- | --- | --- | --- | --- | --- |
| vB_EclM_ECLFM1 | *E. cloacae* | ECL73134 (clinical) | 96/123 | 78.05% | no | *K. pneumoniae* (8);  *E. coli* (15);  *P. aeruginosa* (10);  *A. baumannii* (12) | Ali et al., 2024 |
| EBP | *E. cloacae* | E44 (clinical) | 12/21 | 57.14% | no | *E. aerogenes* (7);  *C. sakazakii* (9);  *E. coli* (13);  *K. pneumoniae* (11);  *A. baumannii* (6);  *P. aeruginosa* (8);  *S. aureus* (4) | Asif et al., 2021 |
| P.A-5 | *E. hormaechei* | no data (no data) | no data | no data | no data |  | Chen et al., 2021 |
| Ehp-YZU08 | *E. hormaechei* | Eh-YZU05 (veterinary reference strain) | no data | no data | no data |  | Chen et al., 2022 |
| Ehp-YZU10 | *E. hormaechei* | Eh-YZU05 (veterinary reference strain) | no data | no data | no data |  | Chen et al., 2022 |
| Ehp-YZU9-1 | *E. hormaechei* | Eh-YZU05 (veterinary reference strain) | no data | no data | no data |  | Chen et al., 2022 |
| Ehp-YZU9-2 | *E. hormaechei* | Eh-YZU05 (veterinary reference strain) | no data | no data | no data |  | Chen et al., 2022 |
| Ehp-YZU9-3 | *E. hormaechei* | Eh-YZU05 (veterinary reference strain) | no data | no data | no data |  | Chen et al., 2022 |
| IME278 | *E. hormaechei* | *E. hormaechei* 529 (clinical) | 2/2 | 100% | yes: *E. cloacae* | *E. cloacae* (4) | Cheng et al., 2023 |
| Entb_43 | *E. cloacae* | 30345 (clinical) | 6/15 | 40% | yes: *E. hormaechei* | *E. hormaechei* (4);  *E. kobei* (1);  *E. faecalis* (2);  *S. aureus* (2);  *K. pneumoniae* (1);  *A. baumannii* (3);  *P. aeruginosa* (2);  *E. coli* (2) | Cieślik et al., 2022 |
| Entb_45 | *E. cloacae* | 29796 (clinical) | 9/15 | 60% | yes: *E. hormaechei*; *E. kobei* | *E. hormaechei* (4);  *E. kobei* (1);  *E. faecalis* (2);  *S. aureus* (2);  *K. pneumoniae* (1);  *A. baumannii* (3);  *P. aeruginosa* (2);  *E. coli* (2) | Cieślik et al., 2022 |
| ФECL22 | E. cloacae | 22 (no data) | 10/19 | 52.63% | yes: *E. hormaechei* | *E. bugandensis* (1);  *E. hormaechei* (17);  *E. faecalis* (1);  *A. baumannii* (6);  *S. aureus* (3);  *E. coli* (2);  *P. aeruginosa* (2);  *K. pneumoniae* (3) | Fu et al., 2025 |
| vB_EclS_AS5 | *E. cloacae* | ATCC 13047 (reference strain) | 3/3 | 100% | no | *E. hormaechei* (1);  *C. freundii* (1);  *S. sonnei* (1);  *C. braakki* (1) | González-Gómez et al., 2024 |
| vB_EclM_AS6 | *E. cloacae* | ATCC 13047 (reference strain) | 2/3 | 66.67% | yes: all other tested strains | *E. hormaechei* (1);  *C. freundii* (1);  *S. sonnei* (1);  *C. braakki* (1) | González-Gómez et al., 2024 |
| vB-Ea-5 | *E. aerogenes* | EIa2 (clinical) | 11/18 | 61.11% | no | *K. pneumoniae* (1);  *P. aeruginosa* (1);  *S. aureus* (1);  *E. coli* (1);  *Salmonella enterica* serovar Typhi (1) | Habibinava et al., 2021 |
| vB_ECC_CW742 | *E. hormaechei* | ST 742 (clinical) | 77/131 | 58.78% | no data |  | Imanaka et al., 2025 |
| vB_ECC_MY742 | *E. hormaechei* | ST 742 (clinical) | 84/131 | 64.12% | no data |  | Imanaka et al., 2025 |
| vB_ECC_YI837 | *E. cloacae* | ST 837 (clinical) | 61/131 | 46.56% | no data |  | Imanaka et al., 2025 |
| MJ2 | *E. cloacae* | no data (clinical) | 3/10 | 30% | yes: *K. pneumoniae*; *P. aeruginosa*; *A. xylosoxidans* | *S. xylosoxidans* (1);  *K. pneumoniae* (3);  *E. coli* (10);  *P. aeruginosa* (5);  *S. aureus* (3);  *E. faecium* (1);  *E. faecalis* (1) | Jamal et al., 2019 |
| phiEap-2 | *E. aerogenes* | 3-SP (clinical) | 20/27 | 74.07% | no | *E. cloacae* (2);  *C. sakazakii* (2);  *S. marcescens* (5);  *E. coli* (1);  *K. pneumoniae* (1);  *A. xylosoxidans* (1);  *L. adcarboxylata* (1);  *R. ornithinolytica* (1);  *S. maltophilia* (1);  *C. freundii* (1);  *V. parahaemolyticus* (1);  *P. aeruginosa* (1);  *A. baumannii* (1);  *S. sonnei* (1) | Li et al., 2016 |
| PZJ0206 | *E. cloacae* | ZJ0206 (environmental) | no data | no data | no data |  | Li et al., 2022 |
| vB_Ent31 | *E. cloacae* | Ent31 (no data) | 4/4 | 100% | no | *Salmonella* sp. (3);  *E. coli* (4);  *V. parahaemolyticus* (3);  *A. veronii* (1);  *B. subtilis* (1);  *S. aureus* (1);  *P. aeruginosa* (1) | Li et al., 2025 |
| vB_EhoP_ZX13 | *E. hormaechei* | Eho13 (no data) | 10/12 | 83.33% | yes: *E. cloacae*; *E. asburiae* | *E. cloacae* (6);  *E. asburiae* (4);  *K. aerogenes* (2) | Liu et al., 2025 |
| myPSH1140 | *E. cloacae* | no data (clinical) | 11/15 | 73.33% | yes: all other tested strains | *E. hormaechei* (4);  *E. asburiae* (4);  *E. aerogenes* (3) | Manohar et al., 2019 |
| F20 | *E. aerogenes* | ATCC 13048 (reference strain) | 2/2 | 100% | no | *A. baumannii* (1);  *B. cereus* (1);  *B. subtilis subsp. spizizenii* (1);  *E. coli* (3);  *L. monocytogenes* (1);  *P. aeruginosa* (2);  *S. enterica* serovar Enteritidis (1);  *S. enterica* serovar Typhimurium (1);  *S. aureus* (2) | Mishra et al., 2012 |
| vB_EclM-EP1 | *E. cloacae* | 6AS1 (food) | 4/4 | 100% | yes: *A. hydrophila*; *K. pneumoniae* | *Enterobacter* spp. (1);  *P. oryzihabitans* (1);  *P. aeruginosa* (1);  *E. coli* (1);  *A. hydrophila* (1);  *K. pneumoniae* (1);  *S. aureus* (1);  *Streptococcus* spp. (1);  *Proteus* spp. (1);  *Salmonella* spp. (1);  *Shigella* spp. (1) | Nasr-Eldin et al., 2023 |
| vB_EclM-EP2 | *E. cloacae* | 6AS1 (food) | 4/4 | 100% | yes: *A. hydrophila*; *K. pneumoniae* | *Enterobacter* spp. (1);  *P. oryzihabitans* (1);  *P. aeruginosa* (1);  *E. coli* (1);  *A. hydrophila* (1);  *K. pneumoniae* (1);  *S. aureus* (1);  *Streptococcus* spp. (1);  *Proteus* spp. (1);  *Salmonella* spp. (1);  *Shigella* spp. (1) | Nasr-Eldin et al., 2023 |
| vB_EclM_HK6 | *E. cloacae* | EC21 (food) | 8/16 | 50% | no data |  | Temsaah et al., 2024 |
| EspM4VN | *Enterobacter* spp. | M4 (environmental) | 1/5 | 20% | no | *P. dispersa* (1);  *Enterobacter* sp. (5);  *A. baumannii* (2);  *A. junii* (1);  *E. asburiae* (1);  *E. coli* (1);  *P. carotovorum subsp. carotovora* (4);  *P. carotovorum subsp. betavasculorum* (1);  *P. carotovorum subsp. wasabiae* (1);  *P. carotovorum subsp. atroseptica* (1);  *P. chrysanthemi pv. Zeae* (1);  *P. milletiae* (1) | Thanh et al., 2020 |
| vB_EaeM_φEap-3 | *E. aerogenes* | 3-SP (clinical) | 18/28 | 64.29% | no | *E. cloacae* (2);  *C. sakazakii* (2);  *S. marcescens* (5);  *K. pneumoniae* (1);  *L. adecarboxylata* (1);  *R. ornithinolytica* (1);  *C. freundii* (1);  *S. sonnei* (1);  *V. parahaemolyticus* (1);  *E. coli* (1);  *P. aeruginosa* (1);  *A. baumannii* (1);  *A. xylosidans* (1);  *S. maltophilia* (1) | Zhao et al., 2019 |
| EcpYZU01 | *E. cloacae* | J01 (environmental) | 13/13 | 100% | no | *P. mendocina* (7);  *K. pneumoniae* (3);  *P. fluorescens* (8);  *E. vulneris* (1);  *E. coli* (1);  *B. subtilis* (1);  *B. megaterium* (1);  *P. rettgeri* (1);  *V. lutrae* (1);  *M. odoratus* (1);  *S. marcescens* (1);  *L. plantarum* | Zheng et al., 2020 |

**Supplementary Table S24** – Bacterial strain species used in analyzed studies.

| **Bacterial strain species used in studies** |
| --- |
| *Achromobacter xylosoxidans* |
| *Acinetobacter baumanii* |
| *Acinetobacter junii* |
| *Aeromonas hydrophila* |
| *Aeromonas veronii* |
| *Bacillus megaterium* |
| *Bacillus subtilis* |
| *Bacillus subtilis subsp. spizizenii* |
| *Citrobacter braakki* |
| *Citrobacter freundii* |
| *Cronobacter sakazakii* |
| *Enterobacter aerogenes* |
| *Enterobacter asburiae* |
| *Enterobacter cloacae* |
| *Enterobacter hormaechei* |
| *Enterobacter kobei* |
| *Enterobacter* spp. |
| *Enterococcus faecalis* |
| *Escherichia coli* |
| *Escherichia vulneris* |
| *Escherishia cloaca* |
| *Klebsiella aerogenes* |
| *Klebsiella pneumoniae* |
| *Lactobacillus plantarum* |
| *Leclercia adcarboxglata* |
| *Listeria monocytogenes* |
| *Myroides odoratus* |
| *Pantoea dispersa* |
| *Pectobacterium carotovora subsp. Atroseptica* |
| *Pectobacterium carotovora subsp. Betavasculorum* |
| *Pectobacterium carotovora subsp. Wasabiae* |
| *Pectobacterium carotovorum subsp. Carotovora* |
| *Pectobacterium chrysanthemi pv. Zeae* |
| *Pectobacterium milletiae* |
| *Proteus* spp. |
| *Providencia rettgeri* |
| *Pseudomonas aeruginosa* |
| *Pseudomonas fluorescens* |
| *Pseudomonas mendocina* |
| *Pseudomonas oryzihabitans* |
| *Raoultella ornithinolytica* |
| *Salmonella enterica* serovar Enteriditis |
| *Salmonella enterica* serovar Typhi |
| *Salmonella enterica* serovar Typhimurium |
| *Salmonella* spp. |
| *Serratia marcescens* |
| *Shigella sonnei* |
| *Shigella* spp. |
| *Staphylococcus aureus* |
| *Stenotrophomonas maltophilia* |
| *Streptococcus* spp. |
| *Vagococcus lutrae* |
| *Vibrio parahaemolyticus* |
