# Supplementary material for: A comprehensive analysis of the kinetics of infection of lytic bacteriophages specific to the ESKAPE and critical pathogens
Source: World J Microbiol Biotechnol. 2026 Feb 28;42(3):110. doi: 10.1007/s11274-025-04762-4 (PMC12950090; doi:10.1007/s11274-025-04762-4)
Supplement: Supplementary file 7 — Supplementary file7 (DOCX 96 KB) [file 11274_2025_4762_MOESM7_ESM.docx]

**Supplementary Table S25** – Collected data regarding to phages infecting *E. coli* in terms of cycle parameters.

| **Phage designation** | **Host strain (source of isolation if given)** | **Results of the studied multiplicity of infection (MOI)** | **Adsorption time [s]** | **Latent period [s]** | **Lysis time [s]** | **Burst size [PFU/cell]** | **Reference** |
| --- | --- | --- | --- | --- | --- | --- | --- |
| AUBRB02 | ATCC 25922 (reference strain) | no data | 300 | 2700 | 600 | 30 | Abdo Ahmad et al., 2025 |
| ɸEcM-vB1 | no data (clinical) | 0.01 | no data | 300 | no data | 271.72 | Abozahra et al., 2025 |
| vB_EcoM_SCS4 | 753 (clinical) | 1 | 480 | 1200 | no data | 100 | Alexyuk et al., 2022 |
| vB_EcoS_SCS44 | 444 (clinical) | 1 | 480 | 1200 | no data | 100 | Alexyuk et al., 2022 |
| vB_EcoM_SCS57 | 3957 (clinical) | 1 | 480 | 600 | no data | 100 | Alexyuk et al., 2022 |
| vB_EcoS_SCS92 | 3992 (clinical) | 1 | 720 | 1200 | no data | 100 | Alexyuk et al., 2022 |
| vB_EcoS_SCS31 | 4231 (clinical) | 1 | 480 | 600 | no data | 100 | Alexyuk et al., 2022 |
| vB_EcoP_FFK3 | 753 (clinical) | 1 | 720 | 600 | no data | 100 | Alexyuk et al., 2022 |
| vB_EcoM_PL/4 | 4 (animal) | 0.1 | 240 | 1200 | no data | 221 | Alexyuk et al., 2025 |
| vB_Eco_CWW/26 | 26 (animal) | 0.1 | 240 | 1200 | no data | 135 | Alexyuk et al., 2025 |
| vB_EcoM_ShWW/46 | 46 (animal) | 0.1 | 240 | 1200 | no data | 225 | Alexyuk et al., 2025 |
| vB_EcoS_ABO/4 | 4 (animal) | 0.1 | 240 | 1200 | no data | 127 | Alexyuk et al., 2025 |
| phiC119 | 63-Fv18-1 (animal) | 0.01 | no data | 1200 | no data | 210 | Amarillas et al., 2016 |
| phiLLS | O157:H7 CECT 4076 (animal) | 0.001 | no data | 4200 | 720 | 176 | Amarillas et al., 2017 |
| vB_EcoS_K1-ULINTec2 | O18:K1 (animal) | 0.001 | 180 | 750 | no data | no data | Antoine et al., 2021 |
| vB_EcoP_K1-ULINTec4 | O18:K1 (animal) | 0.001 | 180 | 300 | no data | no data | Antoine et al., 2021 |
| vB_EcoP_K1-ULINTec6 | O18:K1 (animal) | 0.001 | 300 | 300 | no data | no data | Antoine et al., 2021 |
| vB_EcoP_K1-ULINTec7 | O18:K1 (animal) | 0.001 | 180 | 300 | no data | no data | Antoine et al., 2021 |
| vB_EcoM_Uniso11 | CEFAR CCCD-E003 (reference strain) | 0.001 | 9000 | 2400 | no data | 106 | Balcão et al., 2022 |
| vB_EcoM_Uniso21 | CEFAR CCCD-E003 (reference strain) | 0.001 | 9000 | 2880 | no data | 10 | Balcão et al., 2022 |
| vB_EcoS-EE09 | DSM613 (reference strain) | 0.001 | no data | 1200 | 600 | 93 | Barrero-Canosa et al., 2023 |
| vB_EcoM_XAM237 | E711 (animal) | 0.01 | 1200 | 1200 | 1800 | 174 | Chai et al., 2025 |
| ɸAPCEc01 | DPC6051 (no data) | 0.001 | no data | 600 | 3600 | 90.3 | Dalmasso et al., 2016 |
| ɸAPCEc02 | DPC6051 (no data) | 0.001 | no data | 600 | 2400 | 30.8 | Dalmasso et al., 2016 |
| ɸAPCEc03 | DPC6051 (no data) | 0.001 | no data | 600 | 2400 | 47.4 | Dalmasso et al., 2016 |
| vB_EcoS_XY3 | MG1655 (clinical) | 0.1 | 360 | 1500 | no data | 750 | Fu et al., 2021 |
| vB_EcoS_XF | 316 (clinical) | 0.01 | no data | 600 | no data | 112 | Fu et al., 2021 |
| vB_EcoS_XY2 | 12938 (clinical) | 0.01 | no data | 1800 | no data | 124 | Fu et al., 2021 |
| vB_Eco_K-02 | no data (clinical) | 0.01 | no data | no data | no data | 552 | García-González et al., 2025 |
| vB_Eco_J-01 | no data (clinical) | 0.01 | no data | no data | no data | 578 | García-González et al., 2025 |
| vB_Eco_K-01 | no data (clinical) | 0.01 | no data | no data | no data | 442 | García-González et al., 2025 |
| vB_Eco_T-01 | no data (clinical) | 0.01 | no data | 600 | 1800 | 987 | García-González et al., 2025 |
| PNJ1809-36 | E. coli K1 (clinical) | 0.01 | no data | 600 | 2400 | 122 | Gong et al., 2021 |
| fBC-Eco01 | #6529 (clinical) | 0.1 | no data | 1800 | no data | 175 | Grami et al., 2023 |
| vB_EcoP-EG1 | MG1655 (clinical) | 10 | no data | 300 | 900 | 152.5 | Gu et al., 2019 |
| KIT06 | NBRC 3972 (reference strain) | 0.01 | 300 | 1200 | no data | 28 | Han et al., 2024 |
| XH12 | Eco‑3 (clinical) | 1 | no data | 1200 | 2400 | no data | Hou et al., 2025 |
| vB_Eco_ZCEC08 | EC‑08 (clinical) | 0.1 | 420 | 900 | 1200 | 900 | Hussein et al., 2025 |
| SPEC13 | ATCC 43890 (reference strain) | 0.001 | 600 | 1200 | no data | 139 | Islam et al., 2024 |
| phiEco273 | VTCCBAA252 | 1 | no data | 1800 | 1200 | 136 | Jaglan et al., 2025 |
| MJ1 | 3 (clinical) | no data | no data | 1260 | no data | 300 | Jamal et al., 2015 |
| vB_Ec_DUEC01 | DU4 (no data) | 0.1 | no data | 1200 | 1200 | 97 | Khalifa & Omar, 2025 |
| ØCJ19 | FC02 (no data) | 0.1 | no data | 300 | 600 | 20 | Kim et al., 2020 |
| ΦCJ20 | BL21 (reference strain) | no data | no data | 2400 | 2400 | 7.5 | Kim et al., 2021 |
| KFS-EC3 | O157:H7 ATCC 10536 (reference strain) | 0.001 | 360 | 1200 | no data | 71 | Kim et al., 2021 |
| LEC10 | ER2738 (reference strain) | 0.001 | no data | 600 | no data | 189 | Kim et al., 2025 |
| LEC2 | NCCP 15961 (reference strain) | 0.001 | no data | 900 | no data | 22 | Kim et al., 2025 |
| 9g | C600 (reference strain) | 0.01 | 900 | 3600 | no data | 400 | Kulikov et al., 2014 |
| KFS-EC | O157:H7 ATCC 43895 (reference strain) | 0.01 | no data | 1800 | no data | 150 | Lee et al., 2020 |
| HY01 | O157:H7 ATCC 43890 (reference strain) | 0.001 | no data | 1500 | no data | 25 | Lee et al., 2016 |
| vB_EcoS-Ro145clw | O145:H28 RM10808 (reference strain) | 0.01 | no data | 1260 | no data | 192 | Liao et al., 2019 |
| vB_EcoP_P64441 | EC1649 (no data) | 1 | no data | 240 | 2160 | 10 | Li et al., 2025 |
| vB_EcoM-4HA13 | O111:NM (clinical) | no data | no data | 5400 | no data | 55 | Lin et al., 2022 |
| P-1 | O157:H7 ATCC 43895 (reference strain) | 0.1 | no data | 1260 | 1620 | 224 | Litt et al., 2017 |
| P-11 | O26 (reference strain) | no data | no data | 1320 | 1740 | 794 | Litt et al., 2018 |
| P-12 | O26 (reference strain) | no data | no data | 900 | 1680 | 48 | Litt et al., 2018 |
| P-13 | O26 (reference strain) | no data | no data | 1140 | 1800 | 12 | Litt et al., 2018 |
| P-14 | O111 (reference strain) | no data | no data | 2100 | 2520 | 288 | Litt et al., 2018 |
| P-16 | O111 (reference strain) | no data | no data | 1260 | 1680 | 49 | Litt et al., 2018 |
| P-17 | O111 (reference strain) | no data | no data | 600 | 1380 | 257 | Litt et al., 2018 |
| P-19 | O103 (reference strain) | no data | no data | 900 | 1860 | 102 | Litt et al., 2018 |
| P-21 | O103 (reference strain) | no data | no data | 780 | 2400 | 68 | Litt et al., 2018 |
| P-22 | O103 (reference strain) | no data | no data | 1920 | 2760 | 13 | Litt et al., 2018 |
| P-8 | O121 (reference strain) | no data | no data | 480 | 1680 | 110 | Litt et al., 2018 |
| J-4 | O121 (reference strain) | no data | no data | 1260 | 2340 | 257 | Litt et al., 2018 |
| J-7 | O121 (reference strain) | no data | no data | 1740 | 2580 | 132 | Litt et al., 2018 |
| P-9 | O45 (reference strain) | no data | no data | 2220 | 2100 | 302 | Litt et al., 2018 |
| J-14 | O45 (reference strain) | no data | no data | 1800 | 2160 | 13 | Litt et al., 2018 |
| J-15 | O45 (reference strain) | no data | no data | 1980 | 1440 | 155 | Litt et al., 2018 |
| J-18 | O145 (reference strain) | no data | no data | 1320 | 1740 | 74 | Litt et al., 2018 |
| J-19 | O145 (reference strain) | no data | no data | 840 | 1680 | 195 | Litt et al., 2018 |
| J-25 | O145 (reference strain) | no data | no data | 1680 | 2340 | 23 | Litt et al., 2018 |
| J-30 | O145 (reference strain) | no data | no data | 1500 | 1980 | 132 | Litt et al., 2018 |
| ST32 | ST130 (clinical) | 0.05 | no data | 3300 | no data | 64 | Liu et al., 2018 |
| ST20 | O165:H8 (clinical) | 0.05 | no data | 2280 | no data | 22 | Liu et al., 2019 |
| KP-BW9 | CREco19 (reference strain) | 0.01 | no data | 1800 | 5400 | 610 | Liu et al., 2025 |
| Φ241 | O157:H7 (environmental) | 0.01 | no data | 900 | no data | 53 | Lu et al., 2015 |
| myPSH1131 | PSH131 (clinical) | 0.001 | 900 | 1200 | no data | 130 | Manohar et al., 2018 |
| myPSH2311 | no data (clinical) | 0.001 | no data | 1560 | no data | 110 | Manohar et al., 2019 |
| ΦK | STEC ATCC 43895 (reference strain) | 0.001 | no data | 600 | 1800 | 427.4 | Na et al., 2025 |
| ΦC | STEC ATCC 43895 (reference strain) | 0.001 | no data | 600 | 1800 | 230 | Na et al., 2025 |
| ΦJ | STEC ATCC 43895 (reference strain) | 0.001 | no data | 600 | 1800 | 157.5 | Na et al., 2025 |
| ΦB | STEC ATCC 43895 (reference strain) | 0.001 | no data | 600 | 1800 | 154.8 | Na et al., 2025 |
| ΦL | STEC ATCC 43895 (reference strain) | 0.001 | no data | 600 | 1800 | 78.7 | Na et al., 2025 |
| vB_Eco4M-7 | ST2–8624 (clinical) | 0.1 | 60 | 600 | no data | 100 | Necel et al., 2020 |
| ECML-117 | ST2–8624 (clinical) | 0.1 | 60 | 600 | no data | 100 | Necel et al., 2020 |
| vB_EcoM_EP57 | O157:H7 (environmental) | 1 | 180 | 1200 | no data | no data | Oluwarinde et al., 2024 |
| vB_EcoM_EP32a | O157:H7 (environmental) | 1 | 180 | 900 | no data | 392 | Oluwarinde et al., 2024 |
| vB_EcoP_EP32b | O157:H7 (environmental) | 1 | 180 | 900 | no data | 200 | Oluwarinde et al., 2024 |
| vB_EcoM_EP-69 | O157:H7 (environmental) | 1 | 180 | 900 | no data | 360 | Oluwarinde et al., 2024 |
| vB_EcoS-BECP10 | NCTC 12079 (reference strain) | 0.1 | 360 | no data | no data | no data | Park et al., 2021 |
| vB_EcoS_HSE2 | 40371 (clinical) | 1 | no data | 1800 | no data | 86 | Peng et al., 2018 |
| phT4A | ATCC 13706 (reference strain) | 0.001 | 4200 | 3000 | no data | 17 | Pereira et al., 2017 |
| ECA2 | ATCC 13706 (reference strain) | 0.001 | 2400 | 1800 | no data | 117 | Pereira et al., 2017 |
| KIT03 | NBRC 3972 (reference strain) | 0.01 | no data | 2100 | no data | 39 | Pham-Khanh et al., 2019 |
| EC200PP | S242 (clinical) | 0.001 | 1200 | 1500 | no data | 10 | Pouillot et al., 2012 |
| vB_EcoS_UTEC10 | EU10 (clinical) | 0.01 | no data | 600 | no data | 198 | Rajab et al., 2024 |
| BPECO 19 | ATCC 43889 (reference strain) | no data | no data | 1500 | no data | 55 | Sadekuzzaman et al., 2017 |
| PhaxI | O157 : H7 B-1 (reference strain) | 1000 | no data | 2400 | no data | 420 | Shahrbabak et al., 2013 |
| PE37 | STEC O157:H7 (clinical) | 0.01 | no data | 900 | no data | 169 | Son et al., 2018 |
| UGKSEcP1 | O157:H7 (clinical) | 0.1 | 123 | 600 | no data | 528 | Ssekatawa et al., 2024 |
| UGKSEcP2 | O157:H7 (clinical) | 0.1 | 123 | 600 | no data | 528 | Ssekatawa et al., 2024 |
| vB_EcoM_swi3 | K88 (animal) | 1 | no data | 1500 | 4500 | 25 | Sui et al., 2021 |
| swi2 | 51 (animal) | 10 | no data | 1500 | 4200 | 1000 | Sui et al., 2021 |
| Ec_MI-02 | O157:H7 NCTC 12900 (reference strain) | 0.1 | 900 | 3000 | no data | 10 | Sultan-Alolama et al., 2023 |
| vB_EcoP-Ro45lw | STEC O45 (environmental) | 0.01 | no data | 900 | no data | 55 | Sun et al., 2022 |
| P206 | EDL933 (clinical) | 0.01 | no data | 3600 | no data | 1000 | Sváb et al., 2018 |
| vB-EcoS-95 | C600 (laboratory strain) | 0.01 | no data | 240 | no data | 115 | Topka et al., 2019 |
| YZ2 | ATCC 25922 (reference strain) | 0.01 | no data | 600 | no data | 30 | Wang et al., 2024 |
| vB_EcoP_E212 | A78 (clinical) | 0.001 | no data | 1200 | no data | 125 | Wei et al., 2023 |
| vB_EcoS_P78 | K88 (reference veterinary strain) | 1 | no data | 1200 | 2400 | 220 | Wen et al., 2025 |
| vB_EcoP_PW8 | MDR APEC PW005 (animal) | 0.1 | 600 | 1200 | no data | 143 | Wintachai et al., 2024 |
| vB_EcoS-B2 | MG1655 (no data) | 10 | no data | 1200 | 600 | 224.1 | Xu et al., 2018 |
| CICC 80001 | CICC 11022S (no data) | 0.1 | no data | 600 | 1200 | 198 | Xu et al., 2016 |
| VB_EcoS-Golestan | 333 (clinical) | 0.01 | 600 | 2400 | no data | 100 | Yazdi et al., 2020 |
| OSYSP | O157:H7 (no data) | 0.01 | no data | 1200 | 3600 | 102 | Yesil et al., 2024 |
| DY1 | 180720 (food) | 0.1 | no data | 1200 | no data | 30 | Yuan et al., 2021 |
| vB_EcoP-Ro103C3lw | RM10744 (animal) | 0.01 | no data | 120 | 960 | 18 | Zhang et al., 2021 |
| vB_EcoM-Pr103Blw | RM10744 (animal) | 0.01 | no data | 900 | 1800 | 152 | Zhang et al., 2021 |
| JP4 | O157:H7 (clinical) | 0.1 | no data | 600 | 1800 | 57 | Zhang et al., 2025 |
| YP6 | MY104 (animal) | 0.1 | no data | 2400 | 3000 | 540 | Zhang et al., 2025 |
| Ec1-7 | M7 (no data) | no data | 300 | 2100 | no data | 41 | Zulkarneev et al., 2025 |

**Supplementary Table S26** – Collected data regarding to phages infecting *E. coli* in terms of presence of ‘halo’ effect, type of phage morphology, phage gene accesion number.

| **Phage designation** | **Host strain (source of isolation if given)** | **Presence of 'halo' effect** | **Type of phage morphology** | **Phage gene accesion number** | **Reference** |
| --- | --- | --- | --- | --- | --- |
| AUBRB02 | ATCC 25922 (reference strain) | no | no data | OY979771 | Abdo Ahmad et al., 2025 |
| ɸEcM‑vB1 | no data (clinical) | no data | myovirus | no data | Abozahra et al., 2025 |
| vB_EcoM_SCS4 | 753 (clinical) | yes | myovirus | ON506924 | Alexyuk et al., 2022 |
| vB_EcoS_SCS44 | 444 (clinical) | yes | siphovirus | OM960734 | Alexyuk et al., 2022 |
| vB_EcoM_SCS57 | 3957 (clinical) | yes | myovirus | ON001686 | Alexyuk et al., 2022 |
| vB_EcoS_SCS92 | 3992 (clinical) | yes | siphovirus | ON548432 | Alexyuk et al., 2022 |
| vB_EcoS_SCS31 | 4231 (clinical) | yes | siphovirus | ON081052 | Alexyuk et al., 2022 |
| vB_EcoP_FFK3 | 753 (clinical) | yes | podovirus | ON548433 | Alexyuk et al., 2022 |
| vB_EcoM_PL/4 | 4 (animal) | yes | myovirus | PV808484 | Alexyuk et al., 2025 |
| vB_Eco_CWW/26 | 26 (animal) | yes | myovirus | PQ900155.1 | Alexyuk et al., 2025 |
| vB_EcoM_ShWW/46 | 46 (animal) | no | myovirus | PV808485 | Alexyuk et al., 2025 |
| vB_EcoS_ABO/4 | 4 (animal) | yes | siphovirus | PV808483 | Alexyuk et al., 2025 |
| phiC119 | 63-Fv18-1 (animal) | no data | siphovirus | KT825490 | Amarillas et al., 2016 |
| phiLLS | O157:H7 CECT 4076 (animal) | no | siphovirus | KY677846.1 | Amarillas et al., 2017 |
| vB_EcoS_K1-ULINTec2 | O18:K1 (animal) | no data | siphovirus | MZ997838 | Antoine et al., 2021 |
| vB_EcoP_K1-ULINTec4 | O18:K1 (animal) | no data | podovirus | MZ997839 | Antoine et al., 2021 |
| vB_EcoP_K1-ULINTec6 | O18:K1 (animal) | no data | podovirus | MZ997840 | Antoine et al., 2021 |
| vB_EcoP_K1-ULINTec7 | O18:K1 (animal) | no data | podovirus | MZ997841 | Antoine et al., 2021 |
| vB_EcoM_Uniso11 | CEFAR CCCD-E003 (reference strain) | no | myovirus | OP557969 | Balcão et al., 2022 |
| vB_EcoM_Uniso21 | CEFAR CCCD-E003 (reference strain) | no | myovirus | OP557970 | Balcão et al., 2022 |
| vB_EcoS-EE09 | DSM613 (reference strain) | yes | siphovirus | OR756193 | Barrero-Canosa et al., 2023 |
| vB_EcoM_XAM237 | E711 (animal) | no | myovirus | no data | Chai et al., 2025 |
| ɸAPCEc01 | DPC6051 (no data) | no data | myovirus | KR422352 | Dalmasso et al., 2016 |
| ɸAPCEc02 | DPC6051 (no data) | no data | myovirus | KR698074 | Dalmasso et al., 2016 |
| ɸAPCEc03 | DPC6051 (no data) | no data | siphovirus | KR422353 | Dalmasso et al., 2016 |
| vB_EcoS_XY3 | MG1655 (clinical) | no | siphovirus | MN781674 | Fu et al., 2021 |
| vB_EcoS_XF | 316 (clinical) | no | siphovirus | MN927225 | Fu et al., 2021 |
| vB_EcoS_XY2 | 12938 (clinical) | no | siphovirus | MN927226 | Fu et al., 2021 |
| vB_Eco_K‑02 | no data (clinical) | no data | no data | PQ435593 | García‑González et al., 2025 |
| vB_Eco_J‑01 | no data (clinical) | no data | no data | PQ436005 | García‑González et al., 2025 |
| vB_Eco_K‑01 | no data (clinical) | no data | no data | PQ438393 | García‑González et al., 2025 |
| vB_Eco_T‑01 | no data (clinical) | no data | no data | PQ438391 | García‑González et al., 2025 |
| PNJ1809-36 | E. coli K1 (clinical) | no data | myovirus | MT944117 | Gong et al., 2021 |
| fBC-Eco01 | #6529 (clinical) | yes | siphovirus | OM272052.1 | Grami et al., 2023 |
| vB_EcoP-EG1 | MG1655 (clinical) | yes | podovirus | MG488277 | Gu et al., 2019 |
| KIT06 | NBRC 3972 (reference strain) | no | myovirus | OQ349392.1 | Han et al., 2024 |
| XH12 | Eco‑3 (clinical) | no | myovirus | PQ327946 | Hou et al., 2025 |
| vB_Eco_ZCEC08 | EC‑08 (clinical) | yes | siphovirus | PP213477 | Hussein et al., 2025 |
| SPEC13 | ATCC 43890 (reference strain) | no | myovirus | no data | Islam et al., 2024 |
| phiEco273 | VTCCBAA252 | no | siphovirus | PP786691.1 | Jaglan et al., 2025 |
| MJ1 | 3 (clinical) | no | myovirus | KF385446 | Jamal et al., 2015 |
| vB_Ec_DUEC01 | DU4 (no data) | no | no data | PQ799475 | Khalifa & Omar, 2025 |
| ØCJ19 | FC02 (no data) | no data | myovirus | MT176427 | Kim et al., 2020 |
| ΦCJ20 | BL21 (reference strain) | no data | myovirus | MT533174 | Kim et al., 2021 |
| KFS-EC3 | O157:H7 ATCC 10536 (reference strain) | no data | myovirus | MZ065353 | Kim et al., 2021 |
| LEC10 | ER2738 (reference strain) | no | myovirus | no data | Kim et al., 2025 |
| LEC2 | NCCP 15961 (reference strain) | no | myovirus | no data | Kim et al., 2025 |
| 9g | C600 (reference strain) | yes | siphovirus | NC_024146.1 | Kulikov et al., 2014 |
| KFS-EC | O157:H7 ATCC 43895 (reference strain) | yes | myovirus | MH560358 | Lee et al., 2020 |
| HY01 | O157:H7 ATCC 43890 (reference strain) | no data | myovirus | KF925357 | Lee et al., 2016 |
| vB_EcoS-Ro145clw | O145:H28 RM10808 (reference strain) | no data | siphovirus | MG852086 | Liao et al., 2019 |
| vB_EcoP_P64441 | EC1649 (no data) | yes | podovirus | no data | Li et al., 2025 |
| vB_EcoM-4HA13 | O111:NM (clinical) | no data | myovirus | MN136198.2 | Lin et al., 2022 |
| P-1 | O157:H7 ATCC 43895 (reference strain) | no | myovirus | no data | Litt et al., 2017 |
| P-11 | O26 (reference strain) | no data | siphovirus | no data | Litt et al., 2018 |
| P-12 | O26 (reference strain) | no data | siphovirus | no data | Litt et al., 2018 |
| P-13 | O26 (reference strain) | no data | myovirus | no data | Litt et al., 2018 |
| P-14 | O111 (reference strain) | no data | siphovirus | no data | Litt et al., 2018 |
| P-16 | O111 (reference strain) | no data | siphovirus | no data | Litt et al., 2018 |
| P-17 | O111 (reference strain) | no data | siphovirus | no data | Litt et al., 2018 |
| P-19 | O103 (reference strain) | no data | myovirus | no data | Litt et al., 2018 |
| P-21 | O103 (reference strain) | no data | myovirus | no data | Litt et al., 2018 |
| P-22 | O103 (reference strain) | no data | myovirus | no data | Litt et al., 2018 |
| P-8 | O121 (reference strain) | no data | myovirus | no data | Litt et al., 2018 |
| J-4 | O121 (reference strain) | no data | myovirus | no data | Litt et al., 2018 |
| J-7 | O121 (reference strain) | no data | myovirus | no data | Litt et al., 2018 |
| P-9 | O45 (reference strain) | no data | tectivirus | no data | Litt et al., 2018 |
| J-14 | O45 (reference strain) | no data | myovirus | no data | Litt et al., 2018 |
| J-15 | O45 (reference strain) | no data | myovirus | no data | Litt et al., 2018 |
| J-18 | O145 (reference strain) | no data | myovirus | no data | Litt et al., 2018 |
| J-19 | O145 (reference strain) | no data | myovirus | no data | Litt et al., 2018 |
| J-25 | O145 (reference strain) | no data | myovirus | no data | Litt et al., 2018 |
| J-30 | O145 (reference strain) | no data | myovirus | no data | Litt et al., 2018 |
| ST32 | ST130 (clinical) | no data | myovirus | MF044458.2 | Liu et al., 2018 |
| ST20 | O165:H8 (clinical) | no | siphovirus | ASH99365.1 | Liu et al., 2019 |
| KP‑BW9 | CREco19 (reference strain) | no | siphovirus | no data | Liu et al., 2025 |
| Φ241 | O157:H7 (environmental) | no | myovirus | no data | Lu et al., 2015 |
| myPSH1131 | PSH131 (clinical) | yes | podovirus | MG983840.1 | Manohar et al., 2018 |
| myPSH2311 | no data (clinical) | no data | siphovirus | MG976803 | Manohar et al., 2019 |
| ΦK | STEC ATCC 43895 (reference strain) | no data | myovirus | MZ868638 | Na et al., 2025 |
| ΦC | STEC ATCC 43895 (reference strain) | no data | myovirus | PQ308253 | Na et al., 2025 |
| ΦJ | STEC ATCC 43895 (reference strain) | no data | myovirus | PQ308254 | Na et al., 2025 |
| ΦB | STEC ATCC 43895 (reference strain) | no data | myovirus | OP114733 | Na et al., 2025 |
| ΦL | STEC ATCC 43895 (reference strain) | no data | myovirus | PQ308255 | Na et al., 2025 |
| vB_Eco4M-7 | ST2–8624 (clinical) | no | myovirus | MN176217 | Necel et al., 2020 |
| ECML-117 | ST2–8624 (clinical) | no | myovirus | JX128258.1 | Necel et al., 2020 |
| vB_EcoM_EP57 | O157:H7 (environmental) | no | myovirus | OR544956 | Oluwarinde et al., 2024 |
| vB_EcoM_EP32a | O157:H7 (environmental) | no | myovirus | OR544954 | Oluwarinde et al., 2024 |
| vB_EcoP_EP32b | O157:H7 (environmental) | no | myovirus | OR544955 | Oluwarinde et al., 2024 |
| vB_EcoM_EP-69 | O157:H7 (environmental) | yes | myovirus | no data | Oluwarinde et al., 2024 |
| vB_EcoS-BECP10 | NCTC 12079 (reference strain) | yes | siphovirus | MW286156.1 | Park et al., 2021 |
| vB_EcoS_HSE2 | 40371 (clinical) | no | siphovirus | MG252615 | Peng et al., 2018 |
| phT4A | ATCC 13706 (reference strain) | no | myovirus | KX130727 | Pereira et al., 2017 |
| ECA2 | ATCC 13706 (reference strain) | yes | podovirus | KX130726 | Pereira et al., 2017 |
| KIT03 | NBRC 3972 (reference strain) | no data | myovirus | AP018932 | Pham-Khanh et al., 2019 |
| EC200PP | S242 (clinical) | no data | podovirus | no data | Pouillot et al., 2012 |
| vB_EcoS_UTEC10 | EU10 (clinical) | no | siphovirus | PP291582 | Rajab et al., 2024 |
| BPECO19 | ATCC 43889 (reference strain) | yes | podovirus | no data | Sadekuzzaman et al., 2017 |
| PhaxI | O157 : H7 B-1 (reference strain) | no data | myovirus | JN673056 | Shahrbabak et al., 2013 |
| PE37 | STEC O157:H7 (clinical) | no data | myovirus | KU925172 | Son et al., 2018 |
| UGKSEcP1 | O157:H7 (clinical) | no data | myovirus | OV877085 | Ssekatawa et al., 2024 |
| UGKSEcP2 | O157:H7 (clinical) | no data | myovirus | OV876900 | Ssekatawa et al., 2024 |
| vB_EcoM_swi3 | K88 (animal) | no data | myovirus | MT768059.1 | Sui et al., 2021 |
| swi2 | 51 (animal) | no | siphovirus | MT768060.1 | Sui et al., 2021 |
| Ec_MI-02 | O157:H7 NCTC 12900 (reference strain) | no | myovirus | OP856590 | Sultan-Alolama et al., 2023 |
| vB_EcoP-Ro45lw | STEC O45 (environmental) | yes | podovirus | MK301532 | Sun et al., 2022 |
| P206 | EDL933 (clinical) | no data | myovirus | MG022440 | Sváb et al., 2018 |
| vB-EcoS-95 | C600 (laboratory strain) | yes | siphovirus | MF564201 | Topka et al., 2019 |
| YZ2 | ATCC 25922 (reference strain) | no | myovirus | no data | Wang et al., 2024 |
| vB_EcoS_P78 | A78 (clinical) | no | siphovirus | no data | Wen et al., 2025 |
| vB_EcoP_E212 | K88 (reference veterinary strain) | no data | myovirus | MZ043897.1 | Wei et al., 2023 |
| vB_EcoP_PW8 | MDR APEC PW005 (animal) | yes | podovirus | PQ362703 | Wintachai et al., 2024 |
| vB_EcoS-B2 | MG1655 (no data) | no data | siphovirus | MG581355 | Xu et al., 2018 |
| CICC 80001 | CICC 11022S (no data) | yes | podovirus | KM242061 | Xu et al., 2016 |
| VB_EcoS-Golestan | 333 (clinical) | no | siphovirus | MG099933.1 | Yazdi et al., 2020 |
| OSYSP | O157:H7 (no data) | no | myovirus | NC_047835.1 | Yesil et al., 2024 |
| DY1 | 180720 (food) | no data | podovirus | MT808983 | Yuan et al., 2021 |
| vB_EcoP-Ro103C3lw | RM10744 (animal) | yes | podovirus | MN067430 | Zhang et al., 2021 |
| vB_EcoM-Pr103Blw | RM10744 (animal) | no data | myovirus | MW481326 | Zhang et al., 2021 |
| JP4 | O157:H7 (clinical) | no | jumbo | PQ330092 | Zhang et al., 2025 |
| YP6 | MY104 (animal) | no | myovirus | OQ376695.1 | Zhang et al., 2025 |
| Ec1‑7 | M7 (no data) | no data | no data | no data | Zulkarneev et al., 2025 |

**Supplementary Table S27** – Collected data regarding to phages infecting *E. coli* in terms of host range and polyvalence.

| **Phage designation** | **Host strain (source of isolation if given)** | **Host range of the bacteriophage against *E. coli* strains (vulnerable/tested)** | **Percentage of host range** | **Activity against other species** | **Tested other species (number of tested strains)** | **Reference** |
| --- | --- | --- | --- | --- | --- | --- |
| AUBRB02 | ATCC 25922 (reference strain) | 9/18 | 50% | no data |  | Abdo Ahmad et al., 2025 |
| ɸEcM‑vB1 | no data (clinical) | 33/65 | 50.77% | yes: *A. baumannii* | *K. pneumoniae* (5);  *A. baumannii* (4);  *P. aeruginosa* (2) | Abozahra et al., 2025 |
| vB_EcoM_SCS4 | 753 (clinical) | 6/9 | 66.66% | no data |  | Alexyuk et al., 2022 |
| vB_EcoS_SCS44 | 444 (clinical) | 3/9 | 33.33% | no data |  | Alexyuk et al., 2022 |
| vB_EcoM_SCS57 | 3957 (clinical) | 6/9 | 66.66% | no data |  | Alexyuk et al., 2022 |
| vB_EcoS_SCS92 | 3992 (clinical) | 3/9 | 33% | no data |  | Alexyuk et al., 2022 |
| vB_EcoS_SCS31 | 4231 (clinical) | 2/9 | 22.22% | no data |  | Alexyuk et al., 2022 |
| vB_EcoP_FFK3 | 753 (clinical) | 3/9 | 33.33% | no data |  | Alexyuk et al., 2022 |
| vB_EcoM_PL/4 | 4 (animal) | 14/35 | 0.4 | no data |  | Alexyuk et al., 2025 |
| vB_Eco_CWW/26 | 26 (animal) | 11/35 | 31.43% | no data |  | Alexyuk et al., 2025 |
| vB_EcoM_ShWW/46 | 46 (animal) | 16/35 | 46.71% | no data |  | Alexyuk et al., 2025 |
| vB_EcoS_ABO/4 | 4 (animal) | 17/35 | 48.57% | no data |  | Alexyuk et al., 2025 |
| phiC119 | 63-Fv18-1 (animal) | 27/35 | 77.14% | yes: *S. enterica* serovar Minnesota; *S. enterica* serovar Luciana; *S. enterica* serovar Oranienburg; *S. enterica* serovar Agona | *S. enterica* serovar Weltevreden (4);  *S. enterica* serovar Oranienburg (11);  *S. enterica* serovar Saintpaul (5);  *S. enterica* serovar Minnesota (2);  *S. enterica* serovar Anatum (2);  *S. enterica* serovar Montevideo (4);  *S. enterica* serovar Luciana (1);  *S. enterica* serovar Agona (2);  *S. enterica* serovar Muenster (2);  *S. enterica* serovar Poona (1);  *S. enterica* serovar Pomona (1);  *S. enterica* serovar Give (5);  *S. enterica* serovar Sandiego (1) | Amarillas et al., 2016 |
| phiLLS | O157:H7 CECT 4076 (animal) | 39/57 | 68.42% | no data |  | Amarillas et al., 2017 |
| vB_EcoS_K1-ULINTec2 | O18:K1 (animal) | 5/21 | 23.81% | no data |  | Antoine et al., 2021 |
| vB_EcoP_K1-ULINTec4 | O18:K1 (animal) | 11/21 | 52.38% | no data |  | Antoine et al., 2021 |
| vB_EcoP_K1-ULINTec6 | O18:K1 (animal) | 9/21 | 42.86% | no data |  | Antoine et al., 2021 |
| vB_EcoP_K1-ULINTec7 | O18:K1 (animal) | 14/21 | 66.67% | no data |  | Antoine et al., 2021 |
| vB_EcoM_Uniso11 | CEFAR CCCD-E003 (reference strain) | 16/24 | 66.67% | no | *S. enterica* (1);  *P. aeruginosa* (1);  *P. mirabilis* (1);  *E. faecalis* (1);  *B. subtilis* (1);  *S. epidermidis* (1);  *S. aureus* (1);  *K. pneumoniae* (2);  *A. baumannii* (1);  *Enterobacter* sp. (1);  *E. aerogenes* (1);  *P. penneri* (1);  *P. vulgaris* (1);  *S. intermedius* (1);  *P. syringae pv. syringae* (1);  *P. syringae pv. garcae* (1);  *X. axopondis pv. citri* (1) | Balcão et al., 2022 |
| vB_EcoM_Uniso21 | CEFAR CCCD-E003 (reference strain) | 16/24 | 66.67% | no | *S. enterica* (1);  *P. aeruginosa* (1);  *P. mirabilis* (1);  *E. faecalis* (1);  *B. subtilis* (1);  *S. epidermidis* (1);  *S. aureus* (1);  *K. pneumoniae* (2);  *A. baumannii* (1);  *Enterobacter* sp. (1);  *E. aerogenes* (1);  *P. penneri* (1);  *P. vulgaris* (1);  *S. intermedius* (1);  *P. syringae pv. syringae* (1);  *P. syringae pv. garcae* (1);  *X. axopondis pv. citri* (1) | Balcão et al., 2022 |
| vB_EcoS-EE09 | DSM613 (reference strain) | 12/17 | 70.59% | no data |  | Barrero-Canosa et al., 2023 |
| vB_EcoM_XAM237 | E711 (animal) | 9/18 | 50% | no data |  | Chai et al., 2025 |
| ɸAPCEc01 | DPC6051 (no data) | 3/16 | 18.75% | yes: *S. sonnei* | *S. sonnei* (1);  *S. enterica* serovar Typhimurium (2) | Dalmasso et al., 2016 |
| ɸAPCEc02 | DPC6051 (no data) | 5/16 | 31.25% | no | *S. sonnei* (1);  *S. enterica* serovar Typhimurium (2) | Dalmasso et al., 2016 |
| ɸAPCEc03 | DPC6051 (no data) | 9/16 | 56.25% | no | *S. sonnei* (1);  *S. enterica* serovar Typhimurium (2) | Dalmasso et al., 2016 |
| vB_EcoS_XY3 | MG1655 (clinical) | 15/30 | 50% | no data |  | Fu et al., 2021 |
| vB_EcoS_XF | 316 (clinical) | 10/24 | 41.67% | no | *S. aureus* (1);  *K. pneumoniae* (1);  *P. aeruginosa* (1);  *P. putida* (1) | Fu et al., 2021 |
| vB_EcoS_XY2 | 12938 (clinical) | 8/24 | 33.33% | no | *S. aureus* (1);  *K. pneumoniae* (1);  *P. aeruginosa* (1);  *P. putida* (1) | Fu et al., 2021 |
| vB_Eco_K‑02 | no data (clinical) | 27/44 | 60% | no data |  | García‑González et al., 2025 |
| vB_Eco_J‑01 | no data (clinical) | 28/44 | 28.88% | no data |  | García‑González et al., 2025 |
| vB_Eco_K‑01 | no data (clinical) | 13/44 | 62.22% | no data |  | García‑González et al., 2025 |
| vB_Eco_T‑01 | no data (clinical) | 30/44 | 66.66% | no data |  | García‑González et al., 2025 |
| PNJ1809-36 | *E. coli* K1 (clinical) | no data | no data | no data |  | Gong et al., 2021 |
| fBC-Eco01 | #6529 (clinical) | 1/7 | 14.29% | yes: *S. enterica* | *P. aeruginosa* (2);  *S. enterica* (1);  *S. enterica* serovar Typhimurium (1);  *S. aureus* (1);  *B. subtilis* (1);  *E. faecalis* (1) | Grami et al., 2023 |
| vB_EcoP-EG1 | MG1655 (clinical) | 20/44 | 45.45% | no data |  | Gu et al., 2019 |
| KIT06 | NBRC 3972 (reference strain) | 7/12 | 58.33% | no data |  | Han et al., 2024 |
| XH12 | Eco‑3 (clinical) | 30/37 | 81.08% | no | *P. aeruginosa*;  *S. enterica* serovar Typhimurium;  *S. aureus* | Hou et al., 2025 |
| vB_Eco_ZCEC08 | EC‑08 (no data) | 4/50 | 8% | Yes: *S. enterica* serovar Blegdam; *S. enterica* serovar Kentucky | *S. enterica* serovar Blegdam (3);  *S. enterica* serovar Kentucky (2);  *S. enterica* serovar Virchom (1);  *S. enterica* serovar Enteritidis (1);  *S. enterica* (2);  *S. enterica* serovar Gallinarum (1) | Hussein et al., 2025 |
| SPEC13 | ATCC 43890 (reference strain) | 35/35 | 100% | no | *S. enterica* serovar Enteritidis (2);  *S. enterica* serovar Typhimurium (2);  *S. aureus* (1);  *L. monocytogenes* (1);  *B. fragilis* (1);  *P. aeruginosa* (1);  *Fusobacterium* sp. | Islam et al., 2024 |
| phiEco273 | VTCCBAA252 (reference strain) | 10/50 | 20% | no data |  | Jaglan et al., 2025 |
| MJ1 | 3 (clinical) | 3/19 | 15.79% | yes: *P. aeruginosa*; *A. xylosidans* | *P. aeruginosa* (7);  *K. pneumoniae* (3);  *A. xylosidans* (1);  *S. aureus* (4) | Jamal et al., 2015 |
| vB_Ec_DUEC01 | DU4 (no data) | 3/8 | 37.5% | no | *K. pneumoniae*;  *P. mirabilis*;  *E. cloacae* | Khalifa & Omar, 2025 |
| ØCJ19 | FC02 (no data) | 3/9 | 33.33% | no data |  | Kim et al., 2020 |
| ΦCJ20 | BL21 (reference strain) | 6/11 | 54.55% | yes: *S. flexneri* | *C. sakazakii* (1);  *S. flexneri* (1);  *S. aureus* (1);  *B. cereus* (1);  *B. atrophaeus* (1);  *L. pentosus* (1);  *L. plantarum* (1);  *L. fermentum* (1);  *L. brevis* (1) | Kim et al., 2021 |
| KFS-EC3 | O157:H7 ATCC 10536 (reference strain) | 3/10 | 30% | yes: *S. sonnei*; *S. enterica* serovar Senftenberg; *S. enterica* serovar Mission; *S. enterica* serovar Enteritidis | *A. hydrophila* (8);  *B. cereus* (4);  *B. subtilis* (1);  *K. pneumoniae* (1);  *L. monocytogenes* (6);  *P. aeruginosa* (1);  *S. enterica* serovar Dublin (1);  *S. enterica* serovar Enteritidis (1);  *S. enterica* serovar Hartford (1);  *S. enterica* serovar Heidelberg (1);  *S. enterica* serovar Mission (1);  *S. enterica* serovar Montevideo (1);  *S. enterica* serovar Newport (1);  *S. enterica* serovar Salamae (1);  *S. enterica* serovar Senftenberg (1);  *S. enterica* serovar Typhi (1);  *S. enterica* serovar Typhimurium (2);  *S. enterica* serovar Panama (1);  *S. boydii* (1);  *S. flexneri* (1);  *S. sonnei* (1);  *S. aureus* (5);  *V. parahaemolyticus* (1);  *V. vulnificus* (1);  *Y. enterocolitica* (3) | Kim et al., 2021 |
| LEC10 | ER2738 (reference strain) | 28/34 | 82.35% | no | *B. cereus* (14);  *C. sakazakii* (4);  *L. monocytogenes* (19);  *S. enterica* serovar Dublin (1);  *S. enterica* serovar Enteritidis (11);  *S. enterica* serovar Hadar (1);  *S. enterica* serovar Infantis (1);  *S. enterica* serovar Paratyphi (2);  *S. enterica* serovar Typhi (2);  *S. enterica* serovar Typhimurium (19);  *S. aureus* (13);  *Y. enterocolitica* (1) | Kim et al., 2025 |
| LEC2 | NCCP 15961 (reference strain) | 30/34 | 88.24% | no | *B. cereus* (14);  *C. sakazakii* (4);  *L. monocytogenes* (19);  *S. enterica* serovar Dublin (1);  *S. enterica* serovar Enteritidis (11);  *S. enterica* serovar Hadar (1);  *S. enterica* serovar Infantis (1);  *S. enterica* serovar Paratyphi (2);  *S. enterica* serovar Typhi (2);  *S. enterica* serovar Typhimurium (19);  *S. aureus* (13);  *Y. enterocolitica* (1) | Kim et al., 2025 |
| 9g | C600 (reference strain) | no data | no data | no data |  | Kulikov et al., 2014 |
| KFS-EC | O157:H7 ATCC 43895 (reference strain) | 6/11 | 54.55% | no | *A. hydrophila* (7);  *A. salmonicida* (2);  *B. cereus* (12);  *B. pumilus* (1);  *B. safensis* (1);  *B. stratosphericus* (1);  *B. subtilis* (1);  *B. toyonensis* (1);  *K. pneumoniae* (1);  *L. innocua* (1);  *L. monocytogenes* (5);  *P. aeruginosa* (1);  *S. enterica* serovar Enteritidis (1);  *S. enterica* serovar Heidelberg (1);  *S. enterica* serovar Hartford (1);  *S. enterica* serovar Mission (1);  *S. enterica* serovar Montevideo (1);  *S. enterica* serovar Newport (1);  *S. enterica* serovar Salama (1);  *S. enterica* serovar Senftenberg (1);  *S. enterica* serovar Typhimurium (1);  *S. boydii* (1);  *S. flexneri* (1);  *S. sonnei* (1);  *S. aureus* (1);  *V. parahaemolyticus* (1);  *Y. enterocolitica* (1) | Lee et al., 2020 |
| HY01 | O157:H7 ATCC 43890 (reference strain) | 5/7 | 71.43% | yes: *S. flexneri* | *S. flexneri* (4);  *S. enterica* serovar Typhimurium (2);  *S. enterica* serovar Enteritidis (1);  *C. sakazakii* (1);  *Y. enterocolitica* (1);  *P. aeruginosa* (1);  *B. cereus* (1);  *B. subtilis* (1);  *L. monocytogenes* (1);  *E. faecalis* (1);  *S. epidermidis* (1);  *S. aureus* (1) | Lee et al., 2016 |
| vB_EcoS-Ro145clw | O145:H28 RM10808 (reference strain) | 6/31 | 19.35% | no | *S. enterica* serovar Montevideo (1);  *S. enterica* serovar Newport (1);  *S. enterica* serovar Heidelberg (1);  *S. enterica* serovar Enteritidis (1);  *S. enterica* serovar Typhimurium (1) | Liao et al., 2019 |
| vB_EcoP_P64441 | EC1649 | 7/23 | 30.43% | no data |  | Li et al., 2025 |
| vB_EcoM-4HA13 | O111:NM (clinical) | 2/22 | 9.09% | no data |  | Lin et al., 2022 |
| P-1 | O157:H7 ATCC 43895 (reference strain) | 50/55 | 90.91% | no data |  | Litt et al., 2017 |
| P-11 | O26 (reference strain) | 1/6 | 16.67% | no data |  | Litt et al., 2018 |
| P-12 | O26 (reference strain) | 1/6 | 16.67% | no data |  | Litt et al., 2018 |
| P-13 | O26 (reference strain) | 1/6 | 16.67% | no data |  | Litt et al., 2018 |
| P-14 | O111 (reference strain) | 2/6 | 33.33% | no data |  | Litt et al., 2018 |
| P-16 | O111 (reference strain) | 2/6 | 33.33% | no data |  | Litt et al., 2018 |
| P-17 | O111 (reference strain) | 2/6 | 33.33% | no data |  | Litt et al., 2018 |
| P-19 | O103 (reference strain) | 1/6 | 16.67% | no data |  | Litt et al., 2018 |
| P-21 | O103 (reference strain) | 1/6 | 16.67% | no data |  | Litt et al., 2018 |
| P-22 | O103 (reference strain) | 1/6 | 16.67% | no data |  | Litt et al., 2018 |
| P-8 | O121 (reference strain) | 2/6 | 33.33% | no data |  | Litt et al., 2018 |
| J-4 | O121 (reference strain) | 2/6 | 33.33% | no data |  | Litt et al., 2018 |
| J-7 | O121 (reference strain) | 2/6 | 33.33% | no data |  | Litt et al., 2018 |
| P-9 | O45 (reference strain) | 1/6 | 16.67% | no data |  | Litt et al., 2018 |
| J-14 | O45 (reference strain) | 1/6 | 16.67% | no data |  | Litt et al., 2018 |
| J-15 | O45 (reference strain) | 1/6 | 16.67% | no data |  | Litt et al., 2018 |
| J-18 | O145 (reference strain) | 3/6 | 50% | no data |  | Litt et al., 2018 |
| J-19 | O145 (reference strain) | 3/6 | 50% | no data |  | Litt et al., 2018 |
| J-25 | O145 (reference strain) | 2/6 | 33.33% | no data |  | Litt et al., 2018 |
| J-30 | O145 (reference strain) | 3/6 | 50% | no data |  | Litt et al., 2018 |
| ST32 | ST130 (clinical) | 28/60 | 46.67% | yes: *S. sonnei* | *S. sonnei* (1);  *S. dysenteriae* (2);  *S. enterica* serovar Paratyphi (1);  *S. enterica* serovar Typhi (1);  *C. freundii* (8) | Liu et al., 2018 |
| ST20 | O165:H8 (clinical) | no data | no data | no data |  | Liu et al., 2019 |
| KP‑BW9 | CREco19 | 8/18 | 44.44% | yes: *K. pneumoniae* | *K. pneumoniae* (18); *S. aureus* (6) | Liu et al., 2025 |
| Φ241 | O157:H7 (environmental) | 48/64 | 75% | no data |  | Lu et al., 2015 |
| myPSH1131 | PSH131 (clinical) | 31/53 | 58.49% | no data |  | Manohar et al., 2018 |
| myPSH2311 | no data (clinical) | 38/80 | 47.50% | no data |  | Manohar et al., 2019 |
| ΦK | STEC ATCC 43895 (reference strain) | 10/27 | 37.04% | yes: *E. fergusonii* | *E. fergusonii*; *E. hermannii* | Na et al., 2025 |
| ΦC | STEC ATCC 43895 (reference strain) | 13/27 | 41.15% | yes: *E. fergusonii* | *E. fergusonii*; *E. hermannii* | Na et al., 2025 |
| ΦJ | STEC ATCC 43895 (reference strain) | 13/27 | 41.15% | yes: *E. fergusonii* | *E. fergusonii*; *E. hermannii* | Na et al., 2025 |
| ΦB | STEC ATCC 43895 (reference strain) | 14/27 | 51.85% | yes: *E. fergusonii* | *E. fergusonii*; *E. hermannii* | Na et al., 2025 |
| ΦL | STEC ATCC 43895 (reference strain) | 16/27 | 59.26% | yes: *E. fergusonii* | *E. fergusonii*; *E. hermannii* | Na et al., 2025 |
| vB_Eco4M-7 | ST2–8624 (clinical) | 39/87 | 44.83% | no | *S. flexneri* (1);  *S. enterica* serovar Anatum (1);  *S. enterica* serovar Heidelberg (1);  *S. enterica* serovar Reading (1);  *S. enterica* serovar Panama (1);  *Bacillus* sp. (1);  *P. aeruginosa* (2);  *E. faecalis* (2);  *E. faecium* (2);  *S. aureus* (2);  *Klebsiella* sp. (1); *Acinetobacter* sp*.* (1) | Necel et al., 2020 |
| ECML-117 | ST2–8624 (clinical) | 34/87 | 39.08% | no | *S. flexneri* (1);  *S. enterica* serovar Anatum (1);  *S. enterica* serovar Heidelberg (1);  *S. enterica* serovar Reading (1);  *S. enterica* serovar Panama (1);  *Bacillus* sp. (1);  *P. aeruginosa* (2);  *E. faecalis* (2);  *E. faecium* (2);  *S. aureus* (2);  *Klebsiella* sp. (1); *Acinetobacter* sp*.* (1) | Necel et al., 2020 |
| vB_EcoM_EP57 | O157:H7 (environmental) | 8/9 | 88.89% | yes: *S. aureus*; *P. aeruginosa* | *S. enterica* serovar Typhi (1);  *Campylobacter* sp. (1);  *V. cholerae* (1); *Staphylococcus* sp. (4);  *Brevibacterium* sp. (1);  *K. pneumoniae* (2);  *P. aeruginosa* (2);  *S. aureus* (2);  *P. multocida* (1) | Oluwarinde et al., 2024 |
| vB_EcoM_EP32a | O157:H7 (environmental) | 7/9 | 77.78% | yes: *Brevibacterium* sp.; *Staphylococcus* sp. | *S. enterica* serovar Typhi (1);  *Campylobacter* sp. (1);  *V. cholerae* (1); *Staphylococcus* sp. (4);  *Brevibacterium* sp. (1);  *K. pneumoniae* (2);  *P. aeruginosa* (2);  *S. aureus* (2);  *P. multocida* (1) | Oluwarinde et al., 2024 |
| vB_EcoP_EP32b | O157:H7 (environmental) | 7/9 | 77.78% | no | *S. enterica* serovar Typhi (1);  *Campylobacter* sp. (1);  *V. cholerae* (1); *Staphylococcus* sp. (4);  *Brevibacterium* sp. (1);  *K. pneumoniae* (2);  *P. aeruginosa* (2);  *S. aureus* (2);  *P. multocida* (1) | Oluwarinde et al., 2024 |
| vB_EcoM_EP-69 | O157:H7 (environmental) | 7/9 | 77.78% | yes: *S. aureus*; *V. cholerae* | *S. enterica* serovar Typhi (1);  *Campylobacter* sp. (1);  *V. cholerae* (1); *Staphylococcus* sp. (4);  *Brevibacterium* sp. (1);  *K. pneumoniae* (2);  *P. aeruginosa* (2);  *S. aureus* (2);  *P. multocida* (1) | Oluwarinde et al., 2024 |
| vB_EcoS-BECP10 | NCTC 12079 (reference strain) | 11/36 | 30.56% | no | *S. enterica* serovar Enteritidis (1);  *S. enterica* serovar Typhimurium (2);  *S. flexneri* (1);  *S. boydii* (1);  *V. parahaemolyticus* (1);  *C. sakazakii* (1);  *S. aureus* (1);  *B. cereus* (1) | Park et al., 2021 |
| vB_EcoS_HSE2 | 40371 (clinical) | 6/11 | 54.55% | no | *Klebsiella* spp. (1);  *P. aeruginosa* (2);  *S. aureus* (1);  *N. gonorrhoeae* (1);  *S. enterica* serovar Typhimurium (1);  *B. cereus* (1) | Peng et al., 2018 |
| phT4A | ATCC 13706 (reference strain) | 6/11 | 54.55% | yes: *S. enterica* serovar Typhimurium; *S. enterica* serovar Enteritidis; *C. freundii*; *P. vermicola*; *Providencia sp.*; *P. vulgaris*; *P. mirabilis*; *E. cloacae* | *S. enterica* serovar Typhimurium (2);  *S. enterica* serovar Enteritidis (5);  *S. flexneri* (1);  *C. freundii* (2);  *P. vermicola* (1);  *Providencia* sp. (1);  *P. vulgaris* (1);  *P. mirabilis* (1);  *K. pneumoniae* (1);  *E. cloacae* (1);  *L. innocua* (1);  *L. monocytogenes* (1);  *V. parahaemolyticus* (1);  *V. anguillarum* (1);  *V. fischeri* (1);  *P. damselae damselae* (1);  *A. hydrophilla* (1);  *A. salmonicida* (1);  *P. aeruginosa* (1);  *P. fluorescens* (1);  *P. putida* (1);  *P. segetis* (1);  *P. gingeri* (1) | Pereira et al., 2017 |
| ECA2 | ATCC 13706 (reference strain) | 6/11 | 54.55% | yes: *S. enterica* serovar Typhimurium; *S. enterica* serovar Enteritidis; *C. freundii*; *P. vermicola*; *Providencia sp.*; *P. vulgaris*; *P. mirabilis*; *E. cloacae* | *S. enterica* serovar Typhimurium (2);  *S. enterica* serovar Enteritidis (5);  *S. flexneri* (1);  *C. freundii* (2);  *P. vermicola* (1);  *Providencia* sp. (1);  *P. vulgaris* (1);  *P. mirabilis* (1);  *K. pneumoniae* (1);  *E. cloacae* (1);  *L. innocua* (1);  *L. monocytogenes* (1);  *V. parahaemolyticus* (1);  *V. anguillarum* (1);  *V. fischeri* (1);  *P. damselae damselae* (1);  *A. hydrophilla* (1);  *A. salmonicida* (1);  *P. aeruginosa* (1);  *P. fluorescens* (1);  *P. putida* (1);  *P. segetis* (1);  *P. gingeri* (1) | Pereira et al., 2017 |
| KIT03 | NBRC 3972 (reference strain) | 4/7 | 57.14% | yes: *S. enterica* serovar Choleraesuis; *S. enterica* serovar Enteritidis | *S. enterica* serovar Choleraesuis (1);  *S. enterica* serovar Enteritidis (1);  *S. enterica* serovar Minnesota (1);  *S. enterica* serovar Typhimurium (7) | Pham-Khanh et al., 2019 |
| EC200PP | S242 (clinical) | 7/63 | 11.11% | no data |  | Pouillot et al., 2012 |
| vB_EcoS_UTEC10 | EU10 (clinical) | 22/30 | 73.33% | no | *S. enterica* serovar Typhimurium (1);  *K. pneumoniae* (1);  *S. aureus* (2);  *P. aeruginosa* (3) | Rajab et al., 2024 |
| BPECO19 | ATCC 43889 (reference strain) | 3/3 | 100% | no | *B. cereus* (1);  *L. monocytogenes* (1);  *S. aureus* (1);  *S. eneterica* serovar Enteritidis (1);  *S. enterica* serovar Typhimurium (1);  *V. parahaemolyticus* (1);  *A. hydrophila* (1);  *P. aeruginosa* (1) | Sadekuzzaman et al., 2017 |
| PhaxI | O157 : H7 B-1 (reference strain) | 6/28 | 21.43% | no | *E. aerogenes* (1);  *E. faecalis* (1);  *K. pneumoniae* (1);  *P. mirabilis* (1);  *S. enterica* serovar Typhi (1);  *S. marcescens* (1);  *S. dysenteriae* (1) | Shahrbabak et al., 2013 |
| PE37 | STEC O157:H7 (clinical) | 65/114 | 57.02% | no | *S. enterica* serovar Aarhus (1);  *S. enterica* serovar Agona (1);  *S. enterica* serovar Anatum (1);  *S. enterica* serovar Braenderup (1);  *S. enterica* serovar Derby (1);  *S. enterica* serovar Hadar (1);  *S. enterica* serovar Heidelberg (1);  *S. enterica* serovar Istanbul (1);  *S. enterica* serovar Infantis (1);  *S. enterica* serovar Litchfield (1);  *S. enterica* serovar London (1);  *S. enterica* serovar Montevideo (1);  *S. enterica* serovar Muenster (1);  *S. enterica* serovar Scharzengrund (1);  *S. enterica* serovar Stanley (1);  *S. enterica* serovar Thompson (1);  *S. enterica* serovar Enteritidis (1);  *S. enterica* serovar Typhimurium (1) | Son et al., 2018 |
| UGKSEcP1 | O157:H7 (clinical) | 8/9 | 88.89% | yes: *K. pneumoniae* | *K. pneumoniae* (3) | Ssekatawa et al., 2024 |
| UGKSEcP2 | O157:H7 (clinical) | 8/9 | 88.89% | yes: *K. pneumoniae* | *K. pneumoniae* (3) | Ssekatawa et al., 2024 |
| vB_EcoM_swi3 | K88 (animal) | 6/65 | 9.23% | yes: *S. enterica* serovar Enteritidis | *S. enterica* serovar Enteritidis (72) | Sui et al., 2021 |
| swi2 | 51 (animal) | 8/11 | 72.73% | no data |  | Sui et al., 2021 |
| Ec_MI-02 | O157:H7 NCTC 12900 (reference strain) | 14/16 | 87.50% | no | *B. subtilis* (1);  *P. aeruginosa* (2);  *S. aureus* (3);  *S. epidermidis* (1);  *S. saprophyticus* (1);  *S. pyogenes* (1);  *E. faecalis* (2);  *E. casseliflavus* (1);  *E. aerogenes* (1);  *E. hormaechei* (1);  *K. pneumoniae* (2);  *H. influenzae* (1);  *S. maltophilia* (1);  *S. enterica* (1);  *Salmonella* sp. (1);  *P. vulgaris* (1);  *M. smegmatis* (1) | Sultan-Alolama et al., 2023 |
| vB_EcoP-Ro45lw | STEC O45 (environmental) | 4/8 | 50% | no | *S. enterica* serovar Newport (1);  *S. enterica* serovar Enteritidis (1);  *S. enterica* serovar Typhimurium (1) | Sun et al., 2022 |
| P206 | EDL933 (clinical) | no data | no data | no data |  | Sváb et al., 2018 |
| vB-EcoS-95 | C600 (laboratory strain) | 9/17 | 52.94% | no | *S. flexneri* (1);  *S. enterica* serovar Anatum (1);  *S. enterica* serovar Heidelberg (1);  *S. enterica* serovar Reading (1);  *S. enterica* serovar Panama (1);  *P. aeruginosa* (2);  *E. faecalis* (1);  *S. sciuri* (1) | Topka et al., 2019 |
| YZ2 | ATCC 25922 (reference strain) | no data | no data | no data |  | Wang et al., 2024 |
| vB_EcoS_P78 | A78 (clinical) | 6/8 | 75% | no data |  | Wen et al., 2025 |
| vB_EcoP_E212 | K88 (reference veterinary strain) | 1/14 | 7.14% | no | *S. enterica* serovar Pullorum (1);  *S. enterica* serovar Enteritidis (1);  *S. enterica* serovar Choleraesuls (1);  *S. enterica* serovar Typhimurium (1);  *S. sonnei* (1);  *S. dysenteriae* (1);  *S. boydii* (1);  *S. flexneri* (1) | Wei et al., 2023 |
| vB_EcoP_PW8 | MDR APEC PW005 (animal) | 11/20 | 55% | no data |  | Wintachai et al., 2024 |
| vB_EcoS-B2 | MG1655 (no data) | 13/44 | 29.55% | no data |  | Xu et al., 2018 |
| CICC 80001 | CICC 11022S (no data) | 3/19 | 15.79% | yes: *S. dysenteriae*; *S. sonnei*; *S. enterica subsp. enterica serovartyphi* | *S. dysenteriae* (1);  *S. sonnei* (1);  *S. bogdii* (1);  *S. enterica subsp.* *enterica serovartyphi* (1);  *S. enterica* serovar Typhimurium (1) | Xu et al., 2016 |
| VB_EcoS-Golestan | 333 (clinical) | 23/41 | 56.10% | no data |  | Yazdi et al., 2020 |
| OSYSP | O157:H7 (no data) | 4/5 | 80% | yes: *S. enterica* serovar Typhimurium; *S. enterica* serovar Tennessee; *S. enterica* serovar Montevideo; *S. enterica* serovar Javiana | *S. enterica* serovar Senftenberg *(1);*  *S. enterica* serovar Saintpaul *(1);*  *S. enterica* serovar Enteritidis *(1);*  *S. enterica* serovar Kentucky *(1);*  *S. enterica* serovar Typhimurium *(3);*  *S. enterica* serovar Tennessee *(1);*  *S. enterica* serovar Poona *(1);*  *S. enterica* serovar Montevideo *(1);*  *S. enterica* serovar Javiana *(1);*  *S. enterica* serovar Eimsbuettel *(1)* | Yesil et al., 2024 |
| DY1 | 180720 (food) | 1/75 | 1.33% | no data |  | Yuan et al., 2021 |
| vB_EcoP-Ro103C3lw | RM10744 (animal) | 3/17 | 17.65% | no | *S. enterica* serovar Agona (1);  *S. enterica* serovar Anatum (1);  *S. enterica* serovar Berta (1);  *S. enterica* serovar Gallinarum (1);  *S. enterica* serovar Infantis (1);  *S. enterica* serovar Javiana (1);  *S. enterica* serovar Mbandaka (1);  *S. enterica* serovar Oranienburg (1);  *S. enterica* serovar Derby (1);  *S. enterica* serovar Dublin (1);  *S. enterica* serovar Montevideo (1);  *S. enterica* serovar Muenster (1);  *S. enterica* serovar Newport (1);  *S. enterica* serovar Saintpaul (1);  *S. enterica* serovar Thompson (1);  *S. enterica* serovar Typhimurium (2) | Zhang et al., 2021 |
| vB_EcoM-Pr103Blw | RM10744 (animal) | 8/17 | 47.06% | yes: *S. enterica* serovar Javiana | *S. enterica* serovar Agona (1);  *S. enterica* serovar Anatum (1);  *S. enterica* serovar Berta (1);  *S. enterica* serovar Gallinarum (1);  *S. enterica* serovar Infantis (1);  *S. enterica* serovar Javiana (1);  *S. enterica* serovar Mbandaka (1);  *S. enterica* serovar Oranienburg (1);  *S. enterica* serovar Derby (1);  *S. enterica* serovar Dublin (1);  *S. enterica* serovar Montevideo (1);  *S. enterica* serovar Muenster (1);  *S. enterica* serovar Newport (1);  *S. enterica* serovar Saintpaul (1);  *S. enterica* serovar Thompson (1);  *S. enterica* serovar Typhimurium (2) | Zhang et al., 2021 |
| JP4 | O157:H7 (clinical) | 3/68 | 4.42% | no | *S. dysenteriae*;  *S. enterica* serovar Typhi;  *S. aureus* | Zhang et al., 2025 |
| YP6 | MY104 (animal) | 94/126 | 74.60% | no data |  | Zhang et al., 2025 |
| Ec1‑7 | M7 (no data) | 11/14 | 78.57% | no data |  | Zulkarneev et al., 2025 |

**Supplementary Table S28** – Bacterial strain species used in analyzed studies.

| **Bacterial strain species used in studies** |
| --- |
| *Acinetobacter* sp. |
| *Aeromonas hydrophila* |
| *Aeromonas salmonicida* |
| *Bacillus atrophaeus* |
| *Bacillus cereus* |
| *Bacillus pumilus* |
| *Bacillus safensis* |
| *Bacillus* sp. |
| *Bacillus stratosphericus* |
| *Bacillus subtilis* |
| *Bacillus toyonensis* |
| *Bacteroides fragilis* |
| *Brevibacterium* sp. |
| *Campylobacter* sp. |
| *Citrobacter freundii* |
| *Cronobacter sakazakii* |
| *Enterobacter aerogenes* |
| *Enterobacter hormaechei* |
| *Enterococcus casseliflavus* |
| *Enterococcus faecalis* |
| *Enterococcus faecium* |
| *Escherichia coli* |
| *Escherichia fergusonii* |
| *Escherichia hermanni* |
| *Fusobacterium* sp. |
| *Haemophilus influenzae* |
| *Klebsiella pneumoniae* |
| *Lactobacillus brevis* |
| *Lactobacillus fermentum* |
| *Lactobacillus pentosus* |
| *Lactobacillus plantarum* |
| *Listeria innocua* |
| *Listeria monocytogenes* |
| *Mycobacterium smegmatis* |
| *Neisseria gonorrhoeae* |
| *Pasteurella multocida* |
| *Photobacterium damselae damselae* |
| *Proteus mirabilis* |
| *Proteus penneri* |
| *Proteus vulgaris* |
| *Providencia* sp. |
| *Providencia vermicola* |
| *Pseudomonas aeruginosa* |
| *Pseudomonas fluorescens* |
| *Pseudomonas gingeri* |
| *Pseudomonas putida* |
| *Pseudomonas segetis* |
| *Pseudomonas syringae pv. Garcae* |
| *Pseudomonas syringae pv. Syringae* |
| *Salmonela enterica serovar Aarhus* |
| *Salmonella enterica* |
| *Salmonella enterica Eimsbuettel* |
| *Salmonella enterica* serovar Agona |
| *Salmonella enterica* serovar Anatum |
| *Salmonella enterica* serovar Bareilly |
| *Salmonella enterica* serovar Berta |
| *Salmonella enterica* serovar Blegdam |
| *Salmonella enterica* serovar Braenderup |
| *Salmonella enterica* serovar Choleraesuls |
| *Salmonella enterica* serovar Derby |
| *Salmonella enterica* serovar Dublin |
| *Salmonella enterica* serovar Enteriditis |
| *Salmonella enterica* serovar Gallinarum |
| *Salmonella enterica* serovar Give |
| *Salmonella enterica* serovar Hadar |
| *Salmonella enterica* serovar Hartford |
| *Salmonella enterica* serovar Heidelberg |
| *Salmonella enterica* serovar Infantis |
| *Salmonella enterica* serovar Java |
| *Salmonella enterica* serovar Javiana |
| *Salmonella enterica* serovar Kent |
| *Salmonella enterica* serovar Kentucky |
| *Salmonella enterica* serovar Litchfield |
| *Salmonella enterica* serovar London |
| *Salmonella enterica* serovar Luciana |
| *Salmonella enterica* serovar Mbandaka |
| *Salmonella enterica* serovar Minnesota |
| *Salmonella enterica* serovar Mission |
| *Salmonella enterica* serovar Montevideo |
| *Salmonella enterica* serovar Muenster |
| *Salmonella enterica* serovar Newport |
| *Salmonella enterica* serovar Oranienburg |
| *Salmonella enterica* serovar Panama |
| *Salmonella enterica* serovar Paratyphi |
| *Salmonella enterica* serovar Pomona |
| *Salmonella enterica* serovar Poona |
| *Salmonella enterica* serovar Pullorum |
| *Salmonella enterica* serovar Reading |
| *Salmonella enterica* serovar Saintpaul |
| *Salmonella enterica* serovar Salama |
| *Salmonella enterica* serovar Salamae |
| *Salmonella enterica* serovar Sandiego |
| *Salmonella enterica* serovar Senftenberg |
| *Salmonella enterica* serovar Stanley |
| *Salmonella enterica* serovar Tennessee |
| *Salmonella enterica* serovar Thompson |
| *Salmonella enterica* serovar Typhi |
| *Salmonella enterica* serovar Typhimurium |
| *Salmonella enterica* serovar Virchom |
| *Salmonella enterica* serovar Weltevreden |
| *Salmonella enterica subsp. enterica serovartyphi* |
| *Salmonella* sp. |
| *Serratia marcescens* |
| *Shigella boydii* |
| *Shigella dysenteriae* |
| *Shigella flexneri* |
| *Shigella sonnei* |
| *Staphylococcus aureus* |
| *Staphylococcus epidermidis* |
| *Staphylococcus intermedius* |
| *Staphylococcus saprophyticus* |
| *Staphylococcus sciuri* |
| *Staphylococcus sp.* |
| *Stenotrophomonas maltophilia* |
| *Streptococcus pyogenes* |
| *Vibrio anguillarum* |
| *Vibrio cholerae* |
| *Vibrio fischeri* |
| *Vibrio parahaemolyticus* |
| *Vibrio vulnificus* |
| *Xanthomonas axonopodis pv. Citri* |
| *Yersinia enterocolitica* |
